# A Predictive Model for Thiol Reactivity of *N*‑Heteroaryl α‑Methylene−γ-Lactams—A Medicinally Relevant Covalent Reactive Group

**DOI:** 10.1021/acs.jmedchem.5c00833

**Published:** 2025-05-23

**Authors:** Mariah C. Meehan, Grace E. Scofield, Corrinne E. Stahl, Jacob A. Wolfe, W. Seth Horne, Peng Liu, Kay M. Brummond

**Affiliations:** Department of Chemistry, 6614 University of Pittsburgh, Pittsburgh, Pennsylvania 15260, United States

## Abstract

Herein, we present a systematic study on the effects of electronically diverse heteroarenes on the rate of glutathione (GSH) addition to novel *N*-heteroaryl α-methylene−γ-lactam covalent reactive groups (CRGs). Despite their unique electronic and drug-like properties, heteroarenes have not been extensively studied as handles for systematically tuning the reactivity of CRGs. Informed by mechanistic insights, we evaluated 16 substrate parameters, including a new heteroaryl Hammett-type substituent constant (σ_Het_), for their correlation with experimental reactivity (Δ*G* ^‡^_exp_) as determined by ^1^H NMR kinetic studies. Of these parameters, electron affinity represents a robust single-parameter predictive model of CRG reactivity with thiols, as demonstrated by test sets of additional *N*-heteroaryl lactams (MUE = 0.4 kcal/mol) and other α,β-unsaturated amide CRGs (MUE = 0.3 kcal/mol). These *N*-heteroaryl lactams were subsequently shown to inhibit cysteine protease activity (i.e., papain enzyme) to varying degrees that correlate with both the experimentally observed and predicted reactivity with GSH.

## Graphical abstract

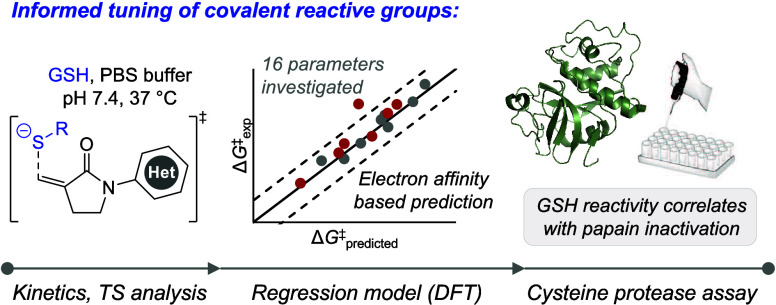

## Introduction

Designing small organic molecules that are able to selectively form covalent bonds with a nonconserved amino acid of a protein target remains a challenge in modern drug discovery.^1^ One powerful strategy for accomplishing this goal is to incorporate a protein-reactive functional group into an otherwise reversible inhibitor. Key examples of this ligand-first approach include ibrutinib and osimertinib (*vide infra*), which both include an acrylamide group that forms a covalent bond with a cysteine residue within the ATP-binding site (e.g., Cys481 of Bruton’s tyrosine kinase and Cys797 of the T790M mutant of epidermal growth factor receptor (EGFR), respectively).^2,3^ Another emerging design strategy involves identifying a covalent fragment hit and then developing additional complexity to produce a high-affinity lead compound. Sotorasib (AMG 510), used for the treatment of nonsmall cell lung cancer, was developed upon structural optimization of an electrophilic lead compound, leading to a highly potent and selective covalent inhibitor of KRAS^G12C^ (Figure 1A).^4,5^

**Figure 1:**
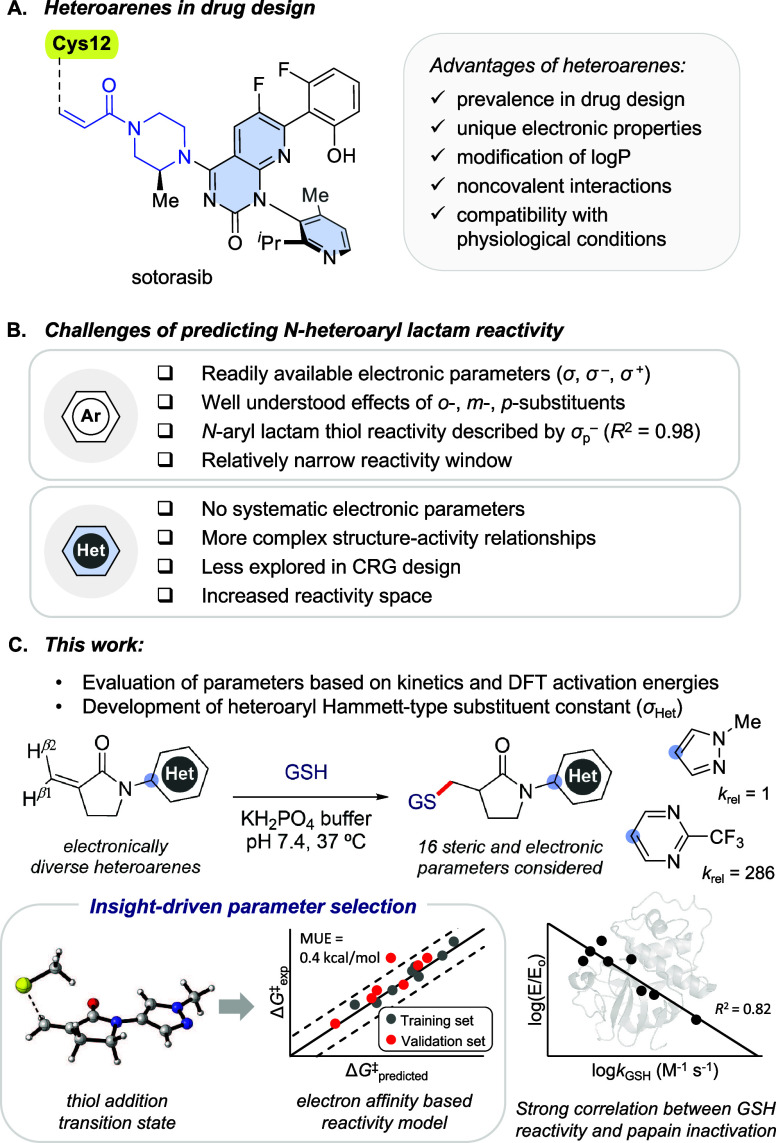
(A) FDA-approved covalent inhibitor sotorasib, which possesses an acrylamide CRG (in blue) and heteroarene moieties (filled in blue). (B) Predicting *N*-aryl versus *N*-heteroaryl lactam reactivity.^8^ (C) This work: after screening 16 possible parameters, reactivity of *N*-heteroaryl α-methylene−γ-lactams and other α,β*-*unsaturated amides with thiols can be predicted using electron affinity (EA).

Driving these drug design strategies is the availability of electrophilic covalent reactive groups (CRGs),^6^ most commonly acrylamides. However, acrylamides are not universally applicable, as multiple factors are important in selecting an optimal CRG, including protein target, amino acid residue target, metabolic stability, toxicity, size, and reactivity.^6^ Moreover, while it is critical that the reactivity of CRGs with target amino acid residues in biological systems can be predicted *a priori*, this remains difficult.^6^ To address these challenges, new chemotypes for inclusion as CRGs in drug design must be identified. Ideally, these new chemotypes should be selective and possess tunable and predictable electrophilic reactivity for use in the rational design of covalent drugs.

Inspired by nature’s omnipresent CRG—the α-methylene−γ-lactone—we have previously demonstrated that exchanging the ring oxygen for a nitrogen allows for electrophilic tuning via *N*-aryl functionalization.^7^ By reacting these *N*-aryl functionalized α-methylene−γ-lactams with glutathione (GSH), we established that diversifying the *N*-aryl group resulted in CRGs with half-lives ranging from minutes to days.^8^ Moreover, we established that Hammett substituent constants for *N*-aryl groups were highly predictive of reactivity with thiols, exhibiting a linear correlation with both ${\sigma_{p}}^{-}$ and σ_m_ (*R* ^2^ = 0.98 and 0.81, respectively) (Figure 1B).^8^

Heteroarenes are already present in many biologically active compounds.^9–11^ Given their ability to facilitate noncovalent interactions (NCIs) with target proteins,^12^ compatibility with the physiological environment,^13^ and variable water solubility and lipophilicity (logP),^11,14^ heteroarenes provide critical versatility within drug discovery programs.^9–11^ Therefore, it is surprising that few studies have considered heteroarenes as a way to systematically tune CRG electrophiles.^15^ Flanagan et al. measured the rate of GSH addition to a variety of CRGs, but only one heteroaryl-substituted CRG [*N*-(pyridine-2-yl)­acrylamide] was studied.^16^ Similarly, in Ward and co-workers’ extensive study of 46 CRGs (acrylamides, vinyl sulfonamides, and propiolamides), only five heteroarene-containing CRGs were evaluated.^17^ In this case, the authors compared the half-lives and the computed adduct formation energy for each CRG in the hopes of developing a predictive model for CRG reactivity with thiols. However, while they found a good correlation for most CRGs, the heteroaryl CRGs were outliers.^17^ In addition, no correlation (*R* ^2^ = 0.009) was observed between the half-lives and the LUMO energies when all CRGs were included.^17^ Furthermore, when the authors analyzed the aryl acrylamides alone, including one *N*-heteroaryl acrylamide, a reasonable correlation was observed (*R* ^2^ = 0.71);^17^ however, when analyzing other parameters (e.g., p*K*_a_), the heteroaryl CRGs were often excluded before a correlation was found.^17^ In a related study, Taunton et al. found that the reversibility of the thiol-Michael addition of a series of heteroaryl-substituted acrylonitriles can be predicted by their computed proton affinity.^18^ Additionally, Baud and co-workers identified 2-sulfonylpyrimidines as tunable CRGs for selective protein arylation via an S_N_Ar mechanism. Their kinetic studies with GSH demonstrated that they could systematically modulate reactivity over nine orders of magnitude using 2-sulfonylpyrimidines, and swapping out heteroaryl scaffolds could also drastically impact S_N_Ar reactivity.^19^

In an extension to biological reactivity, Uehling et al. showed that alkynyl heteroarenes form a covalent bond with Cys797 of EGFR.^20^ Similarly, Weerapana et al. systematically evaluated the proteome reactivity of six halopyridines, halopyrimidines, and dichlorotriazines, showing that the latter was selective for lysine.^21^

In chemical reactivity, Baran et al. developed a model to predict the regioselective radical functionalization of several nitrogen-containing heteroarenes by defining activating and deactivating factors.^22^ To predict the relative rate and regioselectivity of nucleophilic aromatic substitution reactions, Leitch and co-workers developed a multivariate linear regression model for various electrophiles (primarily heteroarenes) using electron affinity (EA) and molecular electrostatic potentials.^23^

Despite these extensive studies, it is still unclear how heteroaryl-substituted CRGs systematically impact reactivity with thiols. This may be due, in part, to a lack of Hammett substituent constants for describing the diverse electronic properties of heteroarenes. To address this gap, we have extended our *N*-functionalized CRG platform to contain heteroaryl groups, enhancing the tunability of the α-methylene−γ-lactam CRG scaffold. We have developed descriptors for heteroarenes that are equivalent to Hammett substituent constants for aryl groups. These efforts required synthesizing a series of electronically diverse *N*-heteroaryl α-methylene−γ-lactams, determining the experimental rate of GSH addition to each CRG, and correlating their activation free energies with the 16 steric and electronic parameters that were chosen following detailed transition state analysis (Figure 1C).

## Results and Discussion

### Properties and Selection of Heteroarenes

To understand the unique electronic and physical properties of *N*-heteroaryl lactams, our selection process intentionally included heteroarenes that exhibit a range of properties important to drug design, including lipophilicity (cLogP), basicity (p*K*_a_), aromaticity (I_A_), ionization potential, hydrogen-bond potential (p*K*_BHX_), topological polar surface area (TPSA), and dipole moment (see Tables S1 and S2 in the Supporting Information for details). Qualitative descriptors were also considered, such as prevalence in FDA-approved drugs^10,11^ and the commercial availability of the heteroaryl halide required for CRG synthesis (see Tables S1 and S2). Based upon Ward’s successful correlation of LUMO energy with the reactivity of aryl acrylamides,^17^ we decided to use computed LUMO energy values as a predictor of *N*-heteroaryl lactam reactivity toward thiols. In this way, five heteroarenes (thiophenyl, pyridinyl, imidazolyl, pyrimidinyl, and pyrazolyl) with a range of LUMO energies from −0.01 to 1.02 eV were chosen as substituents on *N*-heteroaryl lactams. Trifluoromethyl substituents were included on the 2-pyridinyl and 5-pyrimidinyl rings, due to the prevalence of this group in fluorine-containing pharmaceuticals.^24^

### Insight-Driven Selection of Parameters for Predicting Reactivity

Although Hammett substituent constants are widely used to describe electronic effects of aryl groups, analogous parameters are not universally available for heteroaryl groups. We surmised that it would be prudent to carefully select and screen various physicochemical parameters that might predict the reactivity of heteroaryl-substituted CRGs. We employed the computational workflow described in Figure 2A to establish a library of parameters based on the analysis of structural and electronic properties of computed ground-state structures of eight CRGs (e.g., **1a**) as well as the corresponding thiol addition transition states (e.g., **TS1a**). The factors that affect the reactivity of **1a** and the stability of **TS1a** were used to guide rational parameter selection, new parameter development and, eventually, the development of a model for predicting thiol reactivity using experimental activation free energies (Δ*G* ^‡^_exp_) as the training set.

**Figure 2:**
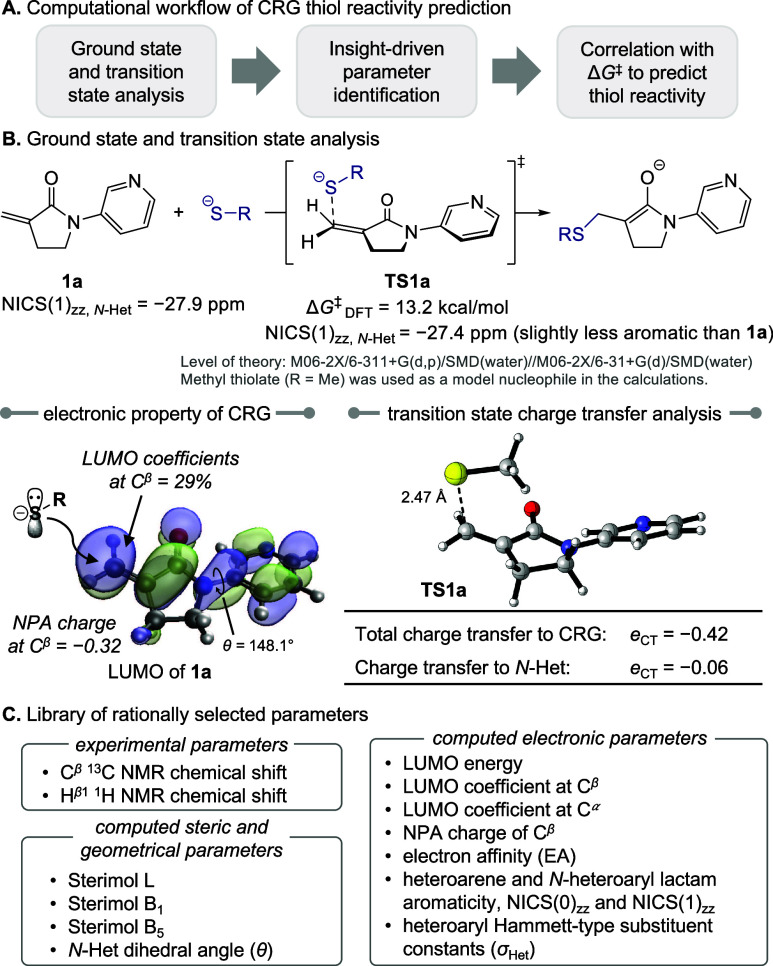
An insight-driven approach to establish a predictive thiol reactivity model.

The DFT-computed LUMO of the heteroaryl-substituted CRGs was found to be delocalized onto both the α-methylene−γ-lactam π system and the *N*-heteroaryl group, with a relatively large lobe at C^β^ of the lactam (29% in the case of **1a**, Figure 2B). This result suggests that LUMO energy, LUMO coefficients at C^β^ and C^α^, natural population analysis (NPA) charge of C^β^, and the dihedral angle about the *N*-heteroaryl bond (θ) (which affects LUMO delocalization) may all impact reactivity with thiols. The computed transition state structures indicated that both the length of the forming C–S bond and the amount of charge transfer from the thiolate nucleophile to the CRG in the transition state (*e*_CT_) are significantly affected by the identity of the *N*-heteroaryl substituent (see the Supporting Information (SI) for details). This result suggests that the ability of the *N*-heteroaryl group to stabilize the cumulating negative charge in the transition state could be an important factor for transition state stabilization. Since the EA of the CRG is easy to calculate, we believed it could be an effective, yet previously underappreciated, descriptor for predicting CRG reactivity.^23,25^ Furthermore, the charge accumulation on the heteroaryl group in **TS1a** implies that a Hammett-type substituent constant would be ideal to describe how the resonance and inductive effects of the heteroaryl group affect the transition state stability. Therefore, we sought to develop a new set of Hammett-type substituent constants for heteroaryl groups, namely, σ_Het_, that are based on DFT-computed heteroaryl carboxylic acid p*K*_a_ values (see below for details). Another consequence of the transition state charge delocalization onto the *N*-heteroaryl substituent is a slight decrease in the aromaticity of the heteroarene, as evidenced by the computed nucleus-independent chemical shift (NICS(1)_*zz*_) value of −27.9 ppm in the ground-state **1a** compared with −27.4 ppm in the transition state **TS1a** (Figure 2B). Therefore, we propose that the aromaticity of the heteroarenes, which can be described by NICS(0)_*zz*_ and NICS(1)_*zz*_, should also be evaluated as potential reactivity descriptors.

Based on these ground- and transition-state analyses, we identified 16 computed and experimental substrate electronic and steric descriptors to evaluate as potential parameters for regression models that can predict the reactivity of *N*-heteroaryl lactams (Figure 2C). While a small subset of these parameters, such as LUMO energy and NMR chemical shift, have been previously examined to study the thiol reactivity of CRGs,^8,17,26^ the remaining parameters have been largely unexplored. We expected that one or more of these parameters would correlate with experimental reaction rates that were measured from our kinetic studies.

### Synthesis of *N*-Heteroaryl α-Methylene−γ-Lactams

3-Methylene-2-pyrrolidinone was prepared in four steps from commercially available 2-pyrrolidinone, as previously described.^8^ The lactam nitrogen was functionalized using a Buchwald copper-catalyzed amidation protocol with either the heteroaryl bromide or iodide (Scheme 1A).^27,28^ In general, heteroaryl iodides afforded higher yields than heteroaryl bromides, due to the milder reaction conditions and decreased amounts of lactam decomposition. *N*-Heteroaryl lactams **1a**–**1h** were isolated in yields ranging from 22 to 86% (Scheme 1B). For lactam **1f**, ^1^H NMR indicated side reactions of 2-bromo-6-(trifluoromethyl)­pyridine.

**Scheme 1:**
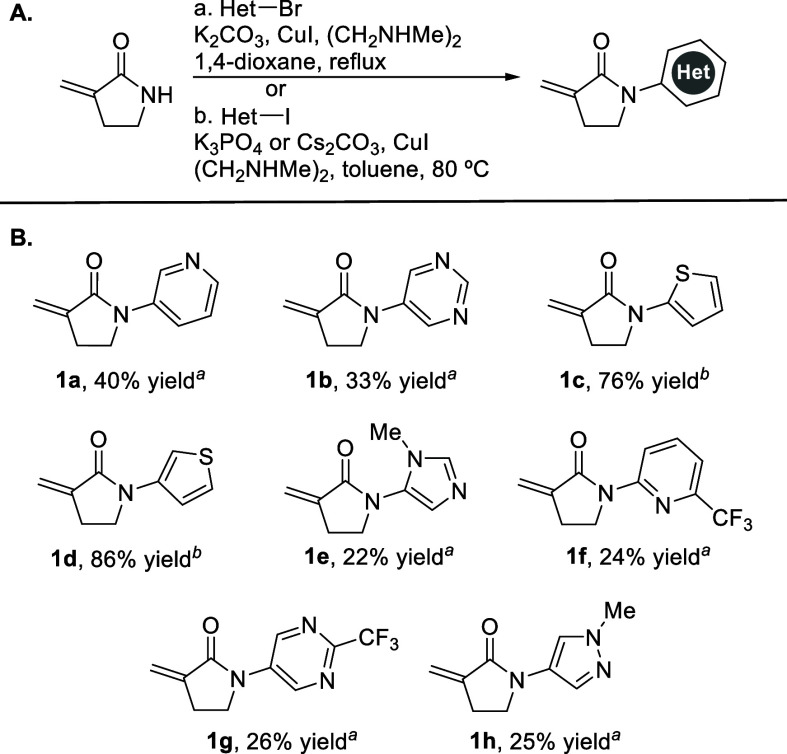
Cu­(I)-Catalyzed Amidation Reaction of Heteroaryl Halides with 3-Methylene-2-pyrrolidinone^a^

a (A) Experimental conditions for Cu-catalyzed amidation protocol using heteroaryl iodides and heteroaryl bromides. (B) Training set of eight *N*-heteroaryl lactams.

### Experimental Determination of Pseudo-First-Order Rate Constants for *N*-Heteroaryl α-Methylene−γ-Lactams **1a**–**1h**

The pseudo-first-order rate constants for the reaction of *N*-heteroaryl α-methylene−γ-lactams **1a**–**1h** with excess GSH were experimentally determined using slight modifications to the continuous *in situ* monitoring NMR method reported by Flanagan et al.^16,29^ Lactams **1a**–**1h** (1 mM) were reacted with GSH (10 mM) in 100 mM phosphate buffer solution (PBS) (pH 7.4, 100% D_2_O) at 37 °C in an NMR tube (see the SI for details). This method differs from that originally reported, as the 90% H_2_O/10% D_2_O PBS described by Flanagan resulted in broadening around the residual water signal and in some cases affected the integration value of the lactam methylene protons.^16^ A solution of lactam in PBS was added to the NMR tube, followed by the addition of GSH in PBS. The NMR tube was inverted several times, followed by vortex mixing for 2 min. A ^1^H NMR spectrum was collected every 10 min for approximately 9 h to monitor reaction progress. The pseudo-first-order rate constants were determined from the slope of the best-fit line after plotting the natural log of the lactam remaining against time (eq 1). The lactam remaining was determined by comparing the integration values for the lactam α-methylenyl protons. While only starting material peaks were utilized to determine reaction rates, evidence of the thiol addition product was also observed by ^1^H NMR (see Figure S1). Reaction half-lives were calculated from the rate constant (eq 2), and rate constants were converted to activation free energy (Δ*G* ^‡^_exp_) at 310.15 K (eq 3).

$$\ln({\left[analyte\right]}_{t})=-k_{{pseudo}_{1\mathrm{st}}}t+\ln({\left[analyte\right]}_{o})$$ 1

$$t_{1/2}=\frac{\ln\;2}{k_{{pseudo}_{1\mathrm{st}}}}$$ 2

$$k=\frac{k_{B}T}{h}e^{-\Delta {G^{‡}}_{exp}/RT}$$ 3

Pseudo-first-order rate constants were determined from triplicate runs performed on different days for each lactam. Benchmarking experiments were performed with *N*-phenylacrylamide and *N*-phenyl α-methylene−γ-lactam **S1** to compare the half-lives obtained using NMR in this study to those measured previously via LC-MS (see Figures S2 and S3 for details).^8,16^ The mean half-life for each lactam is reported in Table 1. 5-Pyrimidinyl **1b** (entry 4), 2-(6-CF_3_)-pyridinyl **1f** (entry 8), and 5-(2-CF_3_)-pyrimidinyl **1g** (entry 9) reacted quickly with GSH (*t*_1/2_ = 80, 25, and 22 min, respectively), while 3-pyridinyl **1a** (entry 3), 2-thiophenyl **1c** (entry 5), and 5-(1-methyl-1*H*)-imidazolyl **1e** (entry 7) showed only a moderate rate of reaction (*t*_1/2_ = 452, 651, and 264 min, respectively). By comparison, 3-thiophenyl **1d** (entry 6) and 4-(1-methyl-1*H*)-pyrazolyl **1h** (entry 10) reacted slowly with GSH (*t*_1/2_ = 1733 and 6188 min). *N*-Heteroaryl lactams showed a much greater range of tunability than *N*-aryl lactams, exhibiting a 286-fold rate increase from **1h** to **1g**, with a number of examples in between, whereas the previously studied *meta-* and *para*-substituted *N*-aryl lactams exhibited only a 40-fold rate increase.^8^

**Table 1:**
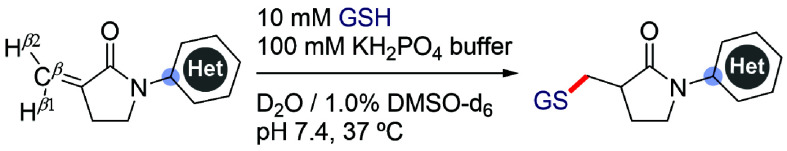

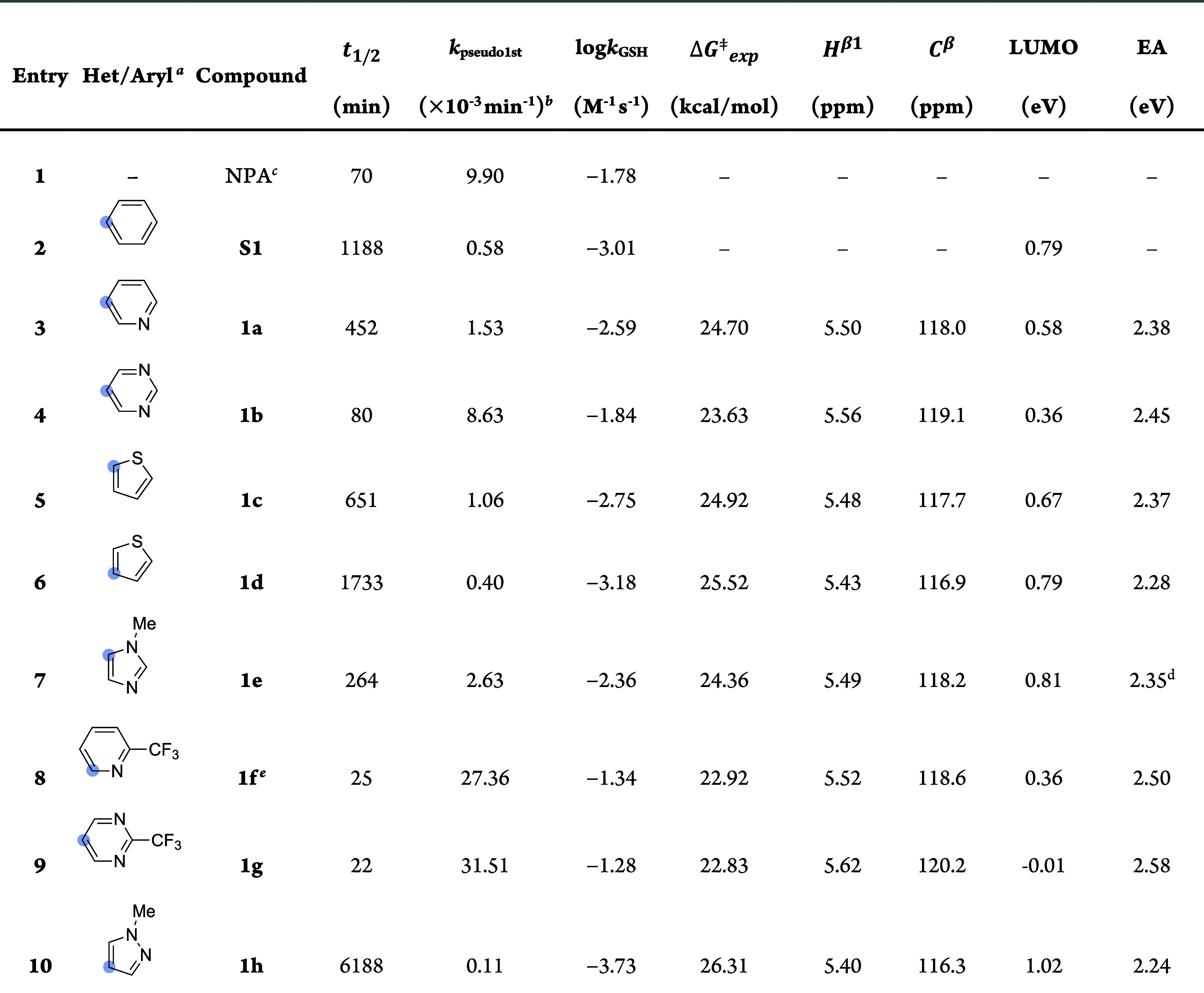
Measured Rates of GSH Addition, Half-Lives, NMR Shifts, and Computed LUMO Energies and Electron Affinity

a Blue dot indicates the location of substitution on the lactam CRG.

b Calculated from the average half-life (*t*_1/2_).

c NPA, *N*-phenylacrylamide.

d EA for unprotonated species.

e **1f** was dissolved in 100% DMSO-d_6_.

We have previously shown that the electronics of the *N*-aryl substituent impacts the measured NMR chemical shifts of the α-methylene−γ-lactam substrates in a way that is highly correlated with the thiol-Michael addition reaction rate.^8^ For example, the reaction rate of GSH addition to *N*-aryl-substituted α-methylene−γ-lactams correlated well with both the ^13^C NMR shifts of *C* ^β^ (*R* ^2^ = 0.92) and the ^1^H NMR shifts of H^β1^ (*R* ^2^ = 0.85).^8^ Similarly, for *N*-heteroaryl lactams **1a**–**1h**, good correlation was observed between the measured reaction rate of GSH addition and both the *C* ^β^ chemical shift (*R* ^2^ = 0.88 (Figure 3A)) and the H^β1^ chemical shift (*R* ^2^ = 0.83 (Figure 3B)). Although these correlations are slightly weaker than those observed for *N*-aryl substituents, this is likely due to substrate **1f** reacting more quickly than predicted, given the need to dissolve **1f** in 100% DMSO-d_6_ to overcome this substrate’s lack of solubility under our standard reaction conditions (see Figure S9 for details). When lactam **1f** is removed from the ^13^C and ^1^H NMR chemical shift plots, the correlation improves, showing *R* ^2^ values of 0.99 and 0.97, respectively (not shown).

**Figure 3:**
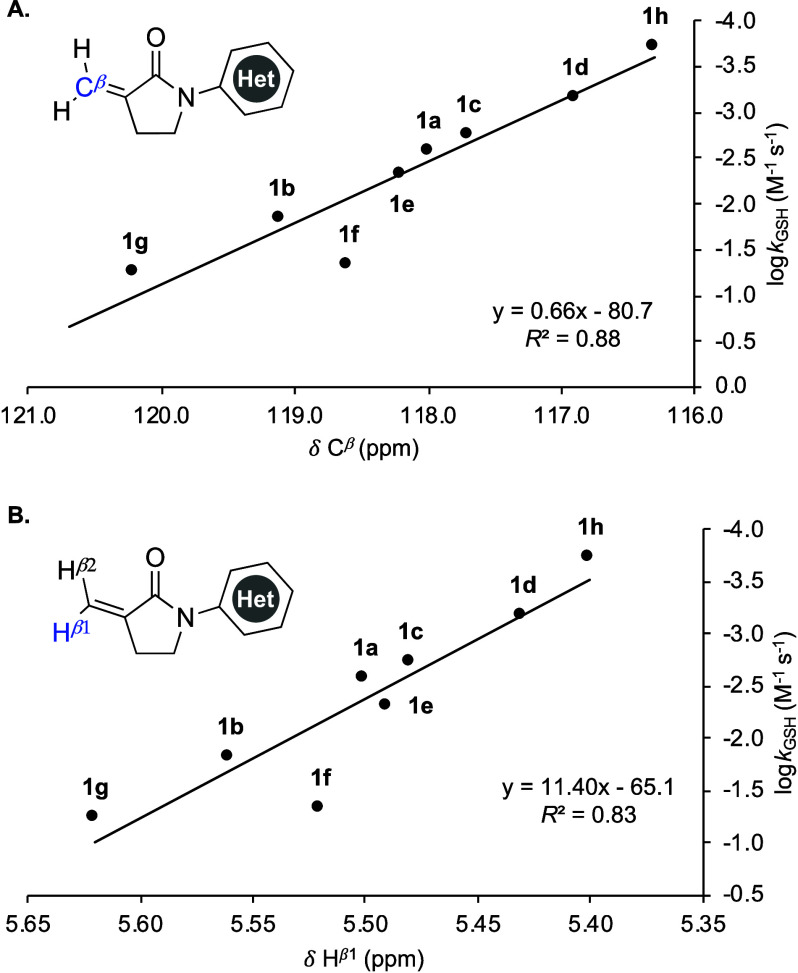
Rate of GSH addition (log*k*_GSH_) correlated with (A) ^13^C NMR chemical shift of *C* ^β^ and (B) ^1^H NMR chemical shift of H^β1^.

### Computational Analysis of the Reactivity of Lactams **1a**–**1h**

A strong correlation between the computed activation free energy (Δ*G* ^‡^_DFT_) and the experimentally determined activation free energy (Δ*G* ^‡^_exp_, eq 3) was obtained (*R* ^2^ = 0.93, Figure 4), suggesting that addition of the thiolate anion to the CRG is the rate-determining step. Consistent with our previous work on *N-*aryl lactams, the computed Δ*G* ^‡^_DFT_ was lower than the experimental Δ*G* ^‡^_exp_,^8^ since GSH exists in its protonated form under experimental conditions, rather than as the methyl thiolate anion used in calculations. On the basis of the reaction pH and the p*K*_a_ of GSH, the deprotonation is expected to be endergonic by 3.1 kcal/mol.

**Figure 4:**
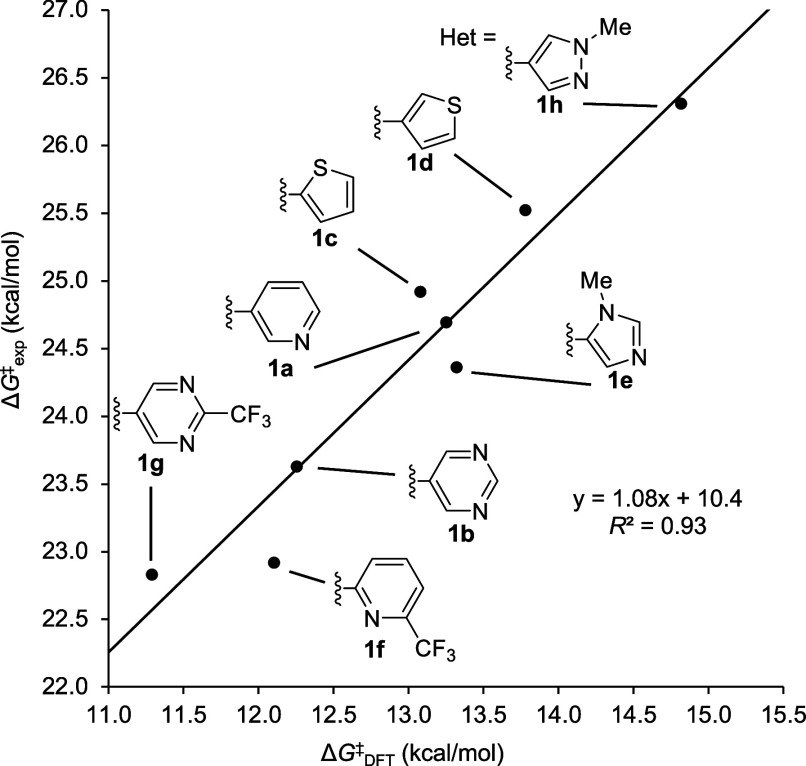
Correlation between experimentally derived activation free energies (Δ*G* ^‡^_exp_) and DFT-calculated activation free energies (Δ*G* ^‡^_DFT_).

Our lab has also reported on the relationship between Hammett substituent constants ( ${\sigma_{p}}^{-}$ and σ_m_) and the rate of GSH addition to *N*-aryl lactams.^8^ Although the distinct electronic properties of heteroarenes have been well documented,^10^ there is no commonly accepted quantitative metric equivalent to Hammett substituent constants to systematically describe their electronic properties.^30^ Using computed aqueous p*K*_a_ values of the heteroaryl carboxylic acids, we sought to describe the electronic effects of heteroaryl substituents with a Hammett-type substituent constant (σ_Het_), which can be calculated from the difference between the p*K*_a_ values of the heteroaryl carboxylic acid, p*K*_a_(Het), and benzoic acid, p*K*_a_(Ph), as a reference (Figure 5A).^31^ Because experimental p*K*_a_ values for many heteroaryl carboxylic acids are not available, we used DFT-calculated p*K*_a_ values to compute the σ_Het_ parameters (Figure 5B. See the SI for computational details of the p*K*_a_ calculations). Similar to Hammett substituent constants for substituted aryl groups, a negative σ_Het_ value indicates a heteroarene that is more electron-donating than phenyl, whereas a positive σ_Het_ indicates a more electron-withdrawing heteroarene. These computed σ_Het_ substituent constants showed good correlation with both calculated and experimental thiol reactivities (*R* ^2^ = 0.94 and 0.81 with Δ*G* ^‡^_DFT_ and Δ*G* ^‡^_exp_, respectively. See Figure 6 and Figure S40 in the SI).

**Figure 5:**
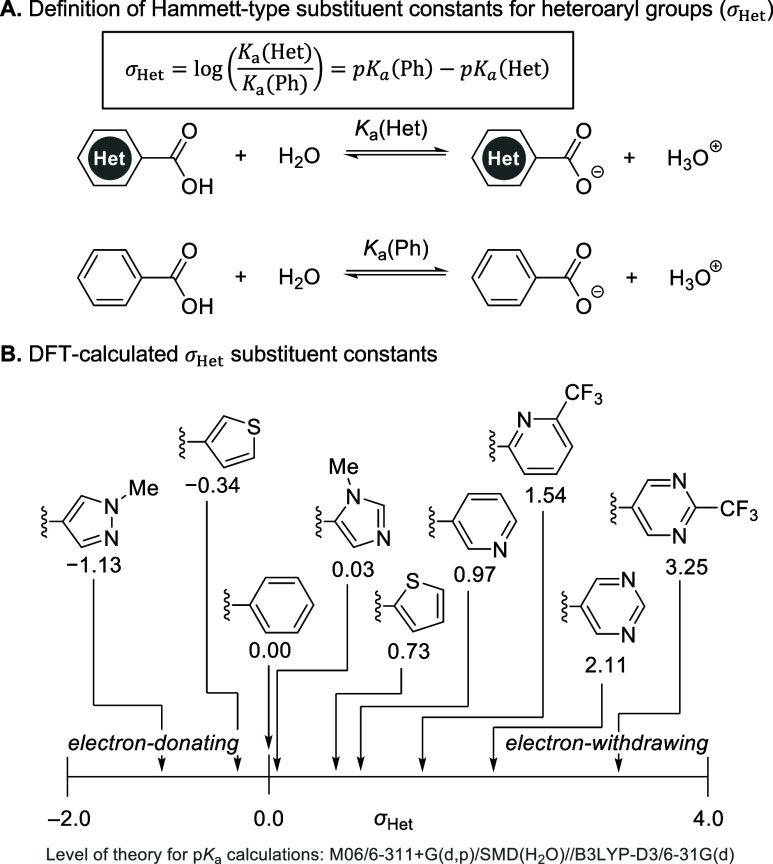
DFT-calculated Hammett-type substituent constants for heteroaryl substituents (σ_Het_).

**Figure 6:**
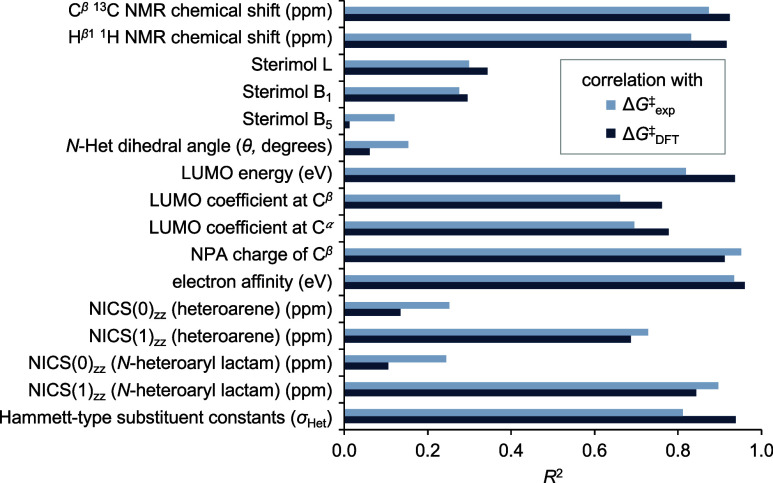
Correlation of Δ*G* ^‡^_exp_ and Δ*G* ^‡^_DFT_ (kcal/mol) with investigated parameters.

We next investigated the correlation of Δ*G* ^‡^_DFT_ and Δ*G* ^‡^_exp_ with each of the 16 descriptors we had previously identified through our insight-driven parameter selection approach (Figure 6). Many of the descriptors that show good correlation with the activation free energy are related to the electronic properties of C^β^ (e.g., NMR chemical shift, LUMO coefficient at C^β^, and NPA charge of C^β^). The best correlation with the computed activation barriers was obtained using the computed EA, which also correlated very well with the experimental activation barriers [*R* ^2^ = 0.96 and 0.93, respectively (Figure 7 and Figure S37)]. This result suggests that the thermodynamics of the rate-determining step govern the relative reactivities of Michael acceptors through the Bell–Evans–Polanyi principle.^32^ NPA charge of C^β^ also showed a very good correlation with computed and experimental activation barriers (*R* ^2^ = 0.91 and *R* ^2^ = 0.95, respectively, Figure 6 and Figure S53). Nonetheless, the NPA charge of C^β^ is difficult to generalize, as the partial charge for carbon connected to different numbers of hydrogen atoms cannot be directly compared (see Figure S61). Given this, we derived a single-parameter predictive model using the line of best fit from the correlation between Δ*G* ^‡^_exp_ and EA for the training set **1a**–**1h** (Figure S37) (see the SI for additional discussion of correlation analysis and evaluation of multivariate linear regression models S26–S31).

**Figure 7:**
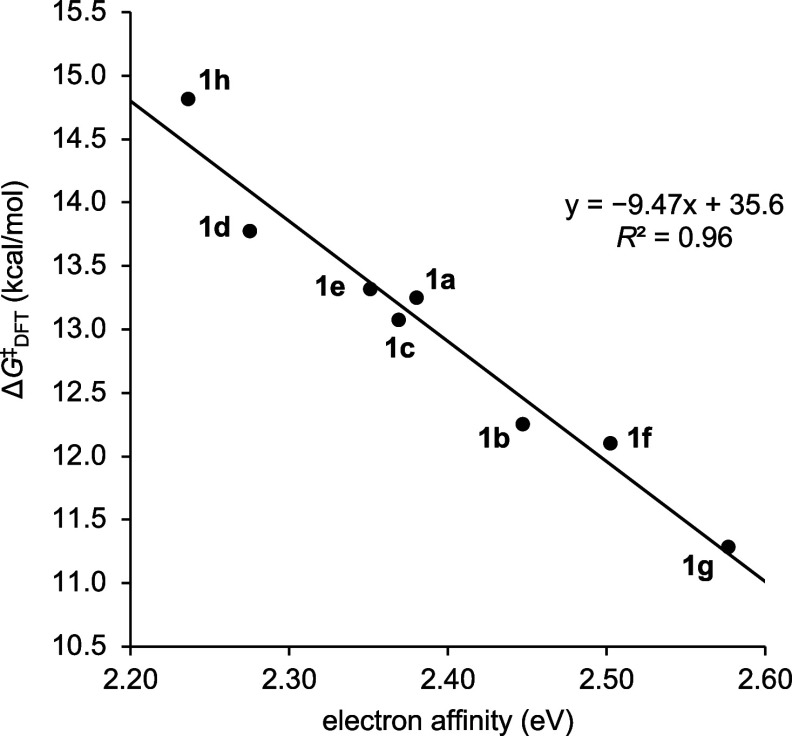
Correlation of Δ*G* ^‡^_DFT_ (kcal/mol) with electron affinity (EA, eV) of *N*-heteroaryl α-methylene−γ-lactams **1a**–**1h**.

### Validation of the Single-Parameter Predictive Model

With EA established as a promising single-parameter model for the *N*-heteroaryl lactam training set, we next set out to establish a test set of additional *N*-heteroaryl lactams to test our model (Figure 8A). Heteroaryl groups for this test set were chosen by evaluating common heteroaryl motifs found in FDA-approved drugs, while striving to increase the diversity of the data set in both molecular structure and EA values (see Figure S56 and Table S12 for heteroaryl groups considered along with calculated EA values).^10,11^ *N*-Heteroaryl α-methylene−γ-lactams **2a**–**2i** were synthesized via similar conditions to those shown in Scheme 1 in 5 to 89% yield.^27,28,33^

**Figure 8:**
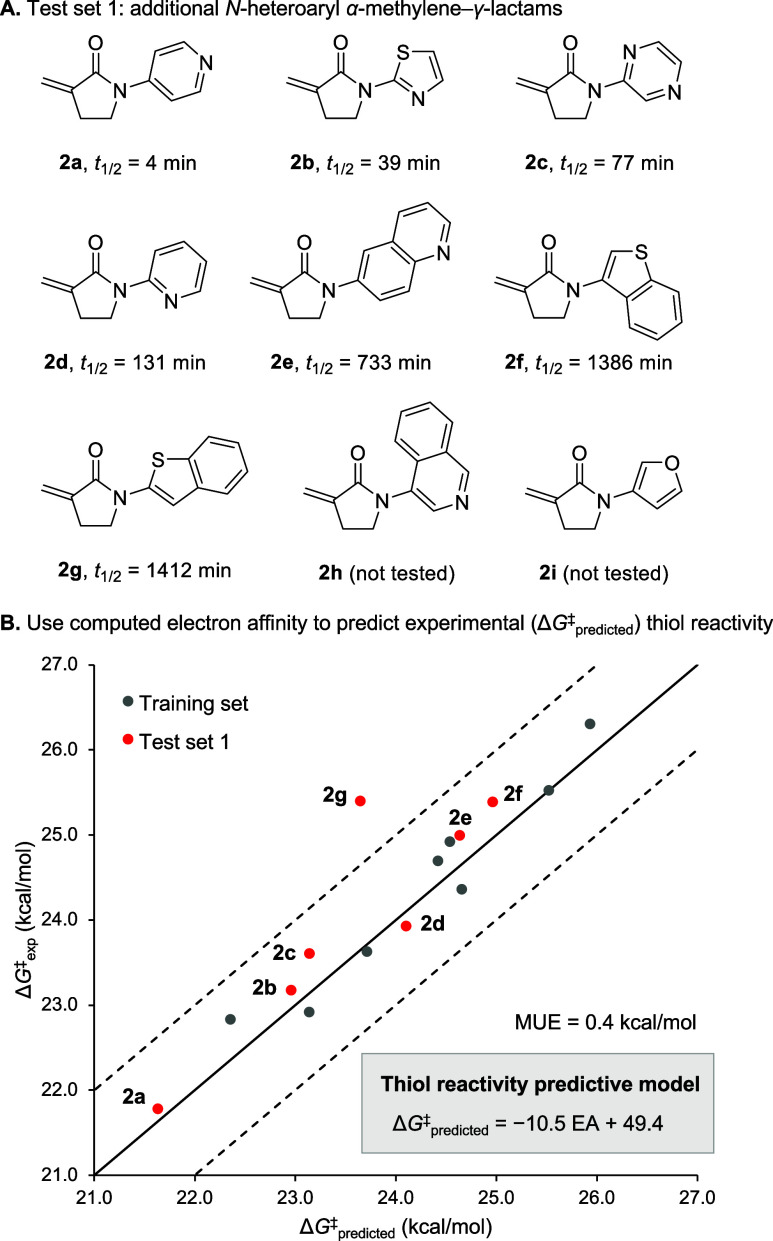
(A) Test set of *N*-heteroaryl α-methylene−γ-lactams with average half-lives determined by ^1^H NMR. (B) Validation of a single-parameter thiol reactivity predictive model (inset), which was derived from Δ*G* ^‡^_exp_ of **1a**–**1h**. EA for **1e** and **2a** was calculated using both the unprotonated basic sp^2^ ring nitrogen and the protonated species with one explicit water molecule. A weighted Δ*G* ^‡^_predicted_ was obtained from these two values for **1e** and **2a** (see the SI for details).

We then conducted duplicate or triplicate ^1^H NMR kinetic studies under pseudo-first-order conditions (*vide supra*, see Table S15). The single-parameter model using EA revealed a good correlation between Δ*G* ^‡^_exp_ and Δ*G* ^‡^_predicted_ for the test set **2a**–**2g** with a mean unsigned error (MUE) of 0.4 kcal/mol (Figure 8B). The basicity of the 4-pyridinyl nitrogen atom indicates an appreciable equilibrium between the unprotonated pyridinyl nitrogen in **2a** and the pyridinium ion at pH 7.4, which could both react with the thiol at different rates. Similarly, the basicity of the sp^2^ ring nitrogen in **1e** indicates an appreciable equilibrium under experimental conditions. Therefore, weighted averages of Δ*G* ^‡^_predicted_ from both protonated and unprotonated forms of **1e** and **2a** were used (see the SI for calculation details) and resulted in a great agreement with Δ*G* ^‡^_exp_. It should be noted that the *t*_1/2_ data for compound **2g** (dissolved in 100% DMSO-d_6_) has a great deviation, likely due to its lack of solubility in even small amounts of PBS (i.e., the small amount used to add GSH to the reaction). Finally, we did not attempt to conduct kinetic studies with lactams **2h** or **2i**, due to loss of material during purification and degradation within several weeks, respectively.

Seeking to investigate the generalizability of these single-parameter predictive models and their applicability to covalent drug design, we identified several α,β-unsaturated amide CRGs (**3a**–**3d**), which have been used in FDA-approved drugs (osimertinib, afatinib, neratinib, ibrutinib, and sotorasib) (Figure 9A). We then calculated Δ*G* ^‡^_exp_ for each, using experimental half-life data from the literature (Figure 9B).^16,17,34^ For the piperazine-based acrylamide CRG of sotorasib, we calculated the EA of a truncated piperazine fragment and used the available GSH *t*_1/2_ data for sotorasib from the literature.^5^ It was also necessary to consider the protonation state of compound **3c** when performing the EA calculations.^25,34^ Here, we found that a weighted Δ*G* ^‡^_predicted_ representing the equilibrium between the protonated and unprotonated amine resulted in the best agreement between Δ*G* ^‡^_exp_ and Δ*G* ^‡^_predicted_ (MUE = 0.3 kcal/mol, Figure 9C) (*vide supra*; see the SI for calculation details). These test calculations demonstrate that EA is an excellent predictor of CRG reactivity with thiols, allowing for direct comparison between α,β-unsaturated amide CRGs containing diverse substitution at the amide nitrogen and alkene.

**Figure 9:**
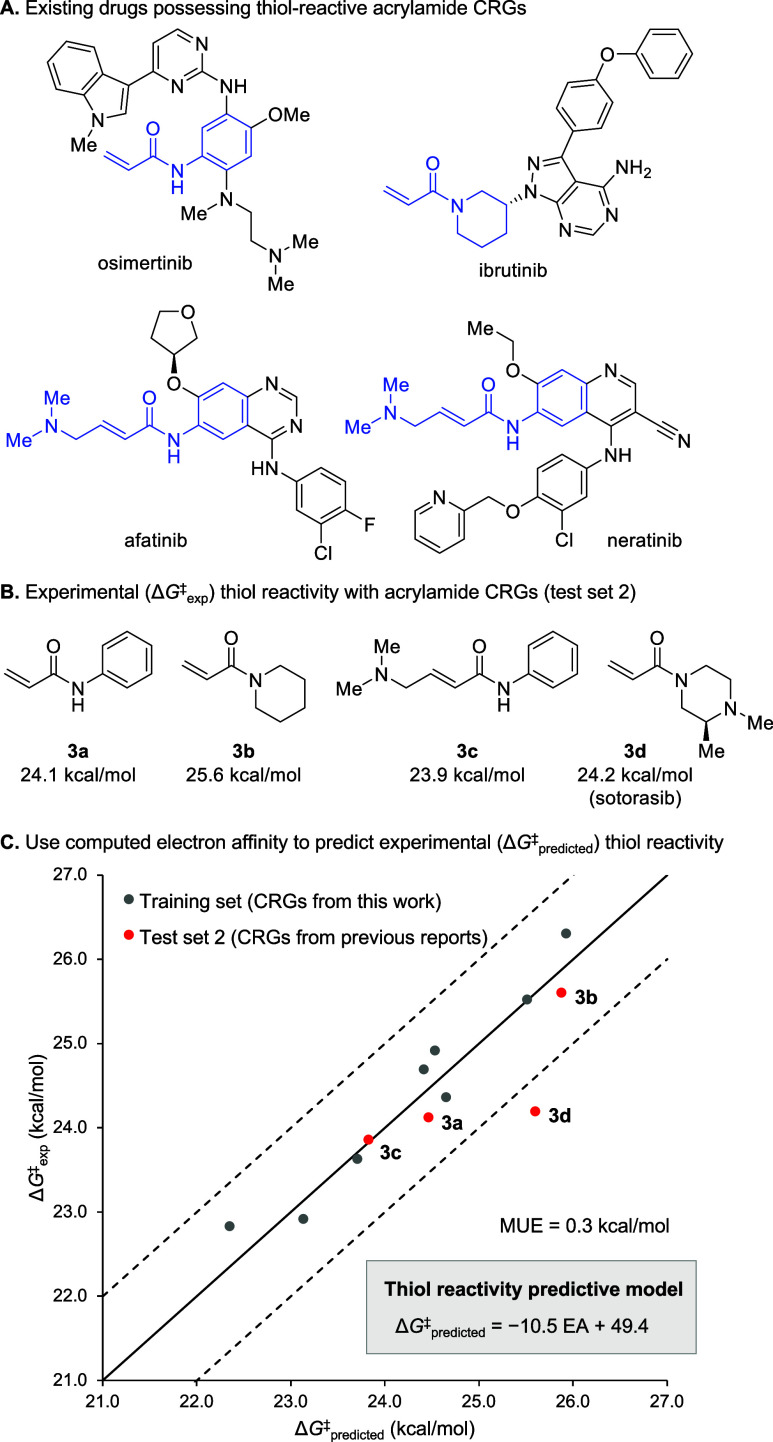
(A) Existing drugs possessing thiol-reactive acrylamide CRGs. (B) Δ*G* ^‡^_exp_ values (310.15 K) were calculated for acrylamide CRGs from experimental *t*_1/2_ for reaction with GSH at 37 °C and pH 7.4 that were obtained previously.^5,16,34^ Experimental *t*_1/2_ data for sotorasib was used for **3d**. (C) Validation of the single-parameter (EA) predictive model of thiol reactivity using acrylamide CRGs. EA for **1e** and **3c** was calculated using both the unprotonated basic nitrogen and the protonated species with one explicit water molecule. A weighted Δ*G* ^‡^_predicted_ was obtained from these two values for **1e** and **3c** (see the SI for details).

### Extension of Tunable Thiol Reactivity to Papain Inactivation

Having established the ability to tune the reactivity of *N*-heteroaryl α-methylene−γ-lactams toward GSH and predict the relative rates of reactivity using a single-parameter EA model, we next sought to determine whether the observed structure–reactivity relationships would translate to a biological context. To this end, we evaluated a subset of the *N*-(hetero)­aryl lactams as inhibitors of papain, a protease from the papaya fruit that depends on a nucleophilic cysteine for its catalytic function. Papain is the prototypical member of the papain-like protease (PLP) family, the largest subgroup of cysteine proteases.^35^ A renewed interest in PLP enzymes has emerged recently, due to the potential of the PLP family member SARS-CoV-2 PL^pro^ as an antiviral drug target.^36^

Eight compounds (**S1**, **1a**, **1c**, **1d**, and **2a**–**2d**), possessing a range of reactivities toward GSH, were selected and assayed for their ability to inhibit papain (200 μM). Each *N*-(hetero)­aryl lactam (2 mM) was incubated with papain in parallel for 22 h at room temperature. Enzymatic activity was then quantified by dilution with a chromogenic substrate (Figure S63). The activity values for papain treated with each inhibitor (*E*) were normalized to the activity of a control, which was incubated under identical conditions but without any added CRG (*E*_o_). The *N*-substituted lactams tested showed varying degrees of papain inactivation, with fraction residual activity (*E*/*E*_o_) values ranging from 7 to 62% (Figure S64). Based on the simple structure of the lactams and high nucleophilicity of the papain active site cysteine,^37^ covalent modification of the active site is the most plausible mechanism for inhibition; however, covalent attachments elsewhere on the protein cannot be ruled out.

Given the high concentrations and long incubation time employed in the assay, the inhibitory potencies of the *N*-heteroaryl lactams are modest. Still, they are consistent with prior work in which papain could be inactivated by Michael acceptors that lacked any functionality to target the active site.^38^ More important, however, are the differences between compounds within the compound set, where a strong correlation between the extent of enzyme inactivation and the rate constant measured for reaction with GSH is observed (Figure 10A). This result suggests that reactions carried out with a model thiol nucleophile (i.e., GSH) are relevant to the thiol in the papain active site and may be more generally applicable. We also observed a correlation between the extent of enzyme inactivation and Δ*G* ^‡^_predicted_ from the single-parameter EA model (Figure 10B). This result suggests that the methods outlined above have utility in the context of enzyme inhibitor development.

**Figure 10:**
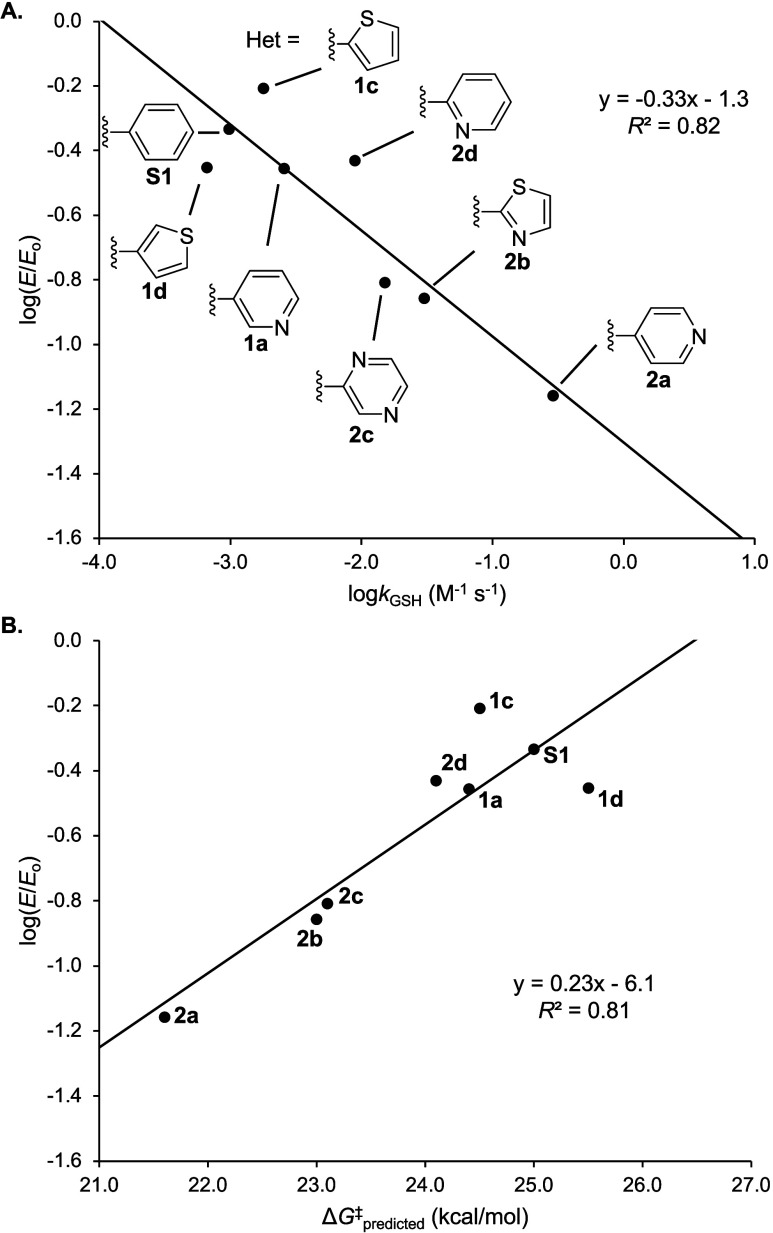
Correlation of papain inactivation [log­(*E*/*E*_o_)] with (A) rate of reaction with GSH (log*k*_GSH_) and (B) computationally predicted activation free energies from the single-parameter EA model (Δ*G* ^‡^_predicted_). A weighted Δ*G* ^‡^_predicted_ value was used for **2a** (*vide supra*; see the SI for details).

## Conclusions

We have developed a single-parameter predictive model to describe the thiol reactivity of *N*-functionalized α-methylene−γ-lactam CRGs bearing a range of electronically diverse heteroaryl groups. ^1^H NMR kinetic studies demonstrate that tuning the electrophilicity of the CRG, and thus its overall reactivity with thiols, is possible by varying the *N*-heteroaryl group. DFT analysis of thiol-Michael transition states revealed a substantial charge transfer from the thiol nucleophile to the CRG, which is partially delocalized into the heteroarene. We then investigated 16 experimental and computed steric and electronic properties of the *N*-heteroaryl lactam CRGs to determine which, if any, are able to describe the nature of the transition state and the ability of the heteroaryl group to stabilize the cumulating charge. In this way, EA was identified as a useful single-parameter model for predicting the reactivity of *N*-heteroaryl lactams with GSH. Using a test set of additional *N*-heteroaryl lactams, we confirmed that EA, an underutilized parameter for reactivity prediction, provides a robust thiol reactivity model (MUE = 0.4 kcal/mol), applicable to a test set of α,β-unsaturated amide CRG fragments that appear in FDA-approved drugs (MUE = 0.3 kcal/mol). We have also developed a novel Hammett-type substituent constant, derived from the p*K*_a_ of the corresponding heteroaryl carboxylic acids, to describe the effect of the electronic properties of heteroaryl groups on thiol addition. Furthermore, we have confirmed that both experimental and predicted reactivity with GSH translates to inactivation of the papain enzyme. We expect that our new Hammett-type substituent constant for heteroaryl groups and single-parameter predictive model will be valuable tools for studying the electronic properties of heteroarenes, allowing for the development of new α,β-unsaturated amide CRGs that can be rationally designed and tuned for optimal reactivity with protein thiols.

## Experimental Procedures

### General Methods

Unless otherwise stated, all reactions were performed in flame-dried glassware under an atmosphere of argon. All commercially available starting materials were used as received, without further purification. Toluene was freshly distilled from CaH_2_ prior to use. 1,4-Dioxane was degassed by bubbling nitrogen through the solvent for 30 min. Column chromatography was performed using 40–63 μm, 60 Å pore size silica gel, grade P60. TLC was performed on SiliCycle glass backed 60 Å plates containing an F_254_ indicator. ^1^H NMR and ^13^C NMR spectra were obtained using a Bruker Avance 400 or 500 MHz spectrometer. Spectra were referenced to chloroform (^1^H: 7.26 ppm and ^13^C: 77.16 ppm). Chemical shifts are reported in ppm and multiplicities are indicated by singlet (s), doublet (d), triplet (t), quartet (q), doublet of doublets (dd), doublet of triplets (dt), triplet of doublets (td), and multiplet (m). Coupling constants are reported in hertz. NMR spectra for compound characterization were obtained at room temperature. Reaction kinetics were performed by NMR at 37 °C using a Bruker Avance 600 MHz spectrometer. All high-resolution mass spectrometry data were obtained on a Fisher Scientific Q Exactive with an Orbitrap mass analyzer using ESI as the ionization source. IR spectra were obtained using a Nicolet Avatar E.S.P. 360 FT-IR. The purity of representative compounds tested for the papain protease inhibition assay was ≥95% as determined by HPLC (Figures S80–S87).

#### General Procedure A: *N*-Heteroarylation of 3-Methylene-2-pyrrolidinone (∼0.1 mmol Scale)

A 2 mL Biotage microwave vial equipped with a 1 cm triangular Teflon-coated stir bar was charged with base (2 equiv), copper­(I) iodide (0.15 equiv), and if a solid, the heteroaryl iodide or heteroaryl bromide (1.6 equiv) via temporary removal of the septum. The septum was replaced, and the vial was sealed with a PTFE crimp cap. The atmosphere was purged and refilled with argon (3×). If the heteroaryl iodide or heteroaryl bromide was a liquid, it was then added via syringe. *N*,*N*′-Dimethylethylenediamine (0.3 equiv) was added via syringe, followed by a solution of 3-methylene-2-pyrrolidinone in solvent (1 equiv, 0.1 M). The reaction was lowered into a preheated oil bath (80 or 110 °C) and maintained until TLC indicated consumption of SM. The vial was removed from the oil bath and allowed to cool to rt. After removing the cap, the reaction mixture was filtered through a silica gel plug (1 × 1 cm) and concentrated *in vacuo*. The crude residue was purified by silica gel flash column chromatography.

##### 3-Methylene-1-(pyridin-3-yl)­pyrrolidin-2-one (**1a**)

The synthesis of **1a** was performed according to General Procedure A using potassium carbonate (K_2_CO_3_, 30 mg, 0.22 mmol), CuI (3 mg, 0.015 mmol), 3-bromopyridine (15 μL, 0.16 mmol), *N,N′*-dimethylethylenediamine (3.3 μL, 0.03 mmol), and 3-methylene-2-pyrrolidinone (10 mg, 0.11 mmol) in 1,4-dioxane (1.1 mL) at 110 °C. The crude residue was purified by silica gel flash column chromatography (1 × 10 cm, 5 mL fractions) eluting with 10% methanol/dichloromethane to yield **1a** as a white solid (7 mg, 40%). ^1^H NMR (500 MHz, CDCl_3_): δ 8.90 (d, *J* = 2.6 Hz, 1H), 8.44–8.40 (m, 1H), 8.40–8.35 (m, 1H), 7.33 (dd, *J* = 4.9, 8.6 Hz, 1H), 6.17 (t, *J* = 2.7 Hz, 1H), 5.50 (t, *J* = 2.4 Hz, 1H), 3.90 (t, *J* = 6.9 Hz, 2H), 2.99–2.92 (m, 2H). ^13^C NMR (125 MHz, CDCl_3_) δ 167.6, 145.8, 140.4, 139.3, 136.5, 127.0, 123.6, 118.0, 44.6, 24.0. IR (thin film) 2922, 2859, 1699, 1660, 1485 cm^–1^. HRMS (ESI) *m*/*z*: [M + H]^+^ calcd for C_10_H_11_ON_2_: 175.0866, found 175.0869. TLC *R_f_* = 0.33 (100% ethyl acetate) [silica gel, KMnO_4_, UV]. mp = 80–85 °C.

##### 3-Methylene-1-(pyrimidin-5-yl)­pyrrolidin-2-one (**1b**)

The synthesis of **1b** was performed according to General Procedure A using potassium carbonate (K_2_CO_3_, 30 mg, 0.22 mmol), CuI (3 mg, 0.015 mmol), 5-bromopyrimidine (25 mg, 0.16 mmol), *N,N′*-dimethylethylenediamine (3.6 μL, 0.03 mmol), and 3-methylene-2-pyrrolidinone (10 mg, 0.11 mmol) in 1,4-dioxane (1.1 mL) at 110 °C. The crude residue was purified by silica gel flash column chromatography (1 × 10 cm, 5 mL fractions) eluting with 10% methanol/dichloromethane to yield **1b** as a white solid (6 mg, 33%). ^1^H NMR (500 MHz, CDCl_3_): δ 9.21 (br s, 2H), 9.02 (br s, 1H), 6.22 (t, *J* = 2.8 Hz, 1H), 5.56 (t, *J* = 2.4 Hz, 1H), 3.90 (t, *J* = 6.9 Hz, 2H), 3.05–2.97 (m, 2H). Water (1.56). ^13^C NMR (125 MHz, CDCl_3_) δ 167.6, 154.4, 146.9, 138.2, 135.0, 119.1, 43.6, 24.0. IR (thin film) 2927, 2856, 1728, 1686, 1657, 1490 cm^–1^. HRMS (ESI) *m*/*z*: [M + H]^+^ calcd for C_9_H_10_N_3_O: 176.0818, found 176.0821. TLC *R_f_* = 0.30 (100% ethyl acetate) [silica gel, KMnO_4_, UV]. mp = 127–133 °C.

##### 3-Methylene-1-(thiophen-2-yl)­pyrrolidin-2-one (**1c**)

The synthesis of **1c** was performed according to General Procedure A using cesium carbonate (Cs_2_CO_3_, 67 mg, 0.21 mmol), CuI (3 mg, 0.015 mmol), 2-iodothiophene (18 μL, 0.16 mmol), *N,N′*-dimethylethylenediamine (3.3 μL, 0.03 mmol), and 3-methylene-2-pyrrolidinone (10 mg, 0.11 mmol) in toluene (1.1 mL) at 80 °C. The crude residue was purified by silica gel flash column chromatography (1 × 10 cm, 5 mL fractions) eluting with 50% ethyl acetate/hexanes to yield **1c** as a white solid (14 mg, 76%). ^1^H NMR (500 MHz, CDCl_3_): δ 6.99 (dd, *J* = 1.4, 5.5 Hz, 1H), 6.94–6.90 (m, 1H), 6.63 (dd, *J* = 1.3, 3.8 Hz, 1H), 6.16 (t, *J* = 2.8 Hz, 1H), 5.48 (t, *J* = 2.4 Hz, 1H), 3.91 (t, *J* = 6.7 Hz, 2H), 3.02–2.94 (m, 2H). Water (1.56). ^13^C NMR (125 MHz, CDCl_3_) δ 165.0, 140.9, 138.4, 124.1, 119.0, 117.7, 110.9, 45.6, 24.0. IR (thin film) 2959, 2912, 1676, 1652, 1478 cm^–1^. HRMS (ESI) *m*/*z*: [M + H]^+^ calcd for C_9_H_10_ONS: 180.0478, found 180.0480. TLC *R_f_* = 0.79 (100% ethyl acetate) [silica gel, KMnO_4_, UV]. mp = 92–96 °C.

##### 3-Methylene-1-(thiophen-3-yl)­pyrrolidin-2-one (**1d**)

The synthesis of **1d** was performed according to General Procedure A using tripotassium phosphate (K_3_PO_4_, 47 mg, 0.22 mmol), copper­(I) iodide (CuI, 3 mg, 0.015 mmol), 3-iodothiophene (16 μL, 0.16 mmol), *N,N′*-dimethylethylenediamine (3.3 μL, 0.03 mmol), and 3-methylene-2-pyrrolidinone (10 mg, 0.11 mmol) in toluene (1.1 mL) at 80 °C. The crude residue was purified by silica gel flash column chromatography (1 × 10 cm, 5 mL fractions) eluting with 30% ethyl acetate/hexanes to yield **1d** as a white solid (17 mg, 86%). ^1^H NMR (500 MHz, CDCl_3_): δ 7.61 (dd, *J* = 1.4, 5.2 Hz, 1H), 7.43–7.38 (m, 1H), 7.35–7.30 (m, 1H), 6.13 (t, *J* = 2.4 Hz, 1H), 5.43 (t, *J* = 2.3 Hz, 1H), 3.86 (t, *J* = 6.6 Hz, 2H), 2.97–2.88 (m, 2H). Ethyl acetate (4.12 and 2.05) and water (1.56). ^13^C NMR (125 MHz, CDCl_3_) δ 166.0, 139.6, 138.3, 125.1, 120.4, 116.9, 109.5, 45.5, 24.0. IR (thin film) 2924, 2854, 1682, 1657, 1491 cm^–1^. HRMS (ESI) *m*/*z*: [M + H]^+^ calcd for C_9_H_10_ONS: 180.0478, found 180.0480. TLC R_f_ = 0.49 (100% ethyl acetate) [silica gel, KMnO_4_, UV]. mp = 79–85 °C.

#### General Procedure B: *N*-Heteroarylation of 3-Methylene-2-pyrrolidinone (∼0.5 mmol Scale)

A flame- or oven-dried 10–20 mL Biotage microwave vial equipped with a 1 cm Teflon-coated magnetic stir bar and a rubber septum was purged and refilled via an argon-filled balloon (3×). The vial was charged with base (2 equiv), copper­(I) iodide (0.15 equiv), and if a solid, the heteroaryl halide (1.6 equiv) via temporary removal of the septum. The septum was replaced, and the vial was sealed with a PTFE crimp cap. The atmosphere was purged and refilled via an argon-filled balloon (3×). In a separate scintillation vial, 3-methylene-2-pyrrolidinone (1 equiv) was dissolved in toluene (if using heteroaryl iodide) or degassed 1,4-dioxane (if using heteroaryl bromide) (1–2 mL), and this solution was transferred to the reaction vial via syringe. The scintillation vial was rinsed with solvent and added to the vial for a final reaction concentration of 0.1 M. *N,N′*-Dimethylethylenediamine (0.3 equiv) was added to the reaction vial via syringe, followed by the heteroaryl halide, if it was a liquid. The reaction was heated in an oil bath preheated to 80–82 °C or 109–110 °C for toluene or 114–123 °C for 1,4-dioxane. The argon balloon was removed, the cap was wrapped with parafilm, and the reaction was maintained until ^1^H NMR indicated consumption of SM or no further conversion of SM. For ^1^H NMR monitoring, an aliquot (0.1 mL) was removed via syringe and concentrated *in vacuo*. The flask was removed from the oil bath and allowed to cool to rt. The reaction mixture was filtered through a silica gel plug and concentrated *in vacuo*. The crude residue was purified by silica gel flash column chromatography.

##### 1-(1-Methyl-1*H*-imidazol-4-yl)-3-methylenepyrrolidin-2-one (**1e**)

The synthesis of **1e** was performed according to General Procedure B using potassium carbonate (K_2_CO_3_, 114 mg, 0.82 mmol), CuI (12 mg, 0.062 mmol), 5-bromo-1-methylimidazole (106 mg, 0.66 mmol), *N,N′*-dimethylethylenediamine (13 μL, 0.12 mmol), and 3-methylene-2-pyrrolidinone (40 mg, 0.41 mmol) in 1,4-dioxane (4.1 mL) at 114 °C. The silica gel plug (2 × 3.5 cm) was flushed with methanol. The crude residue was purified by silica gel flash column chromatography (2 × 12 cm, 10 mL fractions) eluting with 10% methanol/dichloromethane to yield **1e** as a brown oil (16 mg, 22%). ^1^H NMR (500 MHz, CDCl_3_): δ 7.40 (s, 1H), 6.92 (s, 1H), 6.13 (t, *J* = 2.8 Hz, 1H), 5.49 (t, *J* = 2.4 Hz, 1H), 3.72 (t, *J* = 6.8 Hz, 2H), 3.54 (s, 3H), 2.98–2.94 (m, 2H). Water (1.52), grease (1.24, 0.90–0.79). Small impurities (7.62–6.72, 6.30–5.08, 3.90–3.32, 3.12–2.18, 2.09–1.74, 1.66–1.41, 0.06). ^13^C NMR (125 MHz, CDCl_3_) δ 168.1, 138.1, 136.7, 128.7, 122.8, 118.2, 48.1, 31.7, 24.9. IR (thin film) 2954, 2923, 2869, 2851, 1693, 1660, 1607, 1574 cm^–1^. HRMS (ESI) *m*/*z*: [M + H]^+^ calcd for C_9_H_12_ON_3_ 178.0975; found 178.0980. TLC *R_f_* = 0.28 (10% methanol/dichloromethane) [silica gel, KMnO_4_, UV].

##### 3-Methylene-1-(6-(trifluoromethyl)­pyridin-2-yl)­pyrrolidin-2-one (**1f**)

The synthesis of **1f** was performed according to General Procedure B using potassium carbonate (K_2_CO_3_, 142 mg, 1.0 mmol), CuI (15 mg, 0.077 mmol), 2-bromo-6-(trifluoromethyl)­pyridine (186 mg, 0.82 mmol), *N,N′*-dimethylethylenediamine (17 μL, 0.15 mmol), and 3-methylene-2-pyrrolidinone (50 mg, 0.51 mmol) in 1,4-dioxane (5.1 mL) at 110 °C (later increased to 123 °C to achieve refluxing). The silica gel plug (2.5 × 3 cm) was flushed with ethyl acetate. The crude residue was purified by silica gel flash column chromatography (3.5 × 12 cm, 10 mL fractions) eluting with 1% methanol/dichloromethane. To remove grease identified by ^1^H NMR, the product was dissolved in ethanol and filtered through a cotton plug to yield **1f** as a yellow-white solid (30 mg, 24%). ^1^H NMR (500 MHz, CDCl_3_): δ 8.77 (d, *J* = 8.6 Hz, 1H), 7.87 (t, *J* = 8.0 Hz, 1H), 7.43 (d, *J* = 8.6 Hz, 1H), 6.21 (t, *J* = 2.8 Hz, 1 H), 5.52 (t, *J* = 2.4 Hz, 1H), 4.14 (t, *J* = 7.0 Hz, 2H), 2.88–2.92 (m, 2H). Water (1.54), grease (1.33–1.16 and 0.86–0.92). Small impurities (1.48–1.33, 1.06, 0.07). ^13^C NMR (125 MHz, CDCl_3_) δ 167.9, 152.3, 146.3 (q, *J* = 35.4 Hz), 140.6, 138.9, 121.4 (q, *J* = 274.3 Hz), 118.6, 117.5, 116.0 (q, *J* = 2.7 Hz), 43.9, 23.4. IR (thin film) 2961, 2927, 2912, 1702, 1660, 1595 cm^–1^. HRMS (ESI) *m*/*z*: [M + H]^+^ calcd for C_11_H_10_ON_2_F_3_ 243.0740; found 243.0740. TLC *R_f_* = 0.62 (1% methanol/dichloromethane) [silica gel, KMnO_4_, UV]. mp = 99–102 °C.

##### 3-Methylene-1-(thiazol-2-yl)­pyrrolidin-2-one (**2b**)

The synthesis of **2b** was performed according to General Procedure B using potassium phosphate (K_3_PO_4_, 175 mg, 0.82 mmol), CuI (12 mg, 0.062 mmol), 2-bromothiozole (59 μL, 0.66 mmol), *N*,*N*′-dimethylethylenediamine (13 μL, 0.12 mmol), and 3-methylene-2-pyrrolidinone (40 mg, 0.41 mmol) in 1,4-dioxane (4.1 mL) at 114 °C. The silica gel plug (1.5 × 2.5 cm) was flushed with ethyl acetate. The crude residue was purified by silica gel flash column chromatography (3 × 14 cm, 5 mL fractions) eluting with 50% ethyl acetate/hexanes to yield **2b** as a white solid (14 mg, 19%). ^1^H NMR (500 MHz, CDCl_3_): δ 7.51 (d, *J* = 3.5 Hz, 1H), 7.06 (d, *J* = 3.5 Hz, 1H), 6.23 (t, *J* = 2.7 Hz, 1H), 5.56 (t, *J* = 2.5 Hz, 1H), 4.17 (t, *J* = 6.8 Hz, 2H), 3.03–2.94 (m, 2H). Water (1.77) and grease (1.26). ^13^C NMR (125 MHz, CDCl_3_) δ 166.1, 158.2, 138.5, 138.0, 119.2, 114.5, 44.8, 24.1. IR (thin film) 3105, 2964, 2914, 1687, 1654, 1510 cm^–1^. HRMS (ESI) *m*/*z*: [M + H]^+^ calcd for C_8_H_9_ON_2_S: 181.0430; found 181.0427. TLC *R_f_* = 0.34 (50% ethyl acetate/hexanes) [silica gel, KMnO_4_, UV].

##### 3-Methylene-1-(pyrazin-2-yl)­pyrrolidin-2-one (**2c**)

The synthesis of **2c** was performed according to General Procedure B using potassium phosphate (K_3_PO_4_, 175 mg, 0.82 mmol), CuI (12 mg, 0.062 mmol), 2-iodopyrazine (65 μL, 0.66 mmol), *N*,*N*′-dimethylethylenediamine (13 μL, 0.12 mmol), and 3-methylene-2-pyrrolidinone (40 mg, 0.41 mmol) in toluene (4.1 mL) at 80 °C. The silica gel plug (2 × 2 cm) was flushed with ethyl acetate. The crude residue was purified by silica gel flash column chromatography (2 × 12 cm, 5 mL fractions) eluting with 50% ethyl acetate/hexanes to yield **2c** as a white solid (28 mg, 39%). ^1^H NMR (500 MHz, CDCl_3_): δ 9.87 (s, 1H), 8.34 (s, 2H), 6.23 (t, *J* = 2.8 Hz, 1H), 5.54 (t, *J* = 2.4 Hz, 1H), 4.05 (t, *J* = 7.0 Hz, 2H), 2.97–2.91 (m, 2H). Water (1.57 ppm). ^13^C NMR (125 MHz, CDCl_3_) δ 167.5, 149.0, 142.0, 139.9, 139.7, 138.0, 118.9, 43.2, 23.8. HRMS (ESI) *m*/*z*: [M + H]^+^ calcd for C_9_H_10_ON_3_: 176.0818; found 176.0823. TLC *R_f_* = 0.31 (50% ethyl acetate/hexanes) [silica gel, KMnO_4_, UV]. mp = 96–98 °C.

##### 3-Methylene-1-(pyridin-2-yl)­pyrrolidin-2-one (**2d**)

The synthesis of **2d** was performed according to General Procedure B using potassium phosphate (K_3_PO_4,_ 175 mg, 0.82 mmol), CuI (12 mg, 0.062 mmol), 2-iodopyridine (70 μL, 0.66 mmol), *N*,*N*′-dimethylethylenediamine (13 μL, 0.12 mmol), and 3-methylene-2-pyrrolidinone (40 mg, 0.41 mmol) in toluene (4.1 mL) at 81–82 °C. The silica gel plug (2 × 3 cm) was flushed with methanol. The crude residue was purified by silica gel flash column chromatography (2 × 12 cm, 5 mL fractions) eluting with 20% ethyl acetate/hexanes to yield **2d** as a white solid (64 mg, 89%). ^1^H NMR (500 MHz, CDCl_3_): δ 8.56 (d, *J* = 8.5 Hz, 1H), 8.39 (br, 1H), 7.75–7.70 (m, 1H), 7.06 (t, *J* = 5.3 Hz, 1H), 6.17 (t, *J* = 2.8 Hz, 1H), 5.48 (t, *J* = 2.4 Hz, 1H), 4.11 (t, *J* = 6.9 Hz, 2H), 2.91–2.86 (m, 2H). Water (1.54). ^13^C NMR (125 MHz, CDCl_3_) δ 167.6, 152.2, 147.7, 141.0, 137.8, 119.9, 117.7, 115.1, 44.0, 23.6. IR (thin film) 3514, 3061, 2958, 2913, 1699, 1659, 1607, 1587, 1572 cm^–1^. HRMS (ESI) *m*/*z*: [M + H]^+^ calcd for C_10_H_11_ON_2_: 175.0866; found 175.0871. TLC *R_f_* = 0.18 (20% ethyl acetate/hexanes) [silica gel, KMnO_4_, UV]. mp = 75–78 °C.

##### 3-Methylene-1-(quinolin-6-yl)­pyrrolidin-2-one (**2e**)

The synthesis of **2e** was performed according to General Procedure B using potassium phosphate (K_3_PO_4_, 175 mg, 0.82 mmol), CuI (12 mg, 0.062 mmol), 6-iodoquinoline (168 mg, 0.66 mmol), *N*,*N*′-dimethylethylenediamine (13 μL, 0.12 mmol), and 3-methylene-2-pyrrolidinone (40 mg, 0.41 mmol) in toluene (4.1 mL) at 109 °C. The silica gel plug (1.5 × 3 cm) was flushed with methanol. The crude residue was purified by silica gel flash column chromatography (2 × 10 cm, 5 mL fractions) eluting with 80% ethyl acetate/hexanes. To remove grease identified by ^1^H NMR, the product was purified further by silica gel flash column chromatography (1 × 10 cm, 5 mL fractions) eluting with 100% ethyl acetate to yield **2e** as a white solid (7 mg, 8%). ^1^H NMR (500 MHz, CDCl_3_): δ 8.86 (d, *J* = 3.1 Hz, 1H), 8.21 (dd, *J* = 2.5, 9.2 Hz, 1H), 8.17–8.11 (m, 3H), 7.40 (dd, *J* = 4.2, 8.2 Hz, 1H), 6.20 (t, *J* = 2.9 Hz, 1H), 5.51 (t, *J* = 2.3 Hz, 1H), 4.00 (t, *J* = 6.8 Hz, 2H), 3.00–2.95 (m, 2H). Water (1.79), grease (1.36–1.18, 0.92–0.79). Small impurities (5.12, 3.36, 1.48). ^13^C NMR (125 MHz, CDCl_3_) δ 167.4, 149.9, 145.7, 139.9, 137.9, 136.1, 130.2, 128.6, 122.7, 121.7, 117.7, 116.5, 45.5, 23.9. IR (thin film) 2960, 2918, 1695, 1658, 1503 cm^–1^. HRMS (ESI) *m*/*z*: [M + H]^+^ calcd for C_14_H_13_ON_2_: 225.1022; found 225.1017. TLC *R_f_* = 0.22 (80% ethyl acetate/hexanes) [silica gel, KMnO_4_, UV].

##### 1-(Benzo­[*b*]­thiophen-3-yl)-3-methylenepyrrolidin-2-one (**2f**)

The synthesis of **2f** was performed according to General Procedure B using potassium phosphate (K_3_PO_4_, 175 mg, 0.82 mmol), CuI (12 mg, 0.062 mmol), 3-bromobenzothiophene (86 μL, 0.66 mmol), *N*,*N*′-dimethylethylenediamine (13 μL, 0.12 mmol), and 3-methylene-2-pyrrolidinone (40 mg, 0.41 mmol) in 1,4-dioxane (4.1 mL) at 115 °C. The silica gel plug (2 × 3 cm) was flushed with ethyl acetate. The crude residue was purified by silica gel flash column chromatography (3 × 14 cm, 5 mL fractions) eluting with 30% ethyl acetate/hexanes to yield **2f** as a white solid (9 mg, 10%). ^1^H NMR (500 MHz, CDCl_3_): δ 7.86–7.81 (m, 1H), 7.74–7.68 (m, 1H), 7.42–7.34 (m, 3H), 6.18 (t, *J* = 2.7 Hz, 1H), 5.50 (t, *J* = 2.2 Hz, 1H), 3.92 (t, *J* = 6.6 Hz, 2H), 3.05–2.98 (m, 2H). Water (1.61). Small impurity (2.17). ^13^C NMR (125 MHz, CDCl_3_) δ 167.5, 139.1, 138.9, 134.3, 132.5, 125.0 124.4, 123.2, 122.7, 119.7, 117.3, 47.7, 25.1. HRMS (ESI) *m*/*z*: [M + H]^+^ calcd for C_13_H_12_ONS: 230.0634; found 230.0630. TLC R_f_ = 0.24 (30% ethyl acetate/hexanes) [silica gel, KMnO_4_, UV].

##### 1-(Isoquinolin-4-yl)-3-methylenepyrrolidin-2-one (**2h**)

The synthesis of **2h** was performed according to General Procedure B using potassium phosphate (K_3_PO_4_, 175 mg, 0.82 mmol), CuI (12 mg, 0.062 mmol), 4-bromoisoquinoline (137 mg, 0.66 mmol), *N,N′*-dimethylethylenediamine (13 μL, 0.12 mmol), and 3-methylene-2-pyrrolidinone (40 mg, 0.41 mmol) in 1,4-dioxane (4.1 mL) at 120 °C. The silica gel plug (2 × 2 cm) was flushed with ethyl acetate. The crude residue was purified by silica gel flash column chromatography (2 × 12 cm, 5 mL fractions) eluting with 80% ethyl acetate/hexanes to yield **2h** as an off-white oily solid (12 mg, 13%). ^1^H NMR (500 MHz, CDCl_3_): δ 9.24 (s, 1H), 8.50 (s, 1H), 8.06 (d, *J* = 8.2 Hz, 1H), 7.77–7.72 (m, 2H), 7.69–7.63 (m, 1H), 6.21 (t, *J* = 2.6 Hz, 1H), 5.56 (br t, *J* = 2.1 Hz, 1H), 3.94 (t, *J* = 6.7 Hz, 2H), 3.14–3.08 (m, 2H). Ethyl acetate (4.12, 2.04), water (1.67), grease (1.32–1.17, 0.91–0.78, 0.07). Small impurities (6.30–6.25, 5.12, 3.35, 2.12–2.10, 2.10–2.07, 1.44–1.41, 1.33, 0.78–0.69). ^13^C NMR (125 MHz, CDCl_3_) δ 168.3, 152.5, 141.1, 138.9, 132.5, 131.2, 131.1, 129.4, 128.3, 128.0, 122.4, 117.7, 48.6, 25.3. Grease (29.8, 1.1), ethyl acetate (60.5, 21.2, 14.3), hexanes (32.0, 22.8, 14.2). Small impurities (56.1, 20.9, 17.6). HRMS (ESI) *m*/*z*: [M + H]^+^ calcd for C_14_H_13_ON_2_: 225.1022; found 225.1018. TLC *R_f_* = 0.1 (80% ethyl acetate/hexanes) [silica gel, KMnO_4_, UV].

#### General Procedure C: *N*-Heteroarylation of 3-Methylene-2-pyrrolidinone (∼0.75 mmol Scale)

An oven-dried 25 mL single-neck, round-bottom flask equipped with a 1 cm Teflon-coated magnetic stir bar and a rubber septum with an argon inlet needle was charged with potassium carbonate (2 equiv), copper­(I) iodide (0.15 equiv), and if a solid, the heteroaryl bromide (1.6 equiv) via temporary removal of the septum. *N*,*N*′-Dimethylethylenediamine (0.3 equiv) was added via syringe, followed by the heteroaryl bromide (if a liquid). The septum was removed and replaced with an oven-dried condenser equipped with a rubber septum. The atmosphere was purged and refilled with Ar (3×). In a separate scintillation vial, 3-methylene-2-pyrrolidinone (1 equiv) was dissolved in degassed 1,4-dioxane (1–2 mL), and this solution was transferred to the reaction flask via syringe. The scintillation vial was rinsed with 1,4-dioxane (1–2 mL) and then added to a flask for a final reaction concentration of 0.1 M. The reaction was refluxed in an oil bath preheated to 111–112 °C. The reaction was maintained until ^1^H NMR indicated consumption of SM. For ^1^H NMR monitoring, an aliquot (0.1 mL) was removed via syringe and concentrated *in vacuo.* The flask was removed from the oil bath and allowed to cool to rt. The reaction mixture was filtered through a silica gel plug and concentrated *in vacuo*. The crude residue was purified by silica gel flash column chromatography.

##### 3-Methylene-1-(2-(trifluoromethyl)­pyrimidin-5-yl)­pyrrolidin-2-one (**1g**)

The synthesis of **1g** was performed according to General Procedure C using potassium carbonate (K_2_CO_3_, 213 mg, 1.5 mmol), CuI (22 mg, 0.12 mmol), 5-bromo-2-(trifluoromethyl)­pyrimidine (280 mg, 1.2 mmol), *N,N′*-dimethylethylenediamine (25 μL, 0.23 mmol), and 3-methylene-2-pyrrolidinone (75 mg, 0.77 mmol) in 1,4-dioxane (7.7 mL). The silica gel plug (2 × 3 cm) was flushed with ethyl acetate. The crude residue was purified by silica gel flash column chromatography (2.5 × 10 cm, 20 mL fractions) eluting with 50% ethyl acetate/hexanes to yield **1g** as a yellow-brown solid (48 mg, 26%). ^1^H NMR (500 MHz, CDCl_3_): δ 9.36 (s, 2H), 6.27 (t, *J* = 2.8 Hz, 1H), 5.62 (t, *J* = 2.4 Hz, 1H), 3.94 (t, *J* = 6.9 Hz, 2H), 3.07–3.03 (m, 2H). Ethyl acetate (4.18, 2.04, and 1.28), water (1.57), grease (1.25 and 0.91–0.86). Small impurities (9.56–8.00, 7.50–7.38, 7.21–6.86, 6.61–5.44, 4.21–3.24, 3.02–2.58, 1.14, 0.07). ^13^C NMR (125 MHz, CDCl_3_) δ 167.9, 151.6 (q, *J* = 37.2 Hz), 146.8, 137.6, 136.3, 120.2, 119.7 (q, *J* = 274.3 Hz), 43.6, 23.9. IR (thin film) 3070, 2923, 1701, 1658 cm^–1^. HRMS (ESI) *m*/*z*: [M + H]^+^ calcd for C_10_H_9_ON_3_F_3_: 244.0692; found 244.0703. TLC *R_f_* = 0.27 (50% ethyl acetate/hexanes) [silica gel, KMnO_4_, UV]. mp = 122–127 °C.

##### 1-(1-Methyl-1*H*-pyrazol-4-yl)-3-methylenepyrrolidin-2-one (**1h**)

The synthesis of **1h** was performed according to General Procedure C using potassium carbonate (K_2_CO_3_, 213 mg, 1.5 mmol), CuI (22 mg, 0.12 mmol), 4-bromo-1-methyl-1*H*-pyrazole (0.13 mL, 1.2 mmol), *N,N′*-dimethylethylenediamine (25 μL, 0.23 mmol), and 3-methylene-2-pyrrolidinone (75 mg, 0.77 mmol) in 1,4-dioxane (7.7 mL). The flask was purged and refilled with argon (1×) after replacing the septum with the condenser due to bubbling of the 4-bromo-1-methyl-1*H*-pyrazole. The silica gel plug (3 × 3 cm) was flushed with ethyl acetate. The crude residue was purified by silica gel flash column chromatography (2.5 × 10 cm, 20 mL fractions) eluting with 80–100% ethyl acetate/hexanes to yield **1h** as a white solid (35 mg, 25%). ^1^H NMR (500 MHz, CDCl_3_): δ 8.11 (s, 1H), 7.49 (s, 1H), 6.07 (t, *J* = 2.7 Hz, 1H), 5.40 (t, *J* = 2.6 Hz, 1H), 3.91 (s, 3H), 3.75 (t, *J* = 6.7 Hz, 2H), 2.94 (m, 2H). Ethyl acetate (4.12, 2.05, and 1.25), water (1.61) and grease (1.24–1.31 and 0.80–0.92). Small impurities (8.00–7.43, 5.25–4.83, 4.20–3.1, 2.51–2.31, 1.38–1.33, 1.09–1.03, 0.07). ^13^C NMR (125 MHz, CDCl_3_) δ 165.5, 139.1, 128.8, 123.4, 121.2, 116.3, 44.4, 39.5, 24.3. IR (thin film) 3161, 3137, 2960, 2928, 2855, 1679, 1655 cm^–1^. HRMS (ESI) *m*/*z*: [M + H]^+^ calcd for C_9_H_12_ON_3_: 178.0975; found 178.0983. TLC *R_f_* = 0.13 (80% ethyl acetate/hexanes) [silica gel, KMnO_4_, UV]. mp = 137–141 °C.

#### General Procedure D: *N*-Heteroarylation of 3-Methylene-2-pyrrolidinone (∼0.4 mmol Scale)

An oven-dried 10–20 mL Biotage microwave vial equipped with a 1 cm Teflon-coated magnetic stir bar and a rubber septum was purged and refilled via an argon-filled balloon (3×). The vial was charged with copper­(I) iodide (0.15 equiv) and base (2 equiv), 3-methylene-2-pyrrolidinone (1 equiv), and if a solid, the heteroaryl halide (1.6 equiv) via temporary removal of the septum. The septum was replaced, and the vial was sealed with a PTFE crimp-cap. The atmosphere was purged and refilled via an argon-filled balloon (3×). Diamine ligand (0.3 equiv) was added to the reaction vial via syringe, followed by the heteroaryl halide, if it was a liquid. In a separate scintillation vial, 3-methylene-2-pyrrolidinone (1 equiv) was dissolved in toluene (if using heteroaryl iodide) or degassed 1,4-dioxane (if using heteroaryl bromide) (1–2 mL), and this solution was transferred to the reaction vial via syringe. Solvent was added to the reaction vial for a final reaction concentration of 0.1 M. The reaction was lowered in a preheated oil bath to the specified temperature. The argon balloon was removed, the cap was wrapped with parafilm, and the reaction was maintained until ^1^H NMR indicated consumption of SM or no further conversion of SM. For ^1^H NMR monitoring, an aliquot (0.1 mL) was removed via syringe and concentrated *in vacuo.* The flask was removed from the oil bath and allowed to cool to rt. The reaction mixture was filtered through a silica gel plug and concentrated *in vacuo*. The crude residue was purified by either silica gel flash column chromatography or recrystallization, as indicated.

##### 3-Methylene-1-(pyridin-4-yl)­pyrrolidin-2-one (**2a**)

The synthesis of **2a** was performed according to General Procedure D using potassium phosphate (K_3_PO_4_, 175 mg, 0.82 mmol), CuI (12 mg, 0.062 mmol), 4-iodopyridine (135 mg, 0.66 mmol), *N*,*N*′-dimethylethylenediamine (13 μL, 0.12 mmol), and 3-methylene-2-pyrrolidinone (40 mg, 0.41 mmol) in toluene (4.1 mL) at 80 °C. The silica gel plug (1 × 2.5 cm silica) was flushed with ethyl acetate. The crude residue was purified by silica gel flash column chromatography (2 × 10 cm, 5 mL fractions) eluting with 100% ethyl acetate to yield **2a** as a white solid (38 mg, 53%). ^1^H NMR (500 MHz, CDCl_3_): δ 8.57 (d, *J* = 5.1 Hz, 2H), 7.71 (dd, *J* = 5.0, 1.4 Hz, 2H), 6.21 (t, *J* = 2.8 Hz, 1H), 5.53 (t, *J* = 2.4 Hz, 1H), 3.86 (t, *J* = 6.9 Hz, 2H), 2.97–2.92 (m, 2H). Water (1.77), grease (1.44–1.24, 0.94–0.86), silicone grease (0.07).^13^C NMR (125 MHz, CDCl_3_) δ 167.9, 150.8, 146.3, 139.3, 118.9, 112.9, 44.2, 23.6. Hexanes (30.6, 23.1, 14.2). Small impurity (67.5, 38.9, 34.1, 30.6, 29.1, 24.7, 23.9, 23.1, 14.2, 11.1). IR (thin film) 2911, 1690, 1658, 1594, 1502, 1476 cm^–1^. HRMS (ESI) *m*/*z*: [M + H]^+^ calcd for C_10_H_11_ON_2_: 175.0866; found 175.0870. TLC *R_f_* = 0.13 (100% ethyl acetate) [silica gel, KMnO_4_, UV]. mp = 132–135 °C.

##### 1-(Benzo­[*b*]­thiophen-2-yl)-3-methylenepyrrolidin-2-one (**2g**)

The synthesis of **2g** was performed according to General Procedure D using potassium phosphate (K_3_PO_4_, 175 mg, 0.82 mmol), CuI (12 mg, 0.062 mmol), 2-iodobenzothiophene (171 mg, 0.66 mmol), *N,N′*-dimethylethylenediamine (13 μL, 0.12 mmol), and 3-methylene-2-pyrrolidinone (40 mg, 0.41 mmol) in toluene (4.1 mL) at 109 °C. The silica gel plug (1 × 0.5 cm silica) was flushed with ethyl acetate. The crude residue was purified by trituration with hexanes (to remove excess 2-iodobenzothiophene) and passive recrystallization with 1,4-dioxane and hexanes to yield **2g** as a white solid (4 mg, 5%). ^1^H NMR (500 MHz, CDCl_3_): δ 7.79 (d, *J* = 8.5 Hz, 1H), 7.66 (d, *J* = 8.5 Hz, 1H), 7.33 (td, *J* = 7.4, 1.1 Hz, 1H), 7.29–7.25 (m, 1H), 6.80 (s, 1H), 6.21 (t, *J* = 2.8 Hz, 1H), 5.53 (t, *J* = 2.4 Hz, 1H), 3.98 (t, *J* = 6.7 Hz, 2H), 3.04–2.99 (m, 2H). Water (1.55 ppm). ^13^C NMR (125 MHz, CDCl_3_) δ 165.6, 140.8, 138.3, 137.1, 136.1, 124.7, 123.5, 122.4, 122.2, 118.5, 106.6, 45.6, 23.9. HRMS (ESI) *m*/*z*: [M + H]^+^ calcd for C_13_H_12_ONS: 230.0634; found 230.0639.

##### 1-(Furan-3-yl)­pyrrolidin-2-one (**2i**)

The synthesis of **2i** was performed according to General Procedure D using potassium phosphate (K_3_PO_4_, 175 mg, 0.82 mmol), CuI (12 mg, 0.062 mmol), 3-bromofuran (59 μL, 0.66 mmol), *trans*-*N*,*N*′-dimethylcyclohexanediamine (19 μL, 0.12 mmol), and 3-methylene-2-pyrrolidinone (40 mg, 0.41 mmol) in 1,4-dioxane (4.1 mL) at 95 °C. The silica gel plug (1 × 2 cm silica) was flushed with ethyl acetate. The crude residue was purified by silica gel flash column chromatography (3 × 12 cm, 5 mL fractions) eluting with 50% ethyl acetate/hexanes to yield **2i** as a red oil (8 mg, 13%). Some degradation was observed by ^1^H NMR within approximately 3 weeks. ^1^H NMR (500 MHz, CDCl_3_): δ 7.95 (s,1H), 7.37 (t, *J* = 1.7 Hz, 1H), 6.75 (d, *J* = 1.2 Hz, 1H), 6.08 (t, *J* = 2.7 Hz, 1H), 5.41 (t, *J* = 2.3 Hz, 1H), 3.72 (t, *J* = 6.8 Hz, 2H), 2.96–2.90 (m, 2H). Acetone (2.17), water (1.56), grease (1.44–1.13, 0.92–0.80). ^13^C NMR (125 MHz, CDCl_3_) δ 166.1, 142.3, 139.1, 131.5, 127.0 116.6, 103.9, 44.5, 24.3. Hexanes (31.7, 23.1, 14.2), grease (29.9). Small impurities (32.1, 31.1, 30.2, 30.1, 29.0, 23.4, 22.8, 22.1, 14.3, 11.1). IR (thin film) 2960, 2925, 1693 cm^–1^. HRMS (ESI) *m*/*z*: [M + H]^+^ calcd for C_9_H_10_O_2_N: 164.0706; found 164.0709. TLC *R_f_* = 0.50 (50% ethyl acetate/hexanes) [silica gel, KMnO_4_, UV].

#### General Procedure for Determination of the Reaction Rate for *N*-Heteroaryl 3-methylene-2-pyrrolidinone via ^1^H NMR

An NMR tube was charged with 0.5 mL of the 2 mM *N*-heteroaryl 3-methylene-2-pyrrolidinone solution. The tube was placed into the probe of a Bruker Avance 600 MHz NMR spectrometer and heated. Once the probe reached 37 °C, a zg30 spectrum—with parameter settings of 16 scans and a relaxation time of 4 s—was taken to establish integrative values for the methylene protons for a zero time point. The tube was promptly removed from the probe, and 0.5 mL of the 20 mM of GSH solution was added. The sample was thoroughly mixed by inverting several times and/or vortexing. The tube was placed into the probe of the 600 MHz NMR spectrometer. Spectra (zg30 pulse program) were taken at 10 min intervals (this excludes the time it takes to collect each spectrum) for approximately 9 h with a total of 46 experiments.

For kinetic experiments measuring the half-life of **2a**, a Bruker Avance 700 MHz NMR was used. Spectra (zg30 pulse program) were taken at 30 s intervals (2.5 min between each data point) for approximately 1 h for a total of 24 experiments. The triplicate experiment for **2c** was performed on the 600 MHz NMR spectrometer with spectra taken at 10 min intervals for 7 h for a total of 35 experiments.

### Papain Protease Inhibition Assay

Experiments below were conducted in a glovebag under an argon atmosphere to minimize exposure of papain to air after initial activation.^39^ 100 mM potassium phosphate buffer (pH 7.4) was sparged with argon for 15 min and transferred to a glovebag (Thermo Scientific, AA93727LK) containing preweighed samples of tris­(2-carboxyethyl)­phosphine hydrochloride [TCEP·HCl], EDTA disodium dihydrate, and papain (Sigma-Aldrich, P5306) along with aliquots of DMSO and 10 μL of each *N*-substituted lactam at 200 mM in DMSO. The glovebag was sealed with duct tape, checked for leaks, and filled with argon in three pump/purge cycles. All manipulations below were carried out in the glovebag under argon unless otherwise noted. Two buffers were prepared: 1 mL of 2 mM TCEP, 1.6 mM EDTA, 100 mM phosphate pH 7.4 (buffer A) and 1 mL of 1.6 mM EDTA, 100 mM phosphate pH 7.4 (buffer B). A 0.5–1 mL solution was prepared in buffer A to a final concentration of 400 μM papain, and this solution allowed to stand for 90 min to activate the enzyme. To initiate the reaction between papain and *N*-substituted lactam, 48 μL of buffer B was added to 2 μL of DMSO or inhibitor dissolved in DMSO, followed by 50 μL of activated papain in buffer A. The final assay composition was 200 μM enzyme, 1 mM TCEP, 1.6 mM EDTA, and 100 mM phosphate pH 7.4, with 1% DMSO and either 0 or 2 mM *N*-substituted lactam (100 μL total volume). Reactions were allowed to stand at room temperature for 22 h. The glovebag seal was then broken and remaining steps performed under atmospheric conditions. In individual wells of a clear, flat-bottomed 96-well bacti plate (Thermo Scientific, 269620), 10 μL of each inhibitor-treated enzyme sample was added to 40 μL of buffer B. Chromogenic substrate (*N*_α_-benzoyl-l-arginine 4-nitroanilide hydrochloride; 50 μL, 2 mM, Millipore Sigma, B3133) in 100 mM potassium phosphate pH 7.4 with 1% DMSO was then added, and the enzyme activity was measured. The final enzyme activity reaction composition included 20 μM papain, 100 μM TCEP, 0.8 mM EDTA, 0.6% DMSO, 0 or 0.2 mM *N*-substituted lactam, and 1 mM substrate in 100 mM potassium phosphate at pH 7.4 (100 μL total volume). Enzymatic hydrolysis of the chromogenic substrate was monitored on a Tecan M1000 plate reader. Absorbance at 400 nm background corrected to absorbance at 790 nm was measured every minute for 1 h at 28 °C. Initial rates were determined using the slopes derived from the first 15 min of data via linear regression (Figure S63). Normalized activity (*E*/*E*_o_) was calculated by the ratio of the rate of substrate consumption by enzyme incubated with an inhibitor to the corresponding rate of substrate consumption by enzyme treated under identical conditions without an inhibitor (Figure S64). The enzyme activity was determined in triplicate for each inhibitor.

## Supplementary Material







## Notes

Properties of heteroarenes; results of GSH reactivity studies; ^1^H, ^13^C, and DEPT NMR spectra of compounds; details of computational studies; Cartesian coordinates; energies of optimized structures; details of papain protease inhibition assay; and HPLC purity traces (PDF)
Molecular formula strings (CSV)
FAIR data, including primary NMR FID files, for compounds **1a**–**1h** and **2a**–**2i** (ZIP)

## Abbreviations

CRG: covalent reactive group
DFT: density functional theory
EA: electron affinity
GSH: glutathione
Het: heteroaryl group
LUMO: lowest-unoccupied molecular orbital
MUE: mean unsigned error
NPA: natural population analysis (or *N* **-**phenylacrylamide where specified)
PBS: phosphate buffer solution
