# Supplementary material for: A Predictive Model for Thiol Reactivity of N‑Heteroaryl α‑Methylene−γ-Lactams—A Medicinally Relevant Covalent Reactive Group
Source: J Med Chem. 2025 May 23;68(11):11948–61. doi: 10.1021/acs.jmedchem.5c00833 (PMC12169685; doi:10.1021/acs.jmedchem.5c00833)
Supplement: Supplementary file 1 [file jm5c00833_si_001.pdf]

SUPPORTING INFORMATION

A predictive model for thiol reactivity of *N*-heteroaryl $\boldsymbol{\alpha}$-methylene–$\boldsymbol{\gamma}$-lactams—a medicinally relevant covalent reactive group

Mariah C. Meehan,[‡] Grace E. Scofield,[‡] Corrinne E. Stahl, Jacob A. Wolfe, W. Seth Horne,[*] Peng Liu,[*] and Kay M. Brummond[*]

Department of Chemistry, University of Pittsburgh, Pittsburgh, Pennsylvania 15260, United States

kbrummon@pitt.edu, pengliu@pitt.edu, horne@pitt.edu

Table of Contents

DETERMINATION OF THE REACTION RATES FOR *N*-HETEROARYL 3-METHYLENE-2-PYRROLIDINONE VIA ^{1}H NMR . 6

WORKFLOW FOR ANALYZING ^{1}H NMR RATE DATA 8

THIOL REACTIVITY FIRST-ORDER PLOTS AND CONTROL EXPERIMENTS........ 9

^{1}H NMR and ^{13}C NMR SPECTRA 28

COMPUTATIONAL DETAILS AND ADDITIONAL COMPUTATIONAL RESULTS 56

Thiol-Michael transition state analysis for the addition of methyl thiolate to 1a-1h 57

Linear regression models for predicting $\Delta G^{\ddagger}_{DFT}$ 59

Correlation between $\Delta G^{\ddagger}_{DFT}$ and the LUMO energy of *N*-heteroaryl *α*-methylene-*γ*-lactams and unsubstituted heteroarenes 64

Correlation between $\Delta G^{\ddagger}_{DFT}$ and the LUMO coefficients at C^{α} and C^{β} of *N*-heteroaryl *α*-methylene-*γ*-lactams 66

Correlation between $\Delta G^{\ddagger}_{exp}$ and the electron affinity of *N*-heteroaryl *α*-methylene-*γ*-lactams (see main text for correlation with $\Delta G^{\ddagger}_{DFT}$) 68

Correlation between $\Delta G^{\ddagger}_{DFT}$ and heteroaryl Hammett-type substituent constants (σ_{Het}) 69

Correlation between $\Delta G^{\ddagger}_{DFT}$ and Sterimol L, B_1, and B_5 values 73

Correlation between $\Delta G^{\ddagger}_{DFT}$ and NICS(0)$_{zz}$ and NICS(1)$_{zz}$ aromaticity indices of heteroarenes and *N*-heteroaryl lactams 76

Correlation between $\Delta G^{\ddagger}_{DFT}$ and *N*-Het dihedral angle (ϑ) 81

Correlation between $\Delta G^{\ddagger}_{DFT}$ and NPA charge of C^{β} of *N*-heteroaryl *α*-methylene-*γ*-lactams in the ground state .. 82

Correlation between $\Delta G^{\ddagger}_{DFT}$ and chemical shift of $H^{\beta 1}$ (^{1}H NMR) and C^{β} (^{13}C NMR) of *N*-heteroaryl *α*-methylene-*γ*-lactams 83

Development of a single-parameter thiol reactivity predictive model using additional *N*-heteroaryl lactams as a test set 84

Development of a single-parameter thiol reactivity predictive model using thiol-reactive acrylamide warheads as a second test set 85

Calculating the weighted $\Delta G^{\ddagger}_{predicted}$ value for 1e, 2a, and 3c 88

CARTESIAN COORDINATES 91

SUMMARY OF MEASURED RATES OF GSH ADDITION FOR TEST SET 108

PAPAIN PROTEASE INHIBITION ASSAY 109

HPLC PURITY 110

REFERENCES 136

Heteroarenes and Factors Considered for Inclusion in This Study

Table S1. Heteroarenes selected for studies[a]

	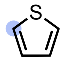	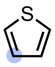	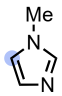	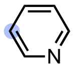	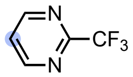	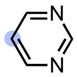	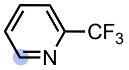	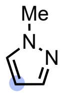
**Prevalence in FDA approved drugs[b]**	16	16	30	54	–	20	–	6
**LUMO (eV)[c]**	0.67	0.79	0.81	0.58	-0.01	0.36	0.36	1.02
**Commercially available halide (as shown)**	Y	Y	Y	Y	Y	Y	Y	Y
**cLogP**	1.79	1.79	-0.03	0.84	–	0.26	–	0.24
**H-bonding potential (pK_{BHX})**	-0.50	-0.50	2.42	1.86	–	1.07	–	–
**Basicity (pK_{aH})[d]**	5.11	6.17	5.81	4.86	2.59	3.73	4.30	6.56
**Dipole μ**	0.53	0.53	3.80	2.22	–	2.33	–	2.21
**Aromaticity I_A[e]**	81.5	81.5	79	86	–	84	–	90
**TPSA (Å²)**	0	0	24.39	12.9	–	25.8	–	24.39
**Ionization Potential (eV)**	8.87	8.87	8.78	9.66	–	9.63	–	9.15

[a]Values for physicochemical properties correspond to the unsubstituted heteroarene rather than the *N*-heteroaryl lactam unless otherwise noted.[1] For the imidazole and pyrazole heteroarenes, the available values correspond to the unsubstituted nitrogen (*i.e.*, hydrogen instead of a methyl group). In cases where the *N*-heteroaryl lactam or heteroaryl carboxylic acid was used, the blue dot indicates the substitution position of the heteroarene, and the pyrazole and imidazole possess the *N*-Me substitution shown; [b]Prevalence in 1175 FDA approved drugs prior to 2014;[1] [c]Values were calculated for the *N*-heteroaryl lactam using Gaussian16 (ωB97X-D/6-31G(d)); [d]Values were calculated for the heteroaryl carboxylic acid (see Figure S38 for details). [e]Aromaticity index.

Table S2. Heteroarenes not selected for studies[a]

	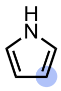	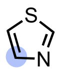	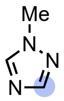	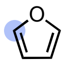	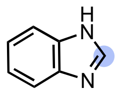	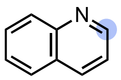
**Prevalence in FDA approved drugs[b]**	3	25	–	11	14	14
**LUMO (eV)[c]**	1.22	0.78	1.05	0.95	0.38	0.29
**Commercially available halide (as shown)**	N	Y	Y	Y	Y	Y
**cLogP**	0.75	0.49	-0.89	1.32	1.32	2.13
**H–bonding potential (pK_{BHX})**	0.15	1.37	–	-0.40	–	1.89
**Basicity (pKa)**	–	–	–	–	–	–
**Dipole μ**	1.74	1.61	2.72	0.66	3.96	2.2
**Aromaticity I_A[e]**	85	79	–	53	148	134
**TPSA (Å^2)**	12.03	12.36	41.6	9.23	28.7	12.9
**Ionization Potential (eV)**	8.23	9.5	–	8.89	8.84	8.62

[a]Values for physicochemical properties correspond to the unsubstituted heteroarene rather than the *N*-heteroaryl lactam unless otherwise noted.[1] For the triazole, the available values correspond to the unsubstituted nitrogen (i.e. hydrogen instead of a methyl group). In cases where the *N*-heteroaryl lactam was used, the blue dot indicates the substitution position of the heteroarene, and the triazole possesses the *N*-Me substitution shown; [b]Prevalence in 1175 FDA approved drugs prior to 2014[1] [c]Values were calculated for the *N*-heteroaryl lactam using Spartan'20 (ωB97XD/6-31G(d)). [e]Aromaticity index.

DETERMINATION OF THE REACTION RATES FOR *N*-HETEROARYL 3-METHYLENE-2-PYRROLIDINONE VIA ^{1}H NMR

Determination of reaction rates for the addition of GSH to *N*-heteroaryl 3-methylene-2-pyrrolidinones was accomplished by monitoring the disappearance of the methylene resonances over time using ^{1}H NMR. The reactions were performed using a single lactam. Spectra (zg30 pulse program) were taken at fixed intervals every 10 min (this excludes the time it takes to collect each spectrum) for 9.2 h, for a total of 46 experiments. Data processing was accomplished using TopSpin. Integration values for signals corresponding to the methylenyl protons of the lactam (6.2–5.4 ppm) were integrated and calibrated to either the residual dimethyl sulfoxide peak (at approximately 2.71 ppm) or residual acetonitrile in a sealed capillary tube (at approximately 2.87 ppm). For (**2b-2g**), the internal standard was not included in the analysis due to broadening of the residual acetonitrile peak, resulting in inconsistencies of the internal standard.

Preparation of Solutions for Rate Studies

Phosphate Buffer Solution (100 mM, 90% H_2O/ 10% D_2O)
A 100-mL volumetric flask was charged with phosphate buffer solution (20 mL of a 0.5 M solution, pH 7.4) and deuterium oxide (8 mL) using a micropipette. The flask was filled to the line with Milli-Q ultrapure distilled and deionized water to make a solution in 90% water and 10% deuterium oxide. The flask was capped and vigorously shaken for 1 min.

Phosphate Buffer Solution (100 mM, 100% D_2O)
A 50-mL Falcon tube was charged with deuterium oxide (32 mL) using a Serological pipette, followed by potassium phosphate dibasic (484.8 mg), potassium phosphate monobasic (165.6 mg), and deuterium oxide (4 mL) using a Serological pipette. The quantities of what were obtained using https://www.aatbio.com/resources/buffer-preparations-and-recipes/potassium-phosphate-ph-5-8-to-8-0. The tube was capped and vigorously shaken for 1 min. The pH of the solution was adjusted to a pH of 7.4 using solutions of 1 M HCl (in deuterium oxide) and 1 M NaOH (in deuterium oxide) measuring with a pH meter. After the pH adjustment, the final volume was 40 mL.

N-heteroaryl 3-methylene-2-pyrrolidinone stock solutions in DMSO-d_6 (200 mM): A 1-dram vial was accurately weighed. Each *N*-heteroaryl 3-methylene-2-pyrrolidinone (ca. 2 mg) was added to the vial and the accurate weight was recorded. Note: the balance was allowed to equilibrate for 5 min for both the vial and addition of lactam. A micropipette was used to transfer dimethylsulfoxide-d_6 to the vial to achieve a 200 mM stock solution. Calculations for quantity of solvent required for each *N*-heteroaryl 3-methylene-2-pyrrolidinone to prepare a 200 mM solutions were obtained using volume from mass & concentration in Molarity Calculator (https://www.graphpad.com/quickcalcs/Molarityform.cfm).

N-heteroaryl 3-methylene-2-pyrrolidinone solutions (2 mM): 20 μL of the 200 mM stock *N*-heteroaryl 3-methylene-2-pyrrolidinone solution was added to a 20-mL scintillation vial using a micropipette and diluted in 100 mM PBS buffer (1980 μL) to make a 2 mM solution. The vial was sonicated for ~ 1min then vortexed for ~1 min until complete dissolution.

Glutathione solution (20 mM): Glutathione (ca. 10 mg) was accurately weighed into a 20-mL scintillation vial. Using a micropipette, an exact amount of 100 mM PBS buffer was added to the vial to create a 20 mM solution. Calculations for the GSH solution were confirmed using Molarity

Calculator (https://www.graphpad.com/quickcalcs/Molarityform.cfm). The vial was vortexed for 1 min.

WORKFLOW FOR ANALYZING ^{1}H NMR RATE DATA

Upon collection of 46 experiments, “multicmd” is typed into the command line which prompts the following questions: “NORMAL (ok), first experiment (#), number of experiments (#), commands: exponential window and fourier transform (ef), automatic phase correction (apk), and automatic baseline correction (abs).” Before the addition of GSH, a zero-time point spectrum was obtained to integrate the values for the α–methylene protons and the internal standard, either residual DMSO or residual acetonitrile peak. Integrated regions were saved as ‘intrng’ and ‘reg’ and file was renamed (wmisc and type in file name). Multi-integration was performed (multi_integ, prompts following questions: 0 expos, number of experiments, file name). Integration values for all experiments are available as a text file (intall.txt) in the folder entitled “pdata” in the zero-time point experiment folder.

In some cases, when the lactam had been consumed or nearly consumed, the α–methylenyl proton integration values were negative due to fluctuations in the baseline. These data points were removed due to the inability to take the natural logarithm of a negative number.

Note: Values reported in Table 1 were determined using the calculated mean of three half-lives.

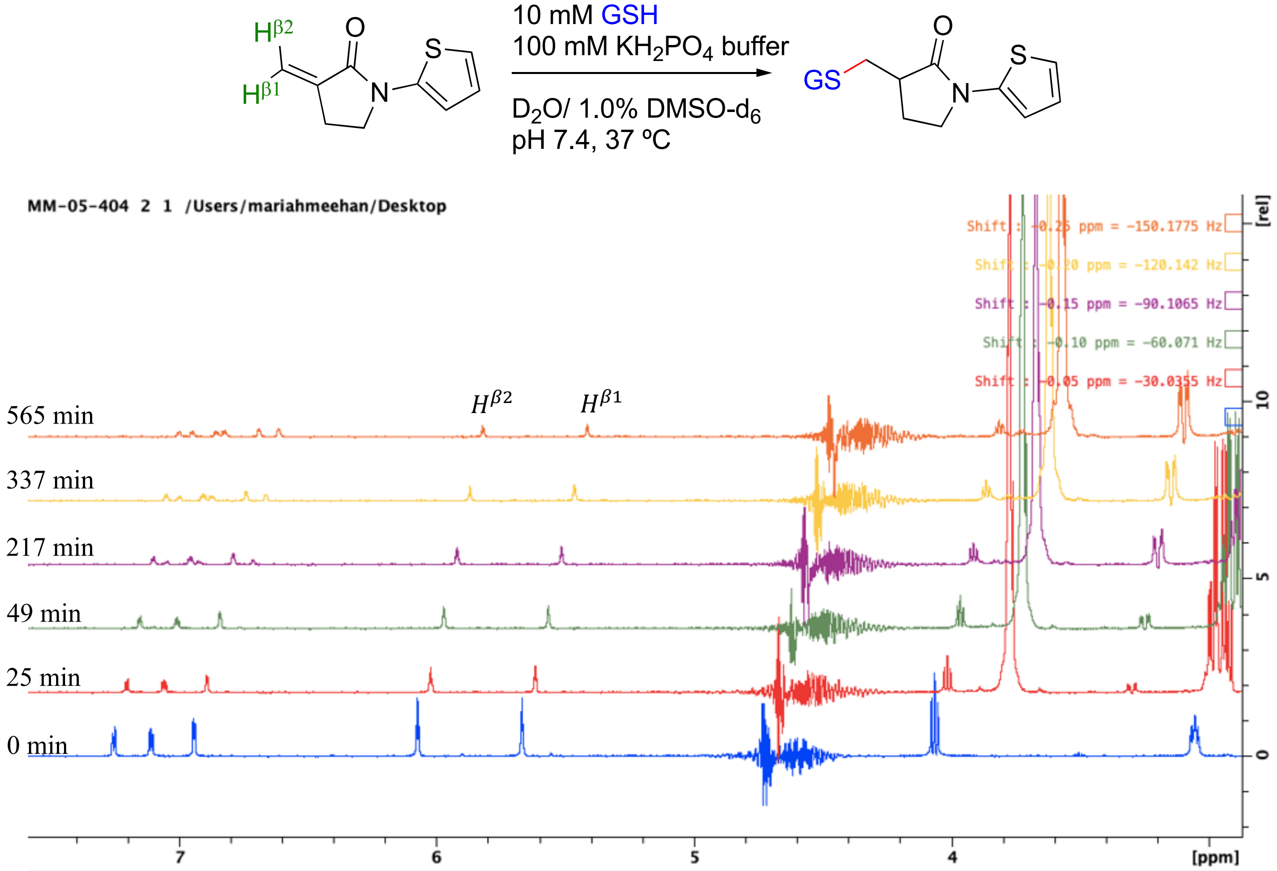

Figure S1. Monitoring reaction progress of **1c** with GSH by ^{1}H NMR.

THIOL REACTIVITY FIRST-ORDER PLOTS AND CONTROL EXPERIMENTS

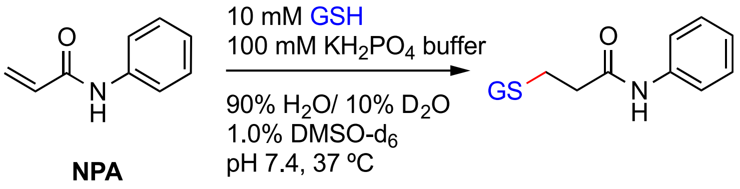

a. Runs 1

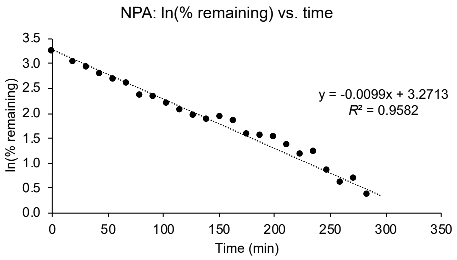

Figure S2. Reaction of *N*-phenyl-acrylamide (**NPA**) with glutathione (GSH) a. Plot of ln(% remaining) vs. time for **NPA**. The data was truncated at 300 min. The half-life of **NPA** was 70 min using the ^{1}H NMR method described above, whereas the previously reported half-life was 53 min using an LC-MS method; however, the difference in $\Delta G^{\ddagger}_{exp}$ is negligible (e.g.**,** 23.55 vs. 23.37 kcal/mol) demonstrating that the reaction rates determined using either LC-MS or ^{1}H NMR can be directly compared for NPA.

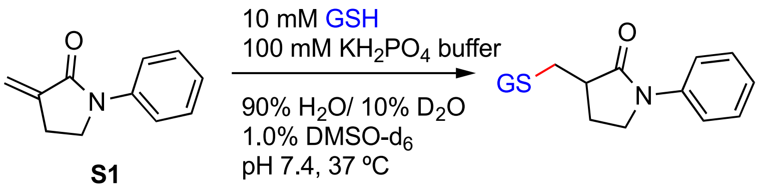

a. Runs 1–2

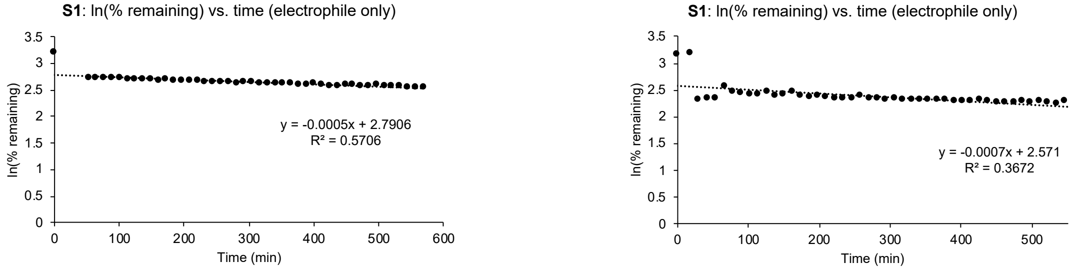

Figure S3. Reaction of *N*-phenyl-3-methylene-2-pyrrolidinone (**S1**) with GSH performed in duplicate on different days a. Plot of ln(% remaining) vs. time for **S1**. The half-life of **S1** was 1188 min via the ^{1}H NMR method, whereas the previously reported half-life was 1386 min using an LC-MS method; however, the difference in $\Delta G^{\ddagger}_{exp}$ is negligible (e.g., 25.29 vs. 25.39 kcal/mol) demonstrating that the reaction rates measured using either LC-MS or ^{1}H NMR can be directly compared for **S1.**

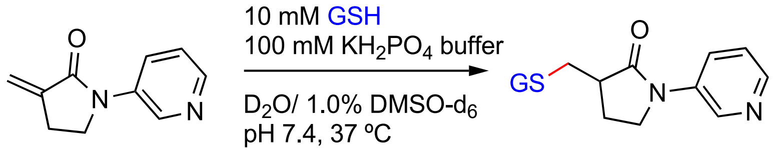

1a

a. Runs 1–3

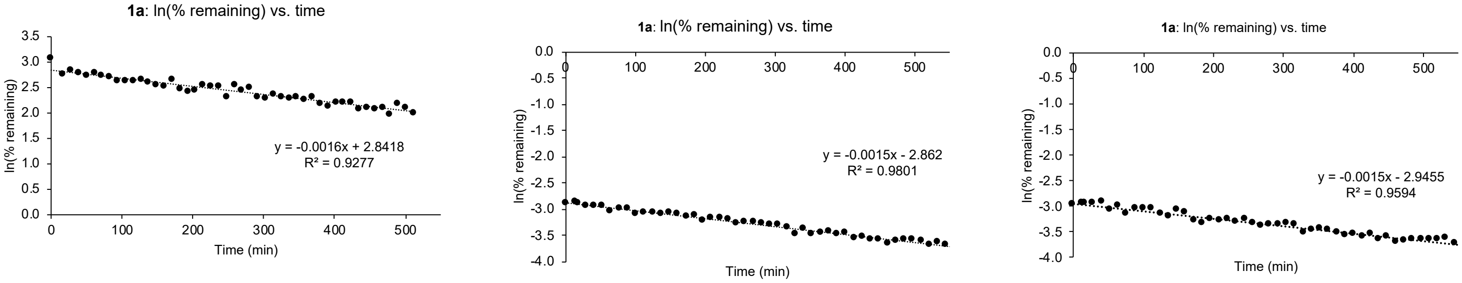

Figure S4. Reaction of lactam **1a** reacted with GSH performed in triplicate on different days. a. Plot of ln(% remaining) vs. time for **1a**.

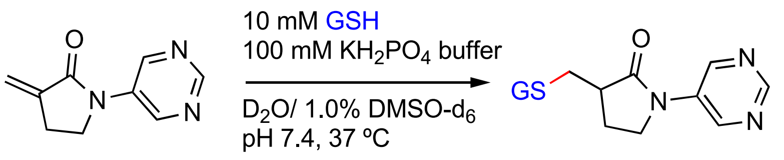

1b

a. Runs 1–3

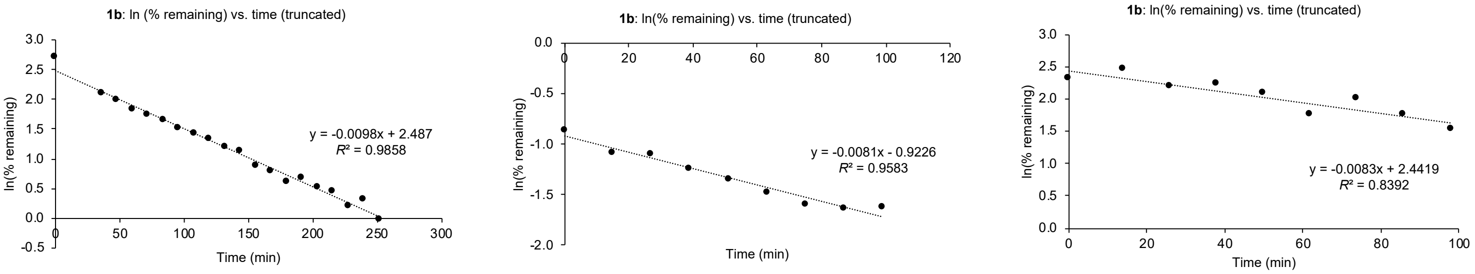

Figure S5. Reaction of lactam **1b** with GSH performed in triplicate on different days. a. Plot of ln(% remaining) vs. time for **1b**. Data was truncated at 100-250 min due to the deviation from pseudo-first order kinetics. For Run 1, the PBS solution in 90% H_2O/ 10% D_2O was utilized.

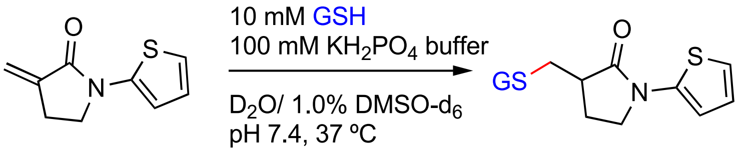

a. Runs 1–3

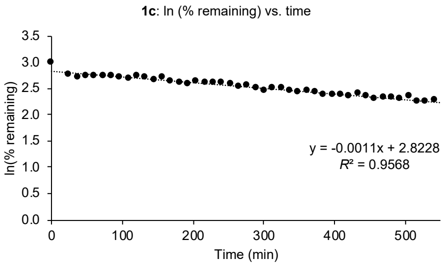

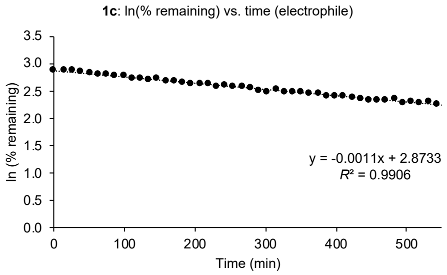

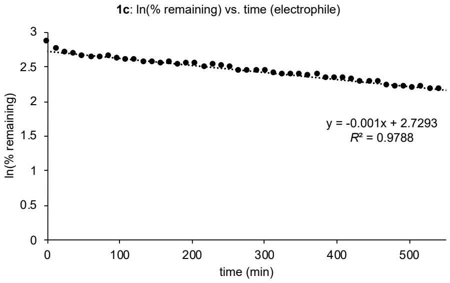

Figure S6. Reaction of lactam **1c** with GSH performed in triplicate on different days. a. Plot of ln(% remaining) vs. time for **1c**. For Runs 2 and 3, only the integrals for **1c** were used, not the internal standard peak as it was overlapping with other signals in the spectrum. For Run 1, the PBS solution in 90% H_2O/ 10% D_2O was utilized.

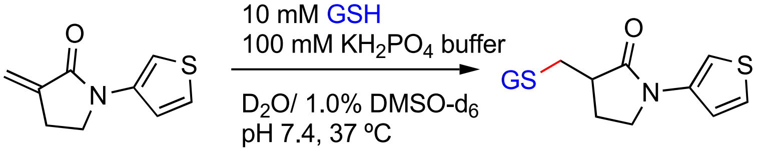

1d

a. Runs 1–3

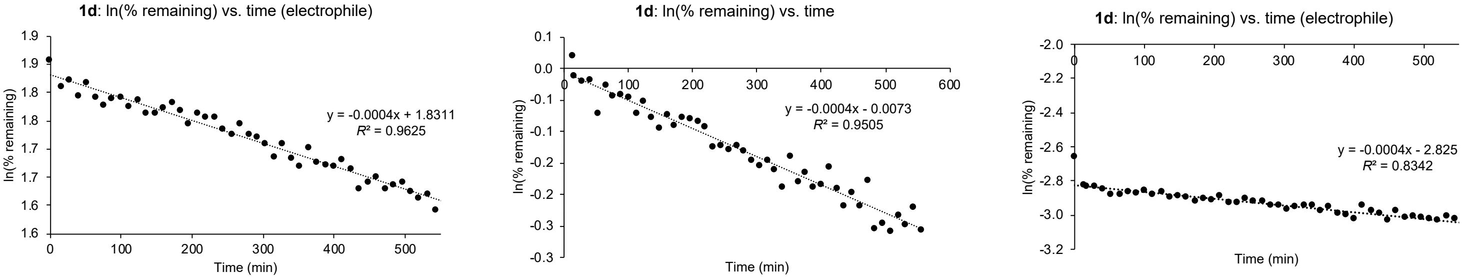

Figure S7. Reaction of lactam **1d** with GSH performed in triplicate on different days. a. Plot of ln(% remaining) vs. time for **1d**. For Runs 1 and 3, only the integrals for **1d** were used, as the internal standard peak was overlapping with other signals in the spectrum. For Run 1, the PBS solution in 90% H_2O/ 10% D_2O was utilized.

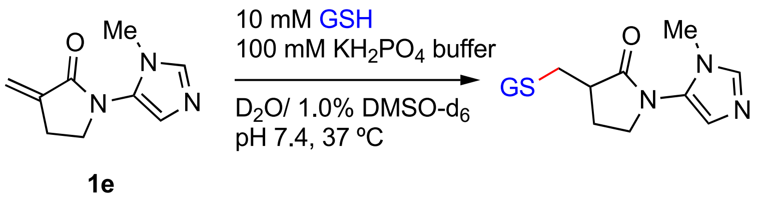

a. Runs 1–3

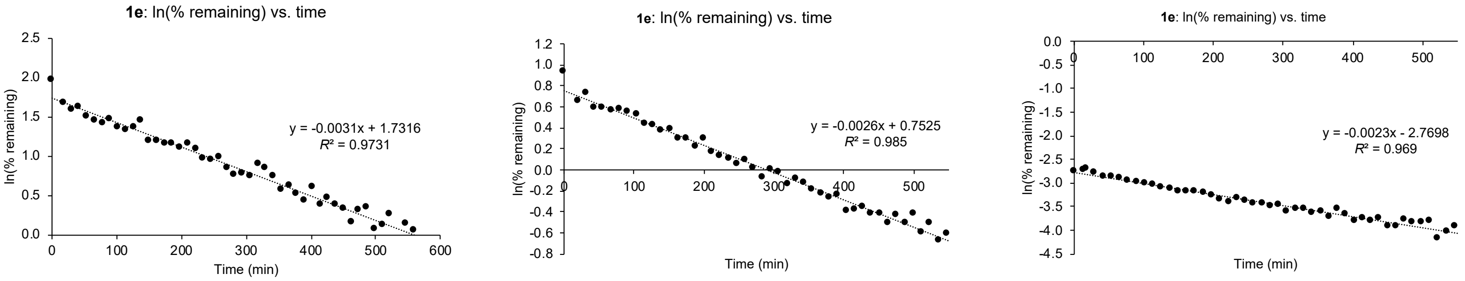

Figure S8. Reaction of lactam **1e** with GSH in triplicate performed on different days. a. Plot of ln(% remaining) vs. time for **1e**.

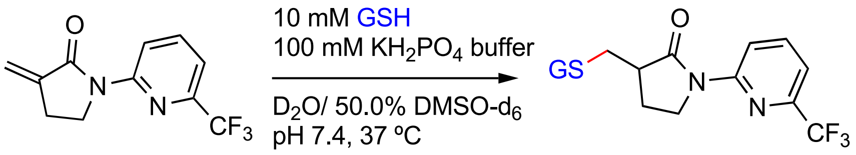

1f

a. Runs 1–3

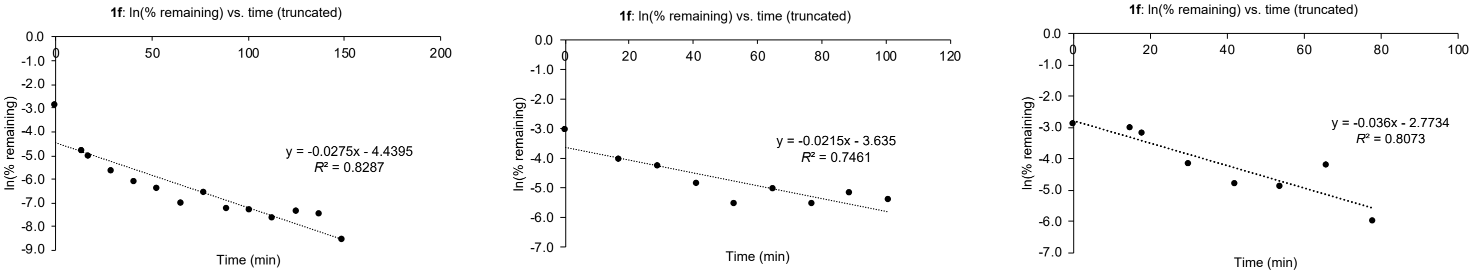

Figure S9. Reaction of lactam **1f** with GSH in triplicate performed on different days. Due to solubility issues, **1f** was dissolved in 100% DMSO-d_6, instead of 1% DMSO-d_6 as used for the other lactams (attempts to dissolve **1f** in 30% and 50% DMSO-d_6 solutions were also unsuccessful). During the addition of GSH in PBS to **1f** in 100% DMSO-d_6, the NMR tube became warm to the touch. To quantify this exothermicity, a two-necked, 5-mL round-bottom flask equipped with an internal thermometer and stir bar was charged with DMSO (0.5 mL) followed by PBS (0.5 mL). A substantial increase in temperature (21–23 ℃ to 33–35 ℃) was measured during the addition. a. Plot of ln(% remaining) vs. time for **1f**. Data was truncated at 80-150 min due to the deviation from pseudo-first order kinetics.

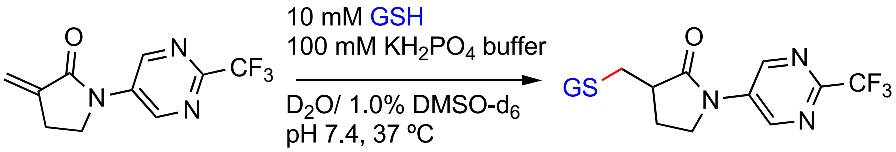

1g

a. Runs 1–3

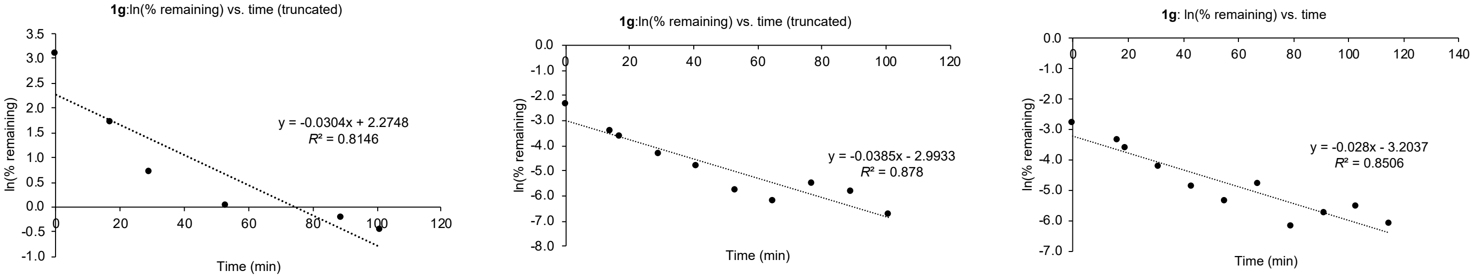

Figure S10. Reaction of lactam **1g** with GSH in triplicate performed on different days. a. Plot of ln(% remaining) vs. time for **1g**. Data was truncated at 100-120 min due to the deviation from pseudo-first order kinetics. For Run 1, the PBS solution in 90% H_2O/ 10% D_2O was utilized.

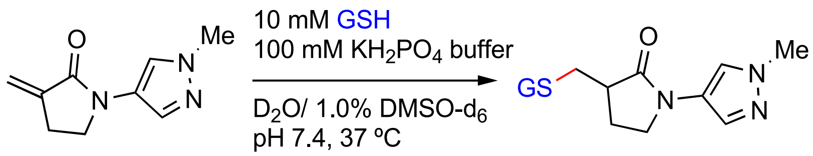

1h

a. Runs 1–3

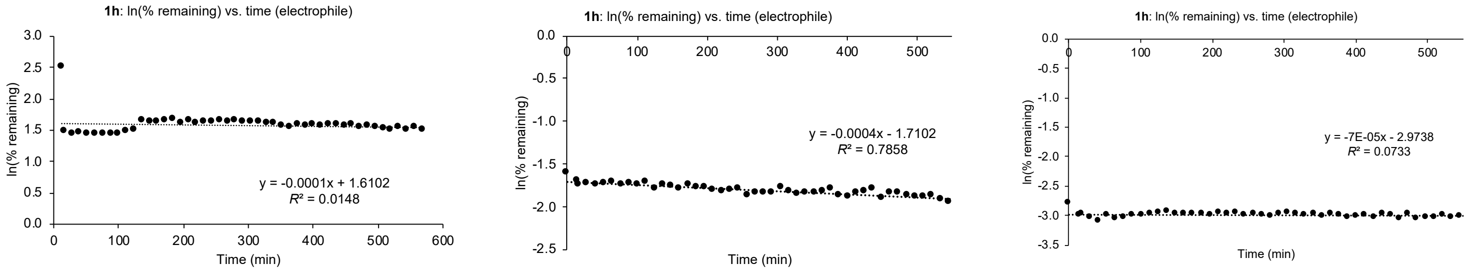

Figure S11. Reaction of lactam **1h** with GSH in triplicate performed on different days. a. Plot of ln(% remaining) vs. time for **1h**. For all three runs, only the integrals for **1h** were used, as the internal standard peak was overlapping with other signals in the spectrum.

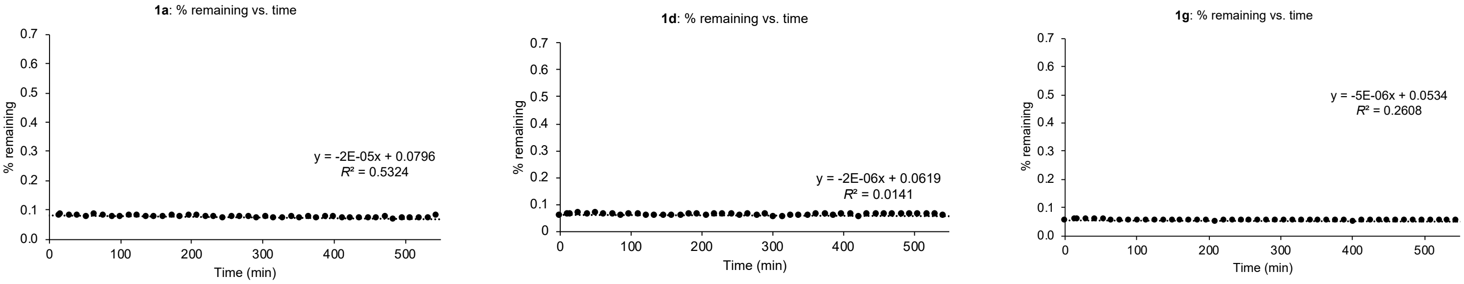

Figure S12. Three additional control experiments were performed to further support no reaction occurs without the addition of GSH to the reaction mixture on different days. a) Plot of % remaining vs. time for **1a**, **1d**, and **1g**.

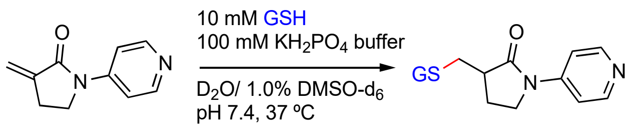

2a

a. Runs 1–3

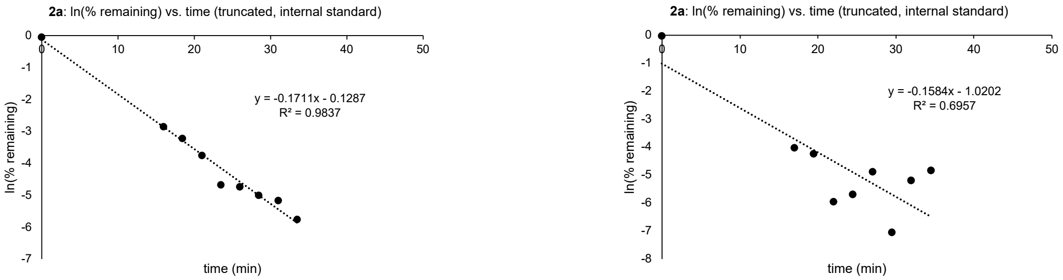

Figure S13. Reaction of lactam **2a** with GSH in duplicate performed on different days. a. Plot of ln(% remaining) vs. time for **2a**. Data was truncated at 35-40 min due to the deviation from pseudo-first order kinetics.

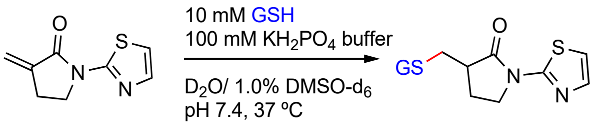

2b

a. Runs 1–2

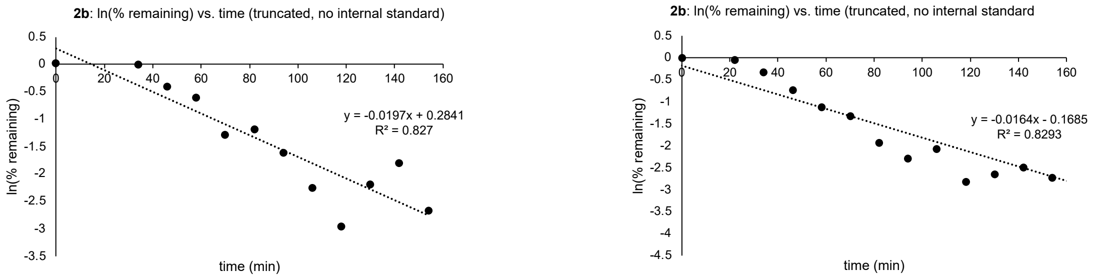

Figure S14. Reaction of lactam **2b** with GSH in duplicate performed on different days. a. Plot of ln(% remaining) vs. time for **2b**. For Runs 1 and 2, only the integrals for **2b** were used, not the internal standard peak. Data was truncated at 150-160 min due to the deviation from pseudo-first order kinetics.

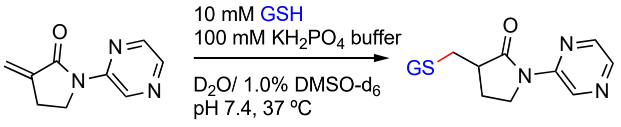

2c

a. Runs 1–3

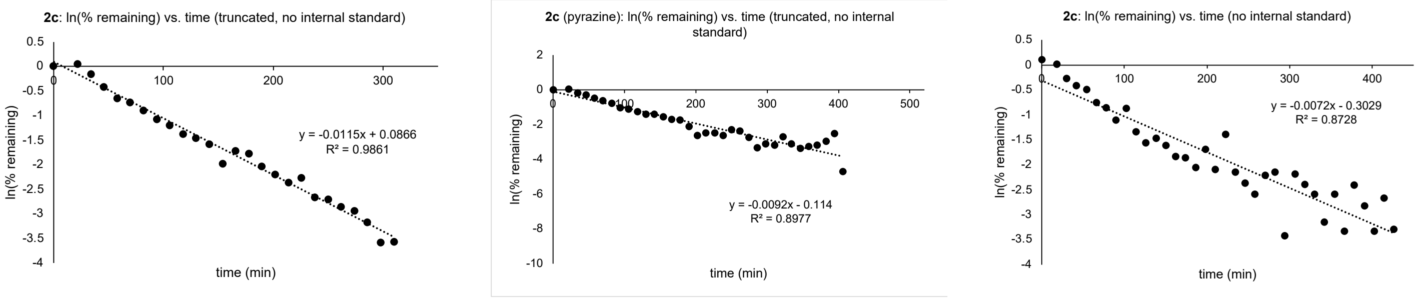

Figure S15. Reaction of lactam **2c** with GSH in triplicate performed on different days. a. Plot of ln(% remaining) vs. time for **2c**. For Runs 1-3, only the integrals for **2c** were used, not the internal standard peak. Data was truncated at 300–400 min due to the deviation from pseudo-first order kinetics for Runs 1-2.

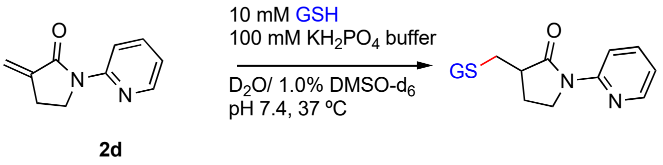

a. Runs 1–2

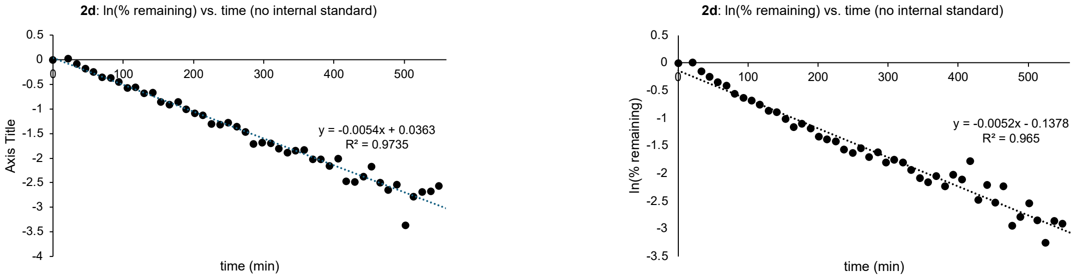

Figure S16. Reaction of lactam **2d** with GSH in duplicate performed on different days. a. Plot of ln(% remaining) vs. time for **2d**. For Runs 1 and 2, only the integrals for **2d** were used, not the internal standard peak.

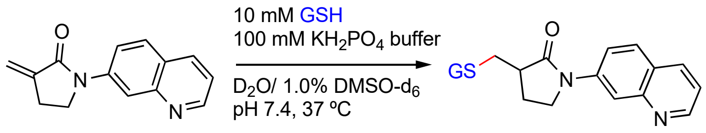

2e

a. Runs 1–3

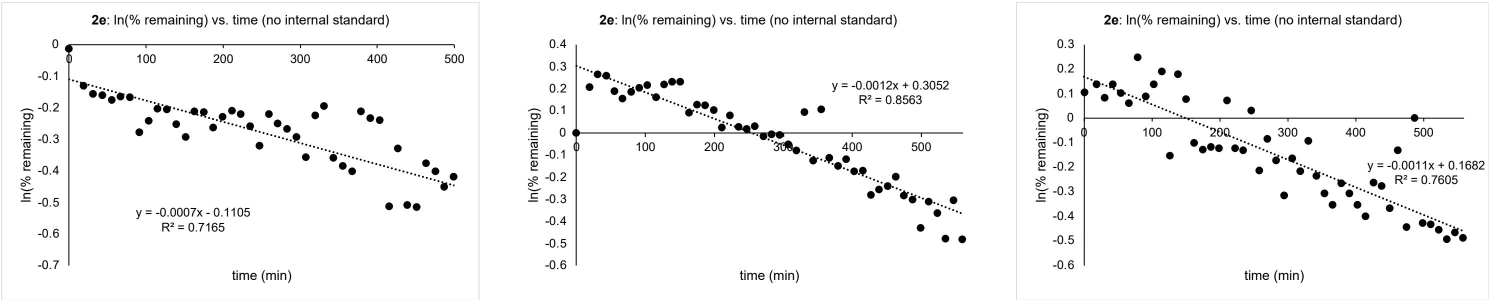

Figure S17. Reaction of lactam **2e** with GSH in triplicate performed on different days. a. Plot of ln(% remaining) vs. time for **2e**. For Runs 1-3, only the integrals for **2e** were used, not the internal standard peak.

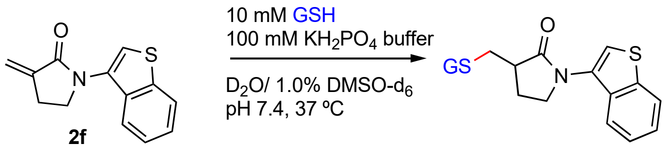

a. Runs 1–2

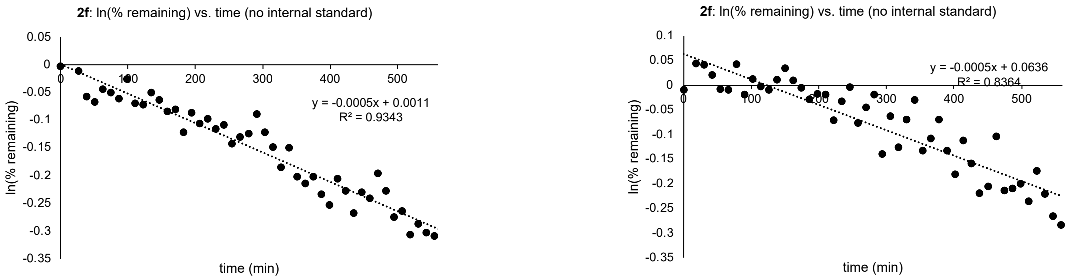

Figure S18. Reaction of lactam **2f** with GSH in duplicate performed on different days. a. Plot of ln(% remaining) vs. time for **2f**. For Runs 1 and 2, only the integrals for **2f** were used, not the internal standard peak.

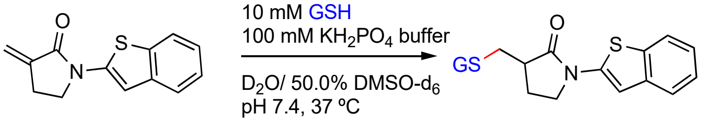

2g

a. Runs 1–3

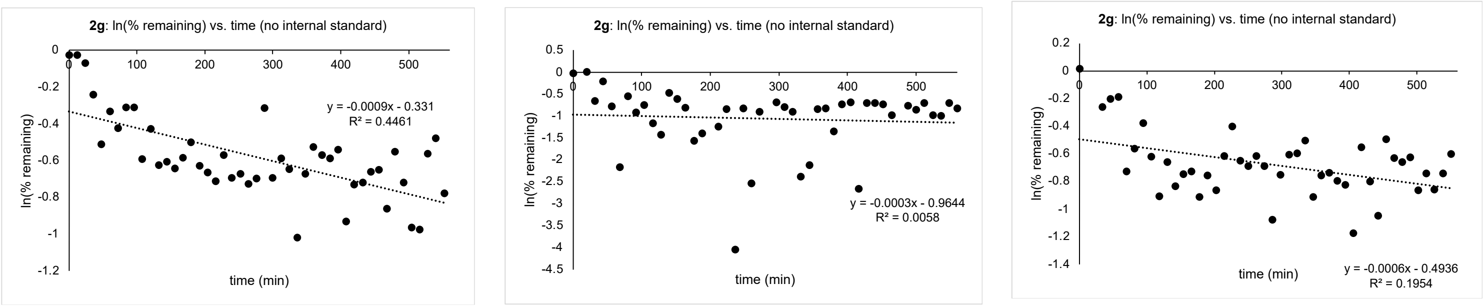

Figure S19. Reaction of lactam **2g** with GSH in triplicate performed on different days. Due to solubility issues, **2g** was dissolved in 100% DMSO-d_6, instead of 1% DMSO-d_6 as used for the other lactams. a. Plot of ln(% remaining) vs. time for **2g**. For Runs 1-3, only the integrals for **2g** were used, not the internal standard peak.

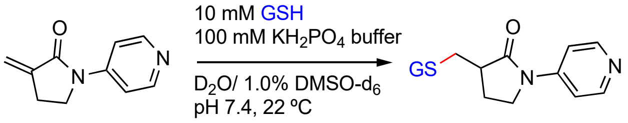

2a

a. Runs 1–2 for **2a**

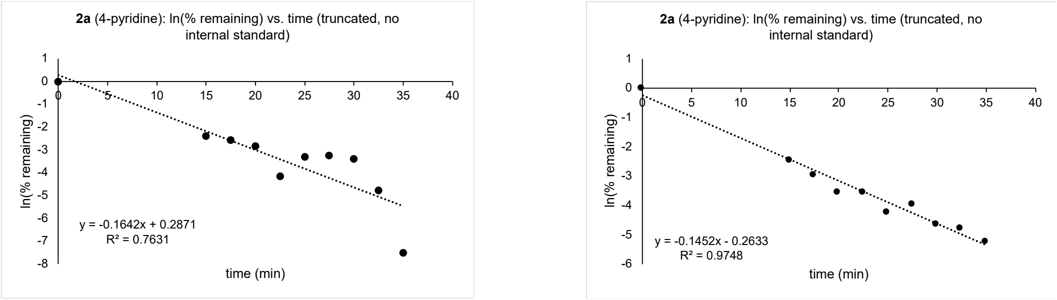

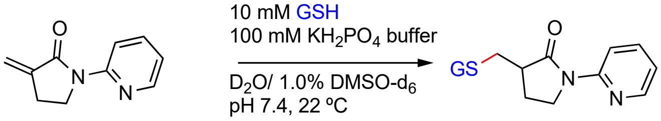

2d

b. Runs 1–2 for **2d**

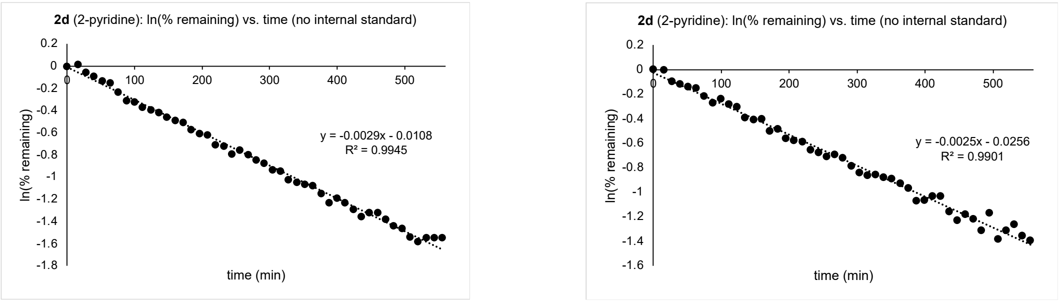

Figure S20. Determination of half-lives for **2a** and **2d** at 22 ºC. a. Reaction of lactam **2a** with GSH in duplicate performed on different days at 22 ºC. Plot of ln(% remaining) vs. time for **2a**. For Runs 1-2, only the integrals for **2a** were used, not the internal standard peak. Data was truncated at 35 min due to the deviation from pseudo-first order kinetics for Runs 1-2. The average half-life was 5 min. b. Reaction of lactam **2d** with GSH in duplicate performed on different days at 22 ºC. Plot of ln(% remaining) vs. time for **2d**. For Runs 1-2, only the integrals for **2d** were used, not the internal standard peak. The average half-life was 258 min. Based on the kinetic experiments performed at 22 ºC, **2a** reacts approximately 57 times faster than **2d**. The relative reactivity between these two lactams suggests that the short half-life for **2a** measured at 37 ºC ($t_{1/2}$ = 4 min) is within a reasonable range.

^{1}H NMR and ^{13}C NMR SPECTRA

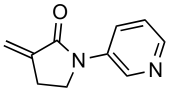

1a

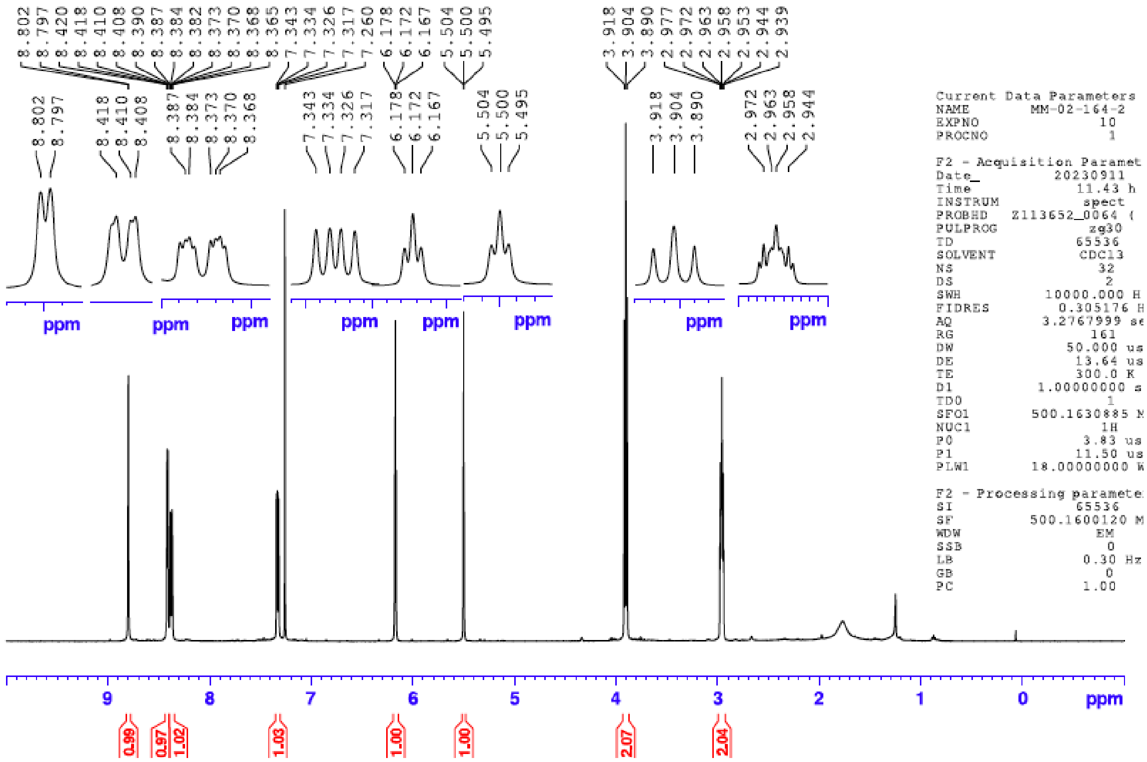

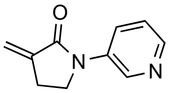

1a

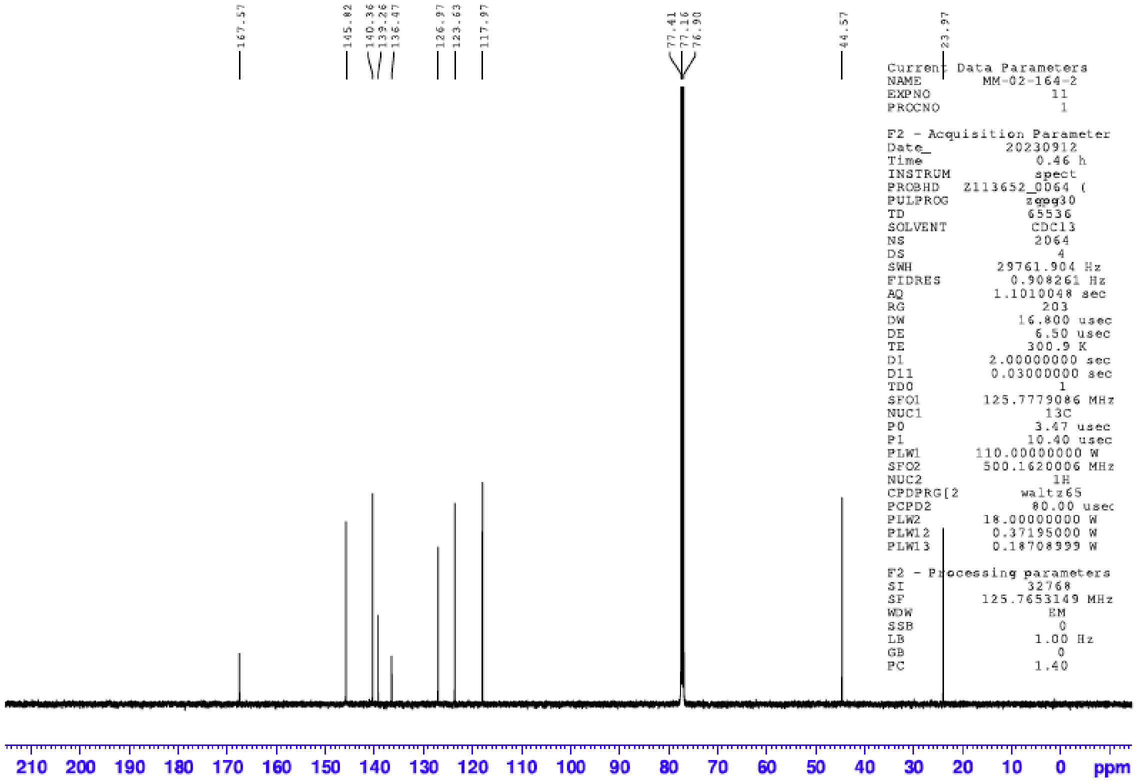

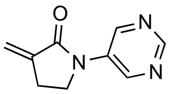

O
N
N
N

1b

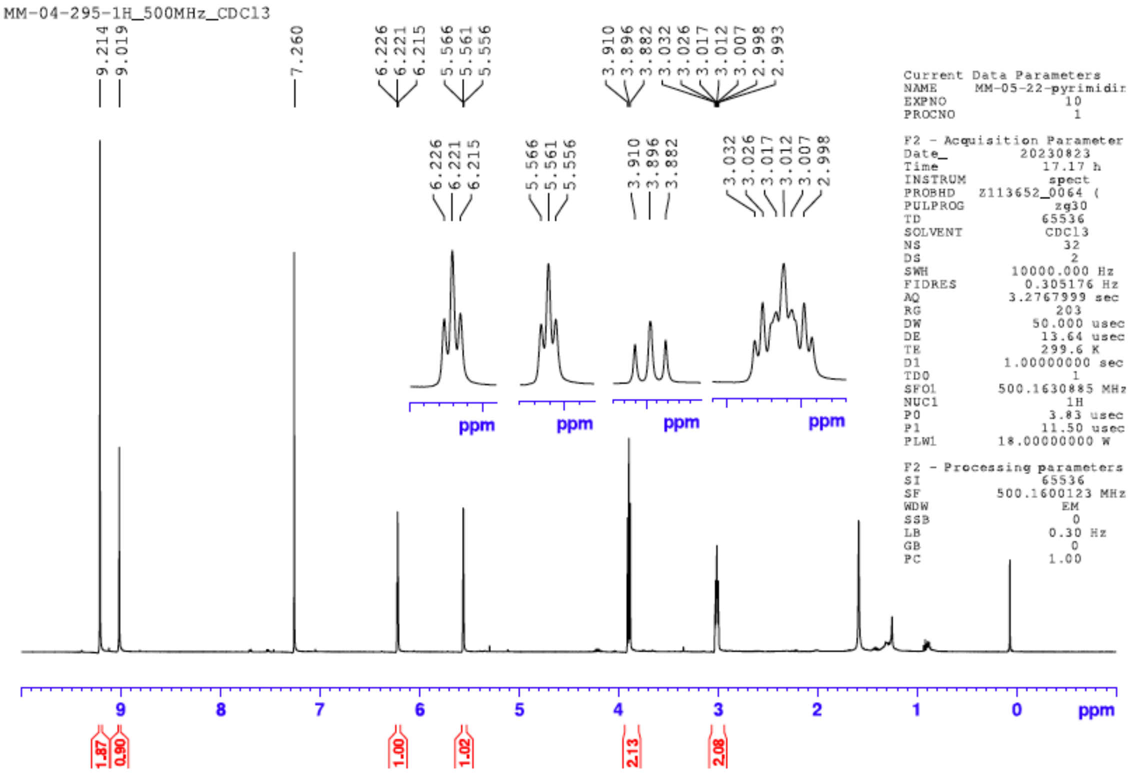

MM-04-295-1H_500MHz_CDCl3
9.214
9.019
7.260
6.226
6.221
6.215
5.566
5.561
5.556
3.910
3.896
3.882
3.032
3.026
3.017
3.012
3.007
2.998
2.993
Current Data Parameters
NAME MM-05-22-pyrimidir
EXPNO 10
PROCNO 1
F2 - Acquisition Parameter
Date_ 20230823
Time 17.17 h
INSTRUM spect
PROBHD Z113652_0064 (
PULPROG zg30
TD 65536
SOLVENT CDCl3
NS 32
DS 2
SWH 10000.000 Hz
FIDRES 0.305176 Hz
AQ 3.2767999 sec
RG 203
DW 50.000 usec
DE 13.64 usec
TE 299.6 K
D1 1.00000000 sec
TD0 1
SFO1 500.1630885 MHz
NUC1 1H
P0 3.83 usec
P1 11.50 usec
PLW1 18.00000000 W
F2 - Processing parameters
SI 65536
SF 500.1600123 MHz
WDW EM
SSB 0
LB 0.30 Hz
GB 0
PC 1.00
ppm
9
8
7
6
5
4
3
2
1
0
1.87
0.90
1.00
1.02
2.13
2.08

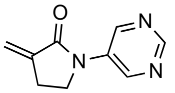

O
N
N
N

1b

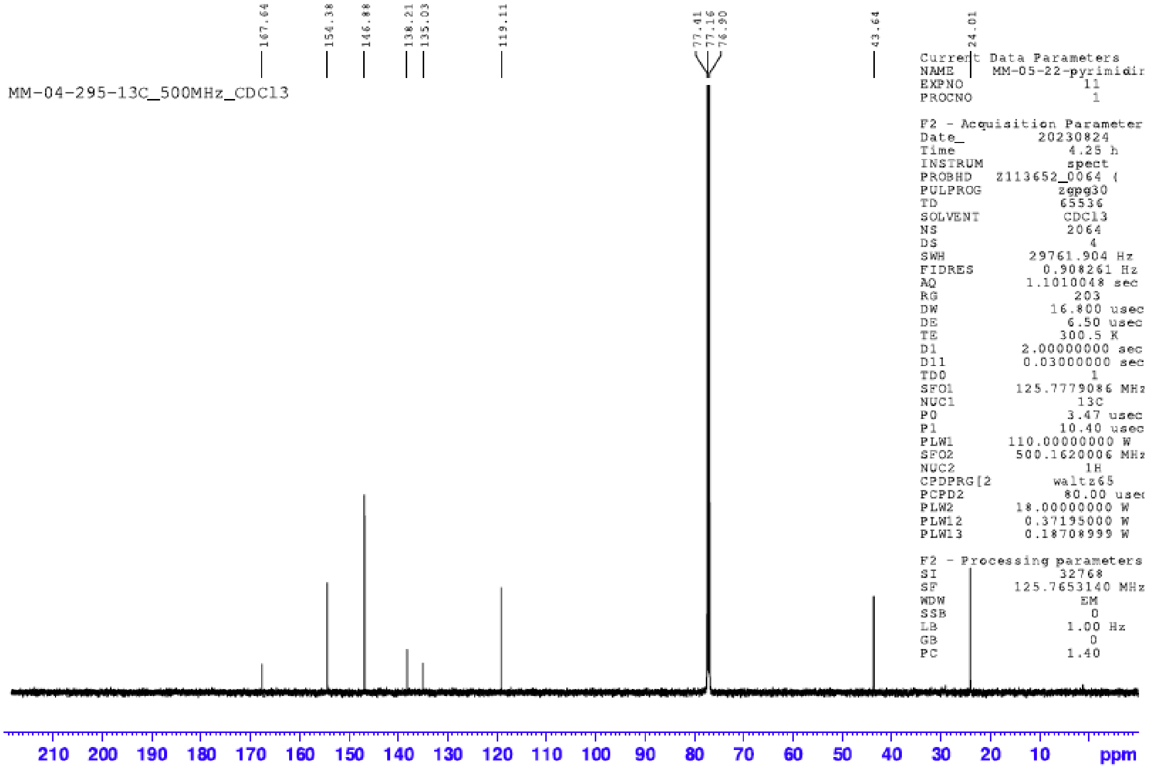

MM-04-295-13C_500MHz_CDCl3
167.64
154.38
146.88
138.21
135.03
119.11
77.41
77.16
76.90
43.64
24.01
Current Data Parameters
NAME MM-05-22-pyrimidir
EXPNO 11
PROCNO 1
F2 - Acquisition Parameter
Date_ 20230824
Time 4.25 h
INSTRUM spect
PROBHD Z113652_0064 (
PULPROG zgpg30
TD 65536
SOLVENT CDCl3
NS 2064
DS 4
SWH 29761.904 Hz
FIDRES 0.908261 Hz
AQ 1.1010048 sec
RG 203
DW 16.800 usec
DE 6.50 usec
TE 300.5 K
D1 2.00000000 sec
D11 0.03000000 sec
TD0 1
SFO1 125.7779086 MHz
NUC1 13C
P0 3.47 usec
P1 10.40 usec
PLW1 110.00000000 W
SFO2 500.1620006 MHz
NUC2 1H
CPDPRG[2 waltz65
PCPD2 80.00 usec
PLW2 18.00000000 W
PLW12 0.37195000 W
PLW13 0.18708999 W
F2 - Processing parameters
SI 32768
SF 125.7653140 MHz
WDW EM
SSB 0
LB 1.00 Hz
GB 0
PC 1.40
210
200
190
180
170
160
150
140
130
120
110
100
90
80
70
60
50
40
30
20
10
ppm

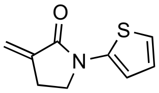

O
N
S

1c

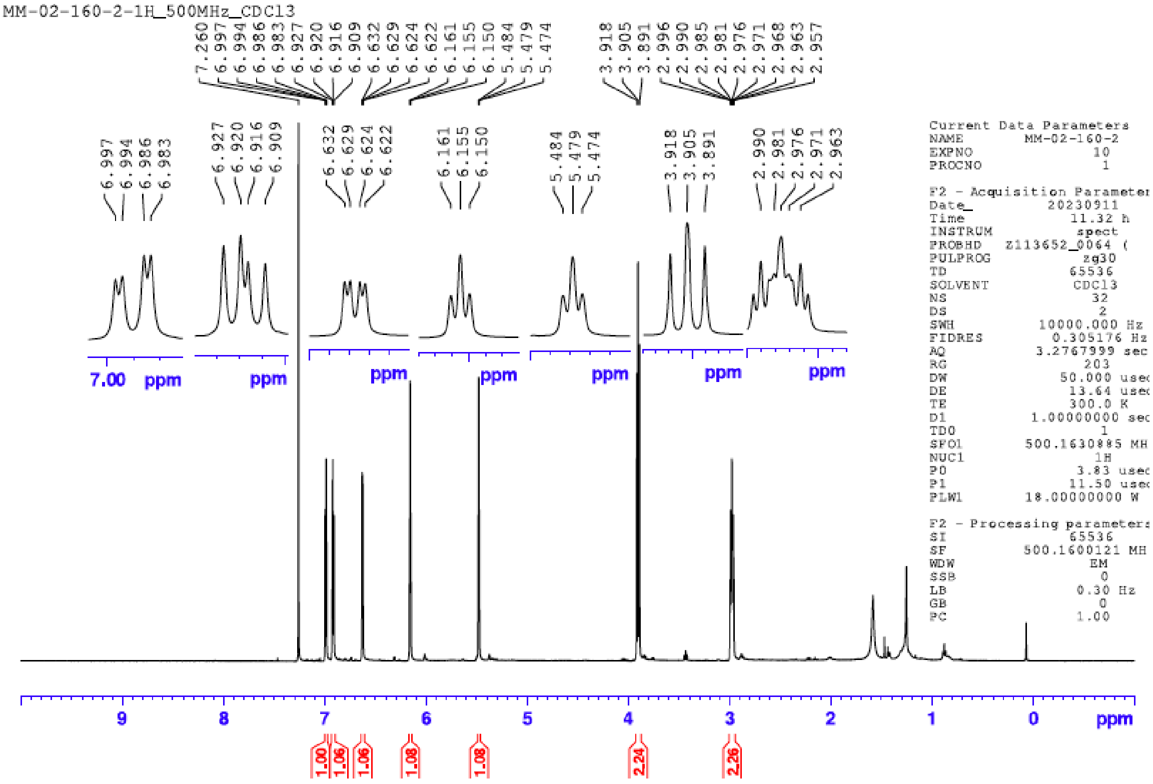

MM-02-160-2-1H_500MHz_CDCl3
7.260 6.997 6.994 6.986 6.983 6.927 6.920 6.916 6.909 6.632 6.629 6.624 6.622 6.161 6.155 6.150 5.484 5.479 5.474
3.918 3.905 3.891 2.996 2.990 2.985 2.981 2.976 2.971 2.968 2.963 2.957
7.00 ppm
ppm
Current Data Parameters
NAME MM-02-160-2
EXPNO 10
PROCNO 1
F2 - Acquisition Parameter
Date_ 20230911
Time 11.32 h
INSTRUM spect
PROBHD Z113652_0064 (
PULPROG zg30
TD 65536
SOLVENT CDCl3
NS 32
DS 2
SWH 10000.000 Hz
FIDRES 0.305176 Hz
AQ 3.2767999 sec
RG 203
DW 50.000 usec
DE 13.64 usec
TE 300.0 K
D1 1.00000000 sec
TD0 1
SFO1 500.1630885 MH
NUC1 1H
P0 3.83 usec
P1 11.50 usec
PLW1 18.00000000 W
F2 - Processing parameters
SI 65536
SF 500.1600121 MH
WDW EM
SSB 0
LB 0.30 Hz
GB 0
PC 1.00
9 8 7 6 5 4 3 2 1 0 ppm
1.00 1.06 1.06 1.08 1.08 2.24 2.26

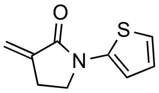

O
N
S

1c

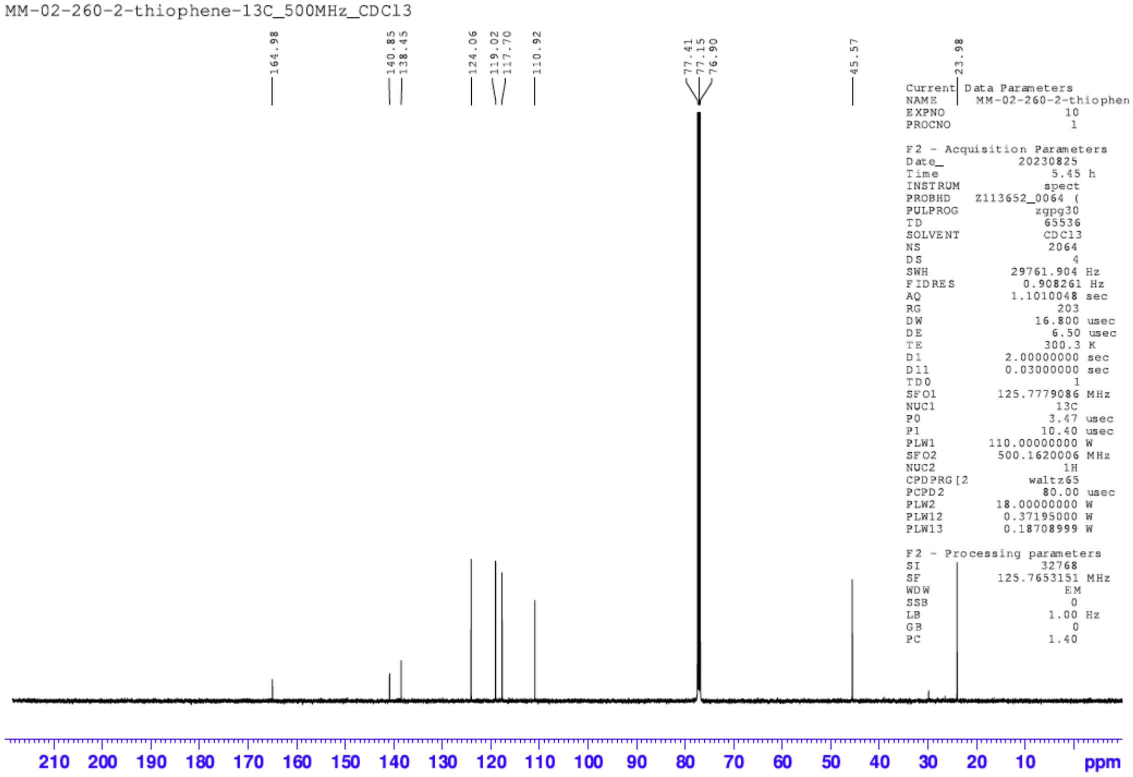

MM-02-260-2-thiophene-13C_500MHz_CDCl3
164.98
140.85 138.45
124.06 119.02 117.70
110.92
77.41 77.15 76.90
45.57
23.98
Current Data Parameters
NAME MM-02-260-2-thiophen
EXPNO 10
PROCNO 1
F2 - Acquisition Parameters
Date_ 20230825
Time 5.45 h
INSTRUM spect
PROBHD Z113652_0064 (
PULPROG zgpg30
TD 65536
SOLVENT CDCl3
NS 2064
DS 4
SWH 29761.904 Hz
FIDRES 0.908261 Hz
AQ 1.1010048 sec
RG 203
DW 16.800 usec
DE 6.50 usec
TE 300.3 K
D1 2.00000000 sec
D11 0.03000000 sec
TD0 1
SFO1 125.7779086 MHz
NUC1 13C
P0 3.47 usec
P1 10.40 usec
PLW1 110.00000000 W
SFO2 500.1620006 MHz
NUC2 1H
CPDPRG[2 waltz65
PCPD2 80.00 usec
PLW2 18.00000000 W
PLW12 0.37195000 W
PLW13 0.18708999 W
F2 - Processing parameters
SI 32768
SF 125.7653151 MHz
WDW EM
SSB 0
LB 1.00 Hz
GB 0
PC 1.40
210 200 190 180 170 160 150 140 130 120 110 100 90 80 70 60 50 40 30 20 10 ppm

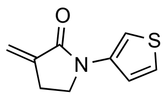
O
N
S

1d

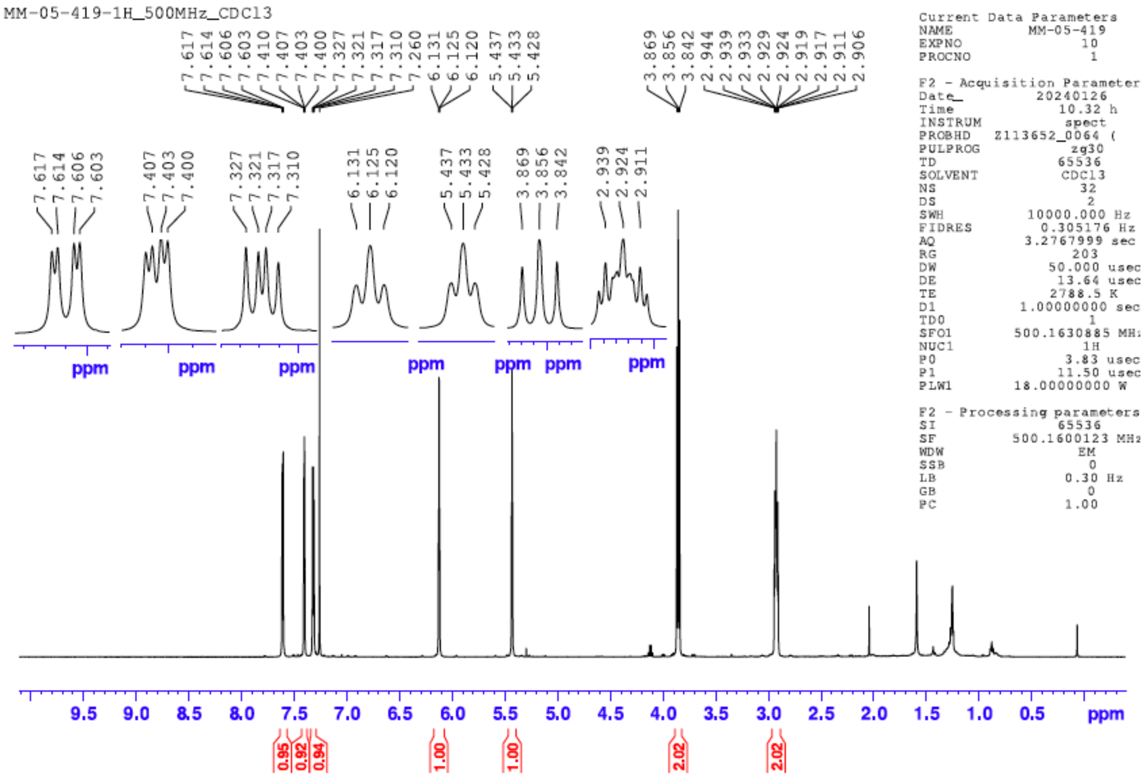
MM-05-419-1H_500MHz_CDCl3
7.617
7.614
7.606
7.603
7.410
7.407
7.403
7.400
7.327
7.321
7.317
7.310
7.260
6.131
6.125
6.120
5.437
5.433
5.428
3.869
3.856
3.842
2.944
2.939
2.933
2.929
2.924
2.919
2.917
2.911
2.906
Current Data Parameters
NAME MM-05-419
EXPNO 10
PROCNO 1
F2 - Acquisition Parameter
Date_ 20240126
Time 10.32 h
INSTRUM spect
PROBHD Z113652_0064 (
PULPROG zg30
TD 65536
SOLVENT CDCl3
NS 32
DS 2
SWH 10000.000 Hz
FIDRES 0.305176 Hz
AQ 3.2767999 sec
RG 203
DW 50.000 usec
DE 13.64 usec
TE 2788.5 K
D1 1.00000000 sec
TD0 1
SFO1 500.1630885 MH:
NUC1 1H
P0 3.83 usec
P1 11.50 usec
PLW1 18.00000000 W
F2 - Processing parameters
SI 65536
SF 500.1600123 MHz
WDW EM
SSB 0
LB 0.30 Hz
GB 0
PC 1.00
ppm
9.5 9.0 8.5 8.0 7.5 7.0 6.5 6.0 5.5 5.0 4.5 4.0 3.5 3.0 2.5 2.0 1.5 1.0 0.5 ppm
0.95
0.92
0.94
1.00
1.00
2.02
2.02

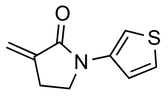
O
N
S

1d

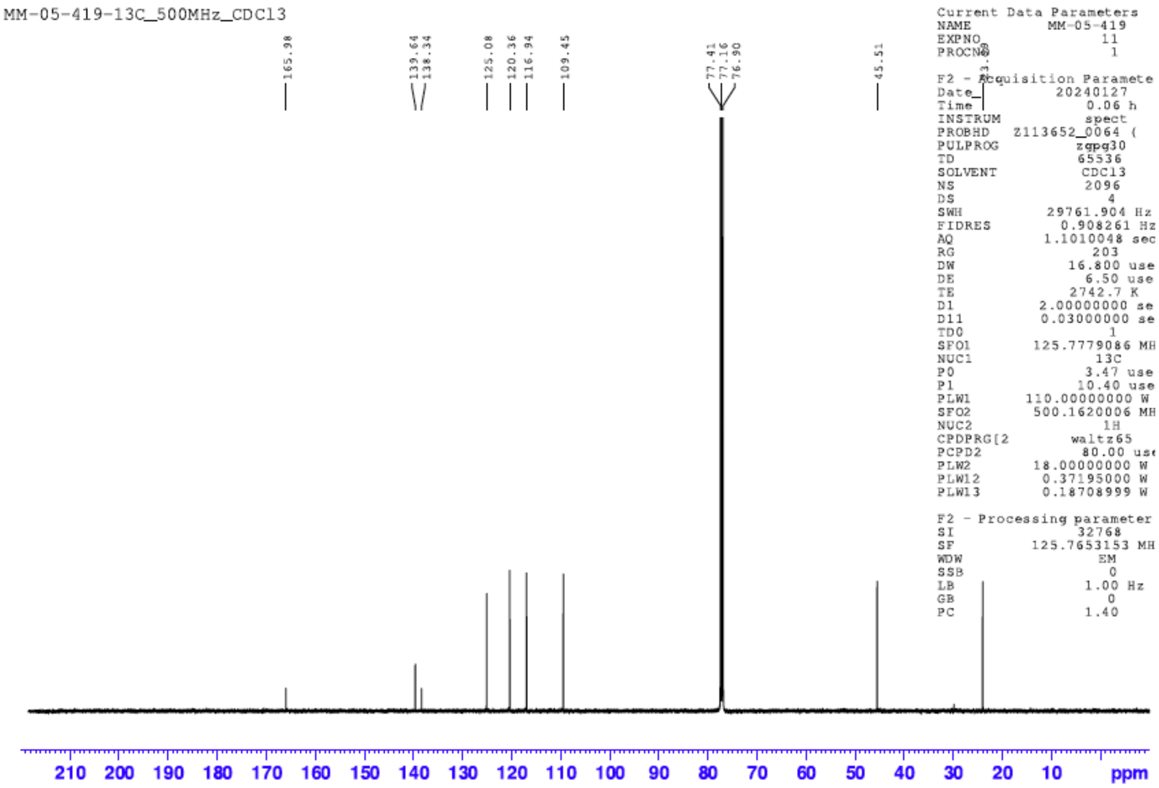
MM-05-419-13C_500MHz_CDCl3
165.98
139.64
138.34
125.08
120.36
116.94
109.45
77.41
77.16
76.90
45.51
Current Data Parameters
NAME MM-05-419
EXPNO 11
PROCNO 1
F2 - Acquisition Paramete
Date_ 20240127
Time 0.06 h
INSTRUM spect
PROBHD Z113652_0064 (
PULPROG zgpg30
TD 65536
SOLVENT CDCl3
NS 2096
DS 4
SWH 29761.904 Hz
FIDRES 0.908261 Hz
AQ 1.1010048 sec
RG 203
DW 16.800 use
DE 6.50 use
TE 2742.7 K
D1 2.00000000 se
D11 0.03000000 se
TD0 1
SFO1 125.7779086 MH
NUC1 13C
P0 3.47 use
P1 10.40 use
PLW1 110.00000000 W
SFO2 500.1620006 MH
NUC2 1H
CPDPRG[2 waltz65
PCPD2 80.00 use
PLW2 18.00000000 W
PLW12 0.37195000 W
PLW13 0.18708999 W
F2 - Processing parameter
SI 32768
SF 125.7653153 MH
WDW EM
SSB 0
LB 1.00 Hz
GB 0
PC 1.40
210 200 190 180 170 160 150 140 130 120 110 100 90 80 70 60 50 40 30 20 10 ppm

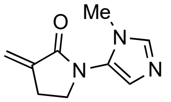
O
Me
N
N
N

1e

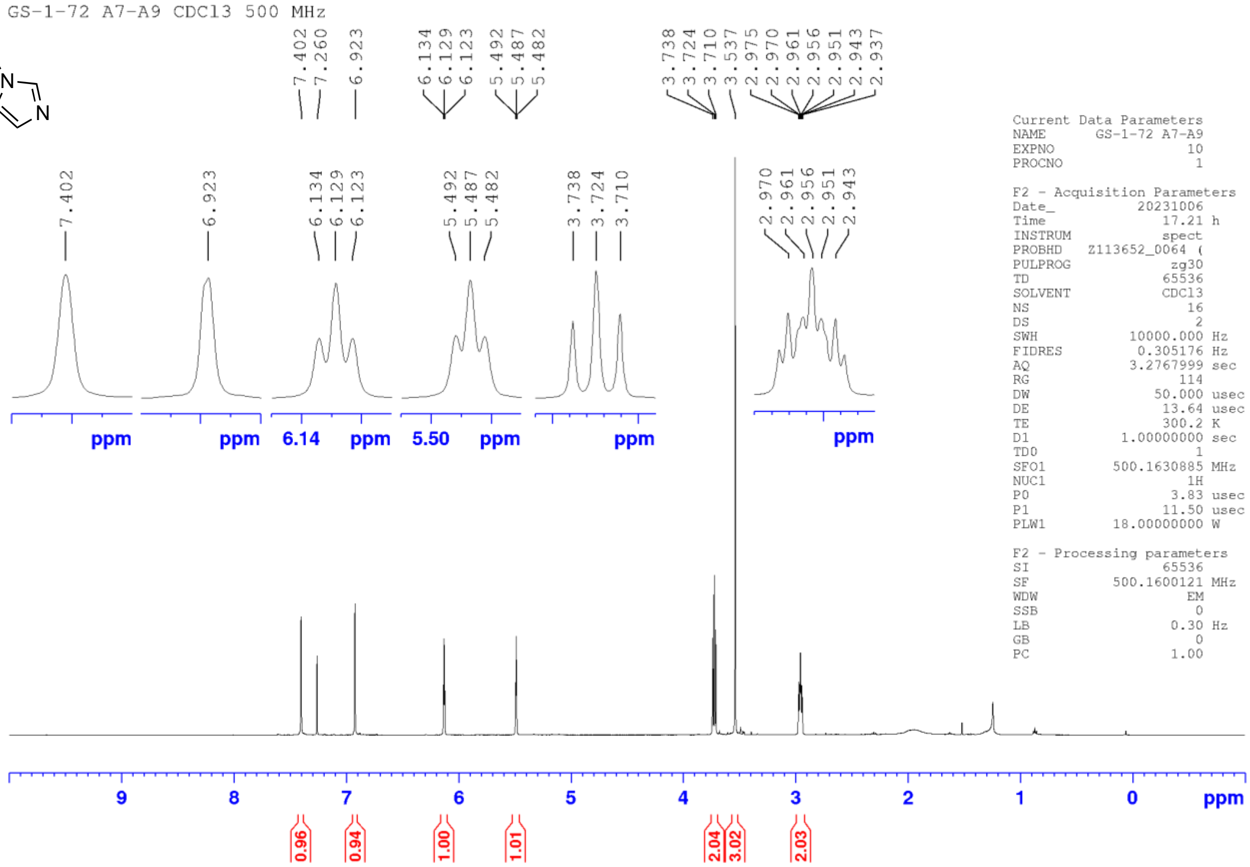
GS-1-72 A7-A9 CDCl3 500 MHz
7.402
7.260
6.923
6.134
6.129
6.123
5.492
5.487
5.482
3.738
3.724
3.710
3.537
2.975
2.970
2.961
2.956
2.951
2.943
2.937
ppm
6.14
5.50
Current Data Parameters
NAME GS-1-72 A7-A9
EXPNO 10
PROCNO 1
F2 - Acquisition Parameters
Date_ 20231006
Time 17.21 h
INSTRUM spect
PROBHD Z113652_0064 (
PULPROG zg30
TD 65536
SOLVENT CDCl3
NS 16
DS 2
SWH 10000.000 Hz
FIDRES 0.305176 Hz
AQ 3.2767999 sec
RG 114
DW 50.000 usec
DE 13.64 usec
TE 300.2 K
D1 1.00000000 sec
TD0 1
SFO1 500.1630885 MHz
NUC1 1H
P0 3.83 usec
P1 11.50 usec
PLW1 18.00000000 W
F2 - Processing parameters
SI 65536
SF 500.1600121 MHz
WDW EM
SSB 0
LB 0.30 Hz
GB 0
PC 1.00
9 8 7 6 5 4 3 2 1 0 ppm
0.96
0.94
1.00
1.01
2.04
3.02
2.03

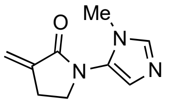
O
Me
N
N
N

1e

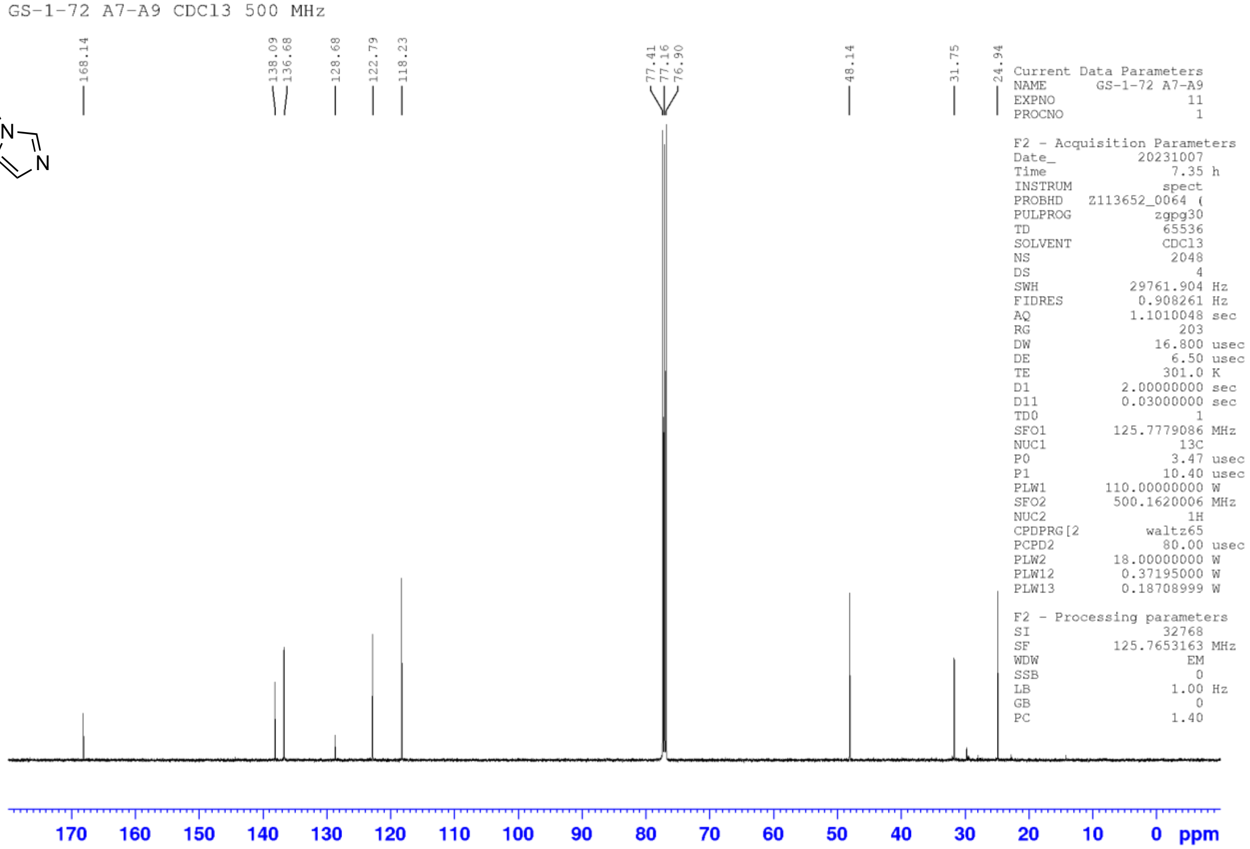
GS-1-72 A7-A9 CDCl3 500 MHz
168.14
138.09
136.68
128.68
122.79
118.23
77.41
77.16
76.90
48.14
31.75
24.94
Current Data Parameters
NAME GS-1-72 A7-A9
EXPNO 11
PROCNO 1
F2 - Acquisition Parameters
Date_ 20231007
Time 7.35 h
INSTRUM spect
PROBHD Z113652_0064 (
PULPROG zgpg30
TD 65536
SOLVENT CDCl3
NS 2048
DS 4
SWH 29761.904 Hz
FIDRES 0.908261 Hz
AQ 1.1010048 sec
RG 203
DW 16.800 usec
DE 6.50 usec
TE 301.0 K
D1 2.00000000 sec
D11 0.03000000 sec
TD0 1
SFO1 125.7779086 MHz
NUC1 13C
P0 3.47 usec
P1 10.40 usec
PLW1 110.00000000 W
SFO2 500.1620006 MHz
NUC2 1H
CPDPRG[2 waltz65
PCPD2 80.00 usec
PLW2 18.00000000 W
PLW12 0.37195000 W
PLW13 0.18708999 W
F2 - Processing parameters
SI 32768
SF 125.7653163 MHz
WDW EM
SSB 0
LB 1.00 Hz
GB 0
PC 1.40
170 160 150 140 130 120 110 100 90 80 70 60 50 40 30 20 10 0 ppm

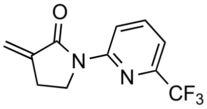
O
N
N
CF3

1f

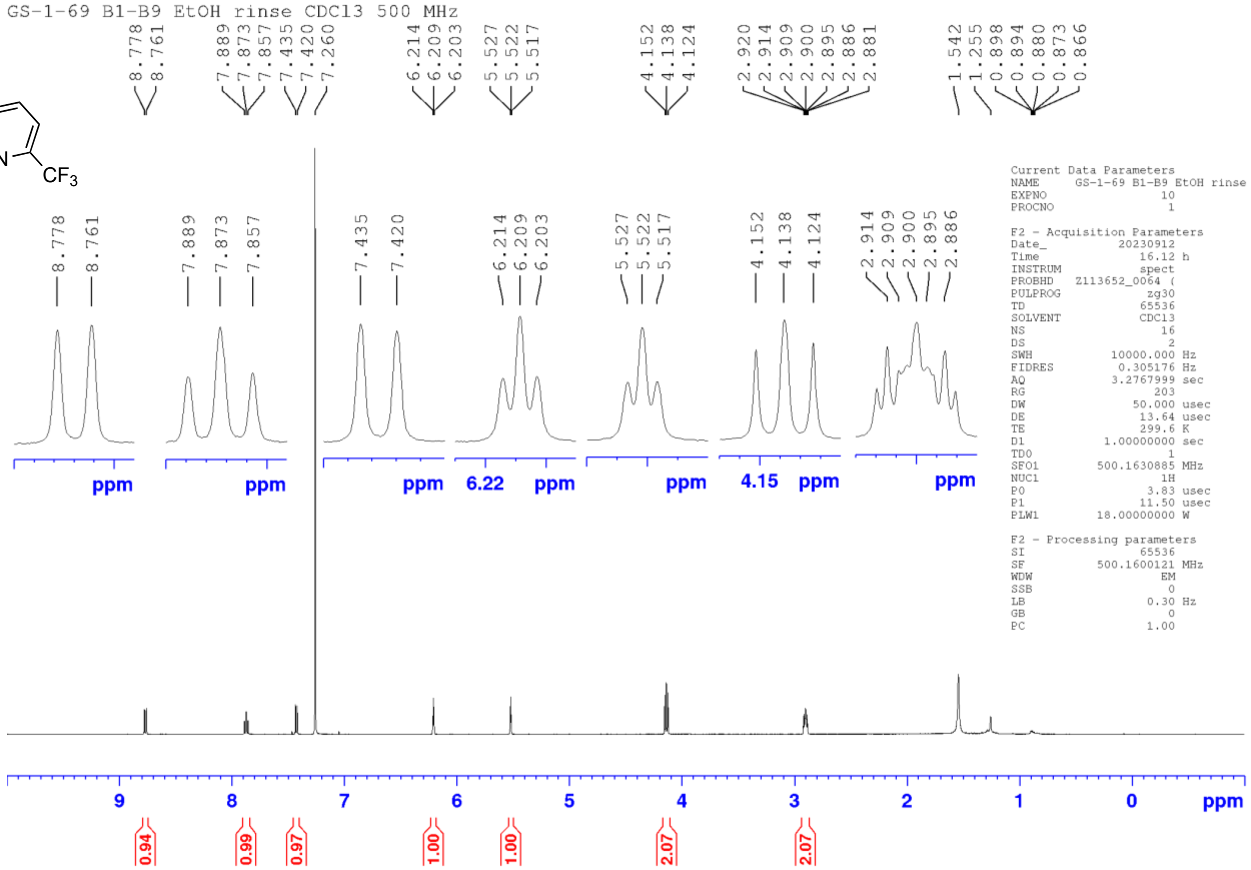
GS-1-69 B1-B9 EtOH rinse CDCl3 500 MHz
8.778
8.761
7.889
7.873
7.857
7.435
7.420
7.260
6.214
6.209
6.203
5.527
5.522
5.517
4.152
4.138
4.124
2.920
2.914
2.909
2.900
2.895
2.886
2.881
1.542
1.255
0.898
0.894
0.880
0.873
0.866
6.22
4.15
ppm
0.94
0.99
0.97
1.00
1.00
2.07
2.07
Current Data Parameters
NAME GS-1-69 B1-B9 EtOH rinse
EXPNO 10
PROCNO 1
F2 - Acquisition Parameters
Date_ 20230912
Time 16.12 h
INSTRUM spect
PROBHD Z113652_0064 (
PULPROG zg30
TD 65536
SOLVENT CDCl3
NS 16
DS 2
SWH 10000.000 Hz
FIDRES 0.305176 Hz
AQ 3.2767999 sec
RG 203
DW 50.000 usec
DE 13.64 usec
TE 299.6 K
D1 1.00000000 sec
TD0 1
SFO1 500.1630885 MHz
NUC1 1H
P0 3.83 usec
P1 11.50 usec
PLW1 18.00000000 W
F2 - Processing parameters
SI 65536
SF 500.1600121 MHz
WDW EM
SSB 0
LB 0.30 Hz
GB 0
PC 1.00

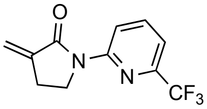
O
N
N
CF3

1f

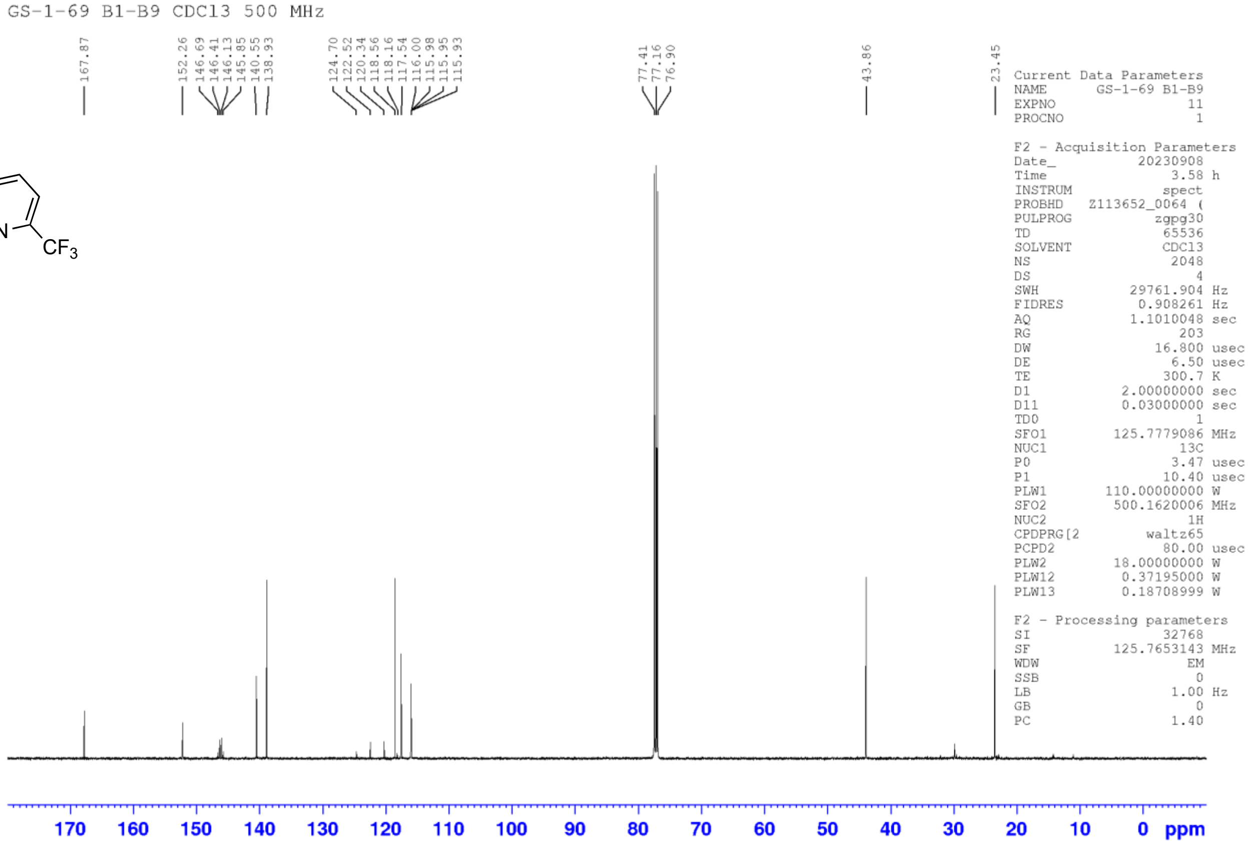
GS-1-69 B1-B9 CDCl3 500 MHz
167.87
152.26
146.69
146.41
146.13
145.85
140.55
138.93
124.70
122.52
120.34
118.56
118.16
117.54
116.00
115.98
115.95
115.93
77.41
77.16
76.90
43.86
23.45
ppm
Current Data Parameters
NAME GS-1-69 B1-B9
EXPNO 11
PROCNO 1
F2 - Acquisition Parameters
Date_ 20230908
Time 3.58 h
INSTRUM spect
PROBHD Z113652_0064 (
PULPROG zgpg30
TD 65536
SOLVENT CDCl3
NS 2048
DS 4
SWH 29761.904 Hz
FIDRES 0.908261 Hz
AQ 1.1010048 sec
RG 203
DW 16.800 usec
DE 6.50 usec
TE 300.7 K
D1 2.00000000 sec
D11 0.03000000 sec
TD0 1
SFO1 125.7779086 MHz
NUC1 13C
P0 3.47 usec
P1 10.40 usec
PLW1 110.00000000 W
SFO2 500.1620006 MHz
NUC2 1H
CPDPRG[2 waltz65
PCPD2 80.00 usec
PLW2 18.00000000 W
PLW12 0.37195000 W
PLW13 0.18708999 W
F2 - Processing parameters
SI 32768
SF 125.7653143 MHz
WDW EM
SSB 0
LB 1.00 Hz
GB 0
PC 1.40

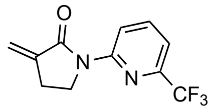

1f

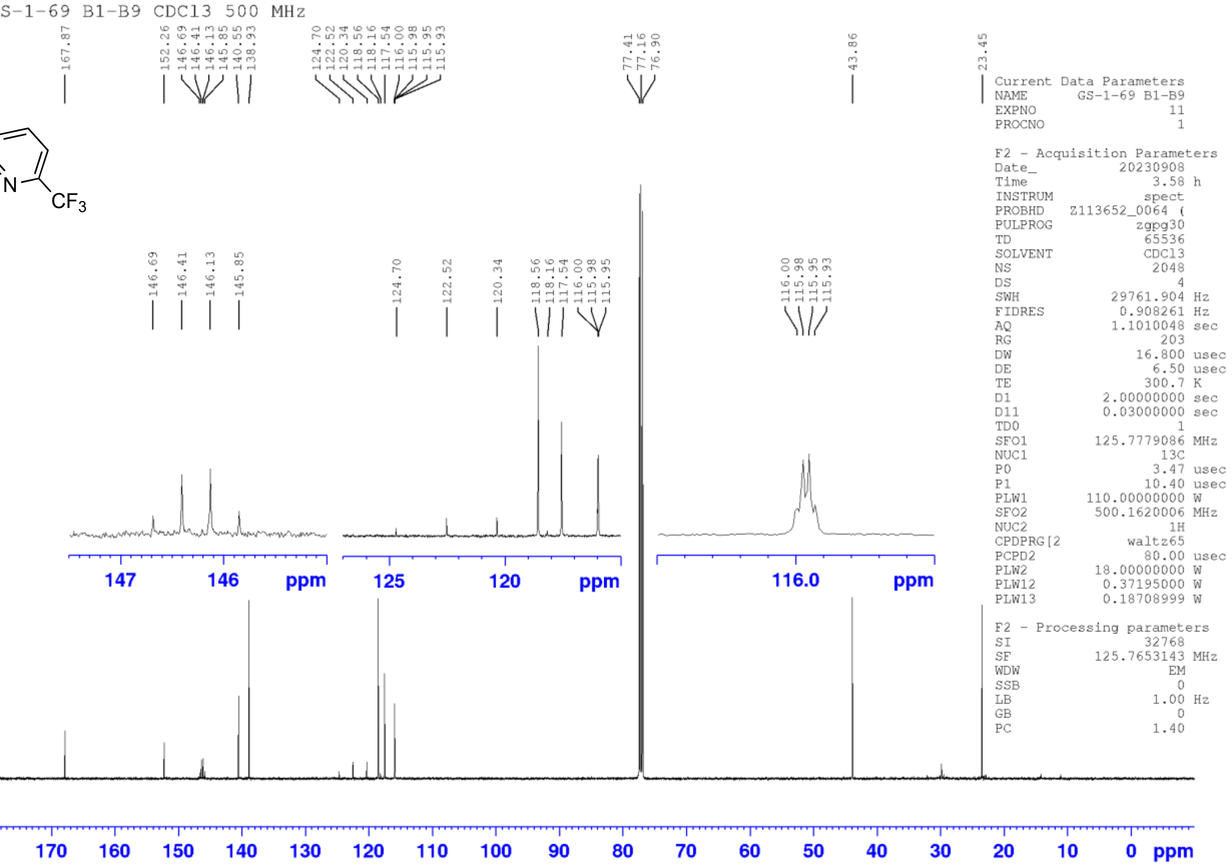

GS-1-69 B1-B9 CDCl3 500 MHz
167.87
152.26 146.69 146.41 146.13 145.85 140.55 138.93
124.70 122.52 120.34 118.56 118.16 117.54 116.00 115.98 115.95 115.93
77.41 77.16 76.90
43.86
23.45
146.69 146.41 146.13 145.85
124.70 122.52 120.34 118.56 118.16 117.54 116.00 115.98 115.95
116.00 115.98 115.95 115.93
147 146 ppm
125 120 ppm
116.0 ppm
170 160 150 140 130 120 110 100 90 80 70 60 50 40 30 20 10 0 ppm
Current Data Parameters
NAME GS-1-69 B1-B9
EXPNO 11
PROCNO 1
F2 - Acquisition Parameters
Date_ 20230908
Time 3.58 h
INSTRUM spect
PROBHD Z113652_0064 (
PULPROG zgpg30
TD 65536
SOLVENT CDCl3
NS 2048
DS 4
SWH 29761.904 Hz
FIDRES 0.908261 Hz
AQ 1.1010048 sec
RG 203
DW 16.800 usec
DE 6.50 usec
TE 300.7 K
D1 2.00000000 sec
D11 0.03000000 sec
TD0 1
SFO1 125.7779086 MHz
NUC1 13C
P0 3.47 usec
P1 10.40 usec
PLW1 110.00000000 W
SFO2 500.1620006 MHz
NUC2 1H
CPDPRG[2 waltz65
PCPD2 80.00 usec
PLW2 18.00000000 W
PLW12 0.37195000 W
PLW13 0.18708999 W
F2 - Processing parameters
SI 32768
SF 125.7653143 MHz
WDW EM
SSB 0
LB 1.00 Hz
GB 0
PC 1.40

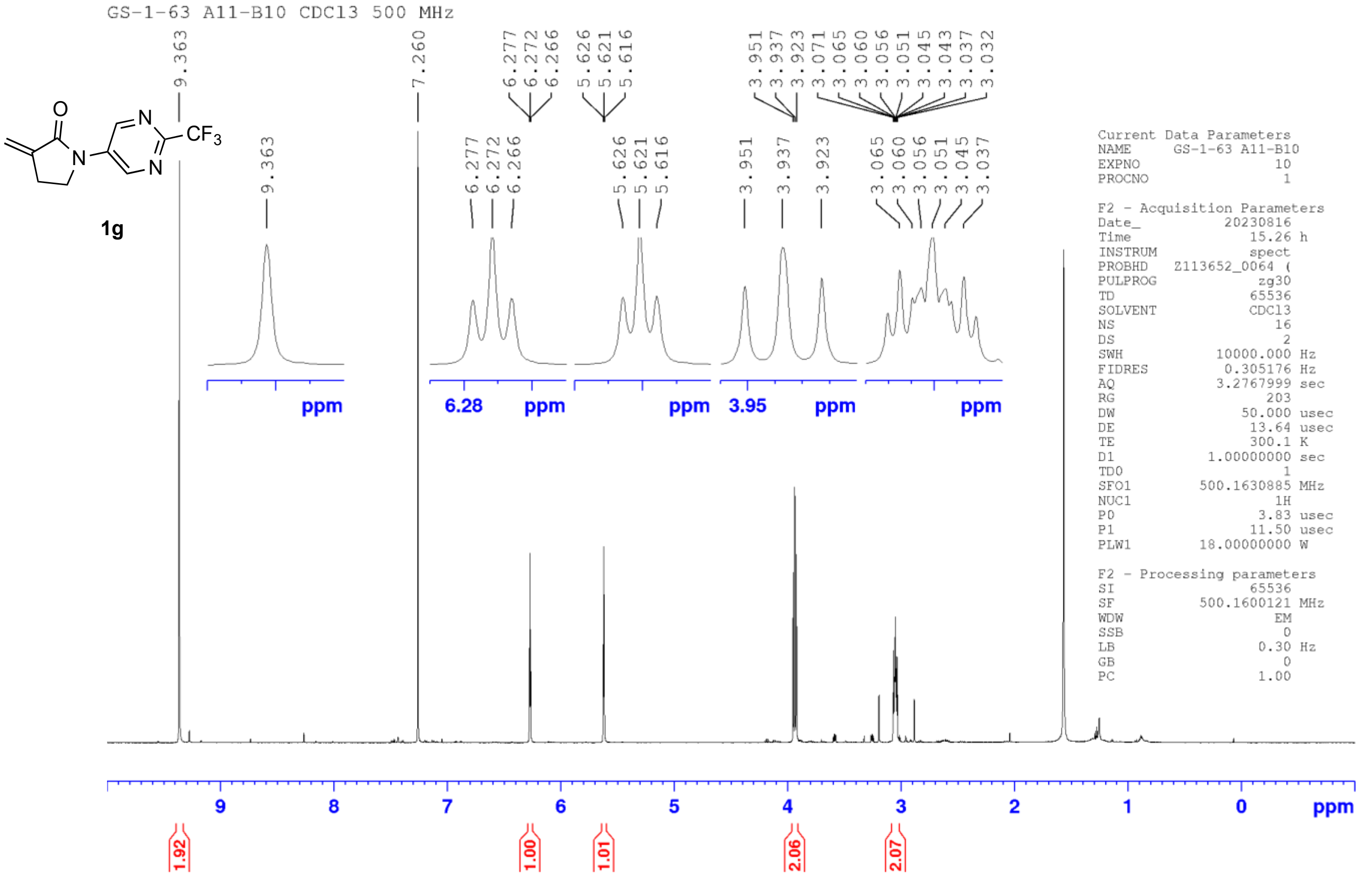
GS-1-63 A11-B10 CDCl3 500 MHz
9.363
7.260
6.277
6.272
6.266
5.626
5.621
5.616
3.951
3.937
3.923
3.071
3.065
3.060
3.056
3.051
3.045
3.043
3.037
3.032
CF3
1g
ppm
6.28
3.95
Current Data Parameters
NAME GS-1-63 A11-B10
EXPNO 10
PROCNO 1
F2 - Acquisition Parameters
Date_ 20230816
Time 15.26 h
INSTRUM spect
PROBHD Z113652_0064 (
PULPROG zg30
TD 65536
SOLVENT CDCl3
NS 16
DS 2
SWH 10000.000 Hz
FIDRES 0.305176 Hz
AQ 3.2767999 sec
RG 203
DW 50.000 usec
DE 13.64 usec
TE 300.1 K
D1 1.00000000 sec
TD0 1
SFO1 500.1630885 MHz
NUC1 1H
P0 3.83 usec
P1 11.50 usec
PLW1 18.00000000 W
F2 - Processing parameters
SI 65536
SF 500.1600121 MHz
WDW EM
SSB 0
LB 0.30 Hz
GB 0
PC 1.00
1.92
1.00
1.01
2.06
2.07

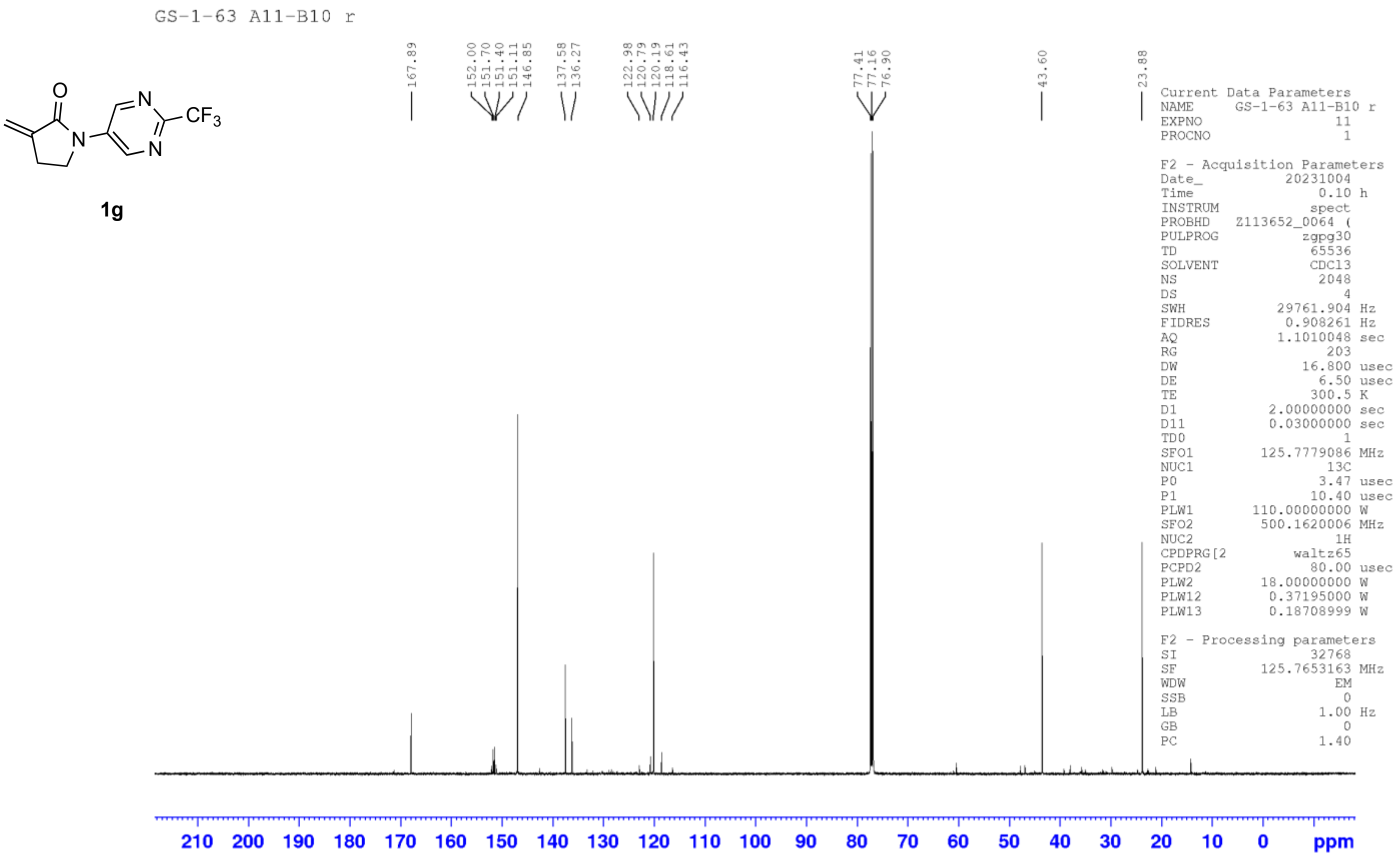
GS-1-63 A11-B10 r
167.89
152.00
151.70
151.40
151.11
146.85
137.58
136.27
122.98
120.79
120.19
118.61
116.43
77.41
77.16
76.90
43.60
23.88
CF3
1g
ppm
Current Data Parameters
NAME GS-1-63 A11-B10 r
EXPNO 11
PROCNO 1
F2 - Acquisition Parameters
Date_ 20231004
Time 0.10 h
INSTRUM spect
PROBHD Z113652_0064 (
PULPROG zgpg30
TD 65536
SOLVENT CDCl3
NS 2048
DS 4
SWH 29761.904 Hz
FIDRES 0.908261 Hz
AQ 1.1010048 sec
RG 203
DW 16.800 usec
DE 6.50 usec
TE 300.5 K
D1 2.00000000 sec
D11 0.03000000 sec
TD0 1
SFO1 125.7779086 MHz
NUC1 13C
P0 3.47 usec
P1 10.40 usec
PLW1 110.00000000 W
SFO2 500.1620006 MHz
NUC2 1H
CPDPRG[2 waltz65
PCPD2 80.00 usec
PLW2 18.00000000 W
PLW12 0.37195000 W
PLW13 0.18708999 W
F2 - Processing parameters
SI 32768
SF 125.7653163 MHz
WDW EM
SSB 0
LB 1.00 Hz
GB 0
PC 1.40

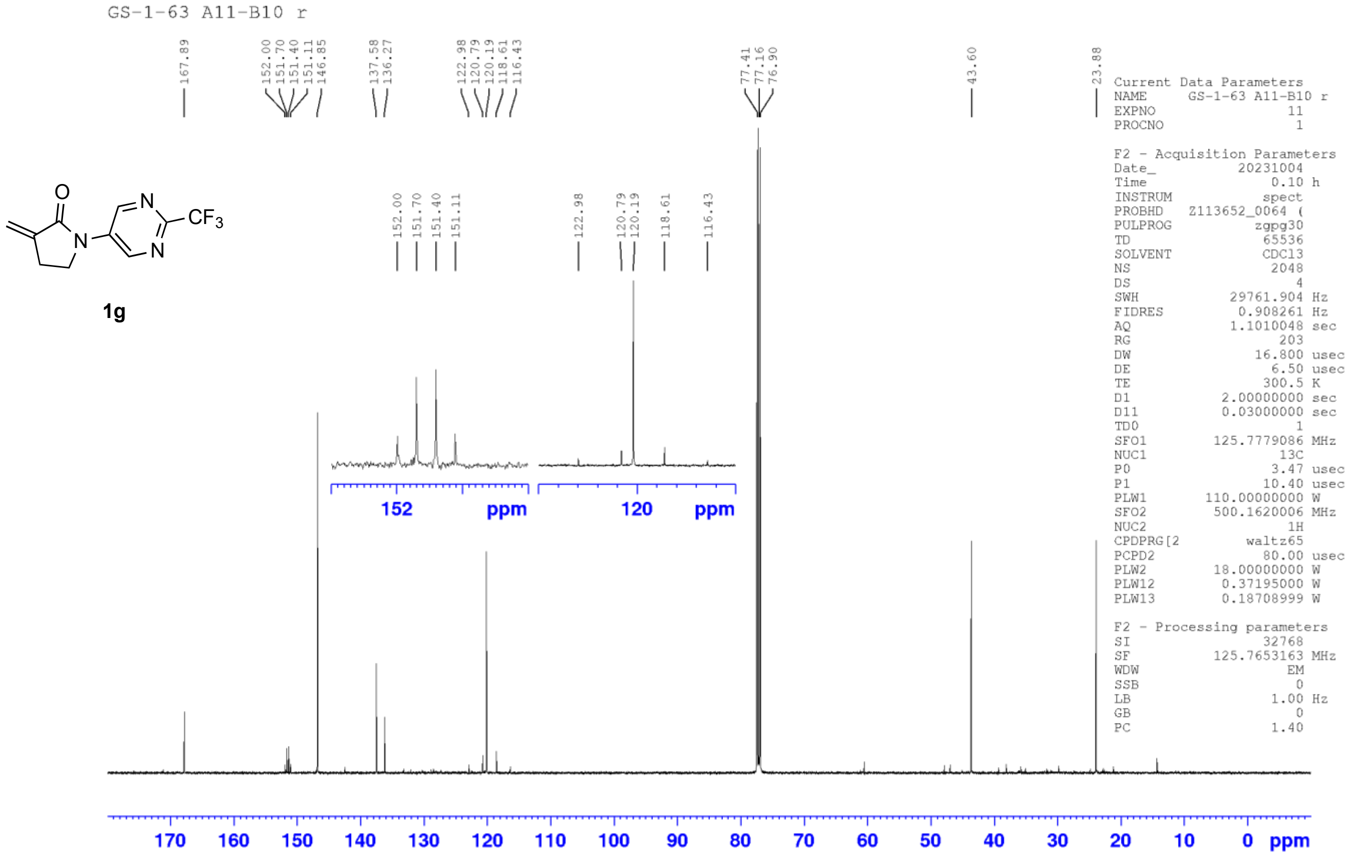

1g

GS-1-63 A11-B10 r
167.89
152.00
151.70
151.40
151.11
146.85
137.58
136.27
122.98
120.79
120.19
118.61
116.43
77.41
77.16
76.90
43.60
23.88
152 ppm
120 ppm
170 160 150 140 130 120 110 100 90 80 70 60 50 40 30 20 10 0 ppm
Current Data Parameters
NAME GS-1-63 A11-B10 r
EXPNO 11
PROCNO 1
F2 - Acquisition Parameters
Date_ 20231004
Time 0.10 h
INSTRUM spect
PROBHD Z113652_0064 (
PULPROG zgpg30
TD 65536
SOLVENT CDCl3
NS 2048
DS 4
SWH 29761.904 Hz
FIDRES 0.908261 Hz
AQ 1.1010048 sec
RG 203
DW 16.800 usec
DE 6.50 usec
TE 300.5 K
D1 2.00000000 sec
D11 0.03000000 sec
TD0 1
SFO1 125.7779086 MHz
NUC1 13C
P0 3.47 usec
P1 10.40 usec
PLW1 110.00000000 W
SFO2 500.1620006 MHz
NUC2 1H
CPDPRG[2 waltz65
PCPD2 80.00 usec
PLW2 18.00000000 W
PLW12 0.37195000 W
PLW13 0.18708999 W
F2 - Processing parameters
SI 32768
SF 125.7653163 MHz
WDW EM
SSB 0
LB 1.00 Hz
GB 0
PC 1.40

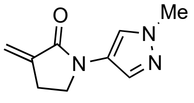

1h

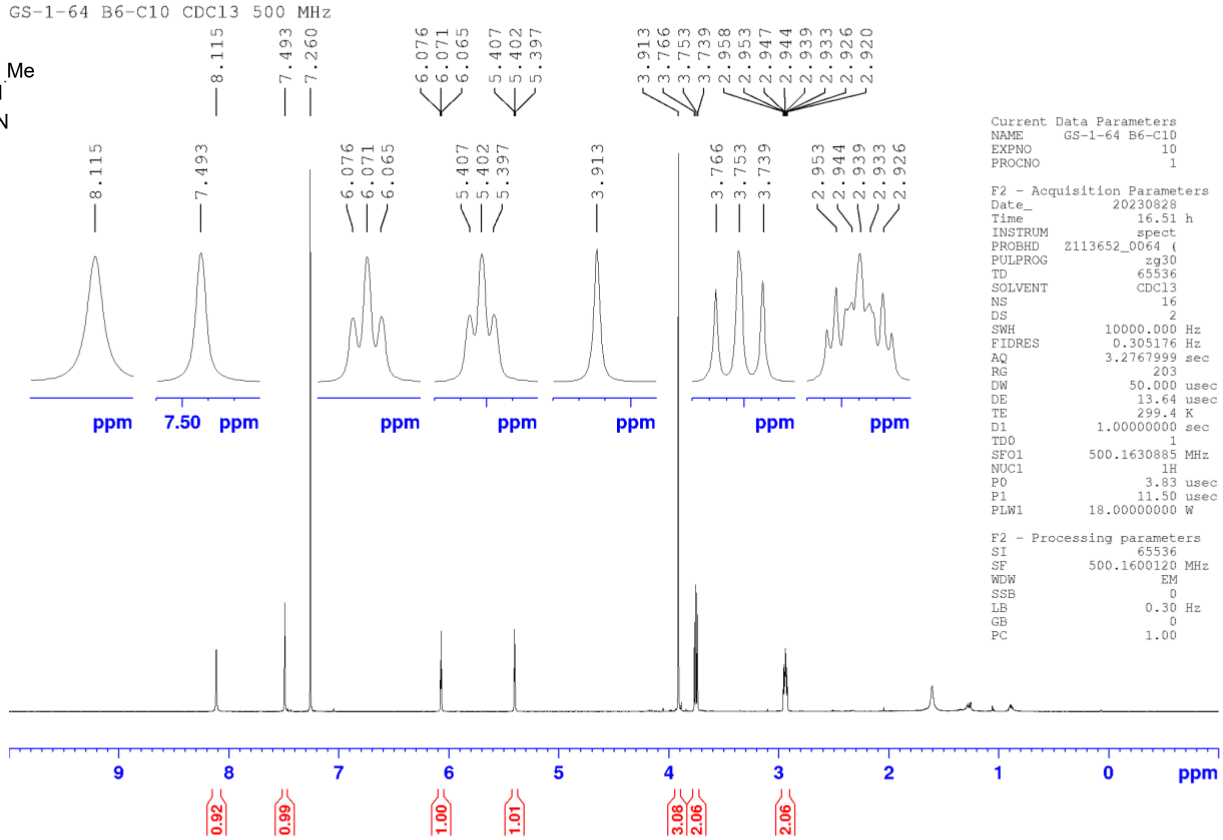
GS-1-64 B6-C10 CDCl3 500 MHz
8.115
7.493
7.260
6.076
6.071
6.065
5.407
5.402
5.397
3.913
3.766
3.753
3.739
2.958
2.953
2.947
2.944
2.939
2.933
2.926
2.920
7.50
ppm
0.92
0.99
1.00
1.01
3.08
2.06
2.06
Current Data Parameters
NAME GS-1-64 B6-C10
EXPNO 10
PROCNO 1
F2 - Acquisition Parameters
Date_ 20230828
Time 16.51 h
INSTRUM spect
PROBHD Z113652_0064 (
PULPROG zg30
TD 65536
SOLVENT CDCl3
NS 16
DS 2
SWH 10000.000 Hz
FIDRES 0.305176 Hz
AQ 3.2767999 sec
RG 203
DW 50.000 usec
DE 13.64 usec
TE 299.4 K
D1 1.00000000 sec
TD0 1
SFO1 500.1630885 MHz
NUC1 1H
P0 3.83 usec
P1 11.50 usec
PLW1 18.00000000 W
F2 - Processing parameters
SI 65536
SF 500.1600120 MHz
WDW EM
SSB 0
LB 0.30 Hz
GB 0
PC 1.00

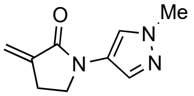

1h

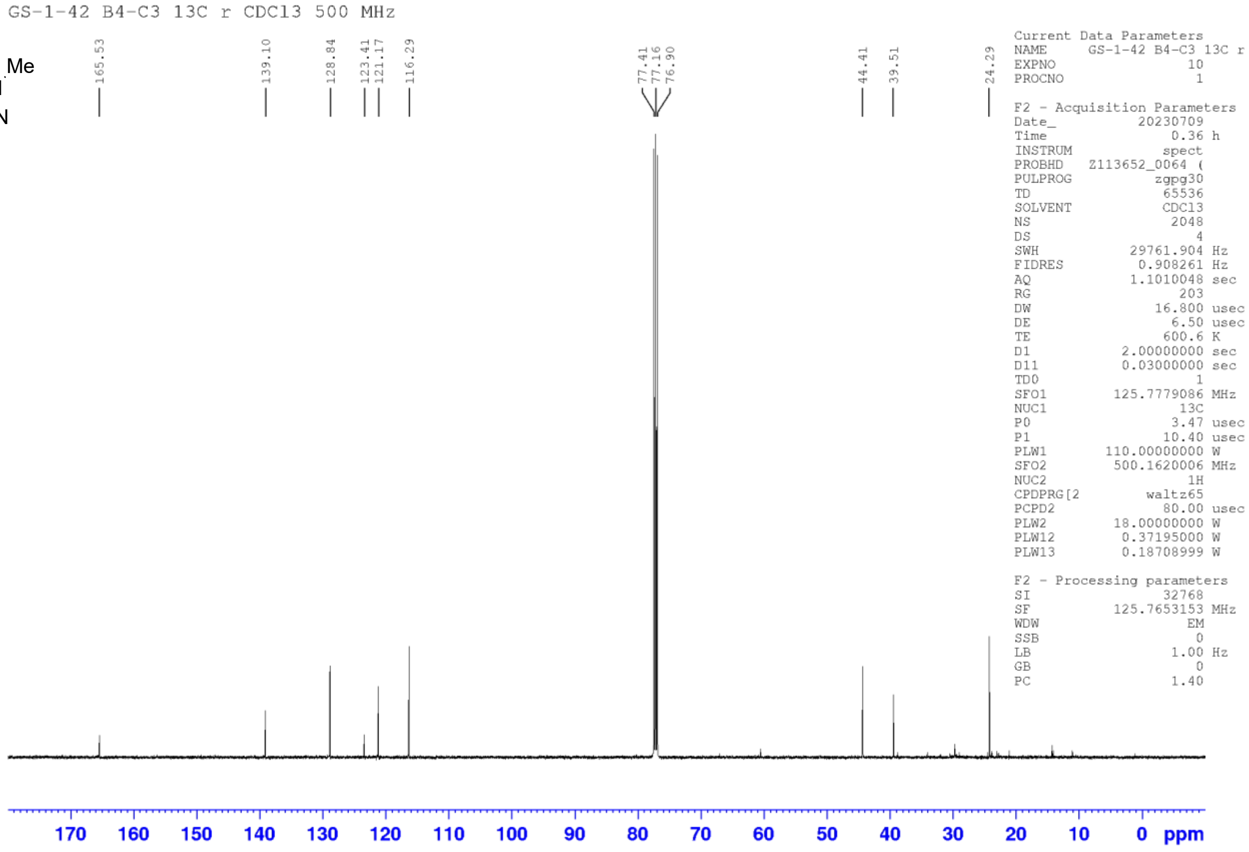
GS-1-42 B4-C3 13C r CDCl3 500 MHz
165.53
139.10
128.84
123.41
121.17
116.29
77.41
77.16
76.90
44.41
39.51
24.29
ppm
Current Data Parameters
NAME GS-1-42 B4-C3 13C r
EXPNO 10
PROCNO 1
F2 - Acquisition Parameters
Date_ 20230709
Time 0.36 h
INSTRUM spect
PROBHD Z113652_0064 (
PULPROG zgpg30
TD 65536
SOLVENT CDCl3
NS 2048
DS 4
SWH 29761.904 Hz
FIDRES 0.908261 Hz
AQ 1.1010048 sec
RG 203
DW 16.800 usec
DE 6.50 usec
TE 600.6 K
D1 2.00000000 sec
D11 0.03000000 sec
TD0 1
SFO1 125.7779086 MHz
NUC1 13C
P0 3.47 usec
P1 10.40 usec
PLW1 110.00000000 W
SFO2 500.1620006 MHz
NUC2 1H
CPDPRG[2 waltz65
PCPD2 80.00 usec
PLW2 18.00000000 W
PLW12 0.37195000 W
PLW13 0.18708999 W
F2 - Processing parameters
SI 32768
SF 125.7653153 MHz
WDW EM
SSB 0
LB 1.00 Hz
GB 0
PC 1.40

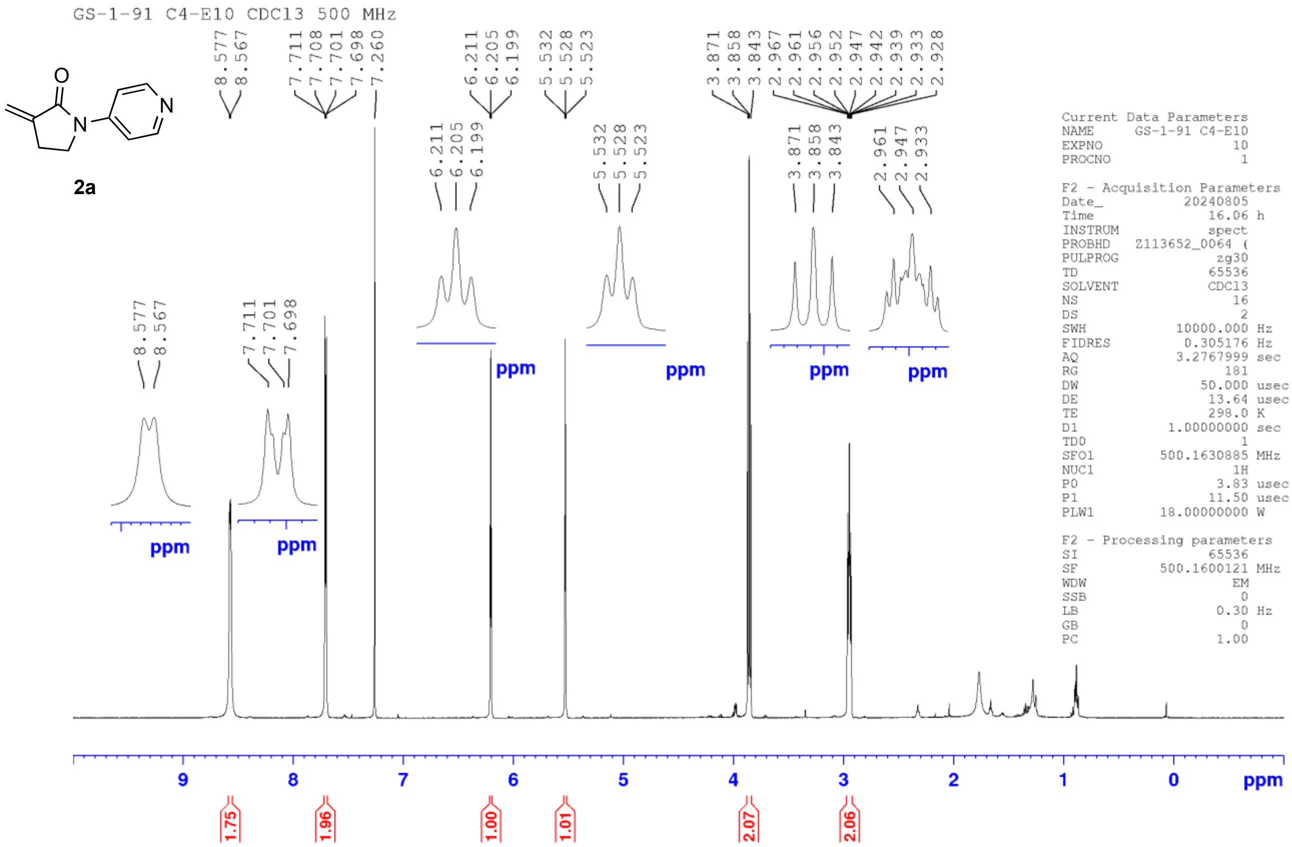
GS-1-91 C4-E10 CDCl3 500 MHz
2a
8.577
8.567
7.711
7.708
7.701
7.698
7.260
6.211
6.205
6.199
5.532
5.528
5.523
3.871
3.858
3.843
2.967
2.961
2.956
2.952
2.947
2.942
2.939
2.933
2.928
Current Data Parameters
NAME GS-1-91 C4-E10
EXPNO 10
PROCNO 1
F2 - Acquisition Parameters
Date_ 20240805
Time 16.06 h
INSTRUM spect
PROBHD Z113652_0064 (
PULPROG zg30
TD 65536
SOLVENT CDCl3
NS 16
DS 2
SWH 10000.000 Hz
FIDRES 0.305176 Hz
AQ 3.2767999 sec
RG 181
DW 50.000 usec
DE 13.64 usec
TE 298.0 K
D1 1.00000000 sec
TD0 1
SFO1 500.1630885 MHz
NUC1 1H
P0 3.83 usec
P1 11.50 usec
PLW1 18.00000000 W
F2 - Processing parameters
SI 65536
SF 500.1600121 MHz
WDW EM
SSB 0
LB 0.30 Hz
GB 0
PC 1.00
9 8 7 6 5 4 3 2 1 0 ppm
1.75
1.96
1.00
1.01
2.07
2.06

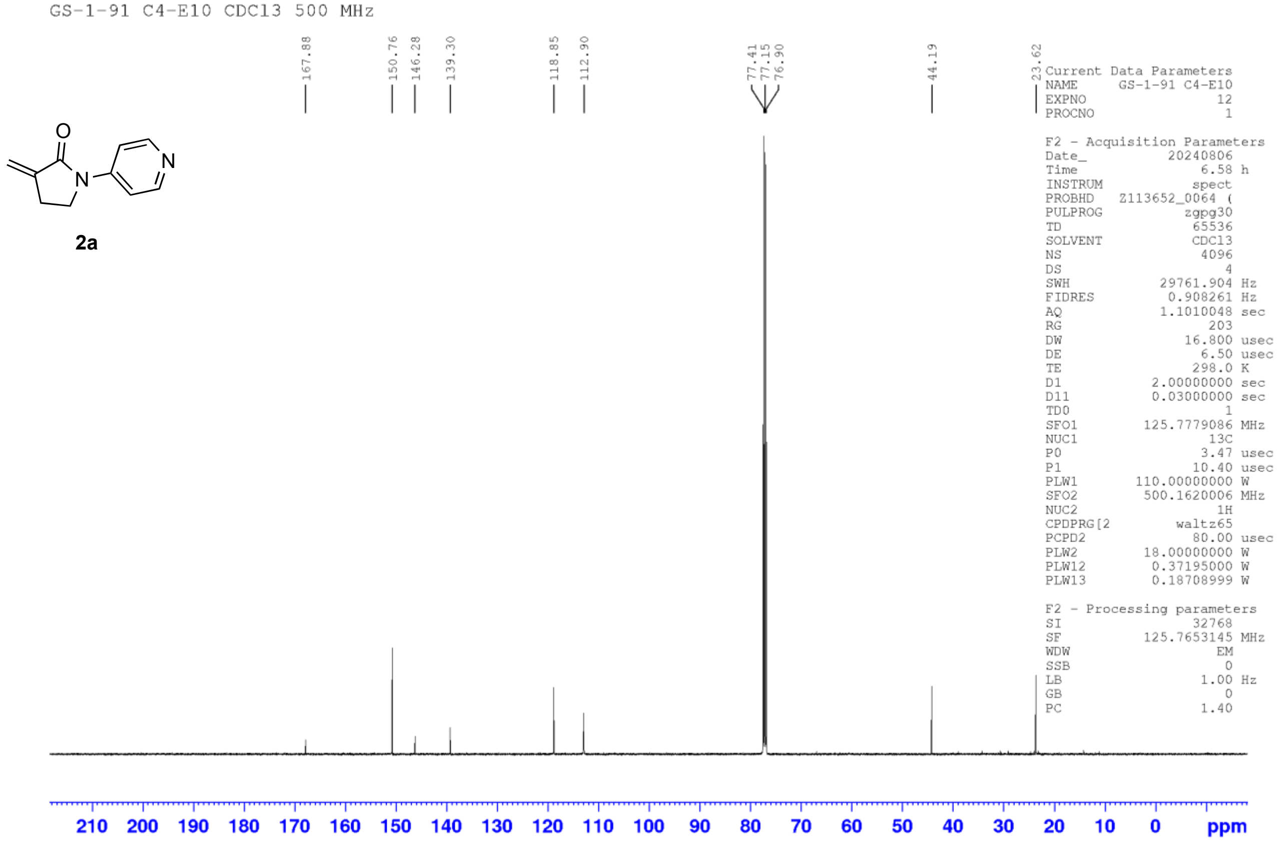
GS-1-91 C4-E10 CDCl3 500 MHz
2a
167.88
150.76
146.28
139.30
118.85
112.90
77.41
77.15
76.90
44.19
23.62
Current Data Parameters
NAME GS-1-91 C4-E10
EXPNO 12
PROCNO 1
F2 - Acquisition Parameters
Date_ 20240806
Time 6.58 h
INSTRUM spect
PROBHD Z113652_0064 (
PULPROG zgpg30
TD 65536
SOLVENT CDCl3
NS 4096
DS 4
SWH 29761.904 Hz
FIDRES 0.908261 Hz
AQ 1.1010048 sec
RG 203
DW 16.800 usec
DE 6.50 usec
TE 298.0 K
D1 2.00000000 sec
D11 0.03000000 sec
TD0 1
SFO1 125.7779086 MHz
NUC1 13C
P0 3.47 usec
P1 10.40 usec
PLW1 110.00000000 W
SFO2 500.1620006 MHz
NUC2 1H
CPDPRG[2 waltz65
PCPD2 80.00 usec
PLW2 18.00000000 W
PLW12 0.37195000 W
PLW13 0.18708999 W
F2 - Processing parameters
SI 32768
SF 125.7653145 MHz
WDW EM
SSB 0
LB 1.00 Hz
GB 0
PC 1.40
210 200 190 180 170 160 150 140 130 120 110 100 90 80 70 60 50 40 30 20 10 0 ppm

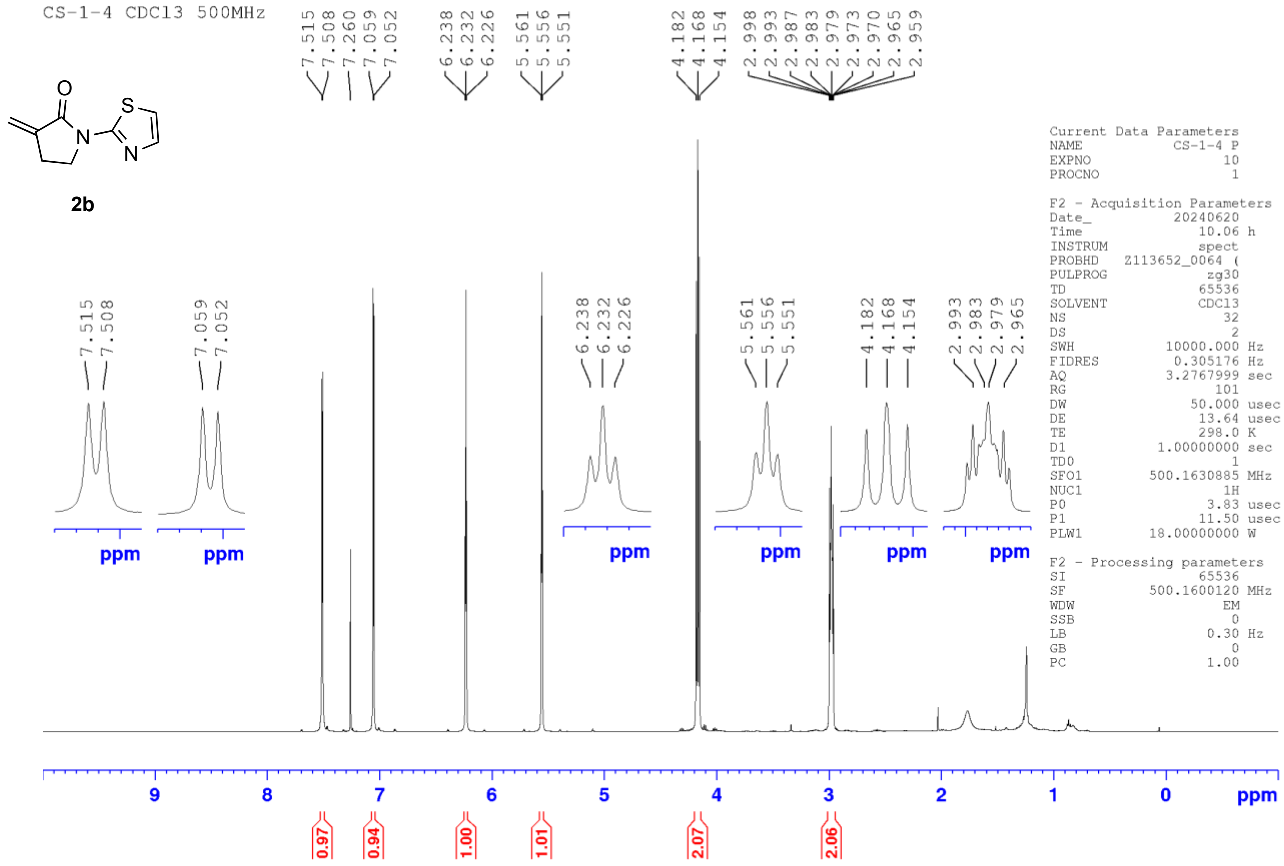
CS-1-4 CDCl3 500MHz
2b
7.515
7.508
7.260
7.059
7.052
6.238
6.232
6.226
5.561
5.556
5.551
4.182
4.168
4.154
2.998
2.993
2.987
2.983
2.979
2.973
2.970
2.965
2.959
2.993
2.983
2.979
2.965
ppm
0.97
0.94
1.00
1.01
2.07
2.06
Current Data Parameters
NAME CS-1-4 P
EXPNO 10
PROCNO 1
F2 - Acquisition Parameters
Date_ 20240620
Time 10.06 h
INSTRUM spect
PROBHD Z113652_0064 (
PULPROG zg30
TD 65536
SOLVENT CDCl3
NS 32
DS 2
SWH 10000.000 Hz
FIDRES 0.305176 Hz
AQ 3.2767999 sec
RG 101
DW 50.000 usec
DE 13.64 usec
TE 298.0 K
D1 1.00000000 sec
TD0 1
SFO1 500.1630885 MHz
NUC1 1H
P0 3.83 usec
P1 11.50 usec
PLW1 18.00000000 W
F2 - Processing parameters
SI 65536
SF 500.1600120 MHz
WDW EM
SSB 0
LB 0.30 Hz
GB 0
PC 1.00

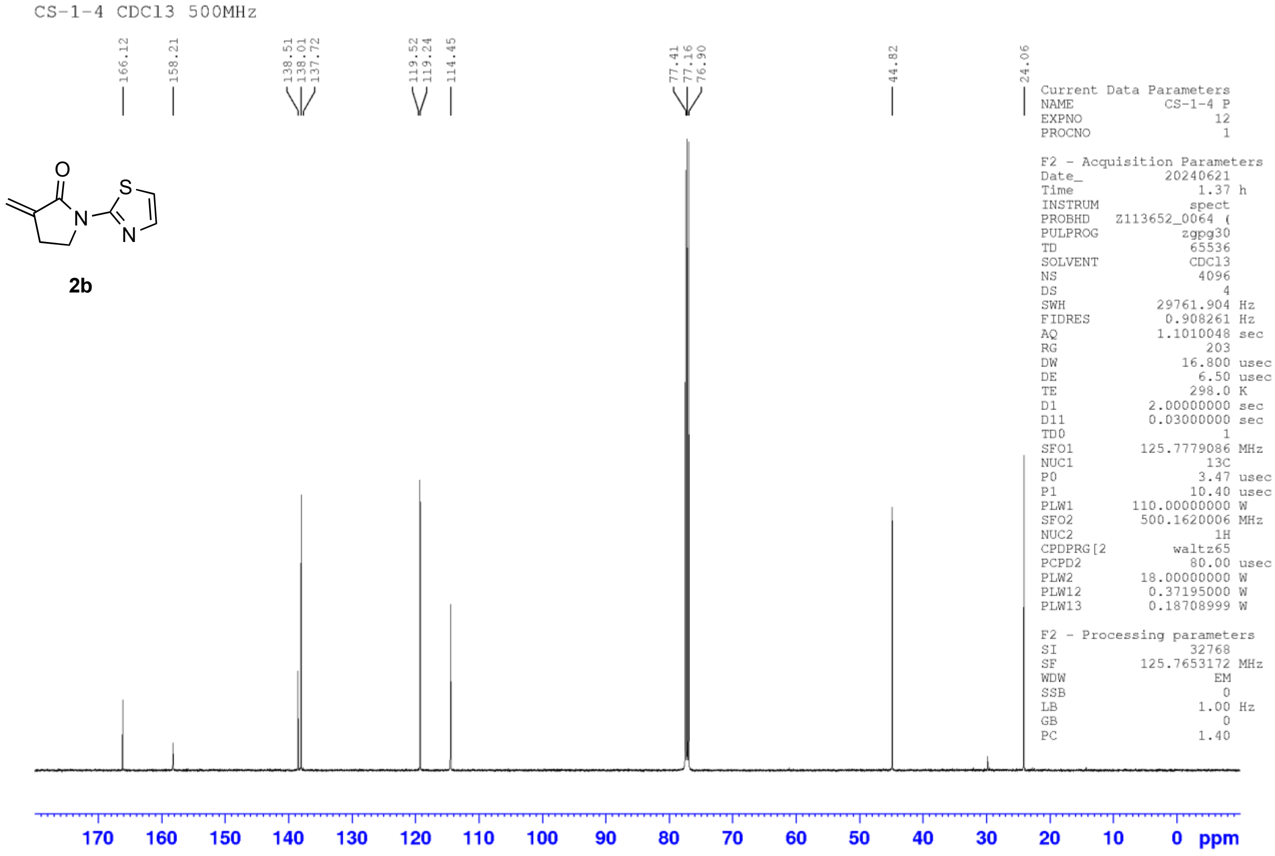
CS-1-4 CDCl3 500MHz
2b
166.12
158.21
138.51
138.01
137.72
119.52
119.24
114.45
77.41
77.16
76.90
44.82
24.06
ppm
Current Data Parameters
NAME CS-1-4 P
EXPNO 12
PROCNO 1
F2 - Acquisition Parameters
Date_ 20240621
Time 1.37 h
INSTRUM spect
PROBHD Z113652_0064 (
PULPROG zgpg30
TD 65536
SOLVENT CDCl3
NS 4096
DS 4
SWH 29761.904 Hz
FIDRES 0.908261 Hz
AQ 1.1010048 sec
RG 203
DW 16.800 usec
DE 6.50 usec
TE 298.0 K
D1 2.00000000 sec
D11 0.03000000 sec
TD0 1
SFO1 125.7779086 MHz
NUC1 13C
P0 3.47 usec
P1 10.40 usec
PLW1 110.00000000 W
SFO2 500.1620006 MHz
NUC2 1H
CPDPRG[2 waltz65
PCPD2 80.00 usec
PLW2 18.00000000 W
PLW12 0.37195000 W
PLW13 0.18708999 W
F2 - Processing parameters
SI 32768
SF 125.7653172 MHz
WDW EM
SSB 0
LB 1.00 Hz
GB 0
PC 1.40

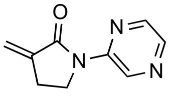
O
N
N
N

2c

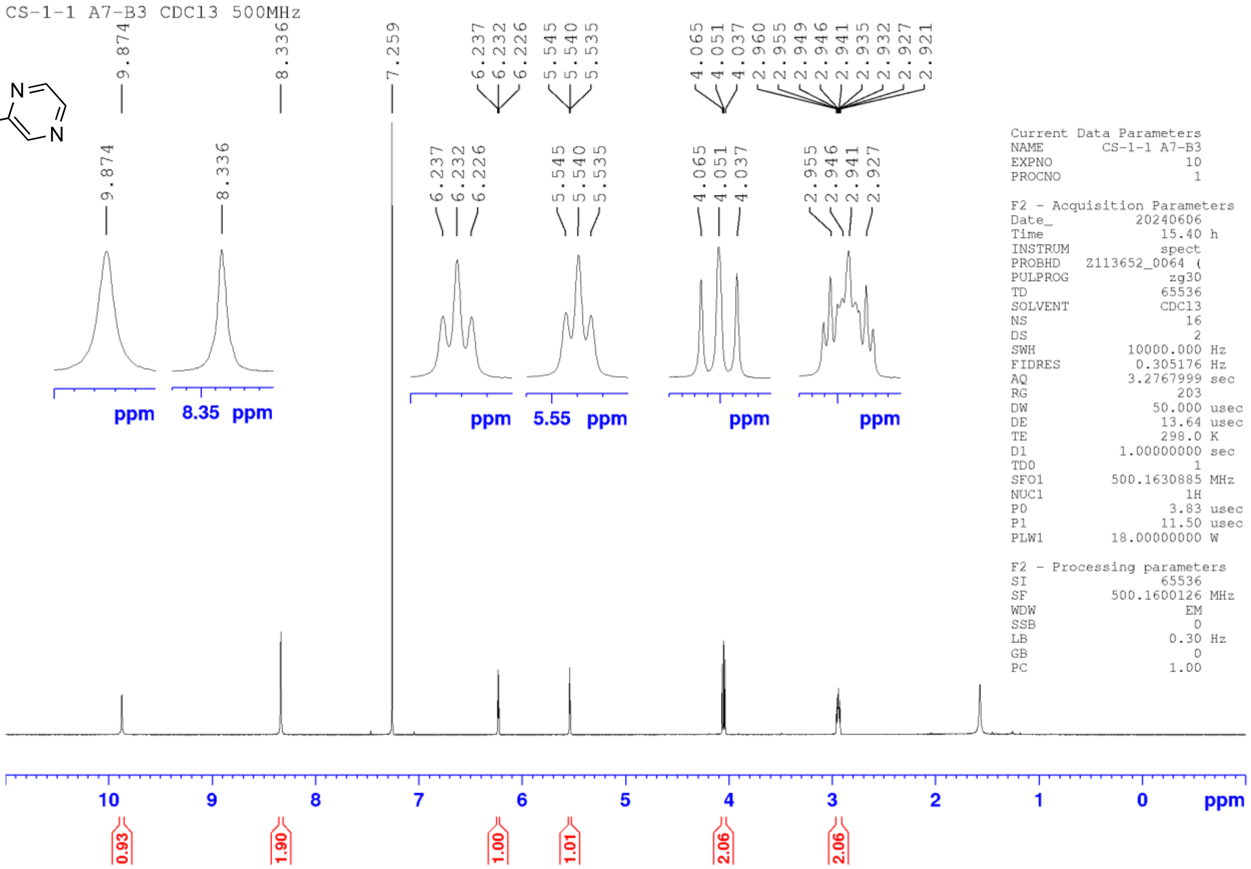
CS-1-1 A7-B3 CDCl3 500MHz
9.874
8.336
7.259
6.237
6.232
6.226
5.545
5.540
5.535
4.065
4.051
4.037
2.960
2.955
2.949
2.946
2.941
2.935
2.932
2.927
2.921
8.35 ppm
5.55 ppm
Current Data Parameters
NAME CS-1-1 A7-B3
EXPNO 10
PROCNO 1
F2 - Acquisition Parameters
Date_ 20240606
Time 15.40 h
INSTRUM spect
PROBHD Z113652_0064 (
PULPROG zg30
TD 65536
SOLVENT CDCl3
NS 16
DS 2
SWH 10000.000 Hz
FIDRES 0.305176 Hz
AQ 3.2767999 sec
RG 203
DW 50.000 usec
DE 13.64 usec
TE 298.0 K
D1 1.00000000 sec
TD0 1
SFO1 500.1630885 MHz
NUC1 1H
P0 3.83 usec
P1 11.50 usec
PLW1 18.00000000 W
F2 - Processing parameters
SI 65536
SF 500.1600126 MHz
WDW EM
SSB 0
LB 0.30 Hz
GB 0
PC 1.00
10 9 8 7 6 5 4 3 2 1 0 ppm
0.93
1.90
1.00
1.01
2.06
2.06

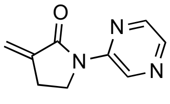
O
N
N
N

2c

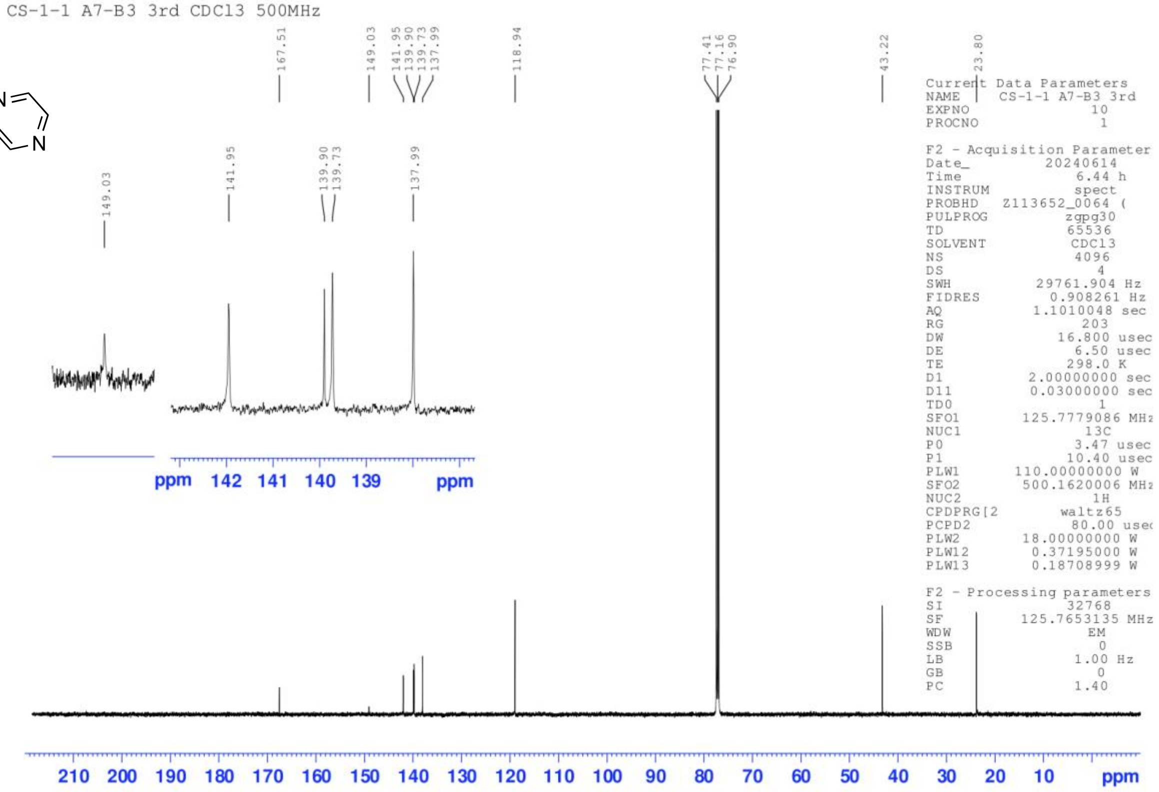
CS-1-1 A7-B3 3rd CDCl3 500MHz
167.51
149.03
141.95
139.90
139.73
137.99
118.94
77.41
77.16
76.90
43.22
23.80
142 141 140 139 ppm
Current Data Parameters
NAME CS-1-1 A7-B3 3rd
EXPNO 10
PROCNO 1
F2 - Acquisition Parameter
Date_ 20240614
Time 6.44 h
INSTRUM spect
PROBHD Z113652_0064 (
PULPROG zgpg30
TD 65536
SOLVENT CDCl3
NS 4096
DS 4
SWH 29761.904 Hz
FIDRES 0.908261 Hz
AQ 1.1010048 sec
RG 203
DW 16.800 usec
DE 6.50 usec
TE 298.0 K
D1 2.00000000 sec
D11 0.03000000 sec
TD0 1
SFO1 125.7779086 MHz
NUC1 13C
P0 3.47 usec
P1 10.40 usec
PLW1 110.00000000 W
SFO2 500.1620006 MHz
NUC2 1H
CPDPRG[2 waltz65
PCPD2 80.00 usec
PLW2 18.00000000 W
PLW12 0.37195000 W
PLW13 0.18708999 W
F2 - Processing parameters
SI 32768
SF 125.7653135 MHz
WDW EM
SSB 0
LB 1.00 Hz
GB 0
PC 1.40
210 200 190 180 170 160 150 140 130 120 110 100 90 80 70 60 50 40 30 20 10 ppm

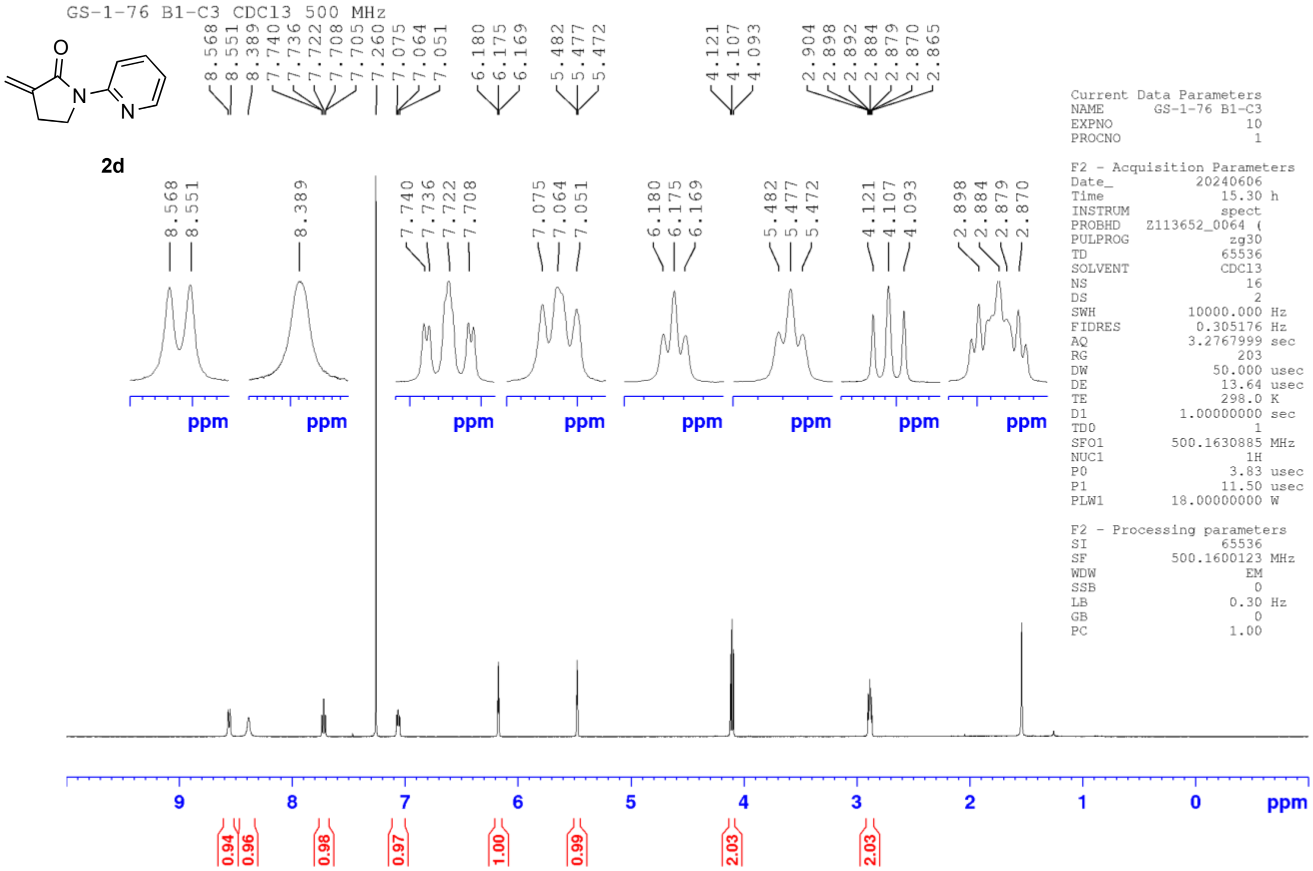
GS-1-76 B1-C3 CDCl3 500 MHz
2d
8.568
8.551
8.389
7.740
7.736
7.722
7.708
7.705
7.260
7.075
7.064
7.051
6.180
6.175
6.169
5.482
5.477
5.472
4.121
4.107
4.093
2.904
2.898
2.892
2.884
2.879
2.870
2.865
Current Data Parameters
NAME GS-1-76 B1-C3
EXPNO 10
PROCNO 1
F2 - Acquisition Parameters
Date_ 20240606
Time 15.30 h
INSTRUM spect
PROBHD Z113652_0064 (
PULPROG zg30
TD 65536
SOLVENT CDCl3
NS 16
DS 2
SWH 10000.000 Hz
FIDRES 0.305176 Hz
AQ 3.2767999 sec
RG 203
DW 50.000 usec
DE 13.64 usec
TE 298.0 K
D1 1.00000000 sec
TD0 1
SFO1 500.1630885 MHz
NUC1 1H
P0 3.83 usec
P1 11.50 usec
PLW1 18.00000000 W
F2 - Processing parameters
SI 65536
SF 500.1600123 MHz
WDW EM
SSB 0
LB 0.30 Hz
GB 0
PC 1.00
ppm
9
8
7
6
5
4
3
2
1
0
ppm
0.94
0.96
0.98
0.97
1.00
0.99
2.03
2.03

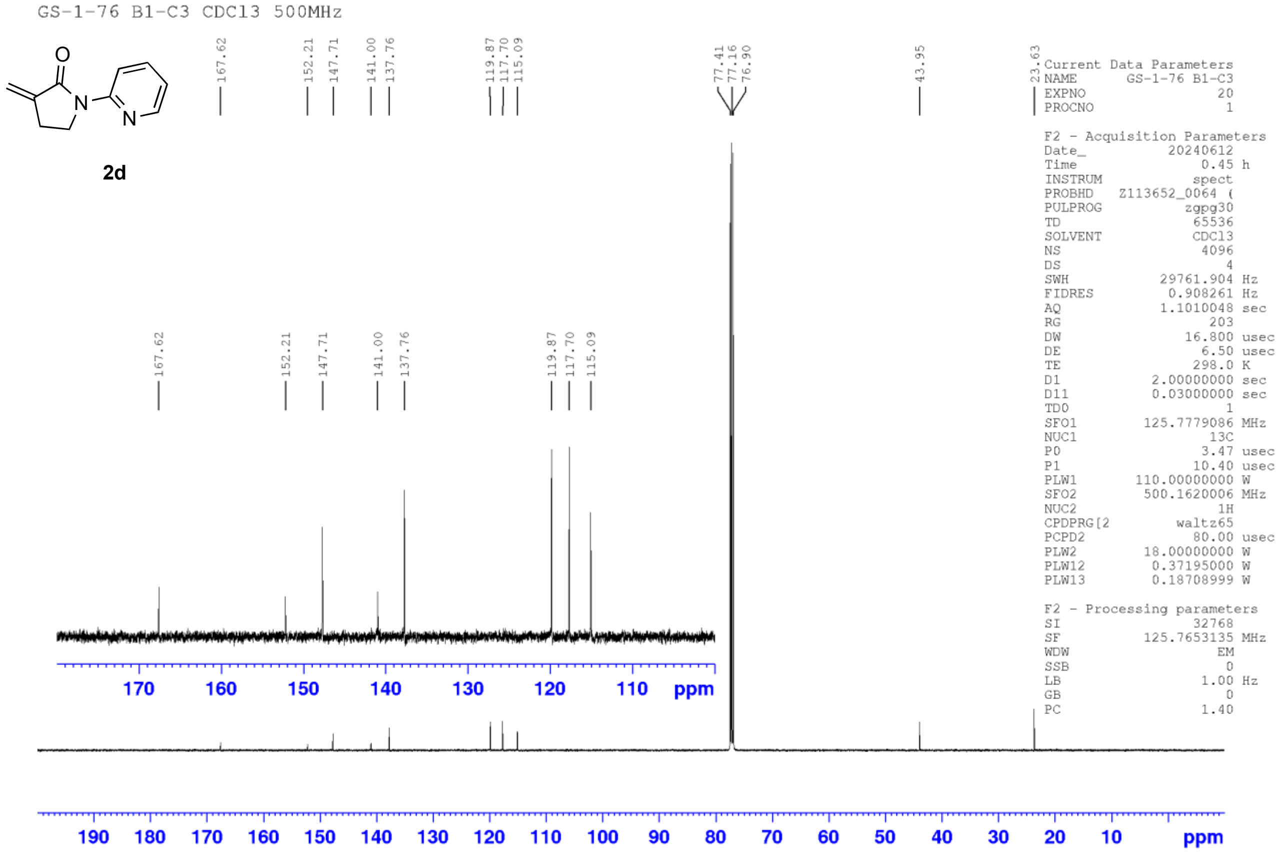
GS-1-76 B1-C3 CDCl3 500MHz
2d
167.62
152.21
147.71
141.00
137.76
119.87
117.70
115.09
77.41
77.16
76.90
43.95
23.63
Current Data Parameters
NAME GS-1-76 B1-C3
EXPNO 20
PROCNO 1
F2 - Acquisition Parameters
Date_ 20240612
Time 0.45 h
INSTRUM spect
PROBHD Z113652_0064 (
PULPROG zgpg30
TD 65536
SOLVENT CDCl3
NS 4096
DS 4
SWH 29761.904 Hz
FIDRES 0.908261 Hz
AQ 1.1010048 sec
RG 203
DW 16.800 usec
DE 6.50 usec
TE 298.0 K
D1 2.00000000 sec
D11 0.03000000 sec
TD0 1
SFO1 125.7779086 MHz
NUC1 13C
P0 3.47 usec
P1 10.40 usec
PLW1 110.00000000 W
SFO2 500.1620006 MHz
NUC2 1H
CPDPRG[2 waltz65
PCPD2 80.00 usec
PLW2 18.00000000 W
PLW12 0.37195000 W
PLW13 0.18708999 W
F2 - Processing parameters
SI 32768
SF 125.7653135 MHz
WDW EM
SSB 0
LB 1.00 Hz
GB 0
PC 1.40
170
160
150
140
130
120
110
ppm
190
180
170
160
150
140
130
120
110
100
90
80
70
60
50
40
30
20
10
ppm

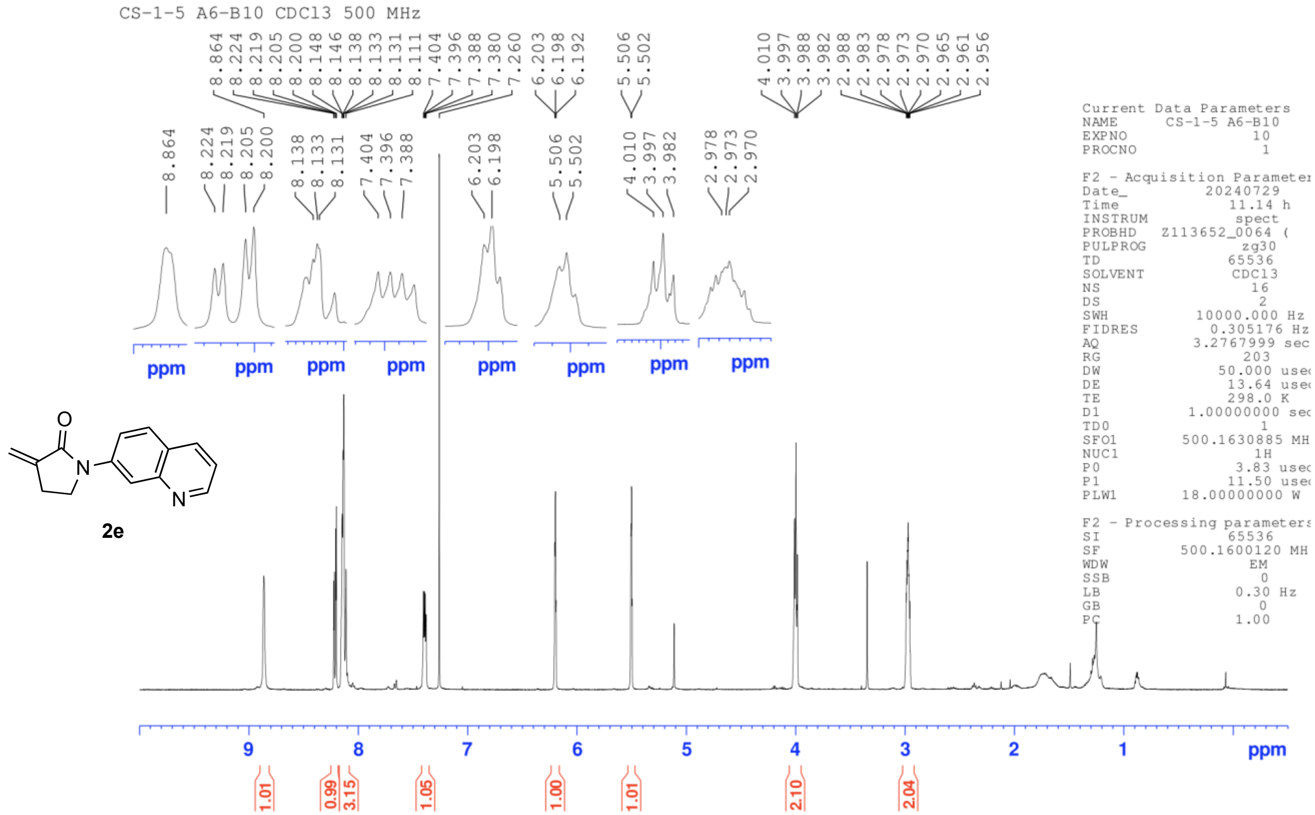
CS-1-5 A6-B10 CDCl3 500 MHz
8.864 8.224 8.219 8.205 8.200 8.148 8.146 8.138 8.133 8.131 8.111 7.404 7.396 7.388 7.380 7.260 6.203 6.198 6.192 5.506 5.502 4.010 3.997 3.988 3.982 2.988 2.983 2.978 2.973 2.970 2.965 2.961 2.956
Current Data Parameters
NAME CS-1-5 A6-B10
EXPNO 10
PROCNO 1
F2 - Acquisition Parameter
Date_ 20240729
Time 11.14 h
INSTRUM spect
PROBHD Z113652_0064 (
PULPROG zg30
TD 65536
SOLVENT CDCl3
NS 16
DS 2
SWH 10000.000 Hz
FIDRES 0.305176 Hz
AQ 3.2767999 sec
RG 203
DW 50.000 usec
DE 13.64 usec
TE 298.0 K
D1 1.00000000 sec
TD0 1
SFO1 500.1630885 MH
NUC1 1H
P0 3.83 usec
P1 11.50 usec
PLW1 18.00000000 W
F2 - Processing parameters
SI 65536
SF 500.1600120 MH
WDW EM
SSB 0
LB 0.30 Hz
GB 0
PC 1.00
2e
1.01 0.99 3.15 1.05 1.00 1.01 2.10 2.04
ppm

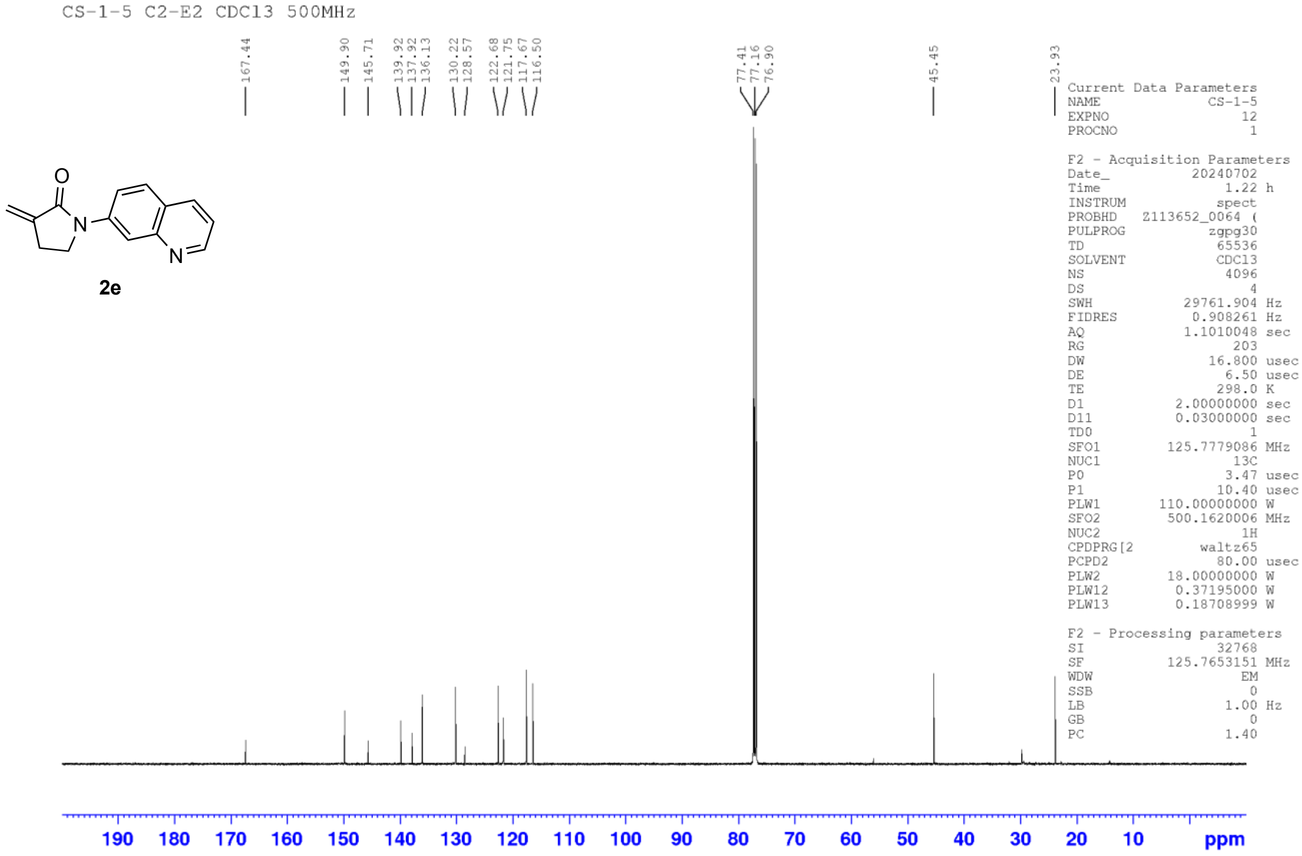
CS-1-5 C2-E2 CDCl3 500MHz
167.44 149.90 145.71 139.92 137.92 136.13 130.22 128.57 122.68 121.75 117.67 116.50 77.41 77.16 76.90 45.45 23.93
Current Data Parameters
NAME CS-1-5
EXPNO 12
PROCNO 1
F2 - Acquisition Parameters
Date_ 20240702
Time 1.22 h
INSTRUM spect
PROBHD Z113652_0064 (
PULPROG zgpg30
TD 65536
SOLVENT CDCl3
NS 4096
DS 4
SWH 29761.904 Hz
FIDRES 0.908261 Hz
AQ 1.1010048 sec
RG 203
DW 16.800 usec
DE 6.50 usec
TE 298.0 K
D1 2.00000000 sec
D11 0.03000000 sec
TD0 1
SFO1 125.7779086 MHz
NUC1 13C
P0 3.47 usec
P1 10.40 usec
PLW1 110.00000000 W
SFO2 500.1620006 MHz
NUC2 1H
CPDPRG[2 waltz65
PCPD2 80.00 usec
PLW2 18.00000000 W
PLW12 0.37195000 W
PLW13 0.18708999 W
F2 - Processing parameters
SI 32768
SF 125.7653151 MHz
WDW EM
SSB 0
LB 1.00 Hz
GB 0
PC 1.40
2e
ppm

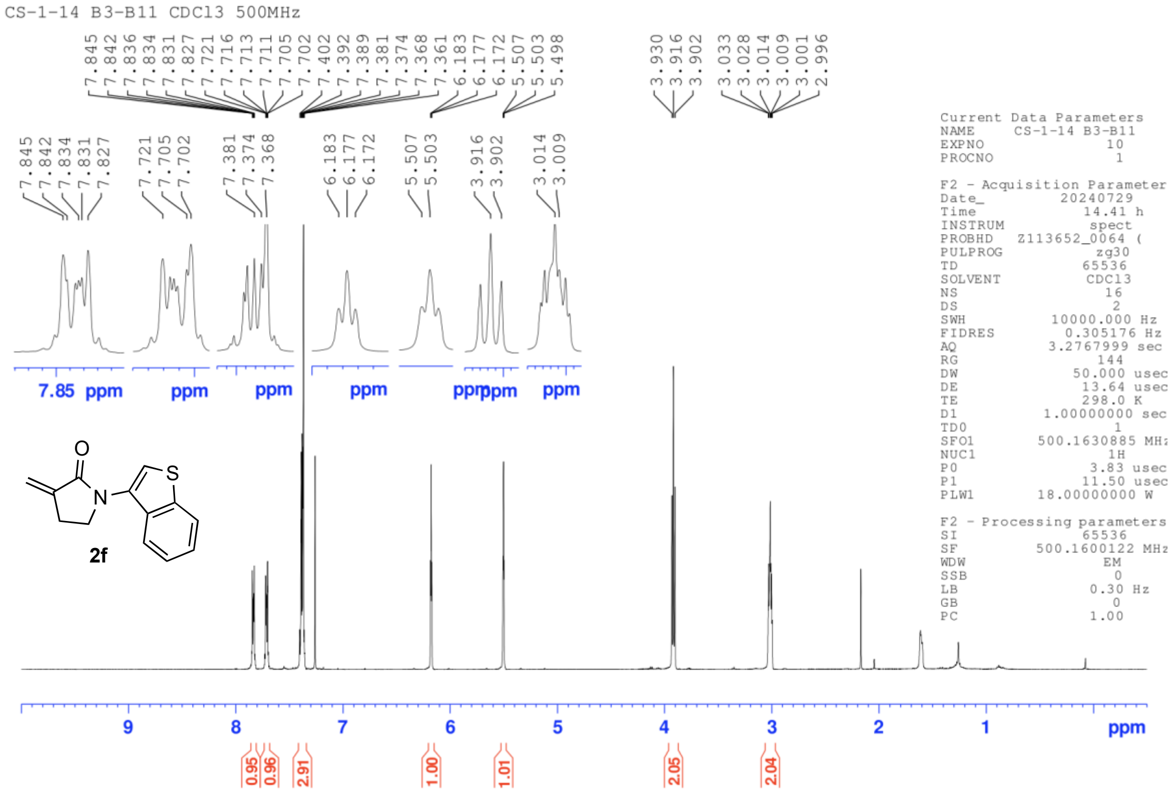
CS-1-14 B3-B11 CDCl3 500MHz
7.845 7.842 7.836 7.834 7.831 7.827 7.721 7.716 7.713 7.711 7.705 7.702 7.402 7.392 7.389 7.381 7.374 7.368 7.361 6.183 6.177 6.172 5.507 5.503 5.498
3.930 3.916 3.902
3.033 3.028 3.014 3.009 3.001 2.996
Current Data Parameters
NAME CS-1-14 B3-B11
EXPNO 10
PROCNO 1
F2 - Acquisition Parameter
Date_ 20240729
Time 14.41 h
INSTRUM spect
PULPROG zg30
TD 65536
SOLVENT CDCl3
NS 16
DS 2
SWH 10000.000 Hz
FIDRES 0.305176 Hz
AQ 3.2767999 sec
RG 144
DW 50.000 usec
DE 13.64 usec
TE 298.0 K
D1 1.00000000 sec
TD0 1
NUC1 1H
P0 3.83 usec
P1 11.50 usec
PLW1 18.00000000 W
F2 - Processing parameters
SI 65536
SF 500.1600122 MHz
WDW EM
SSB 0
LB 0.30 Hz
GB 0
PC 1.00
2f
ppm
0.95 0.96 2.91 1.00 1.01 2.05 2.04

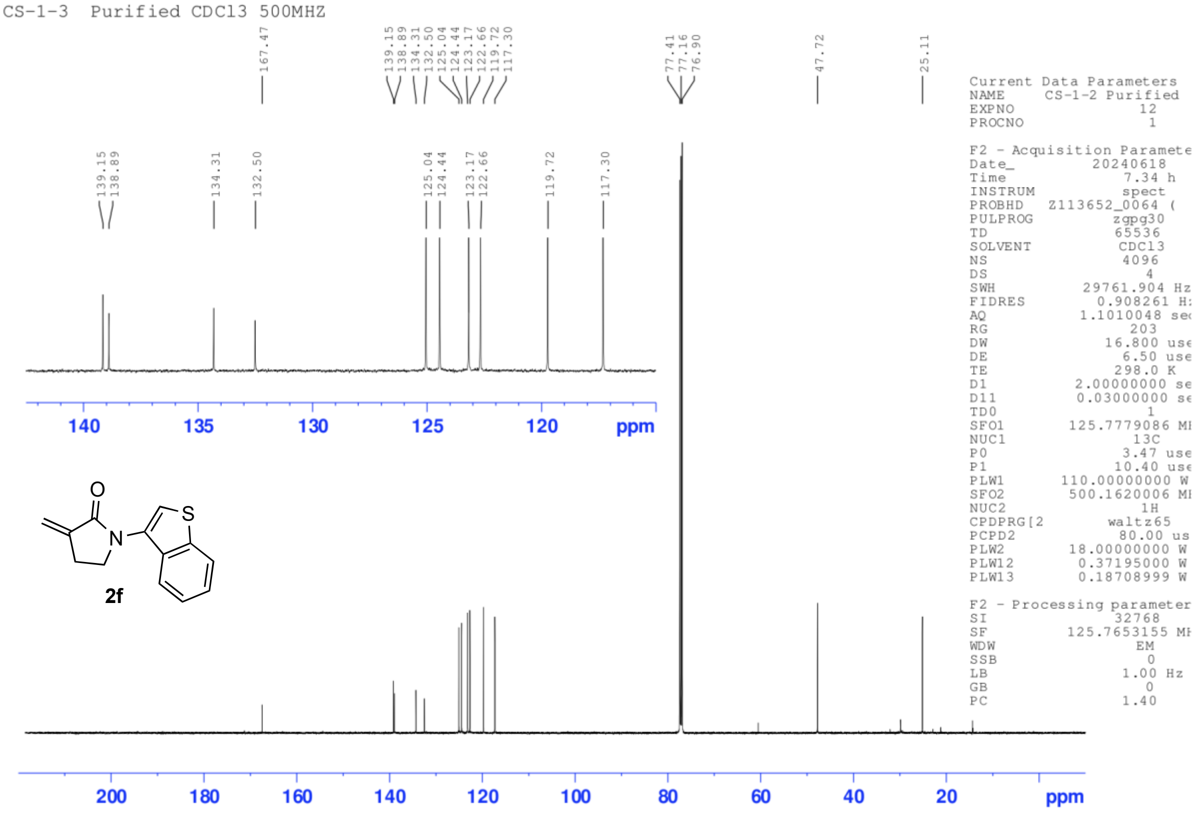
CS-1-3 Purified CDCl3 500MHZ
167.47
139.15 138.89 134.31 132.50 125.04 124.44 123.17 122.66 119.72 117.30
77.41 77.16 76.90
47.72
25.11
Current Data Parameters
NAME CS-1-2 Purified
EXPNO 12
PROCNO 1
Date_ 20240618
Time 7.34 h
INSTRUM spect
PULPROG zgpg30
TD 65536
SOLVENT CDCl3
NS 4096
DS 4
SWH 29761.904 Hz
RG 203
TE 298.0 K
NUC1 13C
NUC2 1H
CPDPRG[2 waltz65
F2 - Processing parameter
SI 32768
WDW EM
SSB 0
LB 1.00 Hz
GB 0
PC 1.40
2f
ppm

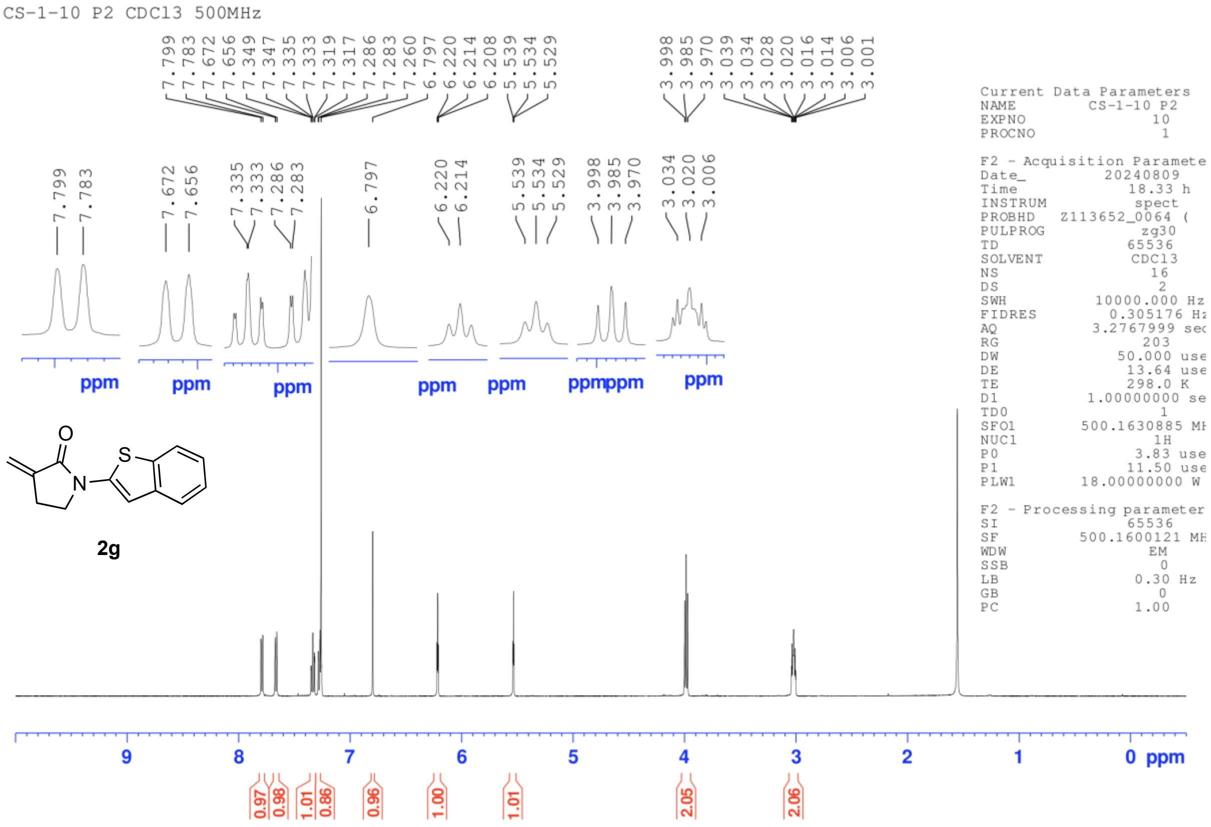
CS-1-10 P2 CDCl3 500MHz
2g
Current Data Parameters
NAME CS-1-10 P2
EXPNO 10
PROCNO 1
F2 - Acquisition Paramete
Date_ 20240809
Time 18.33 h
INSTRUM spect
PULPROG zg30
TD 65536
SOLVENT CDCl3
NS 16
DS 2
SWH 10000.000 Hz
FIDRES 0.305176 Hz
AQ 3.2767999 sec
RG 203
DW 50.000 use
DE 13.64 use
TE 298.0 K
D1 1.00000000 se
TD0 1
SFO1 500.1630885 MH
NUC1 1H
P0 3.83 use
P1 11.50 use
PLW1 18.00000000 W
F2 - Processing parameter
SI 65536
SF 500.1600121 MH
WDW EM
SSB 0
LB 0.30 Hz
GB 0
PC 1.00
ppm

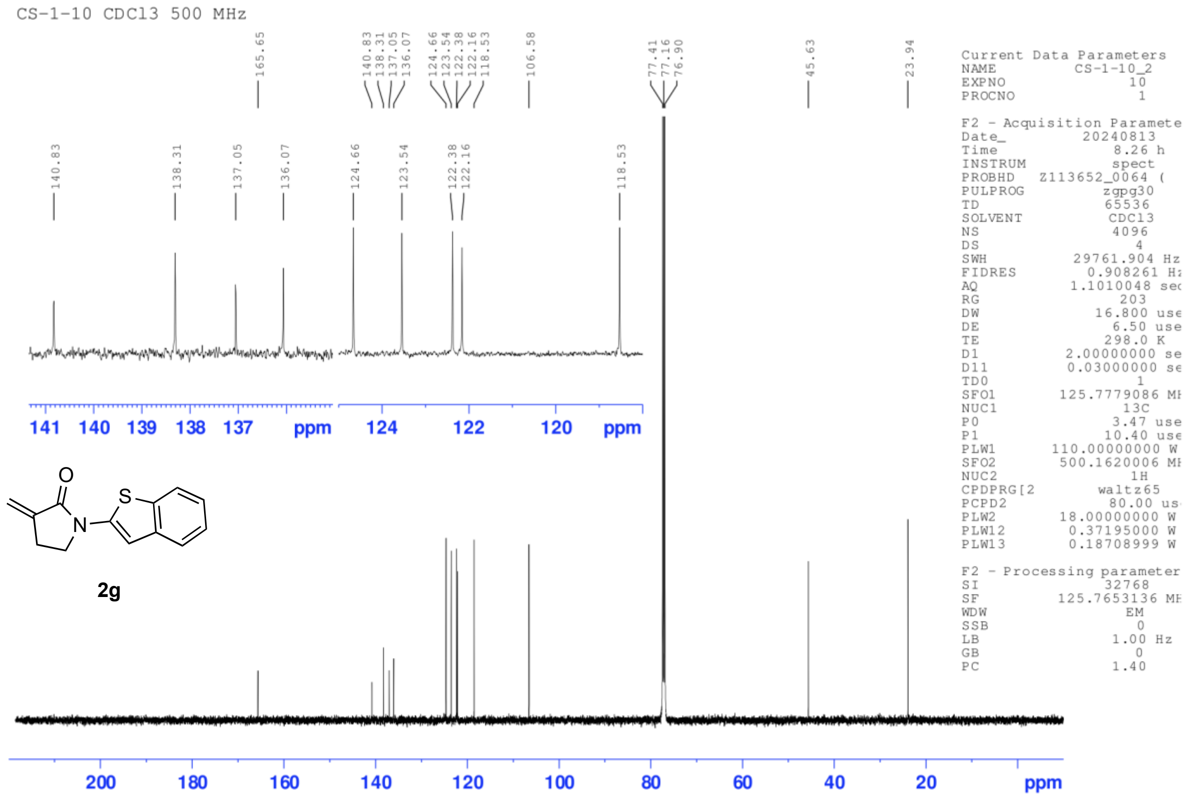
CS-1-10 CDCl3 500 MHz
2g
Current Data Parameters
NAME CS-1-10_2
EXPNO 10
PROCNO 1
F2 - Acquisition Paramete
Date_ 20240813
Time 8.26 h
INSTRUM spect
PULPROG zgpg30
TD 65536
SOLVENT CDCl3
NS 4096
DS 4
SWH 29761.904 Hz
FIDRES 0.908261 Hz
AQ 1.1010048 sec
RG 203
DW 16.800 use
DE 6.50 use
TE 298.0 K
D1 2.00000000 se
D11 0.03000000 se
TD0 1
SFO1 125.7779086 MH
NUC1 13C
P0 3.47 use
P1 10.40 use
PLW1 110.00000000 W
SFO2 500.1620006 MH
NUC2 1H
CPDPRG[2 waltz65
PCPD2 80.00 us
PLW2 18.00000000 W
PLW12 0.37195000 W
PLW13 0.18708999 W
F2 - Processing parameter
SI 32768
SF 125.7653136 MH
WDW EM
SSB 0
LB 1.00 Hz
GB 0
PC 1.40
ppm

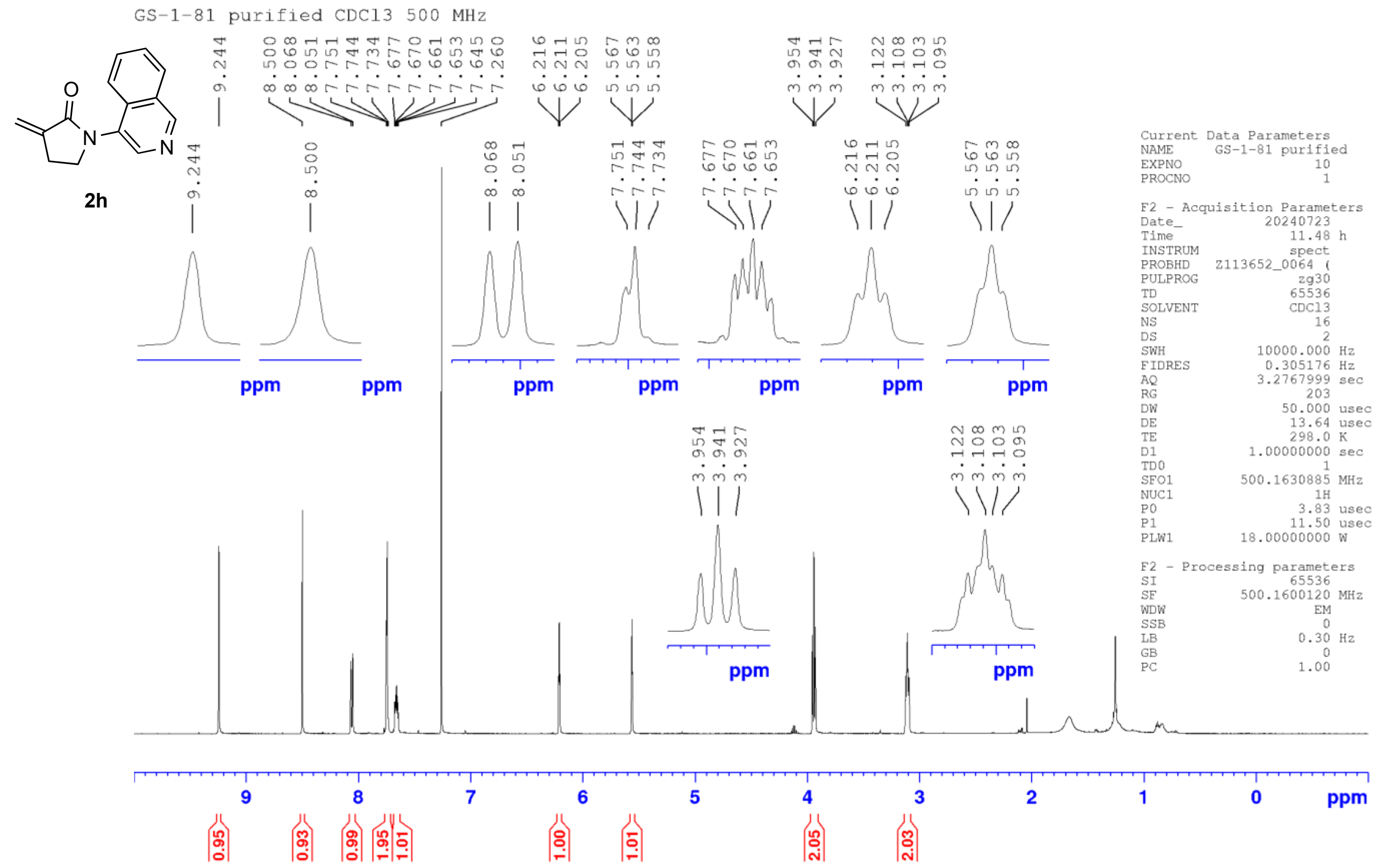
GS-1-81 purified CDCl3 500 MHz
2h
9.244
8.500
8.068
8.051
7.751
7.744
7.734
7.677
7.670
7.661
7.653
7.645
7.260
6.216
6.211
6.205
5.567
5.563
5.558
3.954
3.941
3.927
3.122
3.108
3.103
3.095
0.95
0.93
0.99
1.95
1.01
1.00
1.01
2.05
2.03
ppm
Current Data Parameters
NAME GS-1-81 purified
EXPNO 10
PROCNO 1
F2 - Acquisition Parameters
Date_ 20240723
Time 11.48 h
INSTRUM spect
PROBHD Z113652_0064 (
PULPROG zg30
TD 65536
SOLVENT CDCl3
NS 16
DS 2
SWH 10000.000 Hz
FIDRES 0.305176 Hz
AQ 3.2767999 sec
RG 203
DW 50.000 usec
DE 13.64 usec
TE 298.0 K
D1 1.00000000 sec
TD0 1
SFO1 500.1630885 MHz
NUC1 1H
P0 3.83 usec
P1 11.50 usec
PLW1 18.00000000 W
F2 - Processing parameters
SI 65536
SF 500.1600120 MHz
WDW EM
SSB 0
LB 0.30 Hz
GB 0
PC 1.00

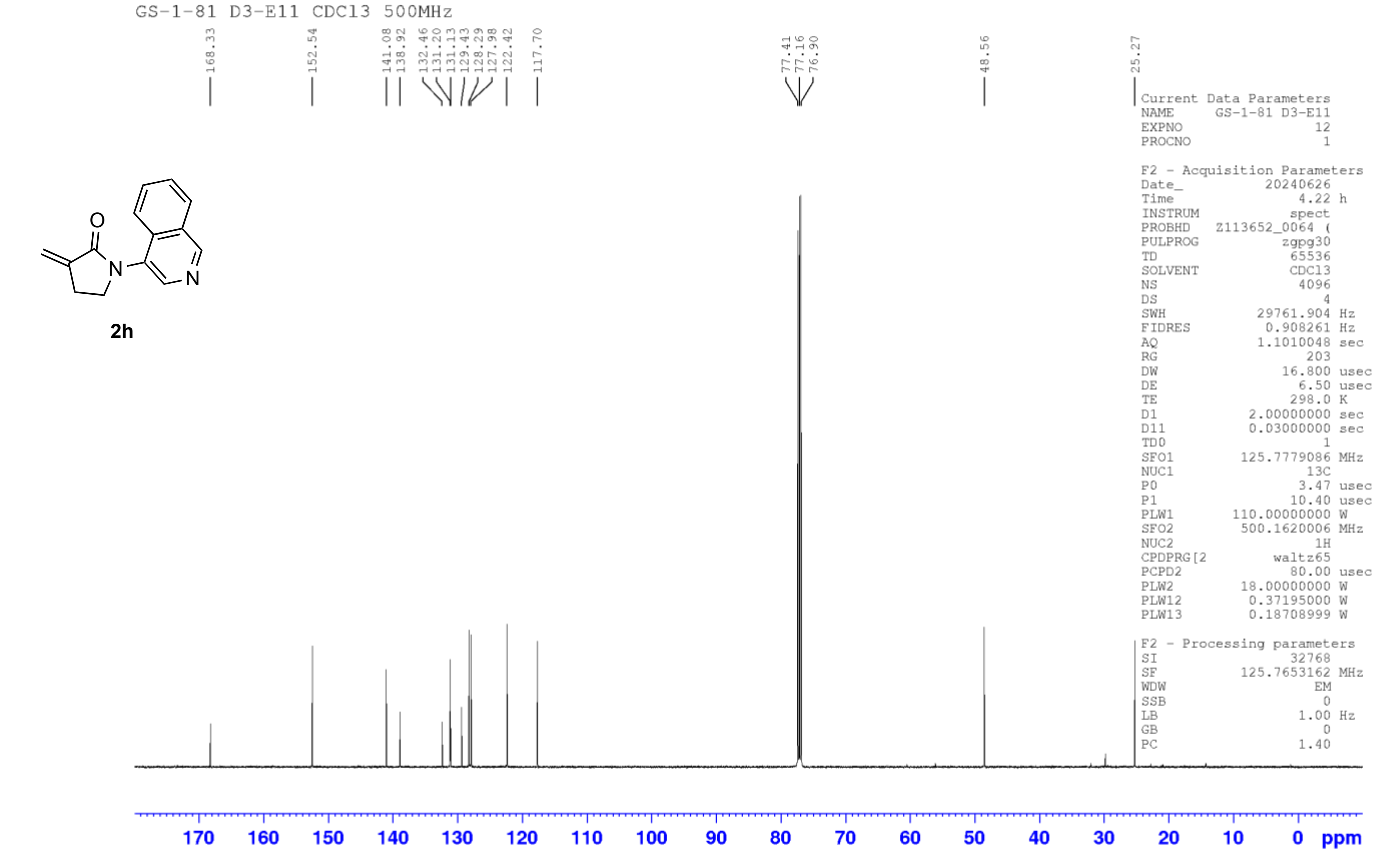
GS-1-81 D3-E11 CDCl3 500MHz
2h
168.33
152.54
141.08
138.92
132.46
131.20
131.13
129.43
128.29
127.98
122.42
117.70
77.41
77.16
76.90
48.56
25.27
ppm
Current Data Parameters
NAME GS-1-81 D3-E11
EXPNO 12
PROCNO 1
F2 - Acquisition Parameters
Date_ 20240626
Time 4.22 h
INSTRUM spect
PROBHD Z113652_0064 (
PULPROG zgpg30
TD 65536
SOLVENT CDCl3
NS 4096
DS 4
SWH 29761.904 Hz
FIDRES 0.908261 Hz
AQ 1.1010048 sec
RG 203
DW 16.800 usec
DE 6.50 usec
TE 298.0 K
D1 2.00000000 sec
D11 0.03000000 sec
TD0 1
SFO1 125.7779086 MHz
NUC1 13C
P0 3.47 usec
P1 10.40 usec
PLW1 110.00000000 W
SFO2 500.1620006 MHz
NUC2 1H
CPDPRG[2 waltz65
PCPD2 80.00 usec
PLW2 18.00000000 W
PLW12 0.37195000 W
PLW13 0.18708999 W
F2 - Processing parameters
SI 32768
SF 125.7653162 MHz
WDW EM
SSB 0
LB 1.00 Hz
GB 0
PC 1.40

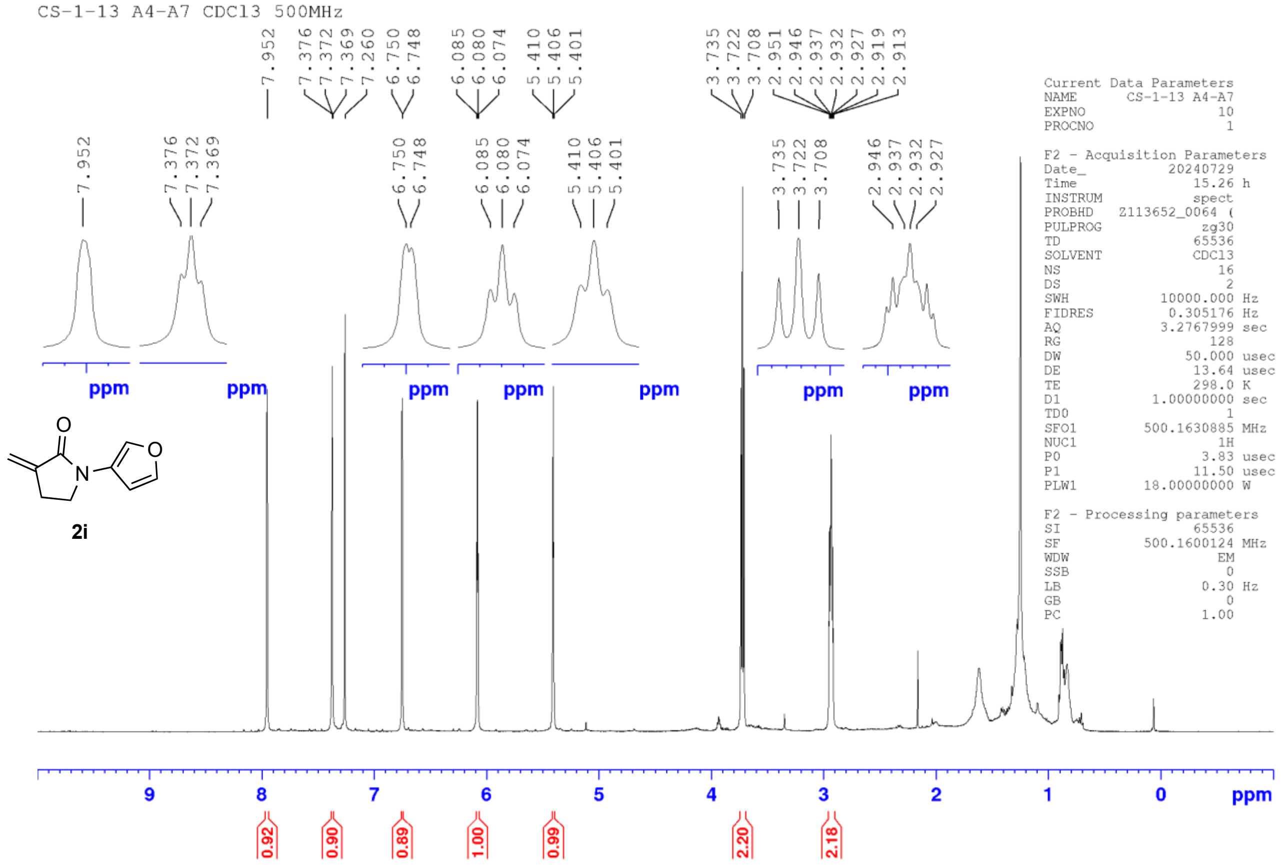

CS-1-13 A4-A7 CDCl3 500MHz
7.952
7.376
7.372
7.369
7.260
6.750
6.748
6.085
6.080
6.074
5.410
5.406
5.401
3.735
3.722
3.708
2.951
2.946
2.937
2.932
2.927
2.919
2.913
Current Data Parameters
NAME CS-1-13 A4-A7
EXPNO 10
PROCNO 1
F2 - Acquisition Parameters
Date_ 20240729
Time 15.26 h
INSTRUM spect
PROBHD Z113652_0064 (
PULPROG zg30
TD 65536
SOLVENT CDCl3
NS 16
DS 2
SWH 10000.000 Hz
FIDRES 0.305176 Hz
AQ 3.2767999 sec
RG 128
DW 50.000 usec
DE 13.64 usec
TE 298.0 K
D1 1.00000000 sec
TD0 1
SFO1 500.1630885 MHz
NUC1 1H
P0 3.83 usec
P1 11.50 usec
PLW1 18.00000000 W
F2 - Processing parameters
SI 65536
SF 500.1600124 MHz
WDW EM
SSB 0
LB 0.30 Hz
GB 0
PC 1.00
2i
0.92
0.90
0.89
1.00
0.99
2.20
2.18
ppm

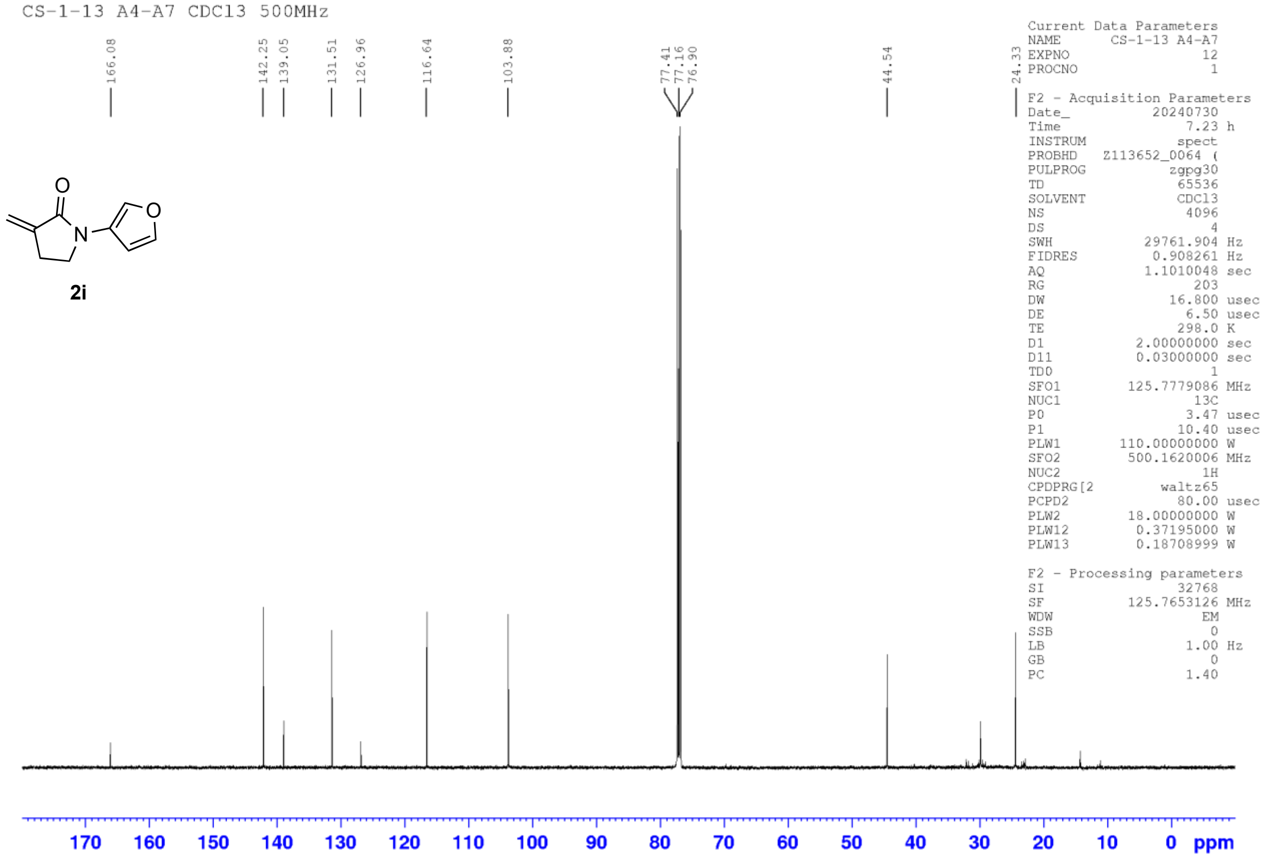

CS-1-13 A4-A7 CDCl3 500MHz
166.08
142.25
139.05
131.51
126.96
116.64
103.88
77.41
77.16
76.90
44.54
24.33
Current Data Parameters
NAME CS-1-13 A4-A7
EXPNO 12
PROCNO 1
F2 - Acquisition Parameters
Date_ 20240730
Time 7.23 h
INSTRUM spect
PROBHD Z113652_0064 (
PULPROG zgpg30
TD 65536
SOLVENT CDCl3
NS 4096
DS 4
SWH 29761.904 Hz
FIDRES 0.908261 Hz
AQ 1.1010048 sec
RG 203
DW 16.800 usec
DE 6.50 usec
TE 298.0 K
D1 2.00000000 sec
D11 0.03000000 sec
TD0 1
SFO1 125.7779086 MHz
NUC1 13C
P0 3.47 usec
P1 10.40 usec
PLW1 110.00000000 W
SFO2 500.1620006 MHz
NUC2 1H
CPDPRG[2 waltz65
PCPD2 80.00 usec
PLW2 18.00000000 W
PLW12 0.37195000 W
PLW13 0.18708999 W
F2 - Processing parameters
SI 32768
SF 125.7653126 MHz
WDW EM
SSB 0
LB 1.00 Hz
GB 0
PC 1.40
2i
ppm

DEPT ^{13}C NMR SPECTRA

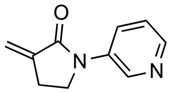

1a

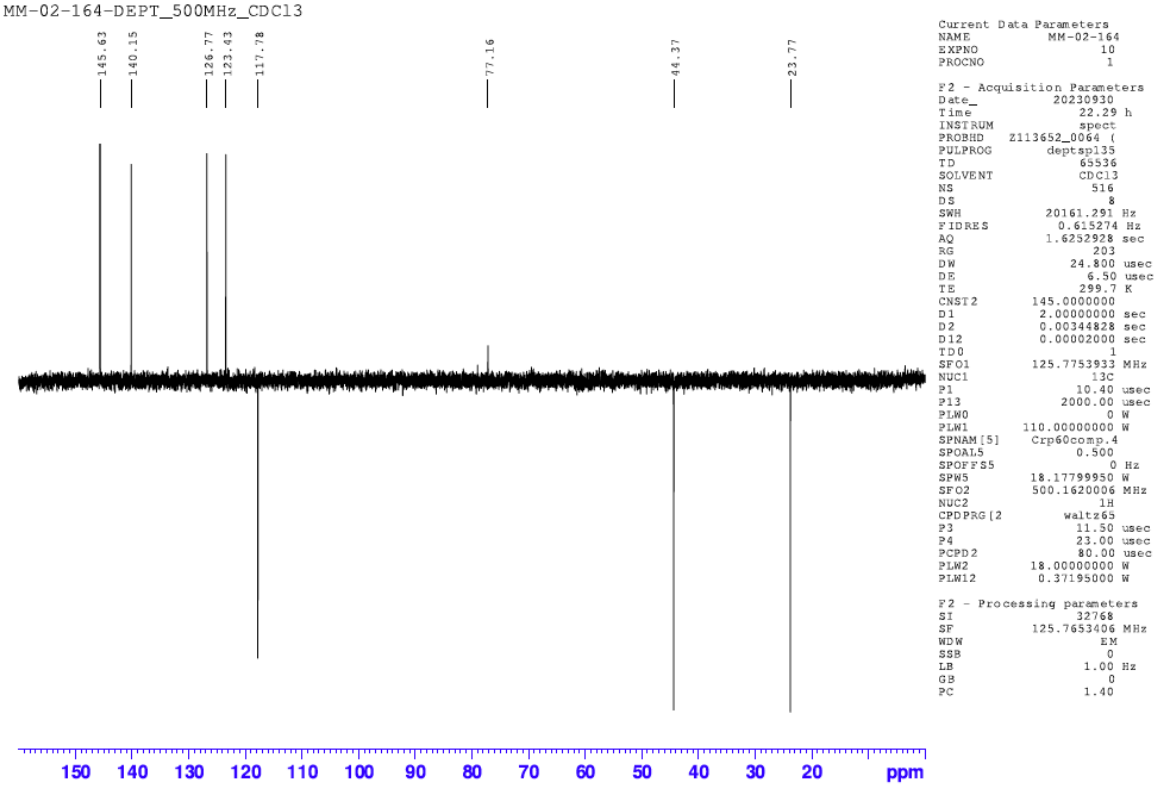

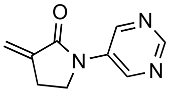

1b

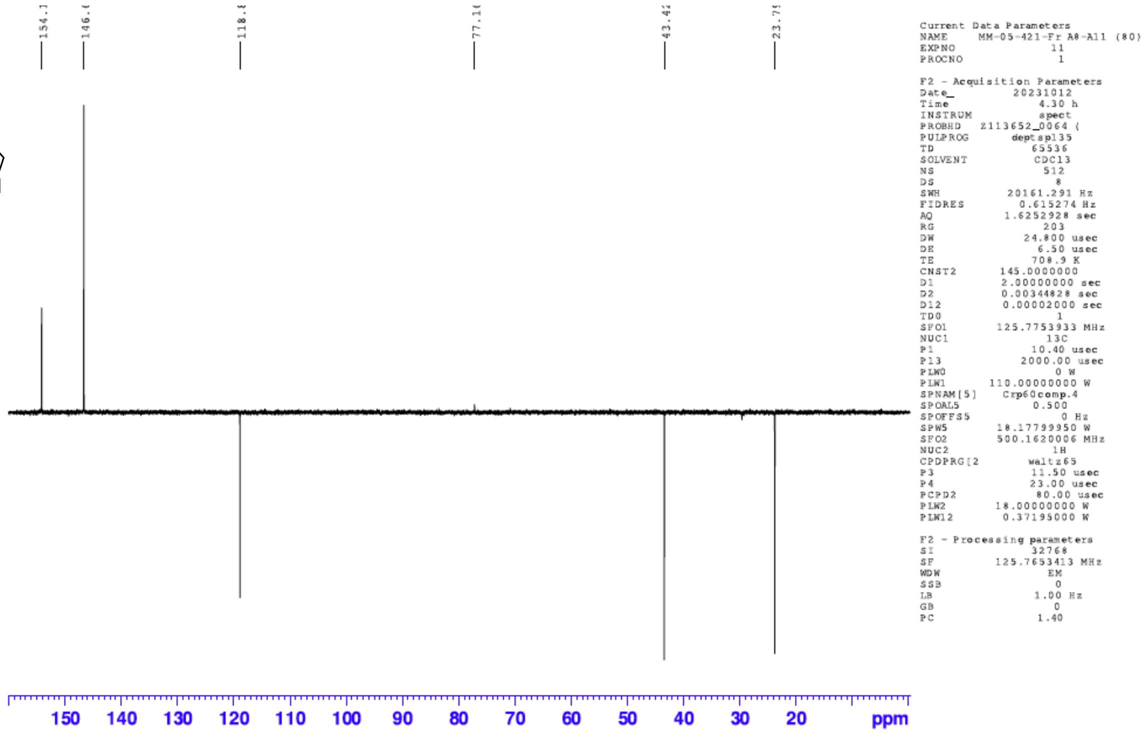

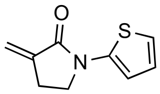
O
S
N

1c

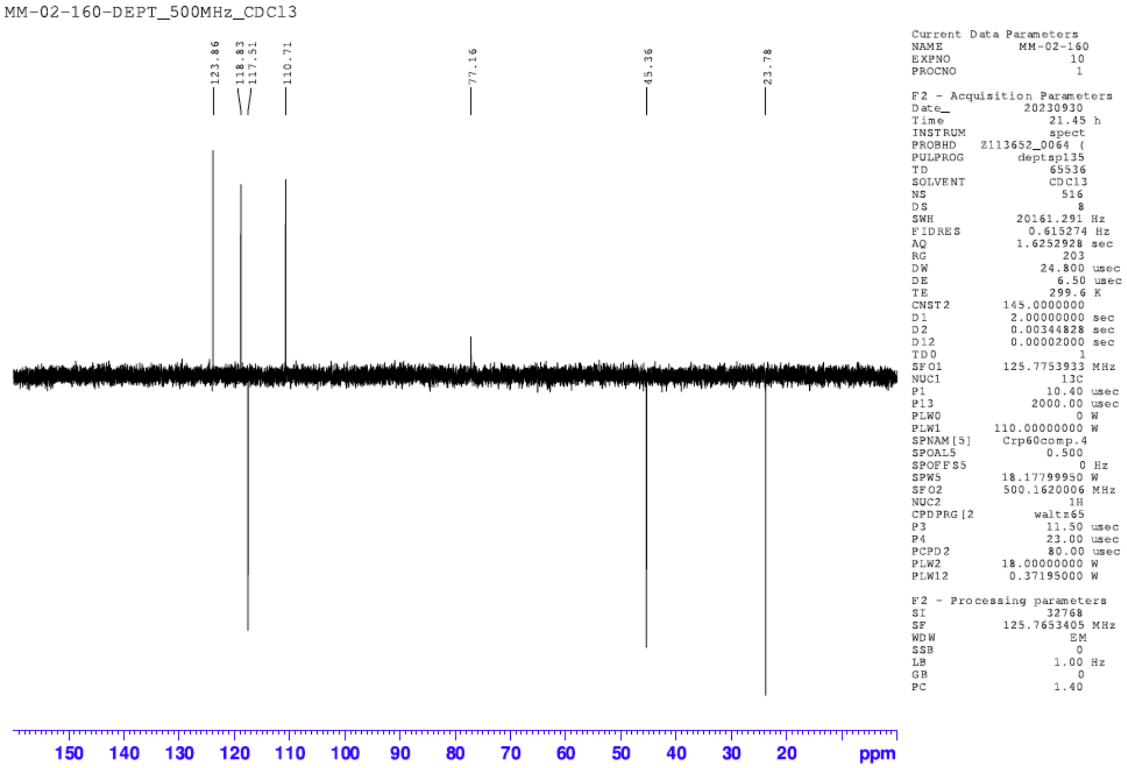
MM-02-160-DEPT_500MHz_CDCl3
123.86
118.83
117.51
110.71
77.16
45.36
23.78
150 140 130 120 110 100 90 80 70 60 50 40 30 20 ppm
Current Data Parameters
NAME MM-02-160
EXPNO 10
PROCNO 1
F2 - Acquisition Parameters
Date_ 20230930
Time 21.45 h
INSTRUM spect
PROBHD Z113652_0064 (
PULPROG deptsp135
TD 65536
SOLVENT CDCl3
NS 516
DS 8
SWH 20161.291 Hz
FIDRES 0.615274 Hz
AQ 1.6252928 sec
RG 203
DW 24.800 usec
DE 6.50 usec
TE 299.6 K
CNST2 145.0000000
D1 2.00000000 sec
D2 0.00344828 sec
D12 0.00002000 sec
TD0 1
SFO1 125.7753933 MHz
NUC1 13C
P1 10.40 usec
P13 2000.00 usec
PLW0 0 W
PLW1 110.00000000 W
SPNAM[5] Crp60comp.4
SPOAL5 0.500
SPOFFS5 0 Hz
SPW5 18.17799950 W
SFO2 500.1620006 MHz
NUC2 1H
CPDPRG[2 waltz65
P3 11.50 usec
P4 23.00 usec
PCPD2 80.00 usec
PLW2 18.00000000 W
PLW12 0.37195000 W
F2 - Processing parameters
SI 32768
SF 125.7653405 MHz
WDW EM
SSB 0
LB 1.00 Hz
GB 0
PC 1.40

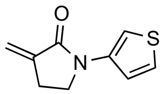
O
S
N

1d

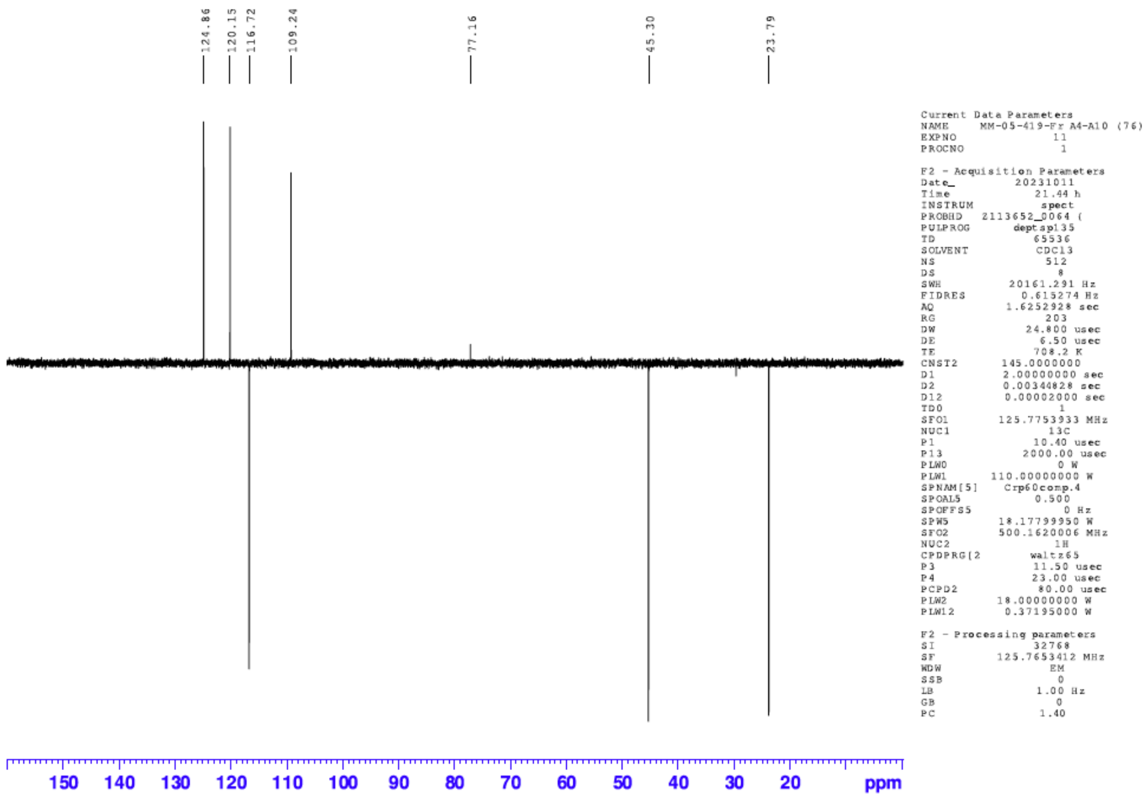
124.86
120.15
116.72
109.24
77.16
45.30
23.79
150 140 130 120 110 100 90 80 70 60 50 40 30 20 ppm
Current Data Parameters
NAME MM-05-419-Fr A4-A10 (76)
EXPNO 11
PROCNO 1
F2 - Acquisition Parameters
Date_ 20231011
Time 21.44 h
INSTRUM spect
PROBHD Z113652_0064 (
PULPROG deptsp135
TD 65536
SOLVENT CDCl3
NS 512
DS 8
SWH 20161.291 Hz
FIDRES 0.615274 Hz
AQ 1.6252928 sec
RG 203
DW 24.800 usec
DE 6.50 usec
TE 708.2 K
CNST2 145.0000000
D1 2.00000000 sec
D2 0.00344828 sec
D12 0.00002000 sec
TD0 1
SFO1 125.7753933 MHz
NUC1 13C
P1 10.40 usec
P13 2000.00 usec
PLW0 0 W
PLW1 110.00000000 W
SPNAM[5] Crp60comp.4
SPOAL5 0.500
SPOFFS5 0 Hz
SPW5 18.17799950 W
SFO2 500.1620006 MHz
NUC2 1H
CPDPRG[2 waltz65
P3 11.50 usec
P4 23.00 usec
PCPD2 80.00 usec
PLW2 18.00000000 W
PLW12 0.37195000 W
F2 - Processing parameters
SI 32768
SF 125.7653412 MHz
WDW EM
SSB 0
LB 1.00 Hz
GB 0
PC 1.40

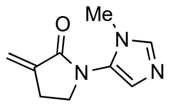

1e

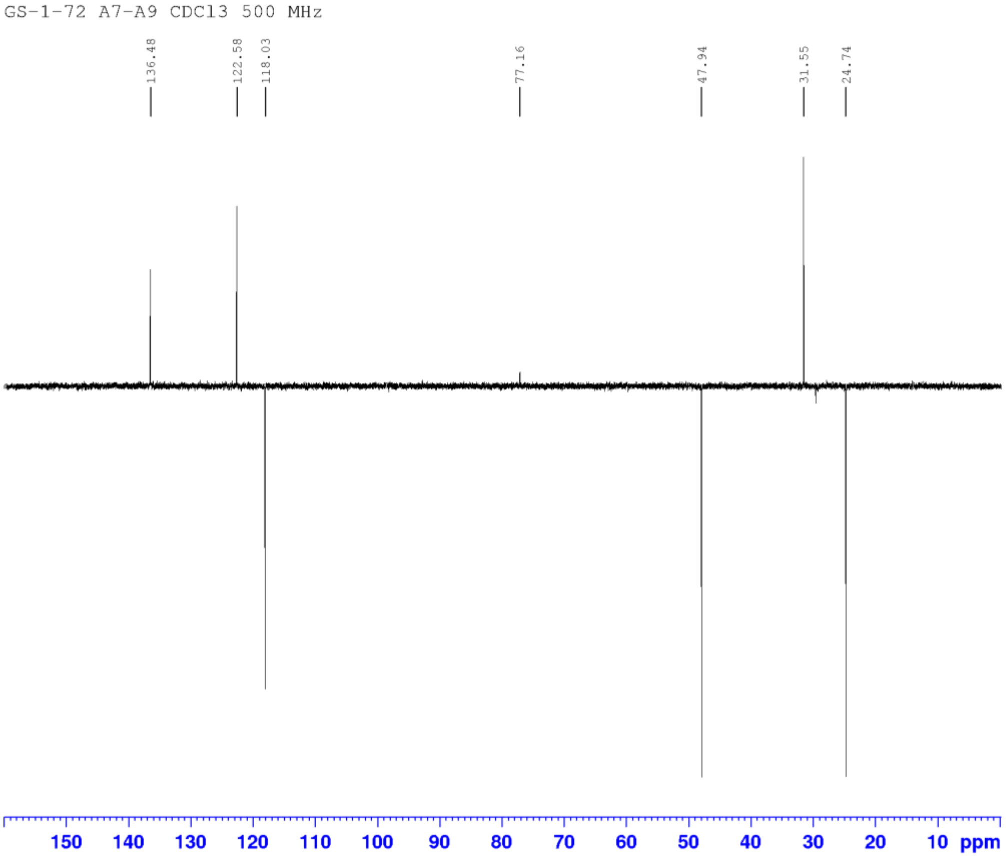

Current Data Parameters
NAME GS-1-72 A7-A9
EXPNO 12
PROCNO 1

F2 - Acquisition Parameters
Date_ 20231007
Time 8.08 h
INSTRUM spect
PROBHD Z113652_0064 (
PULPROG deptsp135
TD 65536
SOLVENT CDCl3
NS 512
DS 8
SWH 20161.291 Hz
FIDRES 0.615274 Hz
AQ 1.6252928 sec
RG 203
DW 24.800 usec
DE 6.50 usec
TE 300.6 K
CNST2 145.0000000
D1 2.00000000 sec
D2 0.00344828 sec
D12 0.00002000 sec
TD0 1
SFO1 125.7753933 MHz
NUC1 13C
P1 10.40 usec
P13 2000.00 usec
PLW0 0 W
PLW1 110.00000000 W
SPNAM[5] Crp60comp.4
SPOAL5 0.500
SPOFFS5 0 Hz
SPW5 18.17799950 W
SFO2 500.1620006 MHz
NUC2 1H
CPDPRG[2 waltz65
P3 11.50 usec
P4 23.00 usec
PCPD2 80.00 usec
PLW2 18.00000000 W
PLW12 0.37195000 W

F2 - Processing parameters
SI 32768
SF 125.7653422 MHz
WDW EM
SSB 0
LB 1.00 Hz
GB 0
PC 1.40

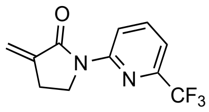

1f

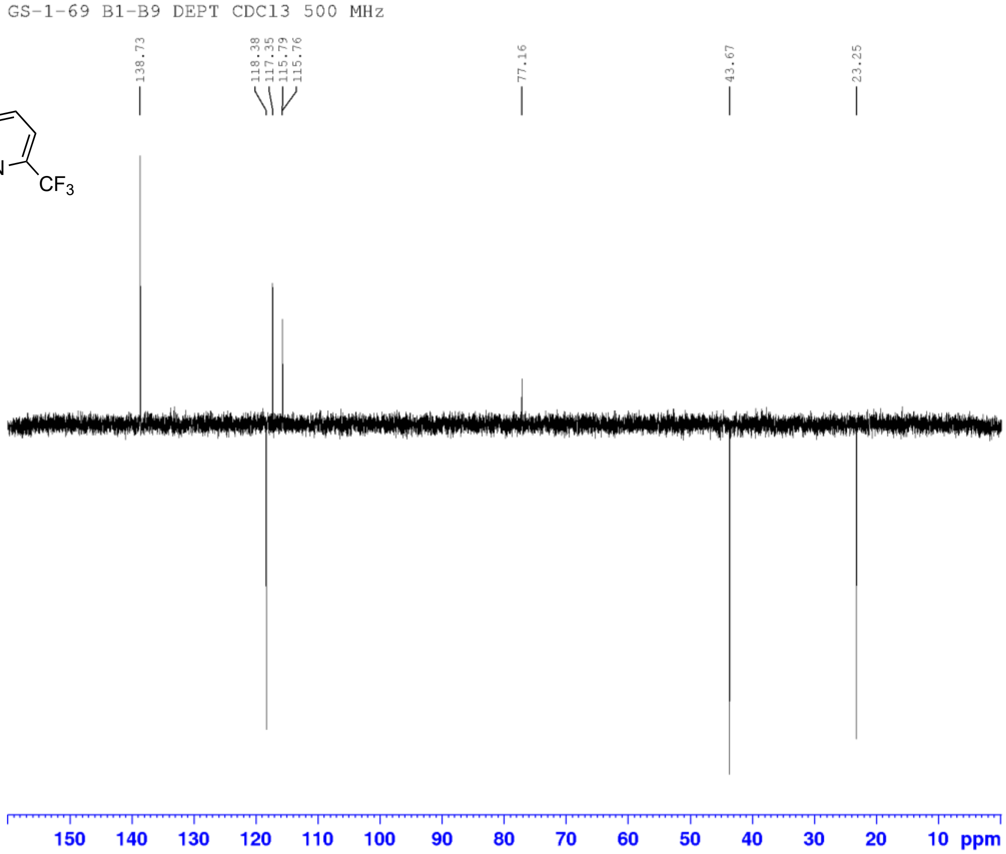

Current Data Parameters
NAME GS-1-69 B1-B9 DEPT
EXPNO 10
PROCNO 1

F2 - Acquisition Parameters
Date_ 20231003
Time 1.04 h
INSTRUM spect
PROBHD Z113652_0064 (
PULPROG deptsp135
TD 65536
SOLVENT CDCl3
NS 512
DS 8
SWH 20161.291 Hz
FIDRES 0.615274 Hz
AQ 1.6252928 sec
RG 203
DW 24.800 usec
DE 6.50 usec
TE 300.0 K
CNST2 145.0000000
D1 2.00000000 sec
D2 0.00344828 sec
D12 0.00002000 sec
TD0 1
SFO1 125.7753933 MHz
NUC1 13C
P1 10.40 usec
P13 2000.00 usec
PLW0 0 W
PLW1 110.00000000 W
SPNAM[5] Crp60comp.4
SPOAL5 0.500
SPOFFS5 0 Hz
SPW5 18.17799950 W
SFO2 500.1620006 MHz
NUC2 1H
CPDPRG[2 waltz65
P3 11.50 usec
P4 23.00 usec
PCPD2 80.00 usec
PLW2 18.00000000 W
PLW12 0.37195000 W

F2 - Processing parameters
SI 32768
SF 125.7653397 MHz
WDW EM
SSB 0
LB 1.00 Hz
GB 0
PC 1.40

GS-1-63 A11-B10 DEPT CDCl3 500 MHZ

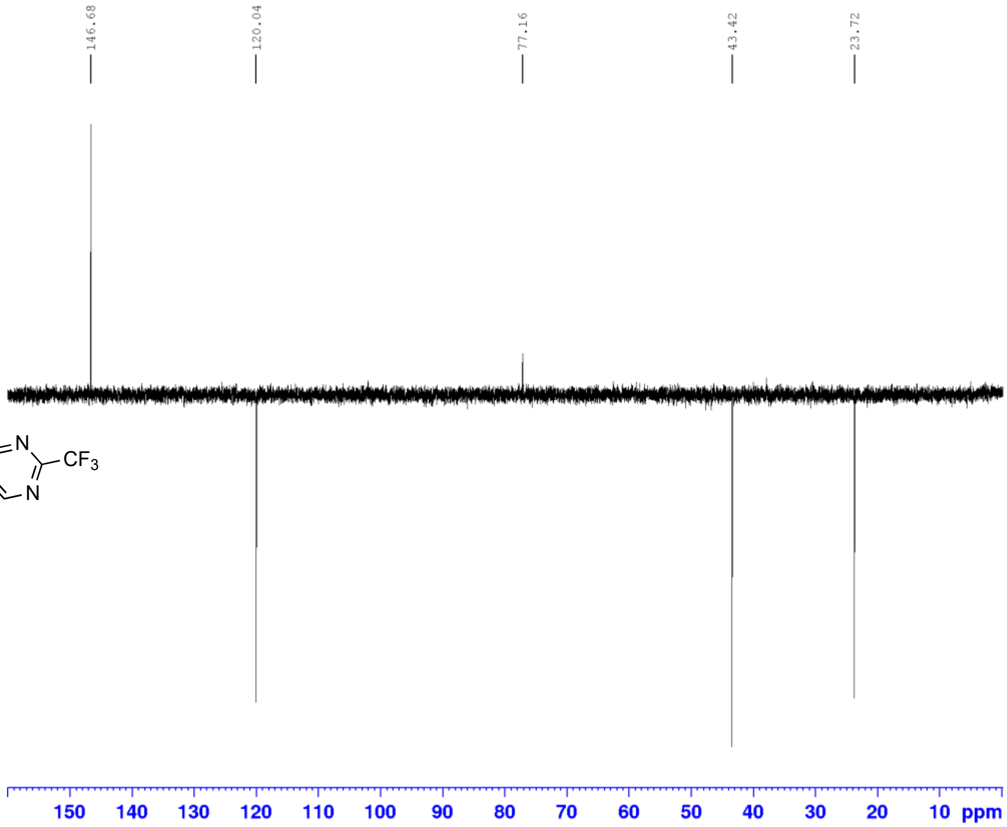

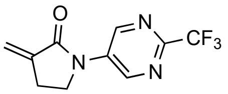

1g

```
Current Data Parameters
NAME      GS-1-63 A11-B10 DEPT
EXPNO               11
PROCNO               1

F2 - Acquisition Parameters
Date_         20231002
Time             23.42 h
INSTRUM          spect
PROBHD   Z113652_0064 (
PULPROG      deptsp135
TD               65536
SOLVENT          CDCl3
NS                 512
DS                   8
SWH          20161.291 Hz
FIDRES        0.615274 Hz
AQ           1.6252928 sec
RG                 203
DW              24.800 usec
DE                6.50 usec
TE               300.1 K
CNST2      145.0000000
D1          2.00000000 sec
D2          0.00344828 sec
D12         0.00002000 sec
TD0                  1
SFO1       125.7753933 MHz
NUC1               13C
P1               10.40 usec
P13            2000.00 usec
PLW0                 0 W
PLW1      110.00000000 W
SPNAM[5]   Crp60comp.4
SPOAL5           0.500
SPOFFS5              0 Hz
SPW5       18.17799950 W
SFO2       500.1620006 MHz
NUC2                1H
CPDPRG[2       waltz65
P3               11.50 usec
P4               23.00 usec
PCPD2            80.00 usec
PLW2       18.00000000 W
PLW12       0.37195000 W

F2 - Processing parameters
SI               32768
SF         125.7653398 MHz
WDW                 EM
SSB                  0
LB                1.00 Hz
GB                   0
PC                1.40
```

GS-1-64 B6-C10 DEPT CDCl3 500 MHz

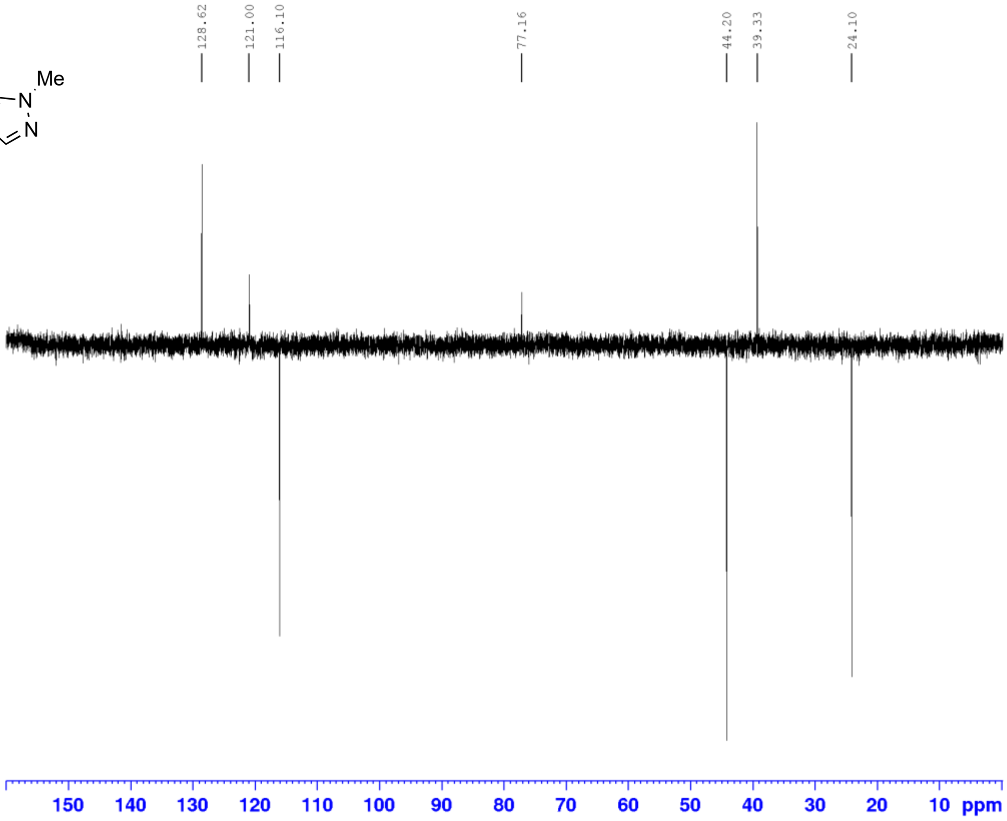

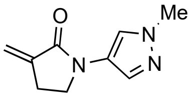

1h

```
Current Data Parameters
NAME      GS-1-64 B6-C10 DEPT
EXPNO               10
PROCNO               1

F2 - Acquisition Parameters
Date_         20231003
Time              0.24 h
INSTRUM          spect
PROBHD   Z113652_0064 (
PULPROG      deptsp135
TD               65536
SOLVENT          CDCl3
NS                 512
DS                   8
SWH          20161.291 Hz
FIDRES        0.615274 Hz
AQ           1.6252928 sec
RG                 203
DW              24.800 usec
DE                6.50 usec
TE               300.1 K
CNST2      145.0000000
D1          2.00000000 sec
D2          0.00344828 sec
D12         0.00002000 sec
TD0                  1
SFO1       125.7753933 MHz
NUC1               13C
P1               10.40 usec
P13            2000.00 usec
PLW0                 0 W
PLW1      110.00000000 W
SPNAM[5]   Crp60comp.4
SPOAL5           0.500
SPOFFS5              0 Hz
SPW5       18.17799950 W
SFO2       500.1620006 MHz
NUC2                1H
CPDPRG[2       waltz65
P3               11.50 usec
P4               23.00 usec
PCPD2            80.00 usec
PLW2       18.00000000 W
PLW12       0.37195000 W

F2 - Processing parameters
SI               32768
SF         125.7653403 MHz
WDW                 EM
SSB                  0
LB                1.00 Hz
GB                   0
PC                1.40
```

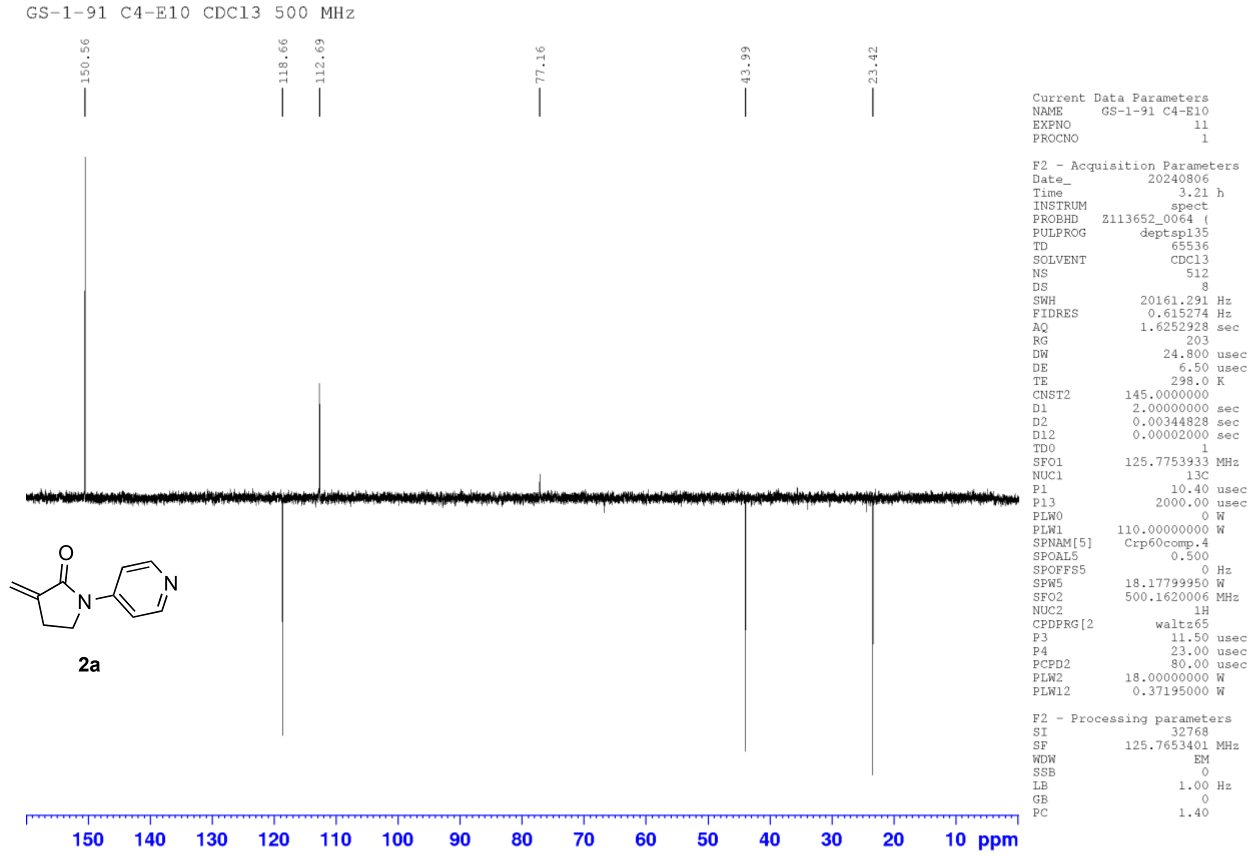
GS-1-91 C4-E10 CDCl3 500 MHz
150.56
118.66
112.69
77.16
43.99
23.42
O
N
N
Current Data Parameters
NAME GS-1-91 C4-E10
EXPNO 11
PROCNO 1
F2 - Acquisition Parameters
Date_ 20240806
Time 3.21 h
INSTRUM spect
PROBHD Z113652_0064 (
PULPROG deptsp135
TD 65536
SOLVENT CDCl3
NS 512
DS 8
SWH 20161.291 Hz
FIDRES 0.615274 Hz
AQ 1.6252928 sec
RG 203
DW 24.800 usec
DE 6.50 usec
TE 298.0 K
CNST2 145.0000000
D1 2.00000000 sec
D2 0.00344828 sec
D12 0.00002000 sec
TD0 1
SFO1 125.7753933 MHz
NUC1 13C
P1 10.40 usec
P13 2000.00 usec
PLW0 0 W
PLW1 110.00000000 W
SPNAM[5] Crp60comp.4
SPOAL5 0.500
SPOFFS5 0 Hz
SPW5 18.17799950 W
SFO2 500.1620006 MHz
NUC2 1H
CPDPRG[2 waltz65
P3 11.50 usec
P4 23.00 usec
PCPD2 80.00 usec
PLW2 18.00000000 W
PLW12 0.37195000 W
F2 - Processing parameters
SI 32768
SF 125.7653401 MHz
WDW EM
SSB 0
LB 1.00 Hz
GB 0
PC 1.40
150 140 130 120 110 100 90 80 70 60 50 40 30 20 10 ppm

2a

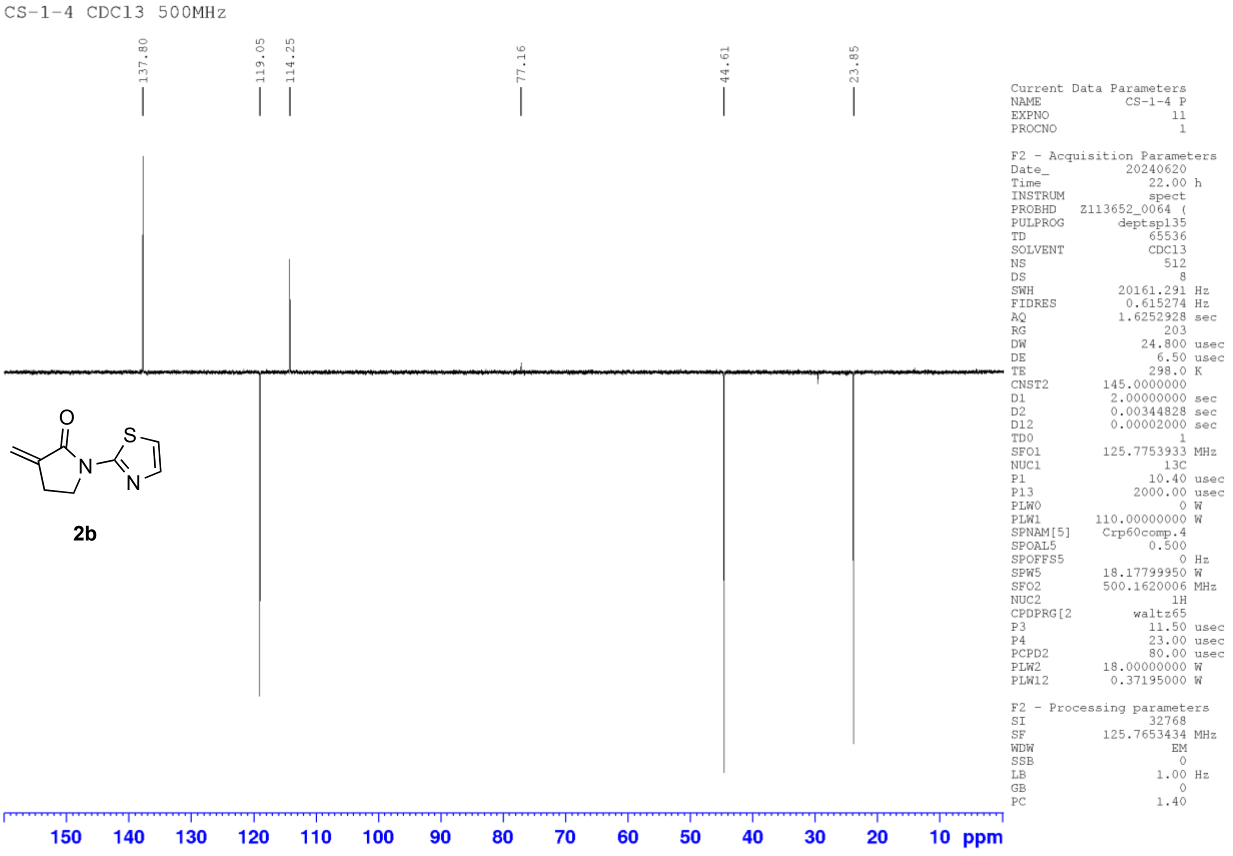
CS-1-4 CDCl3 500MHz
137.80
119.05
114.25
77.16
44.61
23.85
O
N
S
N
Current Data Parameters
NAME CS-1-4 P
EXPNO 11
PROCNO 1
F2 - Acquisition Parameters
Date_ 20240620
Time 22.00 h
INSTRUM spect
PROBHD Z113652_0064 (
PULPROG deptsp135
TD 65536
SOLVENT CDCl3
NS 512
DS 8
SWH 20161.291 Hz
FIDRES 0.615274 Hz
AQ 1.6252928 sec
RG 203
DW 24.800 usec
DE 6.50 usec
TE 298.0 K
CNST2 145.0000000
D1 2.00000000 sec
D2 0.00344828 sec
D12 0.00002000 sec
TD0 1
SFO1 125.7753933 MHz
NUC1 13C
P1 10.40 usec
P13 2000.00 usec
PLW0 0 W
PLW1 110.00000000 W
SPNAM[5] Crp60comp.4
SPOAL5 0.500
SPOFFS5 0 Hz
SPW5 18.17799950 W
SFO2 500.1620006 MHz
NUC2 1H
CPDPRG[2 waltz65
P3 11.50 usec
P4 23.00 usec
PCPD2 80.00 usec
PLW2 18.00000000 W
PLW12 0.37195000 W
F2 - Processing parameters
SI 32768
SF 125.7653434 MHz
WDW EM
SSB 0
LB 1.00 Hz
GB 0
PC 1.40
150 140 130 120 110 100 90 80 70 60 50 40 30 20 10 ppm

2b

CS-1-1 A7-B3 CDCl3 500MHz

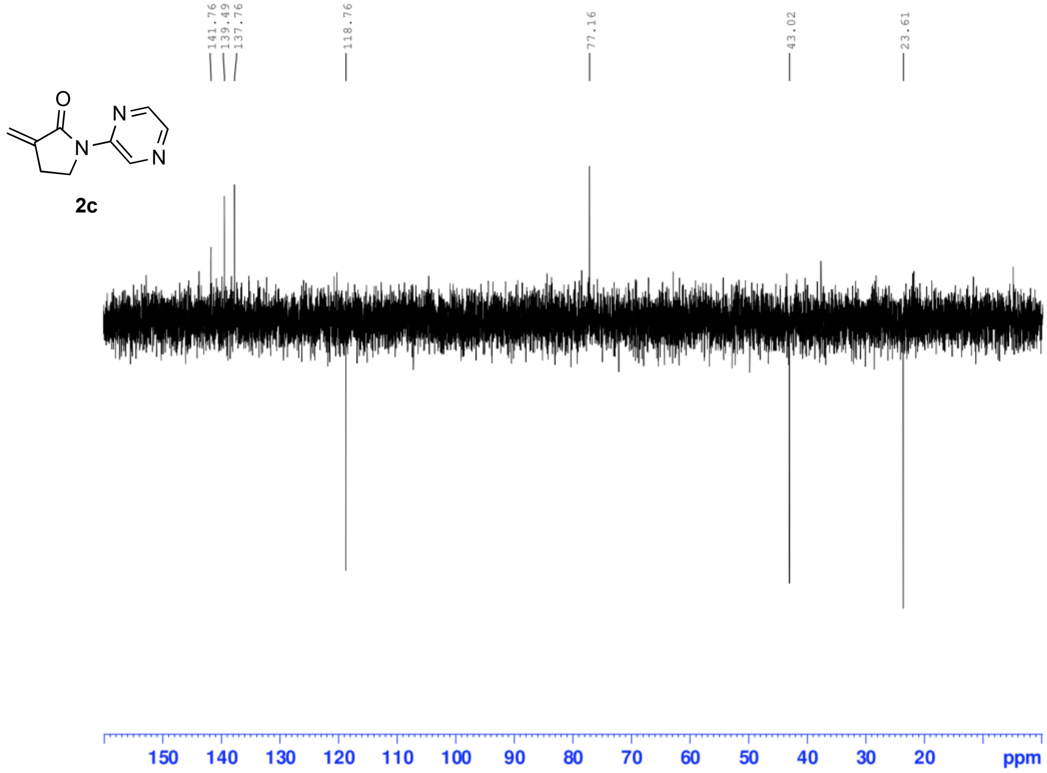

2c

Current Data Parameters
NAME CS-1-1 A7-B3
EXPNO 20
PROCNO 1

F2 - Acquisition Parameters
Date_ 20240607
Time 12.18 h
INSTRUM spect
PROBHD Z113652_0064 (
PULPROG deptsp135
TD 65536
SOLVENT CDCl3
NS 512
DS 8
SWH 20161.291 Hz
FIDRES 0.615274 Hz
AQ 1.6252928 sec
RG 203
DW 24.800 usec
DE 6.50 usec
TE 298.0 K
CNST2 145.0000000
D1 2.00000000 sec
D2 0.00344828 sec
D12 0.00002000 sec
TD0 1
SFO1 125.7753933 MHz
NUC1 13C
P1 10.40 usec
P13 2000.00 usec
PLW0 0 W
PLW1 110.00000000 W
SPNAM[5] Crp60comp.4
SPOAL5 0.500
SPOFFS5 0 Hz
SPW5 18.17799950 W
SFO2 500.1620006 MHz
NUC2 1H
CPDPRG[2 waltz65
P3 11.50 usec
P4 23.00 usec
PCPD2 80.00 usec
PLW2 18.00000000 W
PLW12 0.37195000 W

F2 - Processing parameters
SI 32768
SF 125.7653392 MHz
WDW EM
SSB 0
LB 1.00 Hz
GB 0
PC 1.40

GS-1-76 11.19.24 DEPT CDCl3 500 MHz

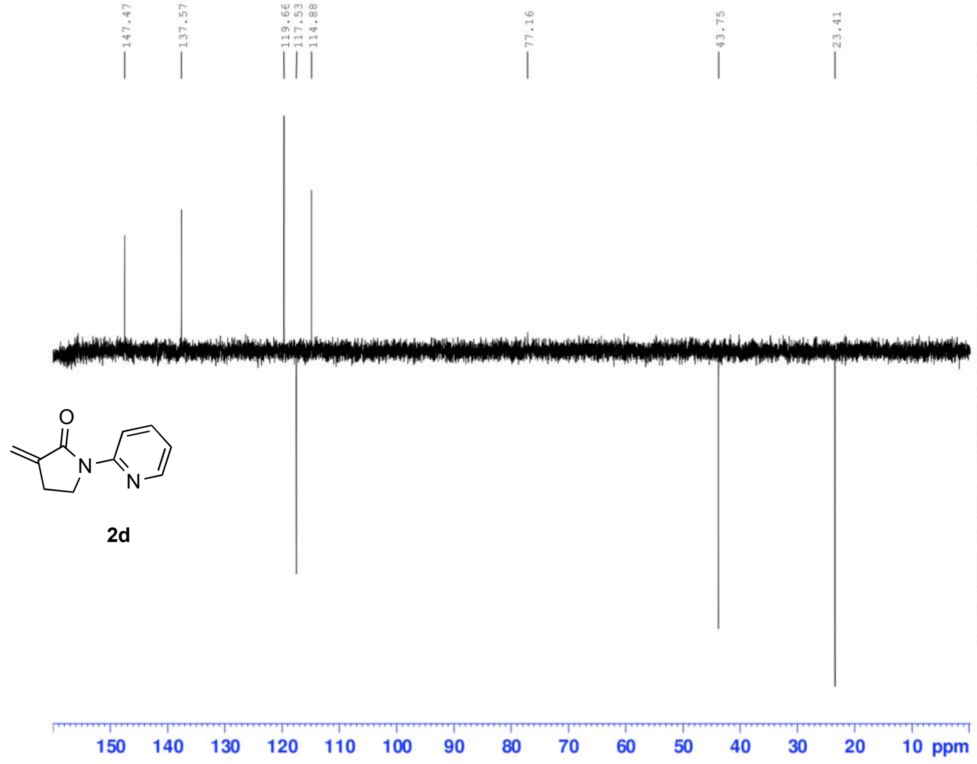

2d

Current Data Parameters
NAME GS-1-76 11.19.24 DEP
EXPNO 10
PROCNO 1

F2 - Acquisition Parameters
Date_ 20241119
Time 17.20 h
INSTRUM spect
PROBHD Z113652_0064 (
PULPROG deptsp135
TD 65536
SOLVENT CDCl3
NS 512
DS 8
SWH 20161.291 Hz
FIDRES 0.615274 Hz
AQ 1.6252928 sec
RG 203
DW 24.800 usec
DE 6.50 usec
TE 298.0 K
CNST2 145.0000000
D1 2.00000000 sec
D2 0.00344828 sec
D12 0.00002000 sec
TD0 1
SFO1 125.7753933 MHz
NUC1 13C
P1 10.40 usec
P13 2000.00 usec
PLW0 0 W
PLW1 110.00000000 W
SPNAM[5] Crp60comp.4
SPOAL5 0.500
SPOFFS5 0 Hz
SPW5 18.17799950 W
SFO2 500.1620006 MHz
NUC2 1H
CPDPRG[2 waltz65
P3 11.50 usec
P4 23.00 usec
PCPD2 80.00 usec
PLW2 18.00000000 W
PLW12 0.37195000 W

F2 - Processing parameters
SI 32768
SF 125.7653412 MHz
WDW EM
SSB 0
LB 1.00 Hz
GB 0
PC 1.40

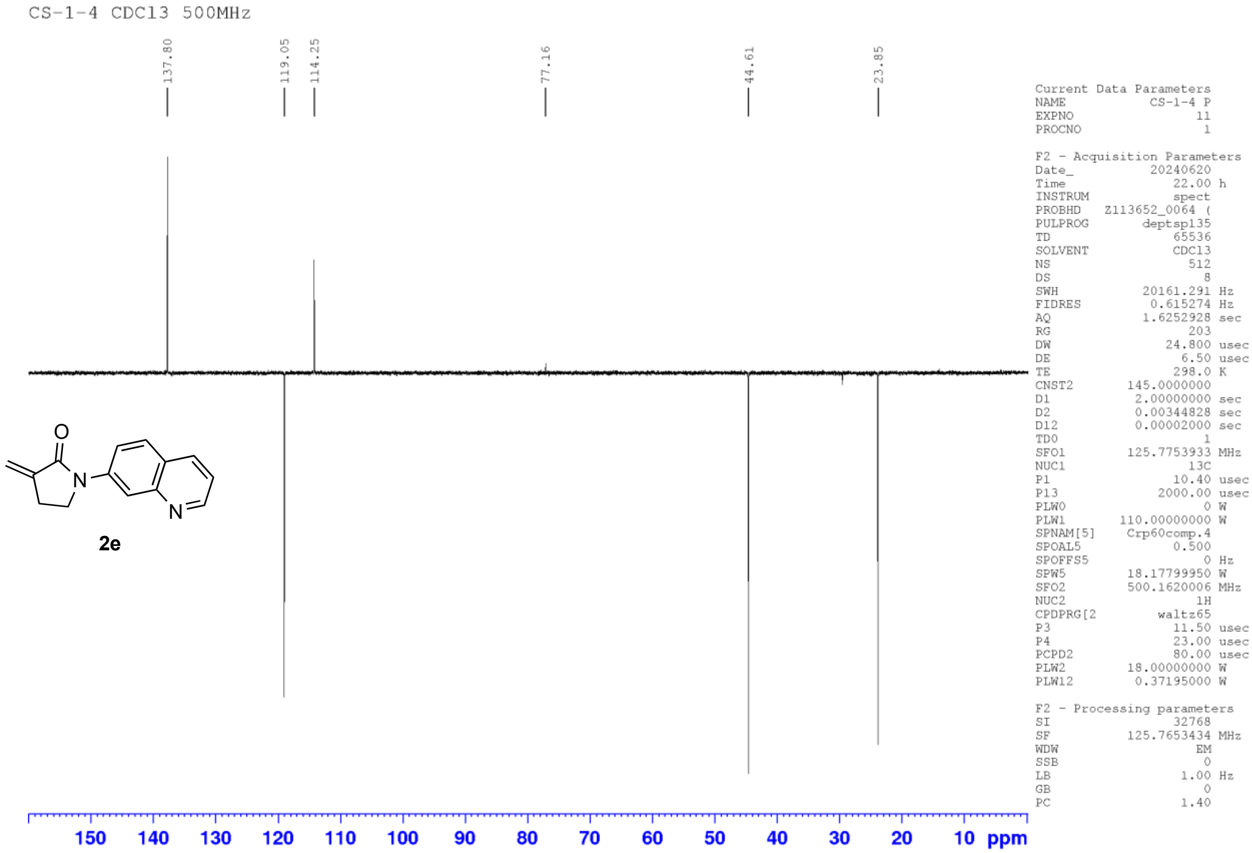
CS-1-4 CDCl3 500MHz
137.80
119.05
114.25
77.16
44.61
23.85
Current Data Parameters
NAME CS-1-4 P
EXPNO 11
PROCNO 1
F2 - Acquisition Parameters
Date_ 20240620
Time 22.00 h
INSTRUM spect
PROBHD Z113652_0064 (
PULPROG deptsp135
TD 65536
SOLVENT CDCl3
NS 512
DS 8
SWH 20161.291 Hz
FIDRES 0.615274 Hz
AQ 1.6252928 sec
RG 203
DW 24.800 usec
DE 6.50 usec
TE 298.0 K
CNST2 145.0000000
D1 2.00000000 sec
D2 0.00344828 sec
D12 0.00002000 sec
TD0 1
SFO1 125.7753933 MHz
NUC1 13C
P1 10.40 usec
P13 2000.00 usec
PLW0 0 W
PLW1 110.00000000 W
SPNAM[5] Crp60comp.4
SPOAL5 0.500
SPOFFS5 0 Hz
SPW5 18.17799950 W
SFO2 500.1620006 MHz
NUC2 1H
CPDPRG[2 waltz65
P3 11.50 usec
P4 23.00 usec
PCPD2 80.00 usec
PLW2 18.00000000 W
PLW12 0.37195000 W
F2 - Processing parameters
SI 32768
SF 125.7653434 MHz
WDW EM
SSB 0
LB 1.00 Hz
GB 0
PC 1.40
2e
150 140 130 120 110 100 90 80 70 60 50 40 30 20 10 ppm

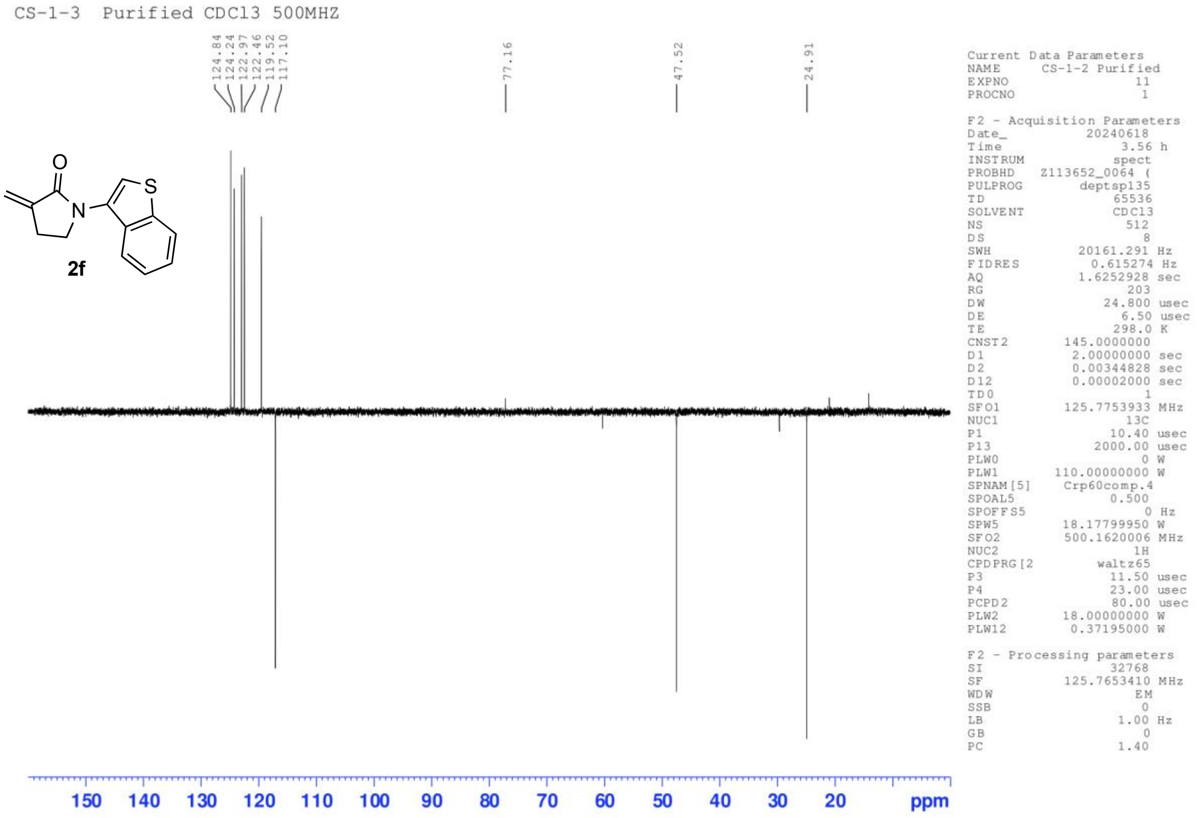
CS-1-3 Purified CDCl3 500MHZ
124.84
124.24
122.97
122.46
119.52
117.10
77.16
47.52
24.91
Current Data Parameters
NAME CS-1-2 Purified
EXPNO 11
PROCNO 1
F2 - Acquisition Parameters
Date_ 20240618
Time 3.56 h
INSTRUM spect
PROBHD Z113652_0064 (
PULPROG deptsp135
TD 65536
SOLVENT CDCl3
NS 512
DS 8
SWH 20161.291 Hz
FIDRES 0.615274 Hz
AQ 1.6252928 sec
RG 203
DW 24.800 usec
DE 6.50 usec
TE 298.0 K
CNST2 145.0000000
D1 2.00000000 sec
D2 0.00344828 sec
D12 0.00002000 sec
TD0 1
SFO1 125.7753933 MHz
NUC1 13C
P1 10.40 usec
P13 2000.00 usec
PLW0 0 W
PLW1 110.00000000 W
SPNAM[5] Crp60comp.4
SPOAL5 0.500
SPOFFS5 0 Hz
SPW5 18.17799950 W
SFO2 500.1620006 MHz
NUC2 1H
CPDPRG[2 waltz65
P3 11.50 usec
P4 23.00 usec
PCPD2 80.00 usec
PLW2 18.00000000 W
PLW12 0.37195000 W
F2 - Processing parameters
SI 32768
SF 125.7653410 MHz
WDW EM
SSB 0
LB 1.00 Hz
GB 0
PC 1.40
2f
150 140 130 120 110 100 90 80 70 60 50 40 30 20 ppm

CS-1-10 DEPT CDCl3 500 MHz

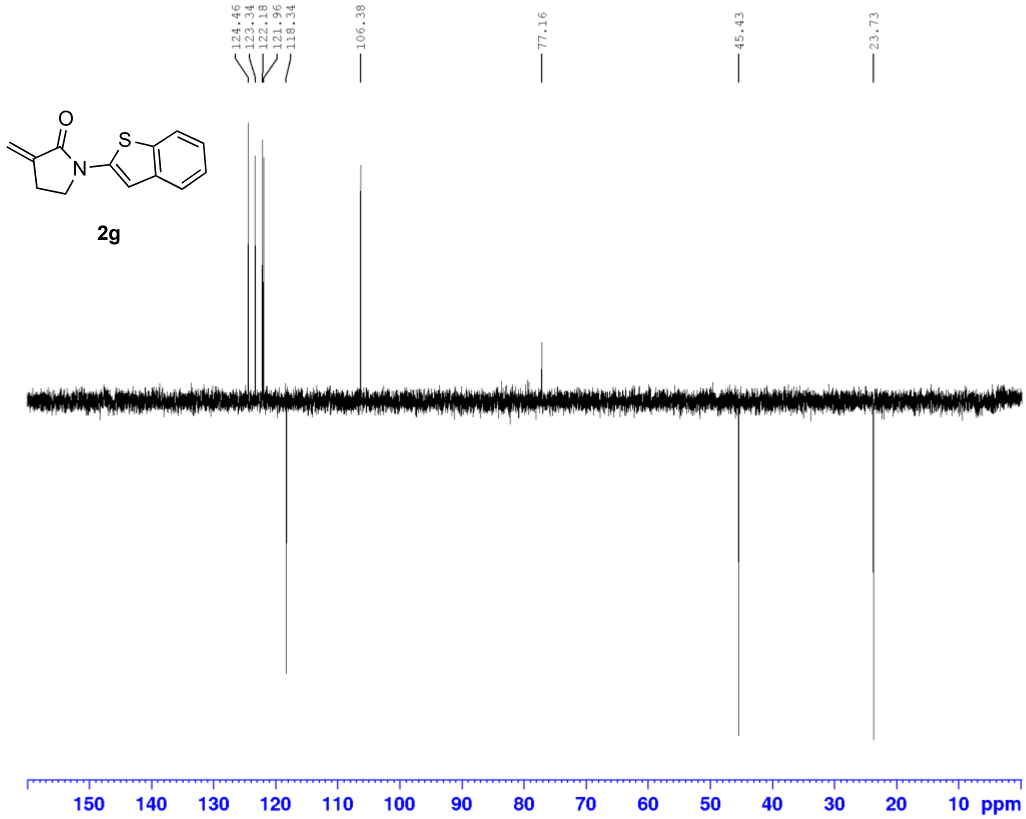

Current Data Parameters
NAME CS-1-10 DEPT
EXPNO 10
PROCNO 1

F2 - Acquisition Parameters
Date_ 20240813
Time 10.18 h
INSTRUM spect
PROBHD Z113652_0064 (
PULPROG deptsp135
TD 65536
SOLVENT CDCl3
NS 512
DS 8
SWH 20161.291 Hz
FIDRES 0.615274 Hz
AQ 1.6252928 sec
RG 203
DW 24.800 usec
DE 6.50 usec
TE 298.0 K
CNST2 145.0000000
D1 2.00000000 sec
D2 0.00344828 sec
D12 0.00002000 sec
TD0 1
SFO1 125.7753933 MHz
NUC1 13C
P1 10.40 usec
P13 2000.00 usec
PLW0 0 W
PLW1 110.00000000 W
SPNAM[5] Crp60comp.4
SPOAL5 0.500
SPOFFS5 0 Hz
SPW5 18.17799950 W
SFO2 500.1620006 MHz
NUC2 1H
CPDPRG[2 waltz65
P3 11.50 usec
P4 23.00 usec
PCPD2 80.00 usec
PLW2 18.00000000 W
PLW12 0.37195000 W

F2 - Processing parameters
SI 32768
SF 125.7653391 MHz
WDW EM
SSB 0
LB 1.00 Hz
GB 0
PC 1.40

GS-1-81 D3-E11 CDCl3 500MHz

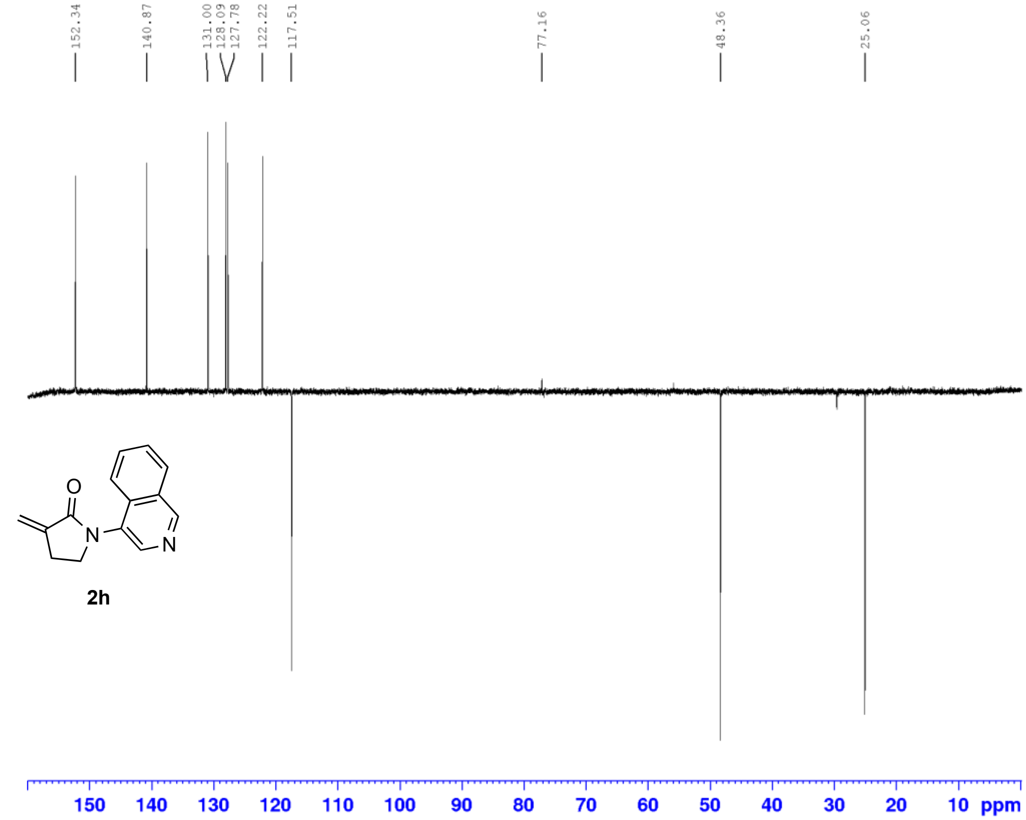

Current Data Parameters
NAME GS-1-81 D3-E11
EXPNO 11
PROCNO 1

F2 - Acquisition Parameters
Date_ 20240626
Time 0.45 h
INSTRUM spect
PROBHD Z113652_0064 (
PULPROG deptsp135
TD 65536
SOLVENT CDCl3
NS 512
DS 8
SWH 20161.291 Hz
FIDRES 0.615274 Hz
AQ 1.6252928 sec
RG 203
DW 24.800 usec
DE 6.50 usec
TE 298.0 K
CNST2 145.0000000
D1 2.00000000 sec
D2 0.00344828 sec
D12 0.00002000 sec
TD0 1
SFO1 125.7753933 MHz
NUC1 13C
P1 10.40 usec
P13 2000.00 usec
PLW0 0 W
PLW1 110.00000000 W
SPNAM[5] Crp60comp.4
SPOAL5 0.500
SPOFFS5 0 Hz
SPW5 18.17799950 W
SFO2 500.1620006 MHz
NUC2 1H
CPDPRG[2 waltz65
P3 11.50 usec
P4 23.00 usec
PCPD2 80.00 usec
PLW2 18.00000000 W
PLW12 0.37195000 W

F2 - Processing parameters
SI 32768
SF 125.7653422 MHz
WDW EM
SSB 0
LB 1.00 Hz
GB 0
PC 1.40

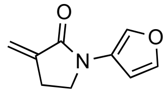

2i

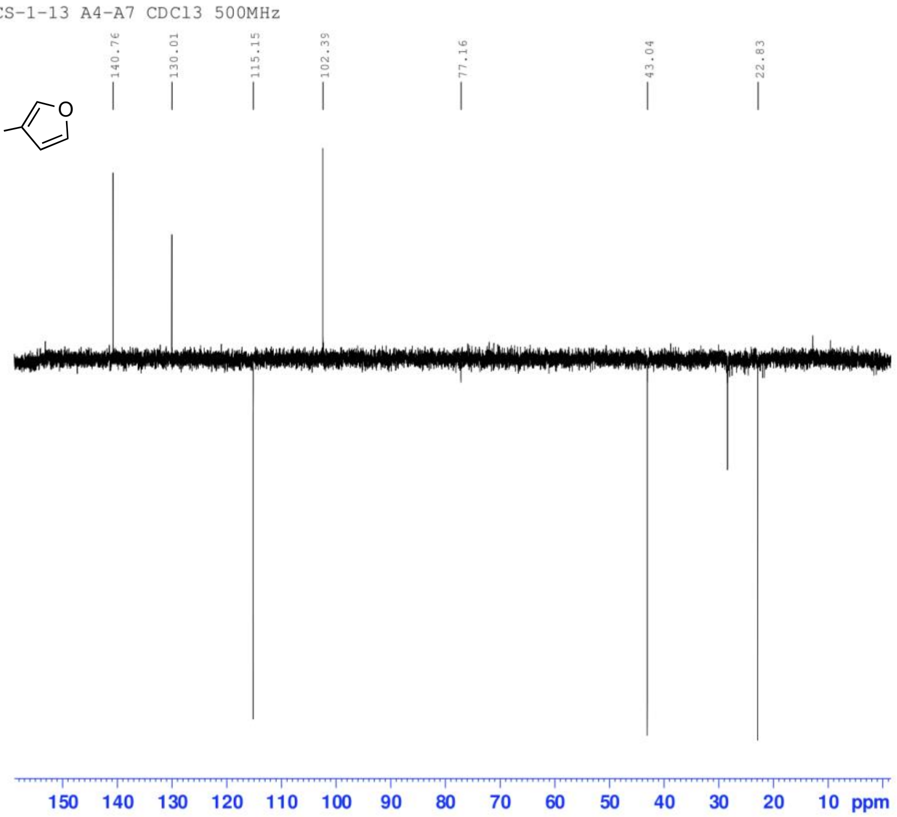


```
Current Data Parameters
NAME        CS-1-13 A4-A7
EXPNO                  11
PROCNO                  1

F2 - Acquisition Parameters
Date_            20240730
Time               3.45 h
INSTRUM             spect
PROBHD   Z113652_0064 (
PULPROG        deptsp135
TD                  65536
SOLVENT             CDCl3
NS                    512
DS                      8
SWH             20161.291 Hz
FIDRES           0.615274 Hz
AQ              1.6252928 sec
RG                    203
DW                 24.800 usec
DE                   6.50 usec
TE                  298.0 K
CNST2         145.0000000
D1             2.00000000 sec
D2             0.00344828 sec
D12            0.00002000 sec
TD0                     1
SFO1          125.7753933 MHz
NUC1                  13C
P1                  10.40 usec
P13               2000.00 usec
PLW0                    0 W
PLW1         110.00000000 W
SPNAM[5]     Crp60comp.4
SPOAL5              0.500
SPOFFS5                 0 Hz
SPW5          18.17799950 W
SFO2          500.1620006 MHz
NUC2                   1H
CPDPRG[2          waltz65
P3                  11.50 usec
P4                  23.00 usec
PCPD2               80.00 usec
PLW2          18.00000000 W
PLW12          0.37195000 W

F2 - Processing parameters
SI                  32768
SF            125.7654996 MHz
WDW                    EM
SSB                     0
LB                   1.00 Hz
GB                      0
PC                   1.40
```

COMPUTATIONAL DETAILS AND ADDITIONAL COMPUTATIONAL RESULTS

All DFT calculations were performed using Gaussian 16.[2] Molecular geometries of **1a-1h**, **2a-2c**, methylthiolate anion, and transition states **TS1a-TS1h** were optimized using the M06-2X functional[3] with the 6-31+G(d) basis set and SMD solvation model[4] in water. Vibrational frequency calculations were performed for all the stationary points to confirm if each optimized structure was a local minimum or a transition state structure. Gibbs free energies were calculated at 310.15 K using Truhlar's quasi-harmonic approximations[5] using the GoodVibes package[6] (version 3.2) with 100 cm^{-1} as the frequency cutoff in entropy calculations. The M06-2X functional with the 6-311+G(d,p) basis set was used for single-point energy calculations in water with the SMD solvation model.

Conformational sampling for **1a-1h**, **2a-2c**, and **TS1a-TS1h** was performed using the Conformer-Rotamer Ensemble Sampling Tool (CREST) program[7] (version 2.12) that uses the semiempirical tight-binding method GFN2-xTB[8] (xTB version 6.6.0) to perform metadynamics sampling with genetic z-matrix crossing (iMTD-GC). In the CREST/GFN2-xTB conformational sampling of each structure, the conformers were filtered using a 0.5 Å root-mean-square-deviation (RMSD) threshold and an energy window of 8 kcal/mol. For each transition state, the forming S-C bond was restrained at a distance of 2.45 Å with a force constant of 0.05 Hartree/$Bohr^2$. The conformational sampling of intermediate and transition state structures was performed using the analytical linearized Poisson-Boltzmann (ALPB) model[9] in water. Low-energy conformers of intermediates were fully optimized at the indicated DFT level, and the lowest-energy conformer obtained from DFT calculations for each substrate was used for the transition state structure. For the transition state structures, several low-energy conformers demonstrating *syn* addition of the methyl thiolate nucleophile to the *N*-heteroaryl *α*-methylene-*γ*-lactam[10] were chosen for geometry optimization using DFT, and the lowest-energy conformer obtained from DFT calculations for each transition state was reported.

Natural population analysis (NPA) was performed using NBO (version 3.1) embedded in Gaussian.[11]

File format conversion was performed using OpenBabel[12] (version 3.1.1). Structures were visualized using CYLView[13] (version 1.0).

Thiol-Michael transition state analysis for the addition of methyl thiolate to 1a-1h

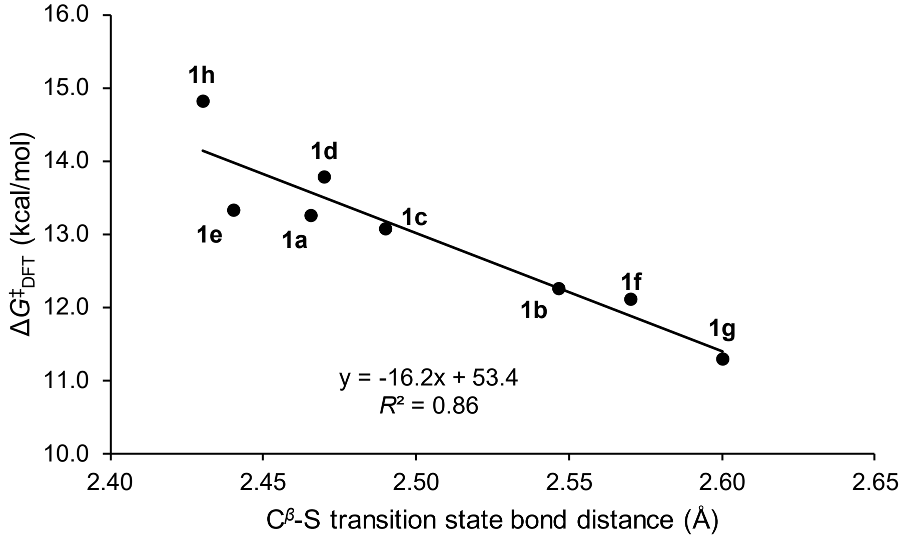

Figure S21. Correlation of $\Delta G^{\ddagger}_{DFT}$ (kcal/mol) with transition state bond distance (Å) between C^{β} of *N*-heteroaryl *α*-methylene-*γ*-lactam and sulfur atom of methyl thiolate.

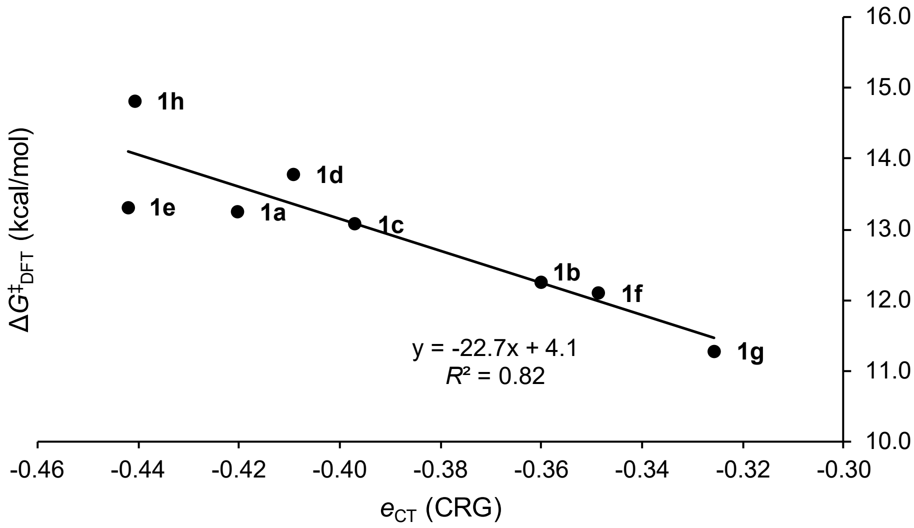

Figure S22. Correlation of $\Delta G^{\ddagger}_{DFT}$ (kcal/mol) with total charge transfer to the CRG in the transition state.

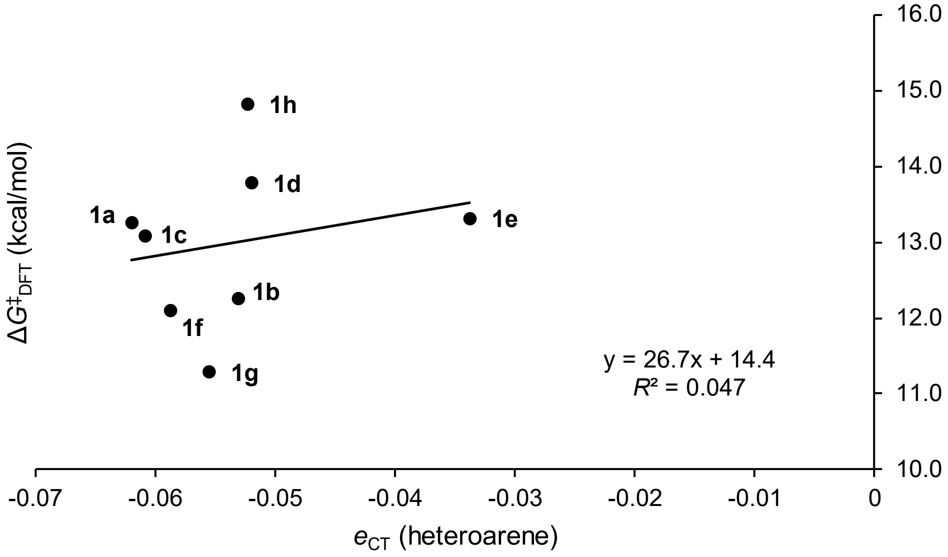

Figure S23. Correlation of $\Delta G^{\ddagger}_{DFT}$ (kcal/mol) with charge transfer to the heteroaryl group on the CRG in the transition state.

Linear regression models for predicting $\Delta G^{\ddagger}_{DFT}$

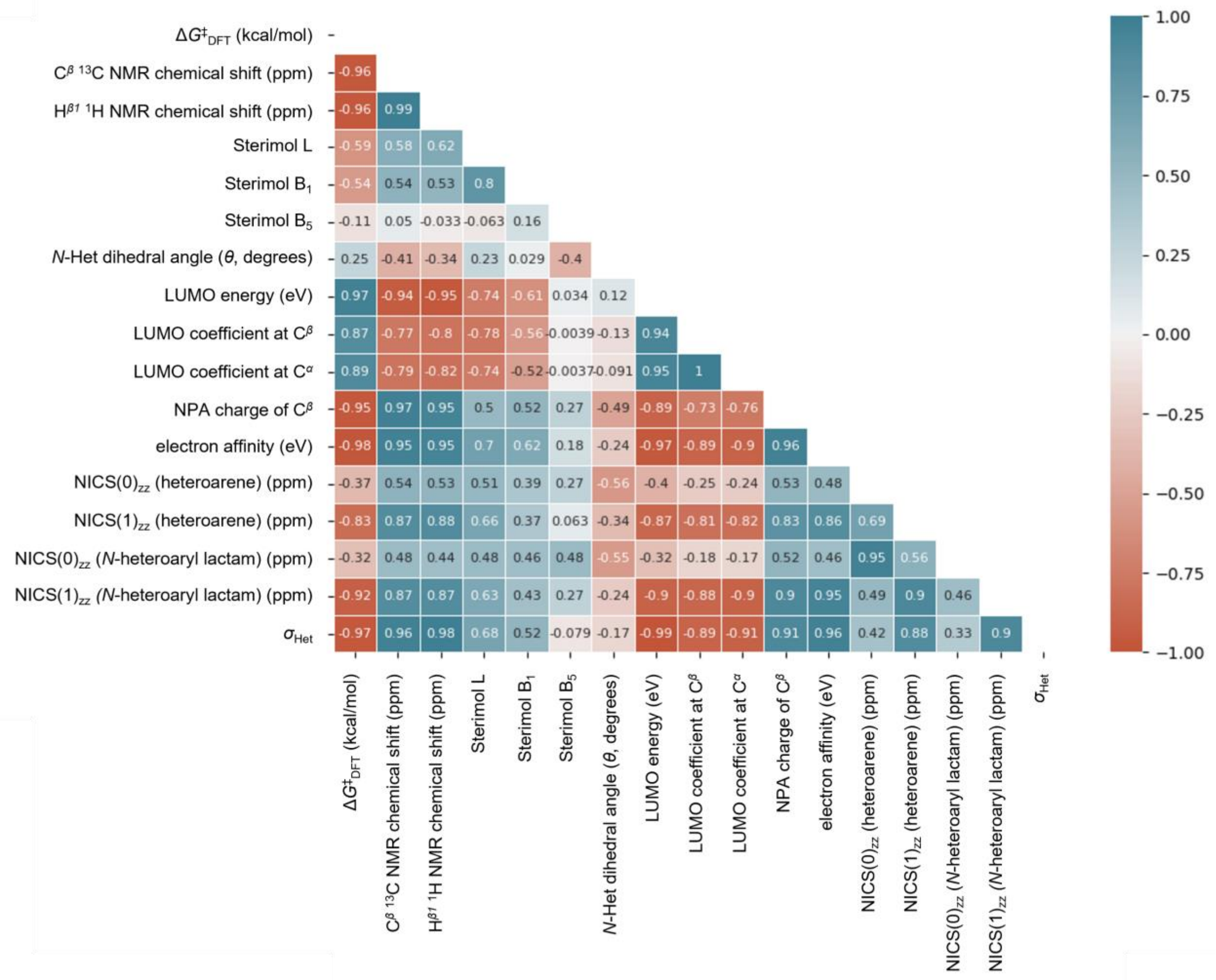

Figure S24. Correlation matrix with Pearson coefficients for calculated and experimental parameters.

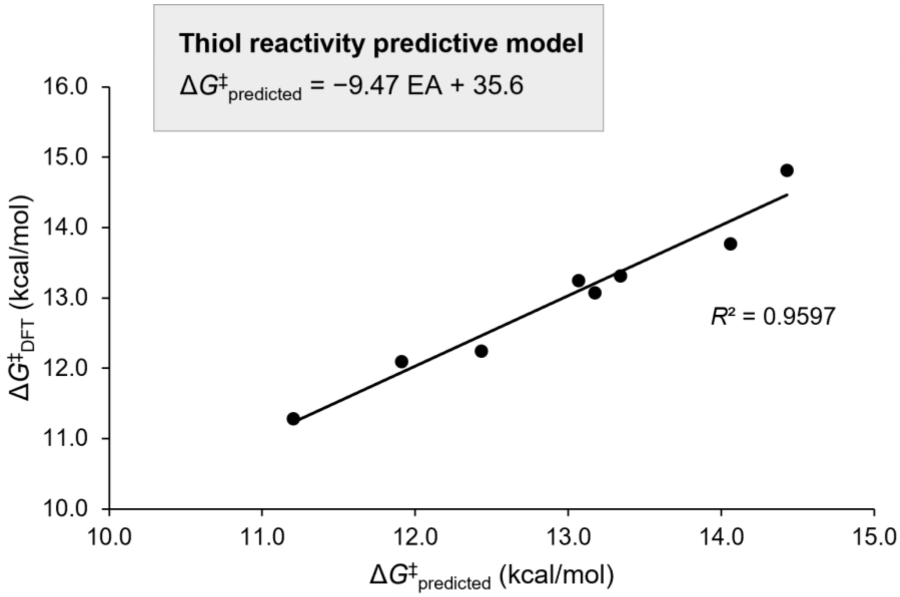

Figure S25. Single-parameter linear regression model using electron affinity.

Multivariate linear regression models were also examined (see Figures S26–S31), although they did not provide substantial performance improvements. Interestingly, one of the best two-parameter models included a steric parameter (Sterimol L) in addition to the electronic parameter EA (see Figure **S27**). However, the improvement to R^2 was minimal, demonstrating that the single-parameter model using EA is already sufficient for predicting the thiol reactivity of this family of *N*-heteroaryl lactams.

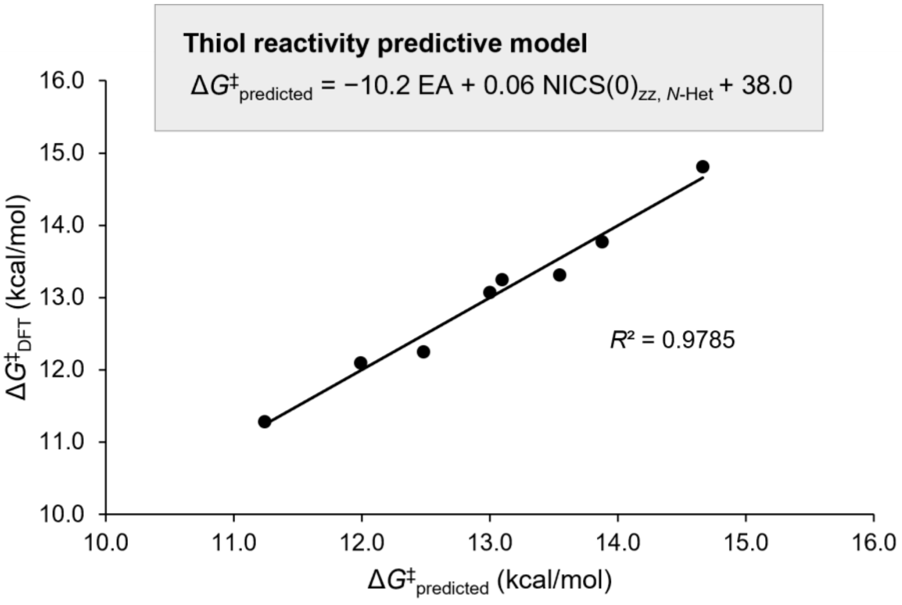

Figure S26. Multivariate linear regression model using electron affinity and $NICS(0)_{zz}$ for *N*-heteroaryl lactams.

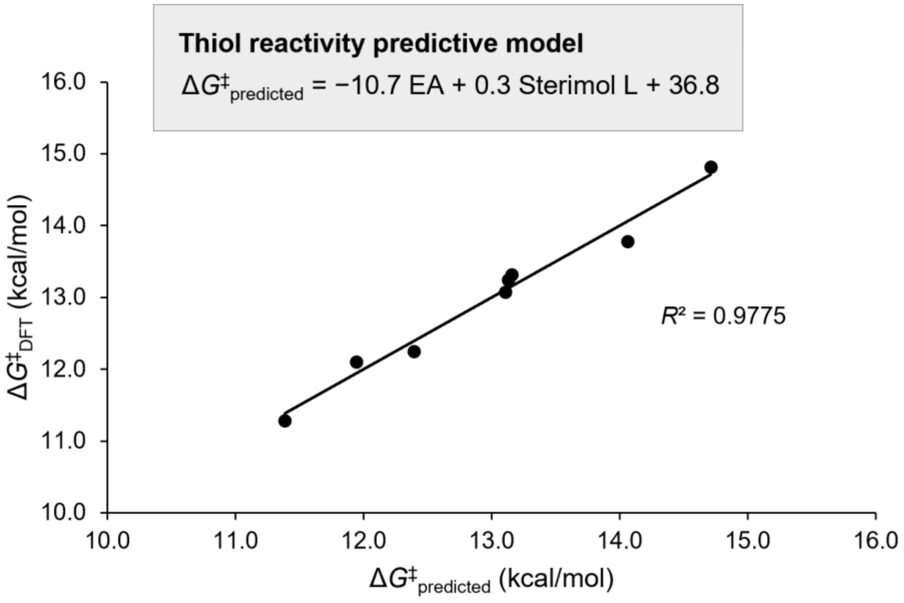

Figure S27. Multivariate linear regression model using electron affinity and Sterimol L.

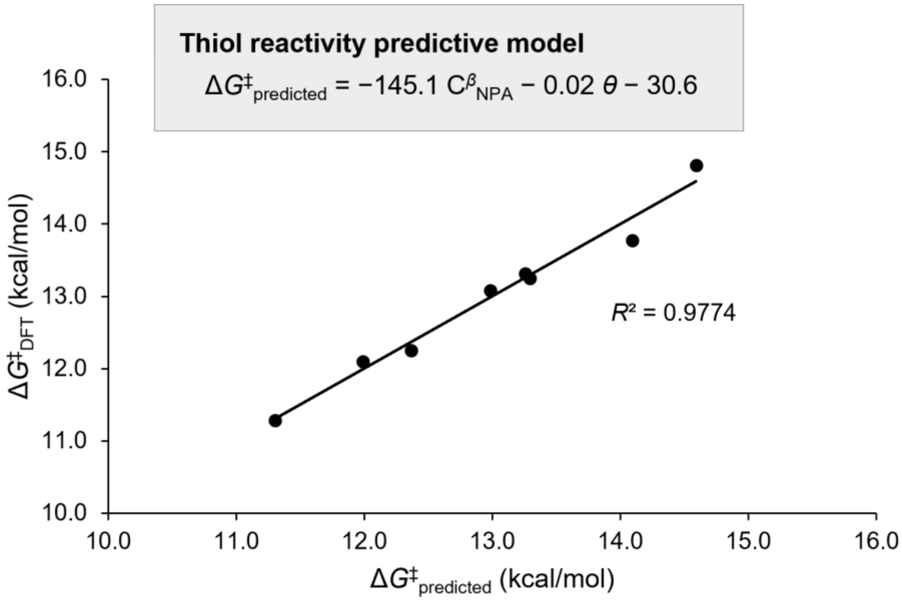

Figure S28. Multivariate linear regression model using NPA charge of C^{β} and *N*-Het dihedral angle, θ.

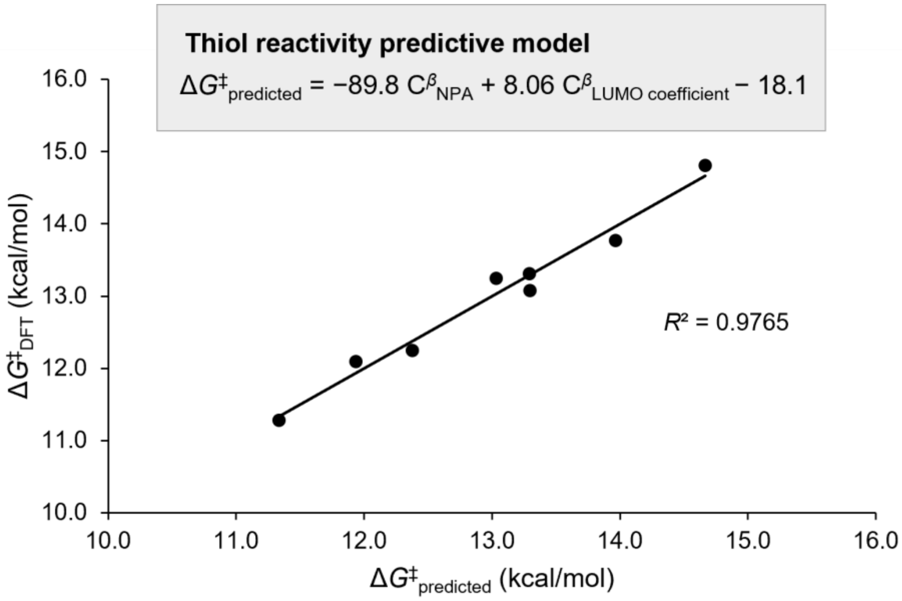

Figure S29. Multivariate linear regression model using NPA charge of C^{β} and LUMO coefficient at C^{β}.

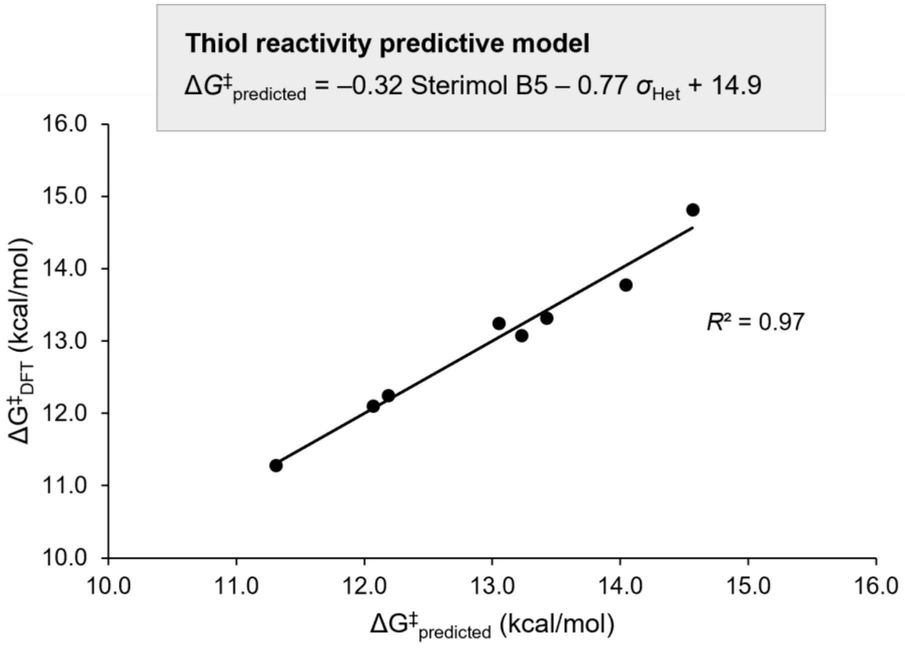

Figure S30. Multivariate linear regression model using σ_{Het} and Sterimol B_5.

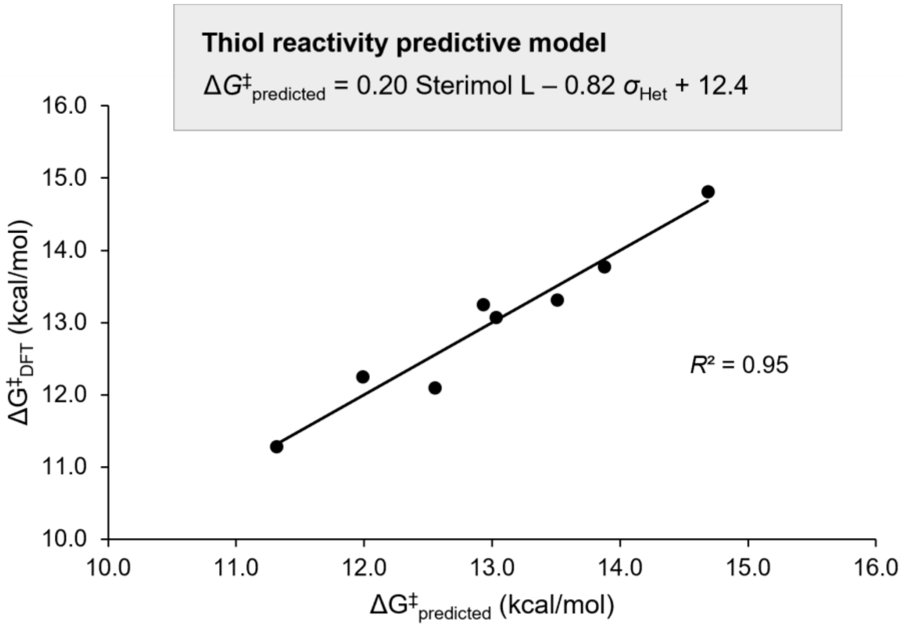

Figure S31. Multivariate linear regression model using σ_{Het} and Sterimol L.

Correlation between $\Delta G^{\ddagger}_{\mathrm{DFT}}$ and the LUMO energy of *N*-heteroaryl *α*-methylene-*γ*-lactams and unsubstituted heteroarenes

Molecular geometries of **1a-1h** and **S2-S9** were optimized using the ωB97X-D functional[14] with the 6-31G(d) basis set for LUMO energy and LUMO coefficient calculations. Vibrational frequency calculations were performed for all the stationary points to confirm that each optimized structure was a local minimum.

CREST/GFN2-xTB conformational sampling of **1a-1h** used for LUMO energy and LUMO coefficient calculations were performed in the gas phase, and the conformers were filtered using a 0.5 Å root-mean-square-deviation (RMSD) threshold and an energy window of 8 kcal/mol. All conformers were fully optimized at the indicated DFT level and the lowest-energy conformer for each substrate was used to obtain LUMO energy and LUMO coefficient values. LUMO orbitals were visualized using IQmol[15] (version 3.1.2).

In previous studies, LUMO energy has been used for CRG reactivity predictions. Here, the LUMO energies of the heteroarenes themselves have a moderate correlation with the computed activation barrier (R^2 = 0.72, see Figure **S34**), and the LUMO energies of the *N*-heteroaryl lactams have a strong correlation with the computed (R^2 = 0.94, see Figure **S32**) and experimental activation barriers (R^2 = 0.82, see Figure **6**). Even so, LUMO energy has a weaker correlation with reactivity with thiols than either EA or NPA charge of C^{β}.

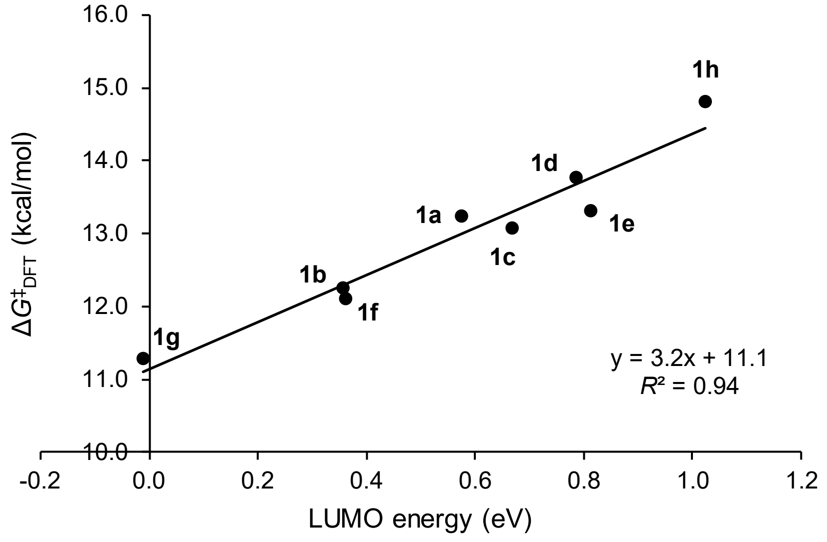

Figure S32. Correlation of $\Delta G^{\ddagger}_{\mathrm{DFT}}$ (kcal/mol) with LUMO energy of *N*-heteroaryl *α*-methylene-*γ*-lactams.

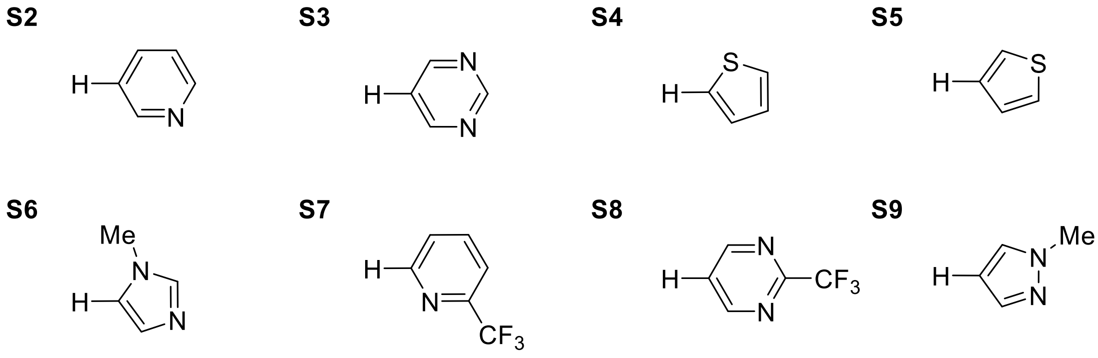

Figure S33. Unsubstituted heteroarene structures used to calculate additional LUMO energy values, Sterimol L/B_1/B_5, NICS(0)$_{zz}$, and NICS(1)$_{zz}$. Hydrogen atoms corresponding to atom 1 (dummy atom) for Sterimol calculations are explicitly shown.

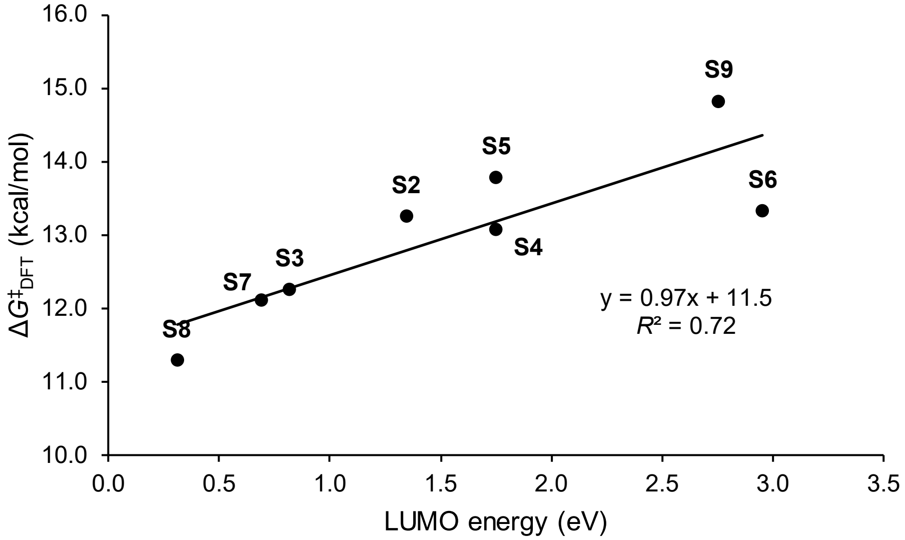

Figure S34. Correlation of $\Delta G^{‡}_{DFT}$ (kcal/mol) with LUMO energy (eV) of unsubstituted heteroarenes.

Table S3. Calculated LUMO energies (E_{LUMO}) of unsubstituted heteroarenes and *N*-heteroaryl *α*-methylene-*γ*-lactams.

Unsubstituted heteroarenes		*N*-heteroaryl *α*-methylene-*γ*-lactams	
**Structure**	**E_{LUMO} (eV)**	**Structure**	**E_{LUMO} (eV)**
**S2**	1.344	**1a**	0.576
**S3**	0.819	**1b**	0.356
**S4**	1.751*	**1c**	0.669
**S5**	1.751*	**1d**	0.787
**S6**	2.955	**1e**	0.814
**S7**	0.690	**1f**	0.361
**S8**	0.308	**1g**	-0.011
**S9**	2.756	**1h**	1.024

*No substitution causes heteroarenes to have identical structures

Correlation between $\Delta G^{\ddagger}_{DFT}$ and the LUMO coefficients at C^{α} and C^{β} of *N*-heteroaryl *α*-methylene-*γ*-lactams

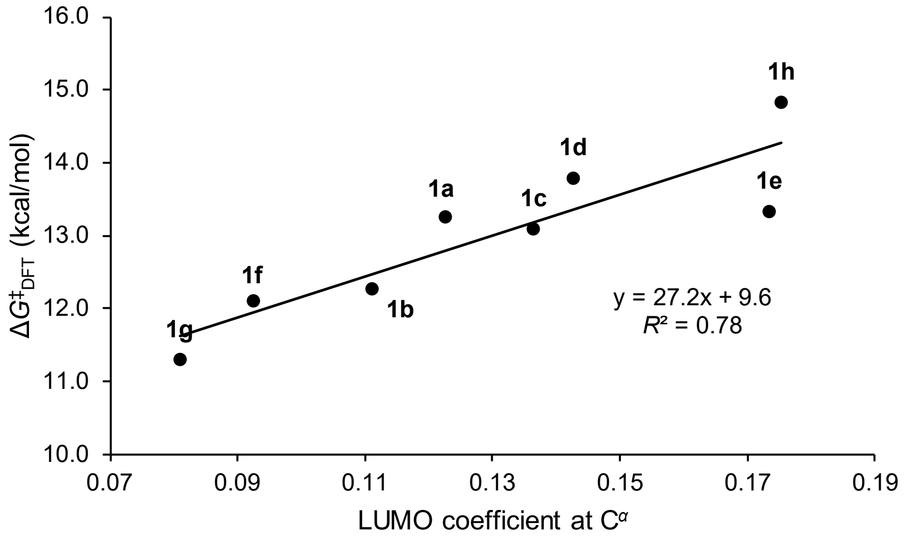

Figure S35. Correlation of $\Delta G^{\ddagger}_{DFT}$ (kcal/mol) with LUMO coefficient at C^{α} of *N*-heteroaryl *α*-methylene-*γ*-lactams.

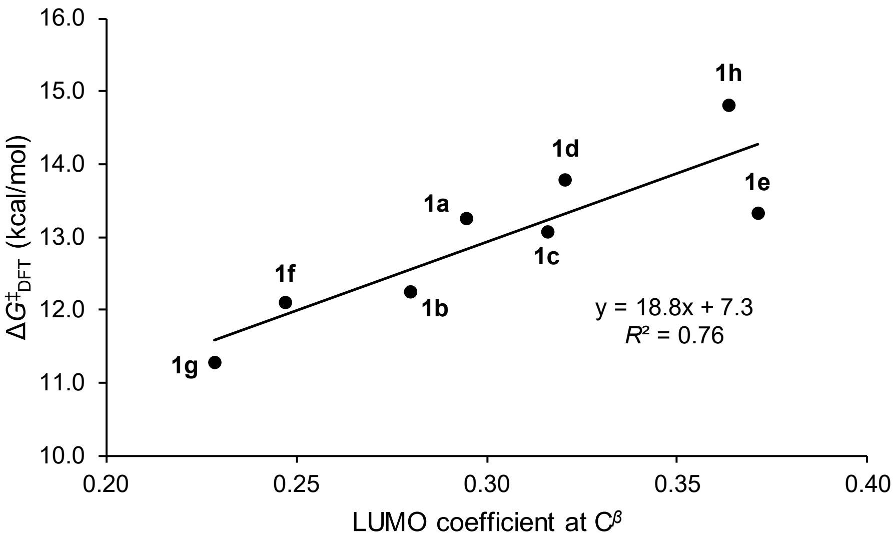

Figure S36. Correlation of $\Delta G^{\ddagger}_{DFT}$ (kcal/mol) with LUMO coefficient at C^{β} of *N*-heteroaryl *α*-methylene-*γ*-lactams.

Table S4. Calculated LUMO coefficients at C^{α} and C^{β} of *N*-heteroaryl *α*-methylene-*γ*-lactams.

**Structure**	**LUMO Coefficient at C^{α}**	**LUMO Coefficient at C^{β}**
**1a**	0.123	0.294
**1b**	0.111	0.280
**1c**	0.136	0.316
**1d**	0.143	0.321
**1e**	0.174	0.371
**1f**	0.092	0.247
**1g**	0.081	0.228
**1h**	0.175	0.363

Correlation between $\Delta G^{\ddagger}_{exp}$ and the electron affinity of *N*-heteroaryl *α*-methylene-*γ*-lactams (see main text for correlation with $\Delta G^{\ddagger}_{DFT}$)

Electron affinity (EA) was defined as the energy released ($E_{initial}$ – E_{final}) when an additional electron is attached to the neutral molecule[16]:

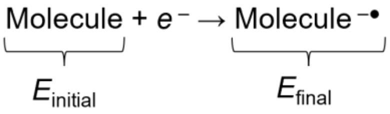

The zero-point corrected energy of the neutral molecule and the radical anion were used to determine $E_{initial}$ and E_{final}, respectively.

The DFT-optimized structures of **1a-1h**, **2a-2i**, **3a-3d**, and **S18-S36** (M06-2X/6-311+G(d,p)/SMD(H_2O)//M06-2X/6-31+G(d)/SMD(H_2O)) were used to obtain $E_{initial}$.

Molecular geometries of radical anions were optimized using the UM06-2X functional with the 6-31+G(d) basis set and SMD solvation model in water. Vibrational frequency calculations were performed for all the stationary points to confirm that each optimized structure was a local minimum. The UM06-2X functional with the 6-311+G(d,p) basis set was used for single point energy calculations in water with the SMD solvation model to obtain E_{final}.

In the case of protonated **1e**, **2a**, and **3c**, $E_{initial}$ represents the cation and E_{final} represents the neutral radical.

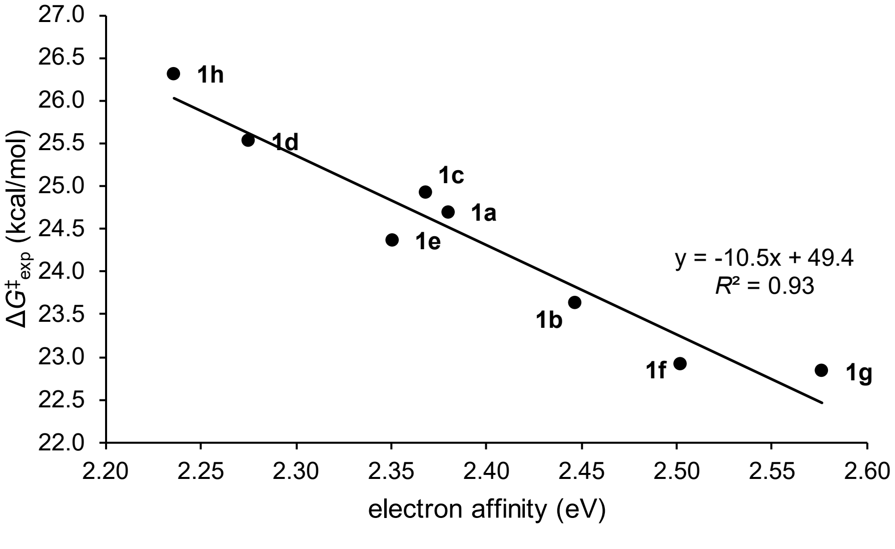

Figure S37. Correlation of $\Delta G^{\ddagger}_{exp}$ (kcal/mol) with electron affinity of *N*-heteroaryl *α*-methylene-*γ*-lactams.

Table S5. Calculated electron affinity (EA) values of *N*-heteroaryl *α*-methylene-*γ*-lactams.

Structure	EA (eV)
**1a**	2.380
**1b**	2.447
**1c**	2.368
**1d**	2.275
**1e**	2.351
**1f**	2.502
**1g**	2.576
**1h**	2.236

Correlation between $\Delta G^{\ddagger}_{DFT}$ and heteroaryl Hammett-type substituent constants (σ_{Het})

Because experimental pK_a values of many heteroaryl carboxylic acids are not available, we calculated the pK_a values of the heteroaryl carboxylic acids of interest in aqueous solution using an approach described by Smith *et al.*[17] In addition to the procedure outlined by Smith *et al.*, two additional steps were added. RDKit[18] was used to generate the initial Cartesian coordinates of the heteroaryl carboxylic acids from SMILES. A tandem conformer search was performed by RDKit and then performed by CREST using the xTB package to find the lowest energy conformer of each heteroaryl carboxylic acid. The lowest energy conformer for each heteroaryl carboxylic acid was used for subsequent DFT calculations.

To calculate Gibbs free energies of the protonated form of the carboxylic acids ($\Delta G^{*}_{\mathrm{aq}}(\mathrm{AH})$) and their corresponding conjugate bases ($\Delta G^{*}_{\mathrm{aq}}(\mathrm{A}^{-})$), the geometries of these compounds were optimized using the B3LYP-D3 functional[19–20] with the 6-31G(d) basis set in water using the SMD solvation model with the default parameters. Vibrational frequency calculations were performed for all the stationary points to confirm that each optimized structure was a local minimum. Single point energies were calculated using the M06 functional[3] with the 6-311+G(d,p) basis set in water using the SMD solvation model with the default parameters.

The Gibbs free energy of proton in aqueous solution ($\Delta G^{*}_{\mathrm{aq}}(\mathrm{H}^{+})$) was calculated from the gas-phase free energy of proton ($G^{\circ}_{\mathrm{g}}(\mathrm{H}^{+}) = -6.28\ \mathrm{kcal/mol}$)[21] and its hydration free energy ($\Delta G_{\mathrm{aq,solv}}(\mathrm{H}^{+}) = -265.9\ \mathrm{kcal/mol}$) from literature.[22–24] $\Delta G^{\circ\rightarrow *} = RT\ln(24.47) = 1.89\ \mathrm{kcal/mol}$ is the free energy change to convert from the standard state of an ideal gas (1 atm) to the standard state in solution (1 M).[4]

$$\mathrm{AH}^{+}(\mathrm{aq}) \xrightarrow{\Delta G^{*}_{\mathrm{aq}}} \mathrm{A}(\mathrm{aq}) + \mathrm{H}^{+}(\mathrm{aq}) \qquad \text{(eqn } \mathbf{S1}\text{)}$$

$$\mathrm{p}K_\mathrm{a} = \frac{\Delta G^*_\mathrm{aq}}{2.303RT} \qquad \text{(eqn } \mathbf{S2}\text{)}$$

$$\Delta G^*_\mathrm{aq} = G^*_\mathrm{aq}(\mathrm{A}) + G^*_\mathrm{aq}(\mathrm{H^+}) - G^*_\mathrm{aq}(\mathrm{AH^+}) \qquad \text{(eqn } \mathbf{S3}\text{)}$$

$$G^*_\mathrm{aq}(\mathrm{H^+}) = G^\mathrm{o}_\mathrm{g}(\mathrm{H^+}) + \Delta G_\mathrm{aq,solv}(\mathrm{H^+}) + \Delta G^{\mathrm{o}\rightarrow *} \qquad \text{(eqn } \mathbf{S4}\text{)}$$

Scheme S1. Acid dissociation constant (pK_a) calculations.

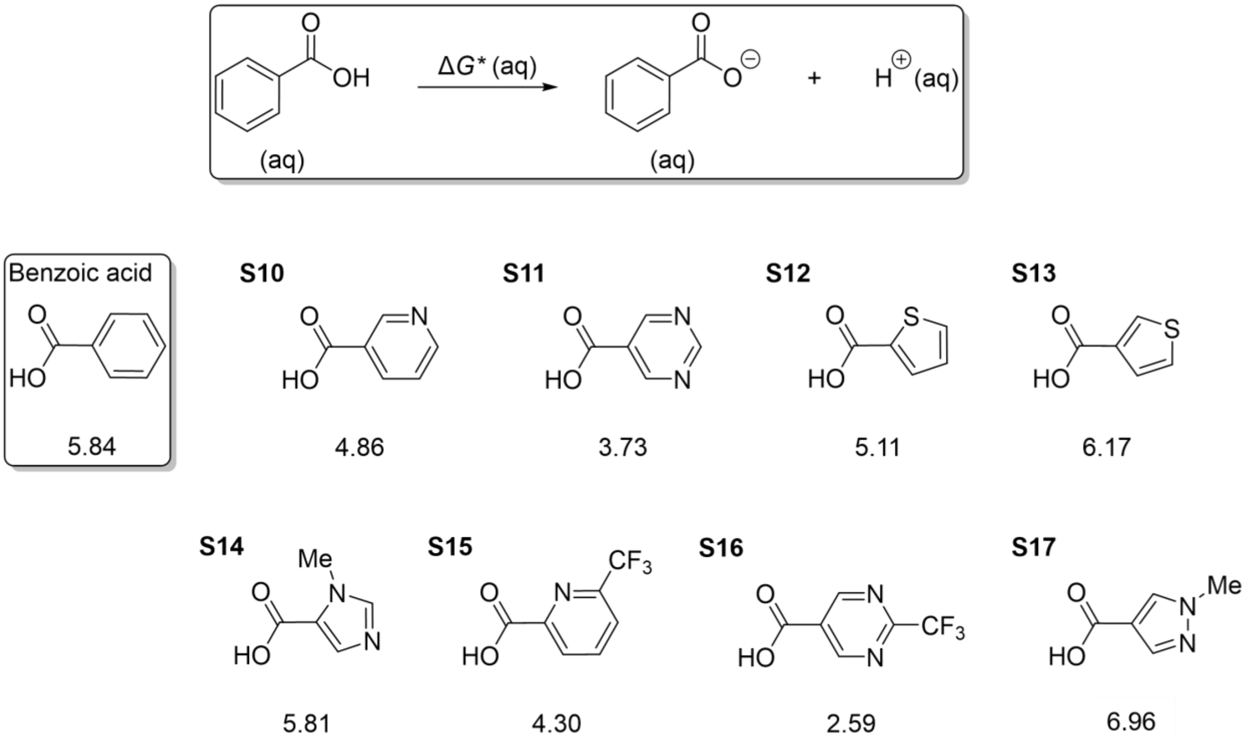

Figure S38. Reference reaction and calculated aqueous pK_a values for heteroaryl carboxylic acids.

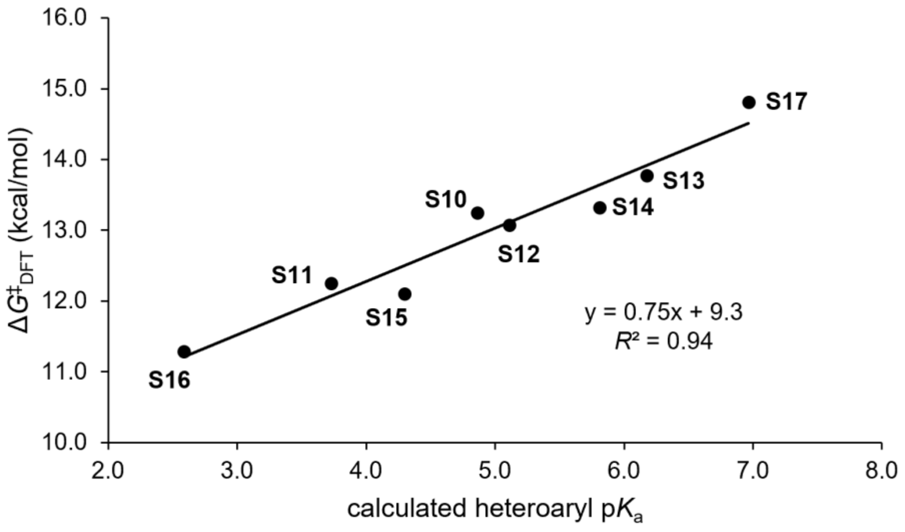

Figure S39. Correlation of $\Delta G^{\ddagger}_{DFT}$ (kcal/mol) with calculated pK_a of heteroaryl carboxylic acids.

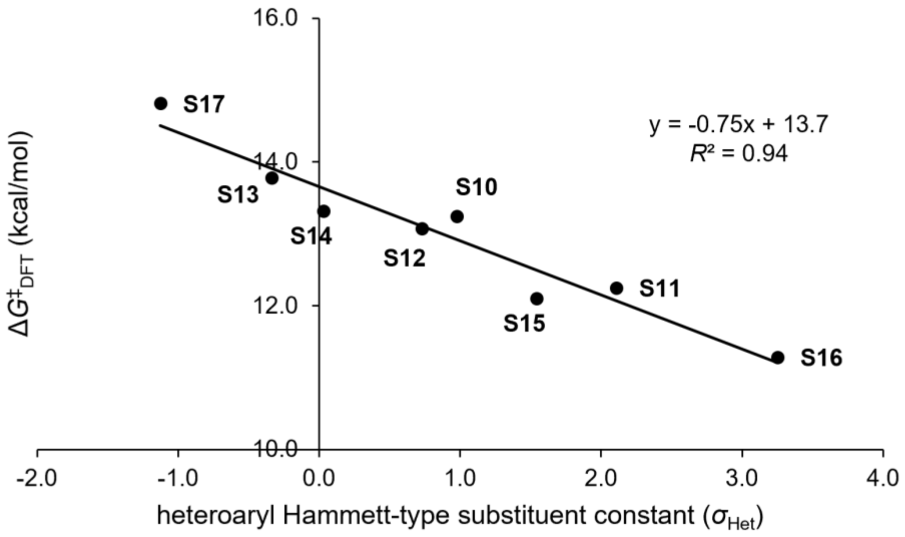

Figure S40. Correlation of $\Delta G^{\ddagger}_{DFT}$ (kcal/mol) with calculated heteroaryl Hammett-type substituent constants (σ_{Het}).

Hammett substituent constants for several heteroaryl groups as *meta*- and *para*-substituents on benzene rings have been reported in the literature. We investigated the correlation between experimental activation barriers with these traditional Hammett substituent constants.

Table S6. Hammett substituent constants for available heteroarenes from literature.[25]

Heteroaryl Group	σ_m	σ_p
3-pyridinyl	0.23	0.25
5-pyrimidinyl	0.28	0.39
2-thiophenyl	0.09	0.05
3-thiophenyl	0.03	-0.02

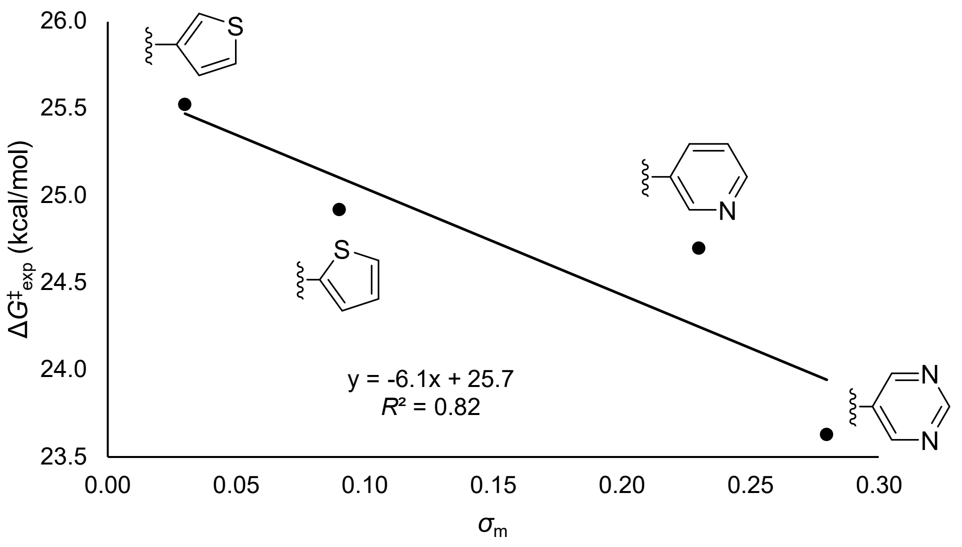

Figure S41. Correlation of $\Delta G^{\ddagger}_{DFT}$ (kcal/mol) with Hammett substituent constants (σ_m) available from literature.

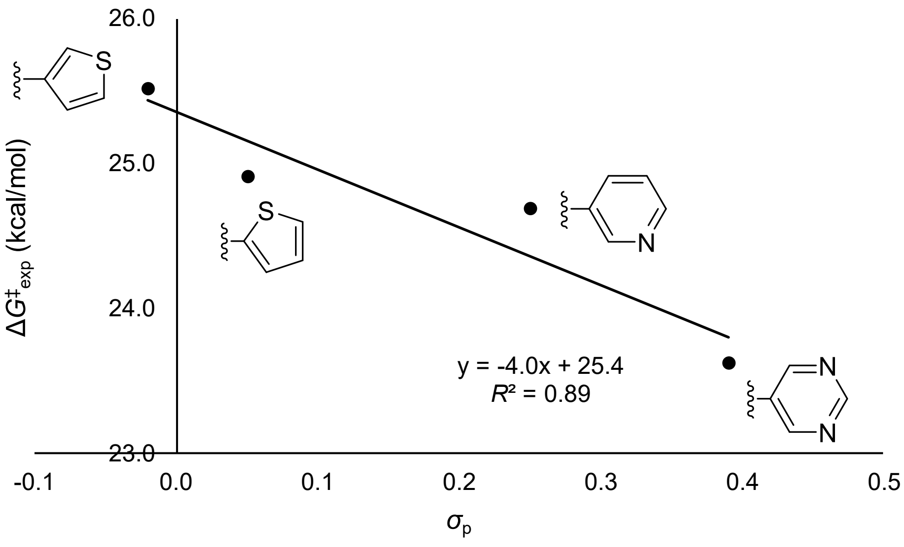

Figure S42. Correlation of $\Delta G^{\ddagger}_{DFT}$ (kcal/mol) with Hammett substituent constants (σ_p) available from literature.

Correlation between $\Delta G^{\ddagger}_{DFT}$ and Sterimol L, B_1, and B_5 values

Molecular geometries of unsubstituted heteroarenes were optimized using the M06-2X functional with the 6-31+G(d) basis set and SMD solvation model in water. Vibrational frequency calculations were performed for all the stationary points to confirm if each optimized structure was a local minimum or a transition state structure. Sterimol L, B_1, and B_5 values[26] were calculated using the Morfeus package.[27] The dummy atom was assigned as hydrogen with Bondi radii (r(H) = 1.20 Å). See Figure S33 for unsubstituted heteroarene structures and dummy atom definition for Sterimol calculations.

The weak correlations of Sterimol parameters with both computed and experimental activation barriers indicate that the steric property of the heteroarenes is not a dominant factor for reactivity (see Figures **S43-S45** and Figure **6**).

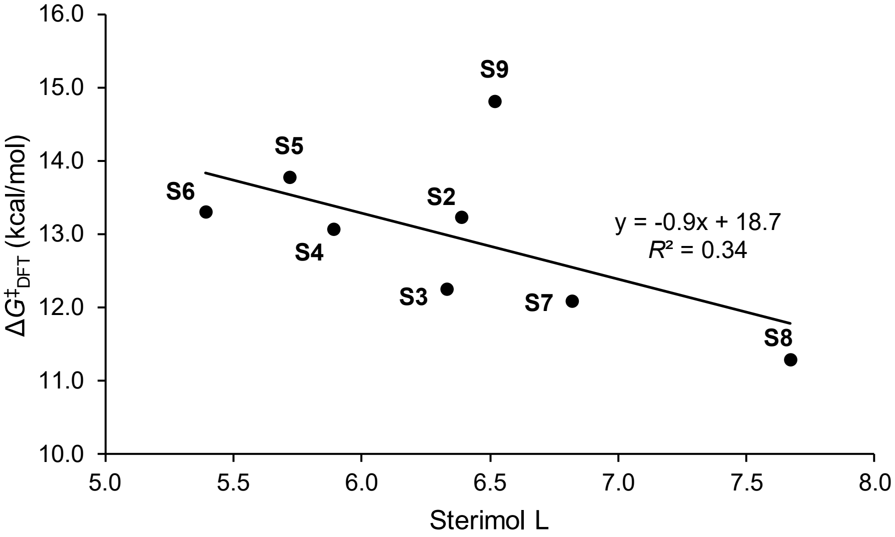

Figure S43. Correlation of $\Delta G^{\ddagger}_{DFT}$ (kcal/mol) with Sterimol L (Å) of unsubstituted heteroarenes.

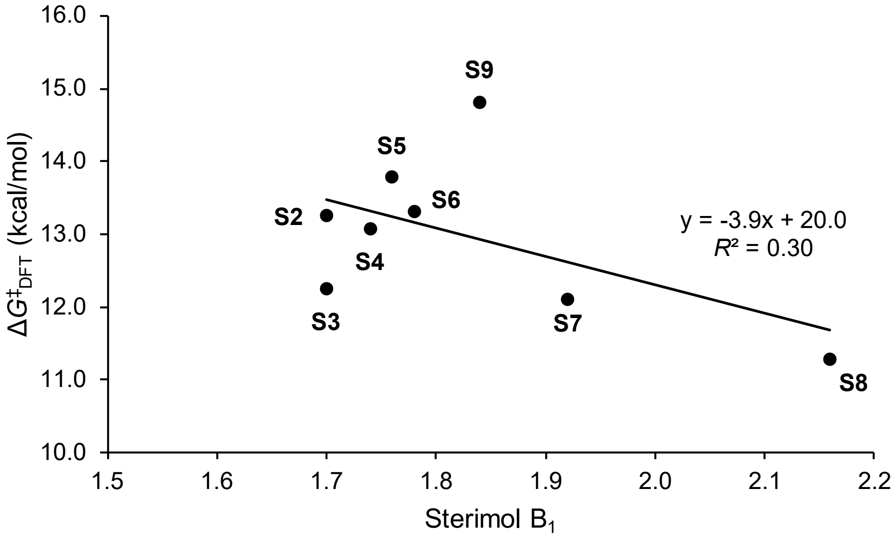

Figure S44. Correlation of $\Delta G^{\ddagger}_{DFT}$ (kcal/mol) with Sterimol B_1 (Å) of unsubstituted heteroarenes.

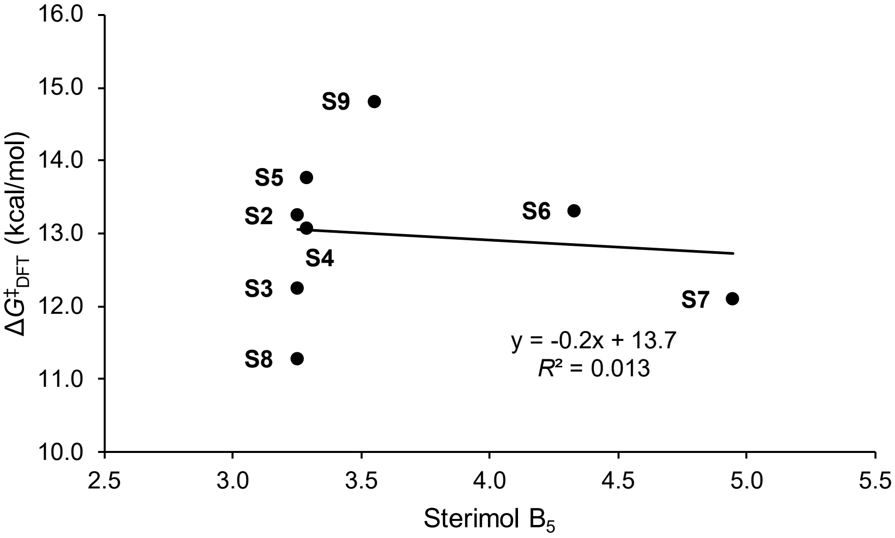

Figure S45. Correlation of $\Delta G^{\ddagger}_{DFT}$ (kcal/mol) with Sterimol B_5 (Å) of unsubstituted heteroarenes.

Table S7. Calculated Sterimol L, B_1, and B_5 values for unsubstituted heteroarenes. All values are reported in Å.

Structure	Sterimol L	Sterimol B_1	Sterimol B_5
**S2**	6.39	1.70	3.25
**S3**	6.33	1.70	3.25
**S4**	5.89	1.74	3.29
**S5**	5.72	1.76	3.29
**S6**	5.39	1.78	4.33
**S7**	6.82	1.92	4.95
**S8**	7.67	2.16	3.25
**S9**	6.52	1.84	3.55

Correlation between Δ$G^‡_{DFT}$ and NICS(0)$_{zz}$ and NICS(1)$_{zz}$ aromaticity indices of heteroarenes and *N*-heteroaryl lactams

The geometries of unsubstituted heteroarenes **S2**-**S9** optimized at the M06-2X/6-31+G(d)/SMD(H_2O) level of theory were used for Sterimol, NICS(0)$_{zz}$, and NICS(1)$_{zz}$ calculations. The structures of **1a-1h** and **TS1a-TS1h** optimized at the M06-2X/6-31+G(d)/SMD(H_2O) level of theory were used to calculate NICS(0)$_{zz}$ and NICS(1)$_{zz}$ for the reactants as well as thio-Michael transition states.

Values were calculated using Multiwfn[28] (version 3.8) in conjunction with Gaussian 16 at the M06-2X/6-31+G(d)/SMD(H_2O) level of theory. NICS(0)$_{zz}$ and NICS(1)$_{zz}$ were calculated using the center of mass of the heteroarene, and NICS(1)$_{zz}$ was calculated as the average of the GIAO magnetic shielding tensors[29] at the points 1 Å below and above the defined plane of the heteroarene.

NICS(0)$_{zz}$ and NICS(1)$_{zz}$ values for **S2**-**S9** and **1a-1h** were evaluated as parameters (Het and *N*-Het, respectively).

Examination of other parameters provided additional insights into the impact of heteroaryl substituents on the reactivity of *N*-heteroaryl lactams. The relationship between NICS(1)$_{zz}$ values for heteroarenes and the computed activation barriers (R^2 = 0.69 for NICS(1)$_{zz}$ of unsubstituted heteroarenes, Figure **S47**; R^2 = 0.84 for NICS(1)$_{zz}$ of *N*-heteroaryl lactams, Figure **S50**) suggests that heteroarenes with greater aromatic character react at slower rate due to the disruption of aromaticity upon thiolate addition (see Figure **2B**).

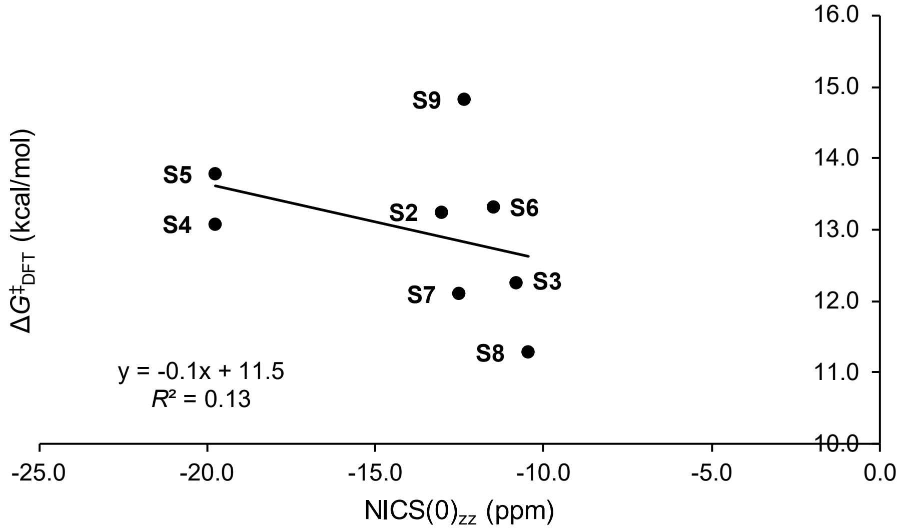

Figure S46. Δ$G^‡_{DFT}$ (kcal/mol) vs. NICS(0)$_{zz}$ value of unsubstituted heteroarenes.

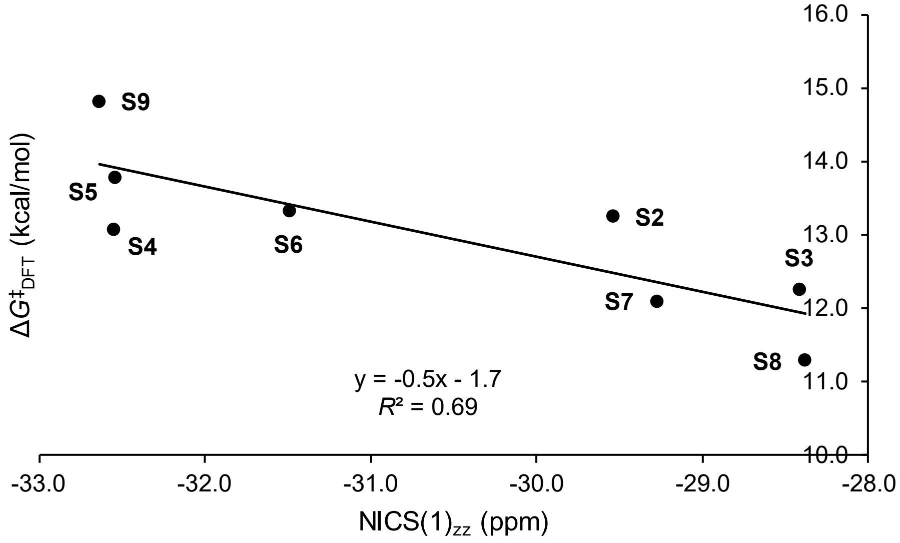

Figure S47. $\Delta G^{‡}_{DFT}$ (kcal/mol) vs. $NICS(1)_{zz}$ value of unsubstituted heteroarenes.

Table S8. Calculated $NICS(0)_{zz}$ and $NICS(1)_{zz}$ values for unsubstituted heteroarenes. All values are reported in ppm.

**Structure**	**$NICS(0)_{zz}$**	**$NICS(1)_{zz}$**
**S2**	-13.023	-29.541
**S3**	-10.796	-28.417
**S4**	-19.751	-32.546
**S5**	-19.743	-32.539
**S6**	-11.483	-31.490
**S7**	-12.491	-29.275
**S8**	-10.447	-28.382
**S9**	-12.334	-32.634

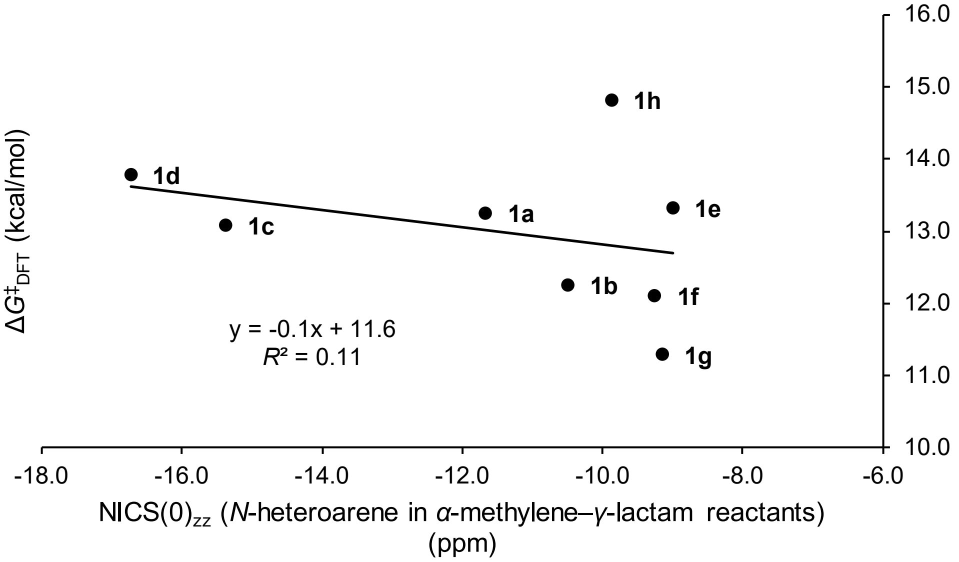

Figure S48. Correlation of $\Delta G^{\ddagger}_{DFT}$ (kcal/mol) with $NICS(0)_{zz}$ value of the heteroarene in the *N*-heteroaryl *α*-methylene–*γ*-lactam reactants.

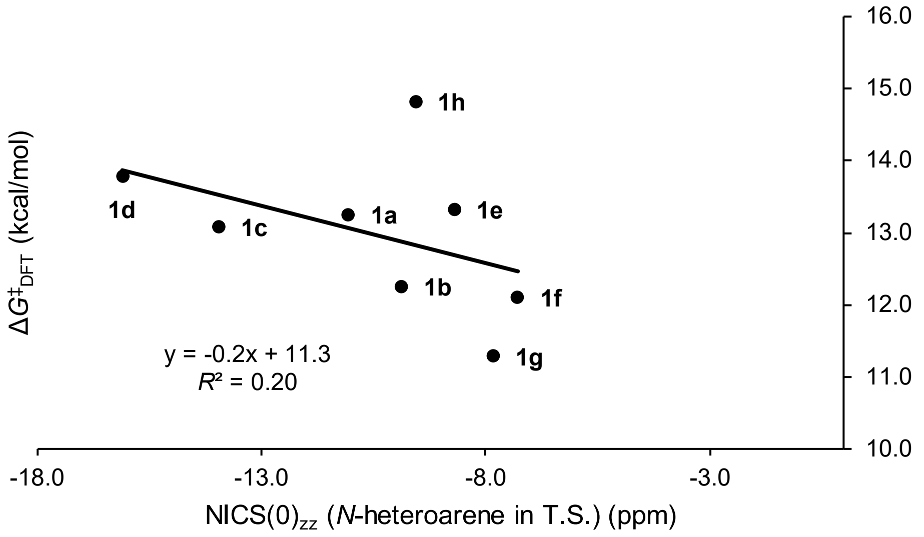

Figure S49. Correlation of $\Delta G^{\ddagger}_{DFT}$ (kcal/mol) with $NICS(0)_{zz}$ value of the heteroarene in the thio-Michael transition states.

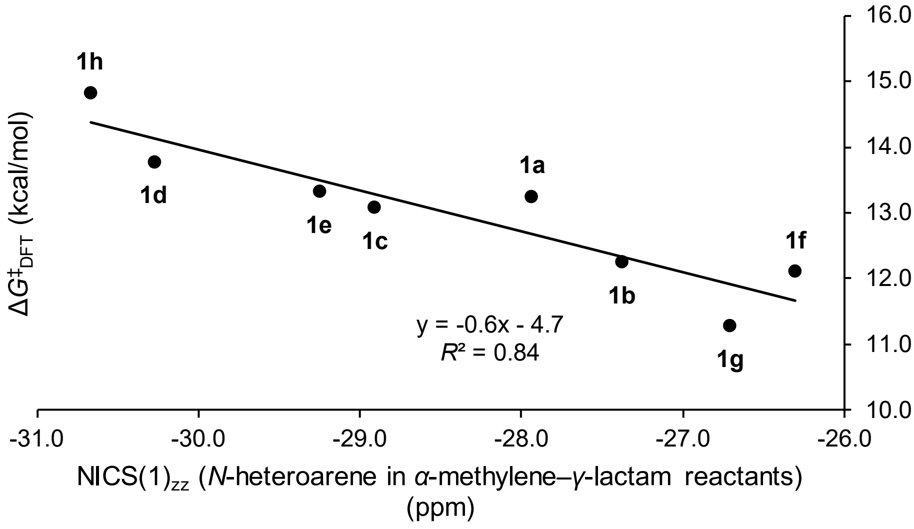

Figure S50. Correlation of $\Delta G^{\ddagger}_{DFT}$ (kcal/mol) with $NICS(1)_{zz}$ value of the heteroarene in the *N*-heteroaryl *α*-methylene–*γ*-lactam reactants.

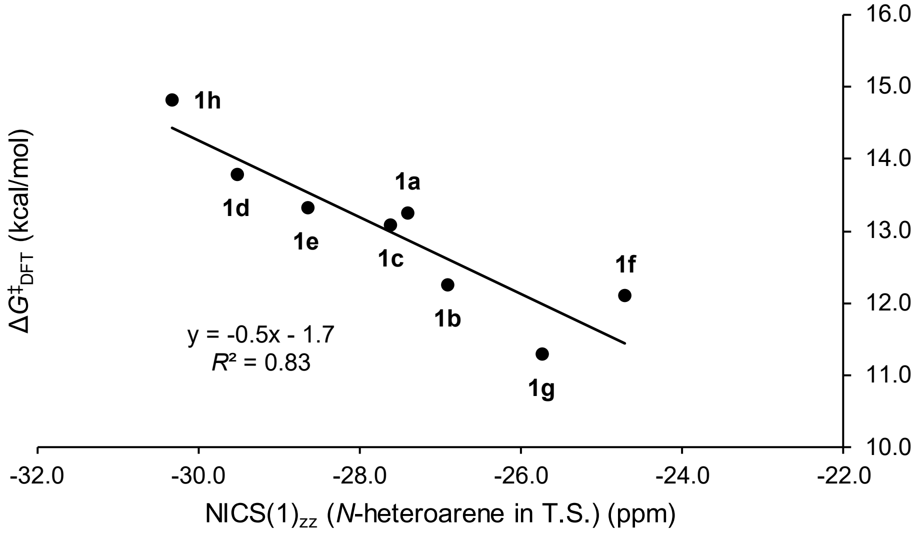

Figure S51. Correlation of $\Delta G^{\ddagger}_{DFT}$ (kcal/mol) with $NICS(1)_{zz}$ value of the heteroarene in the thio-Michael transition states.

Table S9. Calculated NICS(0)$_{zz}$ and NICS(1)$_{zz}$ values for the heteroarenes in the *N*-heteroaryl *α*-methylene–*γ*-lactam reactants and thio-Michael transition states. All values are reported in ppm.

Structure	NICS(0)$_{zz}$	NICS(1)$_{zz}$
Heteroarenes in the *N*-heteroaryl *α*-methylene–*γ*-lactam reactants		
**1a**	-11.673	-27.933
**1b**	-10.493	-27.374
**1c**	-15.380	-28.907
**1d**	-16.731	-30.274
**1e**	-8.994	-29.245
**1f**	-9.257	-26.303
**1g**	-9.148	-26.704
**1h**	-9.875	-30.668
Heteroarenes in the thio-Michael transition states		
**TS1a**	-11.070	-27.412
**TS1b**	-9.873	-26.907
**TS1c**	-13.966	-27.621
**TS1d**	-16.099	-29.528
**TS1e**	-8.685	-28.655
**TS1f**	-7.291	-24.714
**TS1g**	-7.832	-25.744
**TS1h**	-9.567	-30.334

Correlation between Δ$G^‡_{DFT}$ and *N*-Het dihedral angle (*θ*)

As seen in Figures **6** and **S52**, *θ* did not significantly impact the reactivity of the lactams.

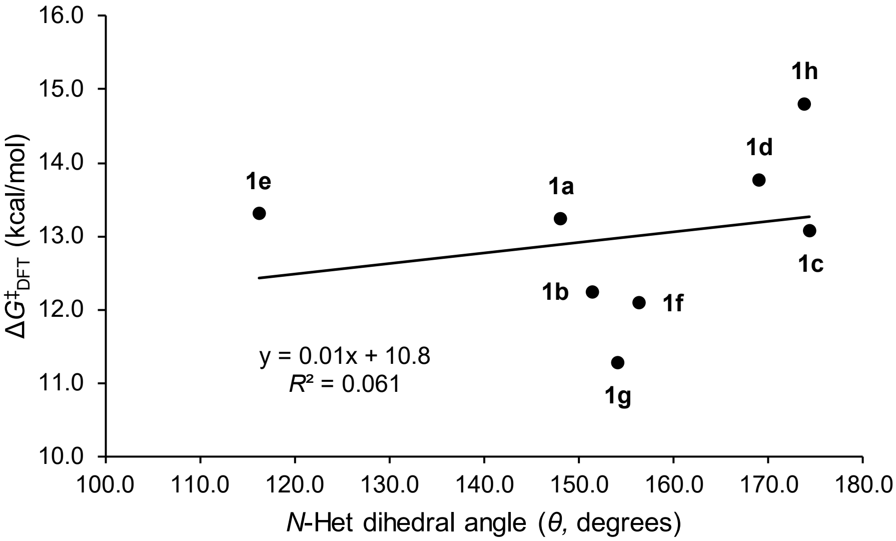

Figure S52. Correlation of Δ$G^‡_{DFT}$ (kcal/mol) with dihedral angle between the plane of the heteroarene and *α*-methylene-*γ*-lactam in the ground state.

Table S10. Calculated dihedral angle between the plane of heteroarene and *α*-methylene-*γ*-lactam in the ground state.

Structure	***N*-Het *θ* (degrees)**
**1a**	148.1
**1b**	151.4
**1c**	174.4
**1d**	169.0
**1e**	116.3
**1f**	156.3
**1g**	154.1
**1h**	173.8

Correlation between $\Delta G^{\ddagger}_{DFT}$ and NPA charge of C^{β} of *N*-heteroaryl *α*-methylene-*γ*-lactams in the ground state

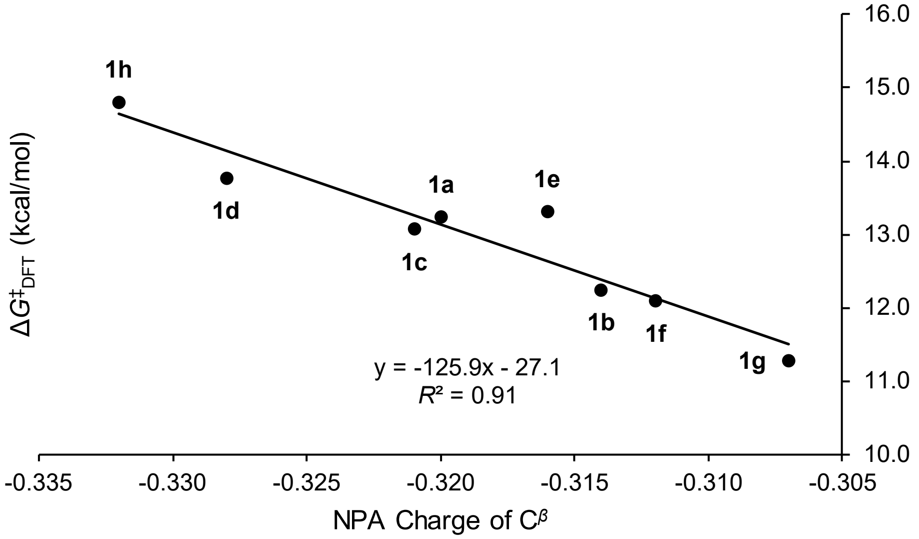

Figure S53. Correlation of $\Delta G^{\ddagger}_{DFT}$ (kcal/mol) with NPA charge of C^{β} of *N*-heteroaryl *α*-methylene-*γ*-lactams in the ground state.

Table S11. Calculated NPA charge of C^{β} of *N*-heteroaryl *α*-methylene-*γ*-lactams in the ground state.

**Structure**	**NPA charge of C^{β}**
**1a**	-0.320
**1b**	-0.314
**1c**	-0.321
**1d**	-0.328
**1e**	-0.316
**1f**	-0.312
**1g**	-0.307
**1h**	-0.332

Correlation between $\Delta G^{\ddagger}_{DFT}$ and chemical shift of $H^{\beta 1}$ (^{1}H NMR) and C^{β} (^{13}C NMR) of *N*-heteroaryl *α*-methylene-*γ*-lactams

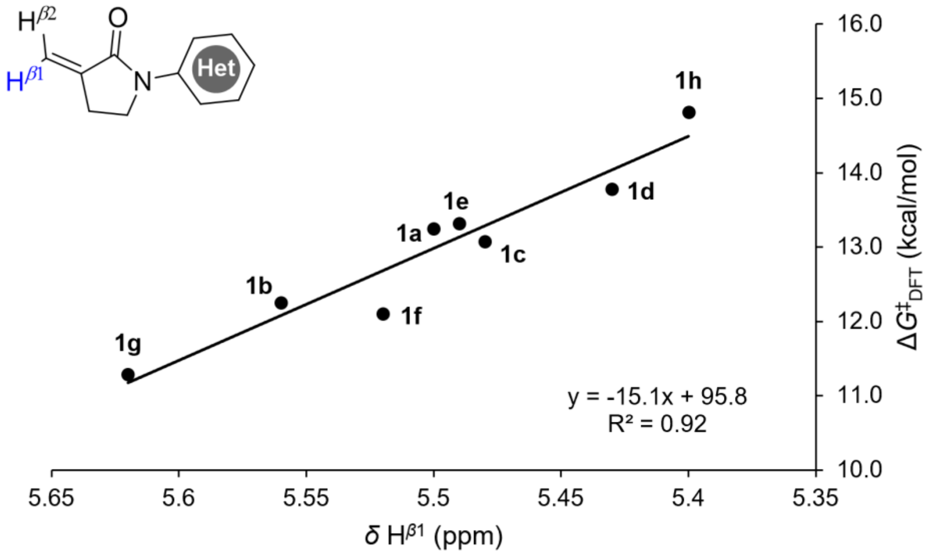

Figure S54. Correlation of $\Delta G^{\ddagger}_{DFT}$ (kcal/mol) with ^{1}H NMR shifts.

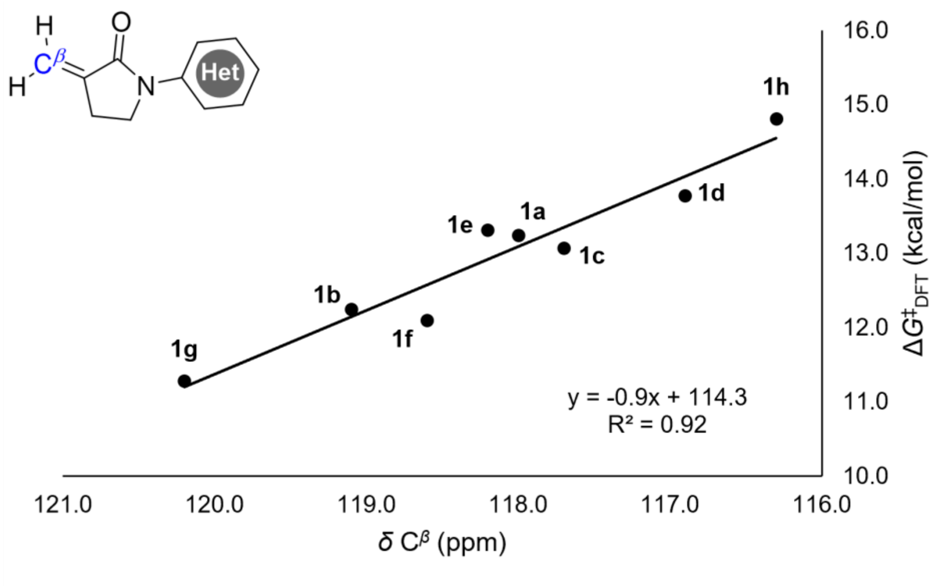

Figure S55. Correlation of $\Delta G^{\ddagger}_{calc}$ (kcal/mol) with ^{13}C NMR shifts.

Development of a single-parameter thiol reactivity predictive model using additional *N*-heteroaryl lactams as a test set

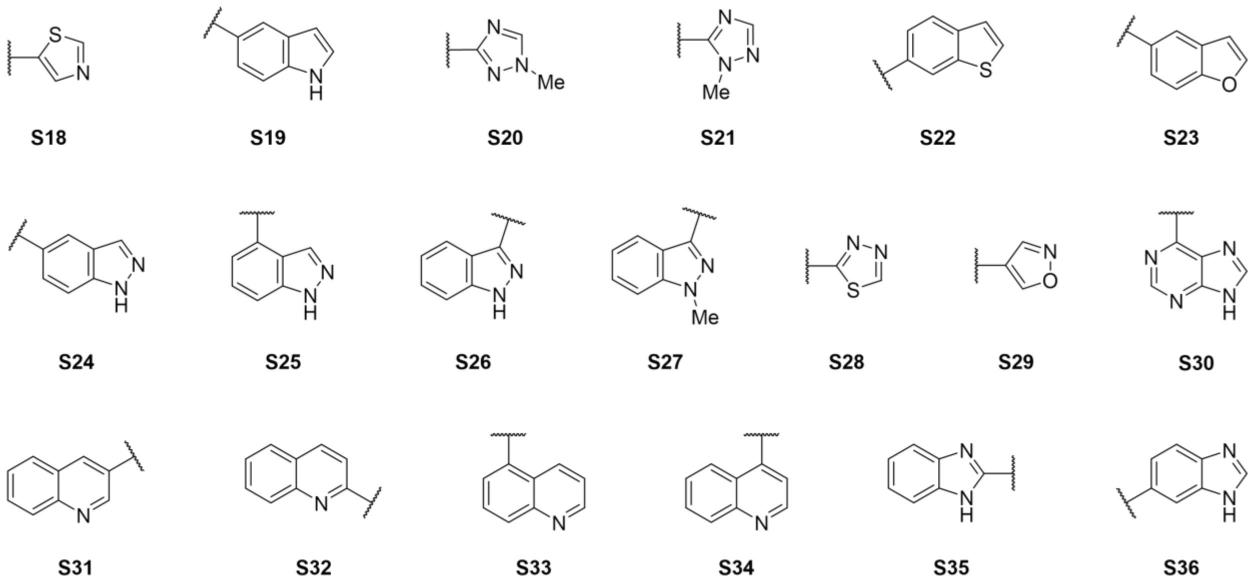

Figure S56. Additional *N*-heteroaryl *α*-methylene–*γ*-lactams considered for a test set based on common heteroarenes found in FDA-approved drugs and expanding the diversity of the dataset.

Table S12. Calculated EA for test set and other considered *N*-heteroaryl *α*-methylene–*γ*-lactams.

Structure	EA (eV)	Structure	EA (eV)
**S18**	2.435	**S28**	2.615
**S19**	2.250	**S29**	2.344
**S20**	2.349	**S30**	2.548
**S21**	2.465	**S31**	2.391
**S22**	2.343	**S32**	2.489
**S23**	2.293	**S33**	2.456
**S24**	2.292	**S34**	2.494
**S25**	2.371	**S35**	2.547
**S26**	2.361	**S36**	2.302
**S27**	2.343		

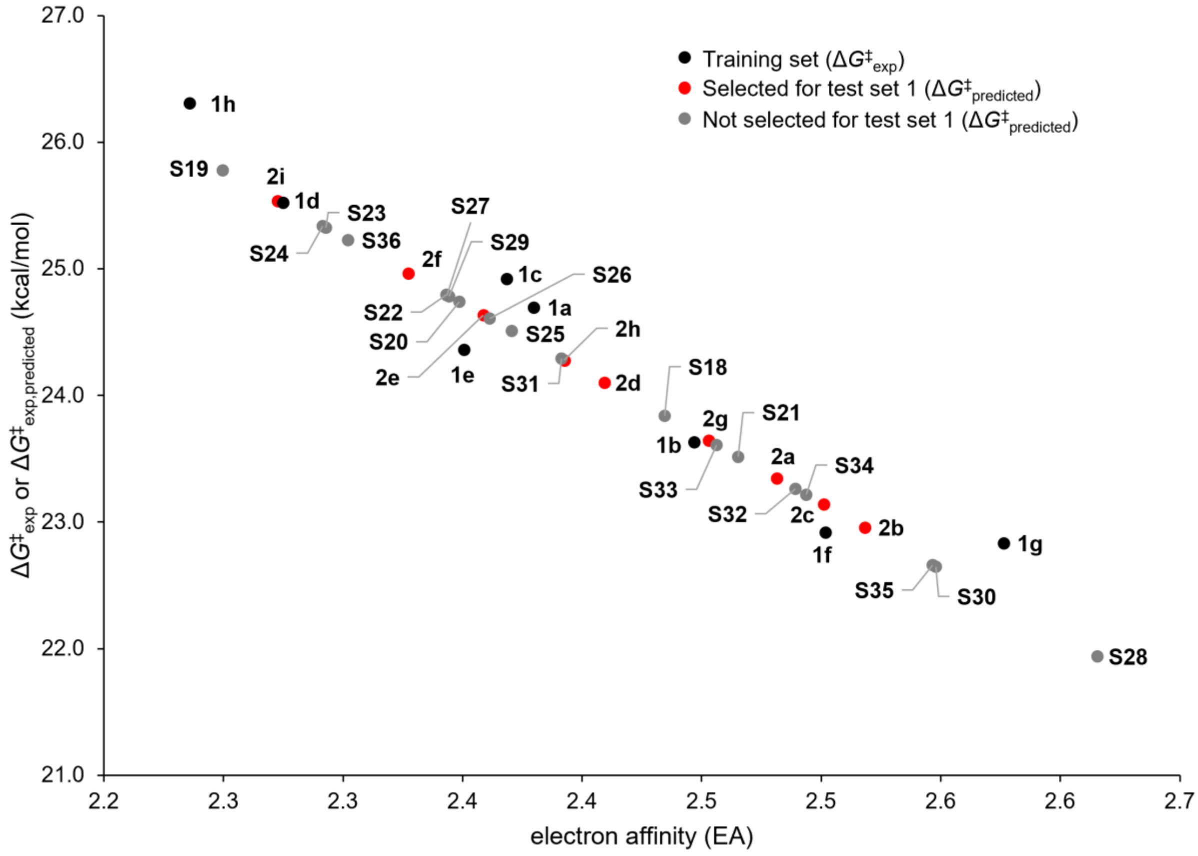

Figure S57. Correlation of $\Delta G^{\ddagger}_{exp}$ (**1a-1h**) or $\Delta G^{\ddagger}_{predicted}$ (**2a-2i**, **S18-S36**) (kcal/mol) with calculated electron affinity (eV). EA for **1e** and EA and $\Delta G^{\ddagger}_{predicted}$ values for **2a** are not weighted (unprotonated values were used).

Development of a single-parameter thiol reactivity predictive model using thiol-reactive acrylamide warheads as a second test set

EA was calculated for **3c** using the unprotonated amine (see Figure **S58**), protonated amine (see Figure **S59**), and the protonated amine forming a hydrogen bonding interaction with one explicit water molecule (see Figure **9C** in the main text and Figure **S60**). The NPA charge of C^{β} was also investigated as a single-parameter predictive model (see Figure **S61**).

Table S13. Calculated EA and NPA charge of C^{β} for test set 2.

Structure	EA (eV)	NPA charge of C^{β}
**3a**	2.375	-0.297
**3b**	2.240	-0.307
**3c'** (**3c** with unprotonated amino group)	2.289	-
**3c''** (**3c** with protonated amino group)	2.623	-
**3c''** (**3c** with protonated amino group bound to one explicit water molecule)	2.567	-0.142
**3d**	2.267	-0.305

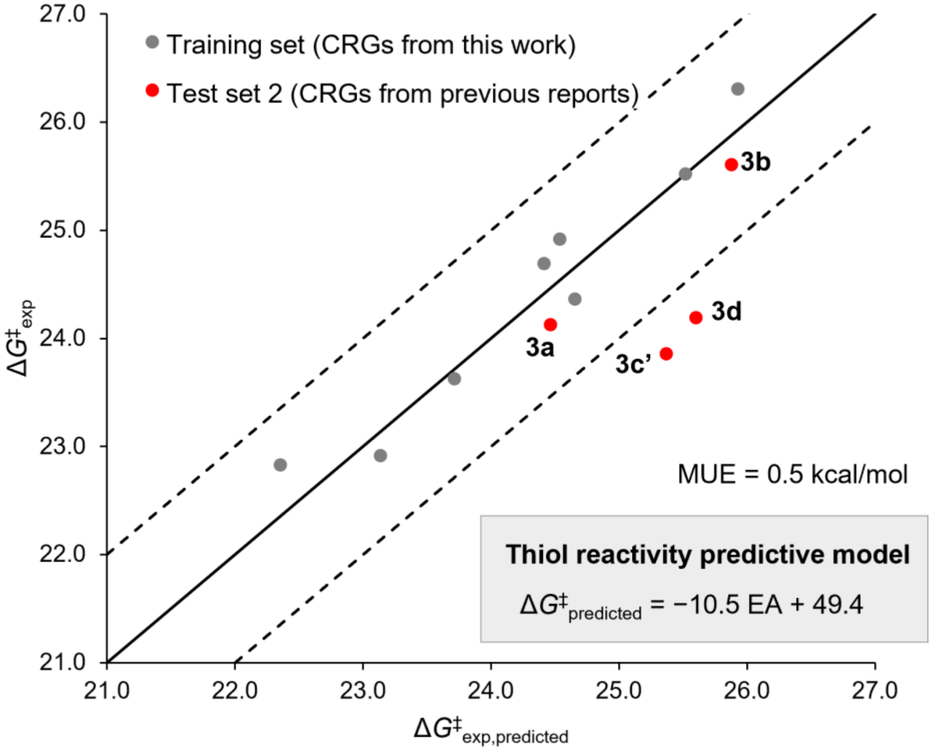

Figure S58. Correlation of $\Delta G^{\ddagger}_{exp}$ (kcal/mol) with $\Delta G^{\ddagger}_{predicted}$ (unprotonated amine).

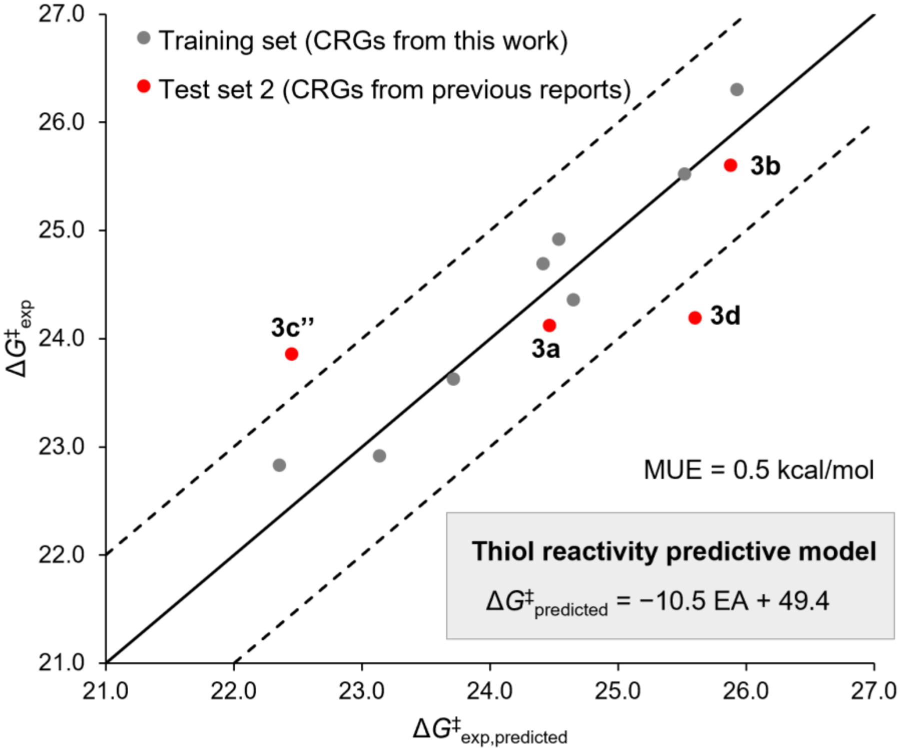

Figure S59. Correlation of $\Delta G^{\ddagger}_{exp}$ (kcal/mol) with $\Delta G^{\ddagger}_{predicted}$ (protonated amine and one hydrogen bonding interaction).

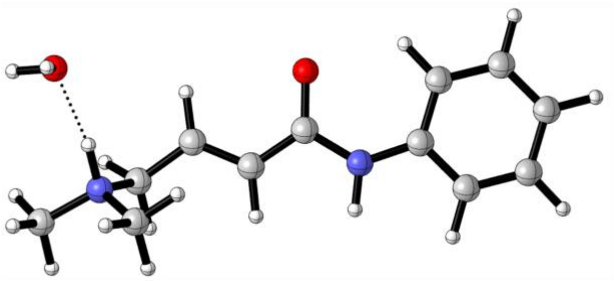

Figure S60. Geometry of **3c''** used in electron affinity calculation for **3c** with protonated amine and one hydrogen bonding interaction.

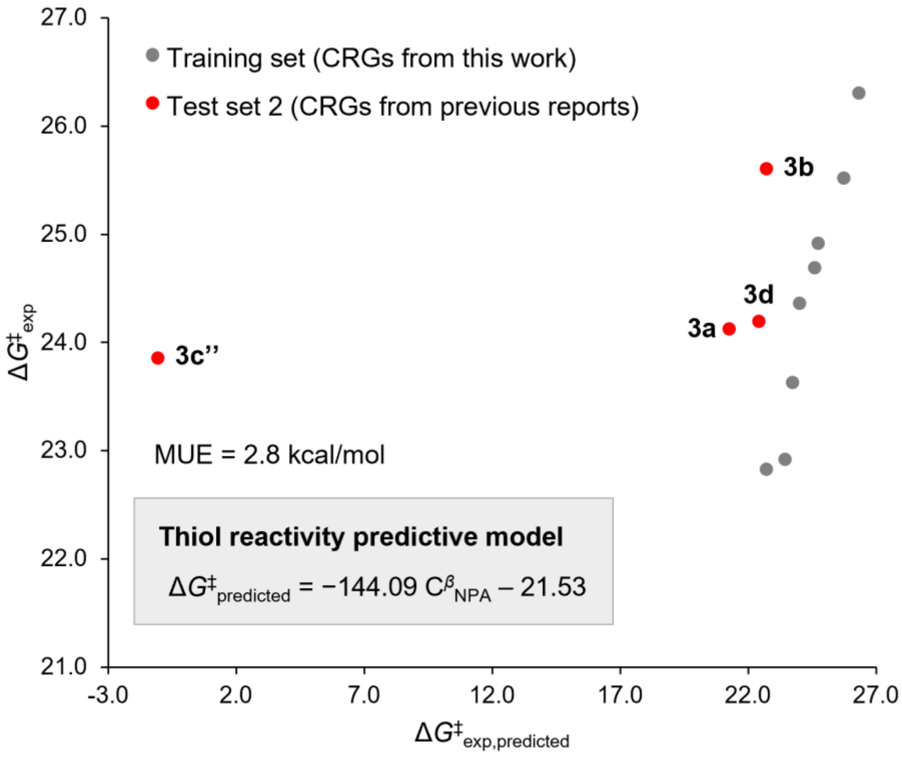

Figure S61. Correlation of $\Delta G^{\ddagger}_{exp}$ (kcal/mol) with $\Delta G^{\ddagger}_{predicted}$ (calculated using NPA charge of C^{β}). The protonated amine with one hydrogen bonding interaction was used to calculate NPA charge for **3c''**. No weighting was performed.

Calculating the weighted $\Delta G^{\ddagger}_{predicted}$ value for 1e, 2a, and 3c

We utilized the machine learning-based pK_a prediction tool from Rowan Scientific to obtain pK_{aH} values for **1a**–**1h**, **2a**–**2g**, and **3a**–**3c**.[8,30–32] Because we expect both unprotonated and protonated **1e**, **2a**, and **3c** exist in equilibrium under the experimental conditions due to their basicity (pK_{aH} ≥ 5.4), and the different protonation states would have different thiol reactivity (e.g. eqn **S5**), we computed the weighted $\Delta G^{\ddagger}_{predicted}$ for **1e**, **2a**, and **3c** based on the ground state equilibrium ratios of the two possible protonation states, and the activation free energy for each protonation state computed according to Figures **8**–**9** in the main text using computed EA values for the corresponding protonation state. The pK_{aH} values were used to obtain a concentration ratio of neutral and protonated species for each lactam ([A]/[HA$^+$]) from the rearranged Henderson-Hasselbalch equation (eqn **S6**) at pH 7.4. DFT-calculated electron affinity values were obtained for both the neutral and protonated species (EA_A and EA_{HA+}, respectively) to obtain $\Delta G^{\ddagger}_{predicted}$ at each state ($\Delta G^{\ddagger}_{predicted,A}$ and $\Delta G^{\ddagger}_{predicted,HA+}$) using the single-parameter model (see Figures **8**–**9** in the main text). $\Delta G^{\ddagger}_{predicted,A}$ and $\Delta G^{\ddagger}_{predicted,HA+}$ were subsequently converted into rate constants (k_A and k_{HA+}) (see eq **3** in the main text). The concentration ratio obtained from eqn **S6** was converted to relative concentrations of A and HA$^+$ ($[A]_{rel}$ and $[HA^+]_{rel}$) using eqn **S7**–**S8**. Weighted rate constants ($k_{weighted,A}$ and $k_{weighted,HA+}$) were obtained by multiplying the rate constant at each

protonation state by the relative concentrations of each species following eqn **S9–S10**. Finally, k_{total} was obtained from adding the weighted rate constants for each species (eqn **S11**), and the weighted $\Delta G^{\ddagger}_{predicted}$ ($\Delta G^{\ddagger}_{weighted}$) was obtained from the Eyring equation using k_{total} (eqn **S12**).

For the EA calculations of the protonated species (EA_{AH+}) of **1e**, **2a**, and **3c**, the nitrogen of interest was protonated and one explicit water molecule was included to form hydrogen bonding with the protonated N-H (e.g. Figure **S60**). The inclusion of an explicit water molecule is expected to improve the accuracy of the EA calculations.

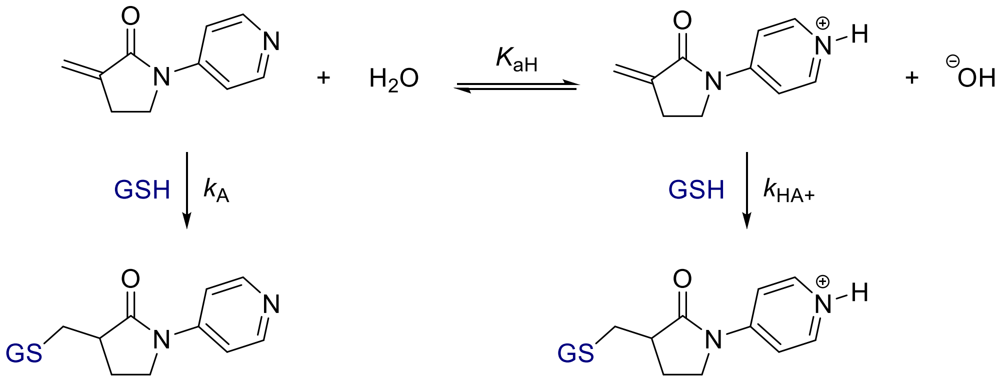

(eqn S5)

$$\frac{[\mathrm{A}]}{[\mathrm{HA}^+]} = 10^{(\mathrm{pH}\,-\,\mathrm{p}K_{\mathrm{aH}})} \quad \text{(eqn S6)}$$

$$[\mathrm{A}]_{\mathrm{rel}} = \frac{[\mathrm{A}]}{[\mathrm{A}] + [\mathrm{HA}^+]} \quad \text{(eqn S7)}$$

$$[\mathrm{HA}^+]_{\mathrm{rel}} = \frac{[\mathrm{HA}^+]}{[\mathrm{A}] + [\mathrm{HA}^+]} \quad \text{(eqn S8)}$$

$$k_{\mathrm{weighted,A}} = k_{\mathrm{A}} \times [\mathrm{A}]_{\mathrm{rel}} \quad \text{(eqn S9)}$$

$$k_{\mathrm{weighted,HA+}} = k_{\mathrm{HA+}} \times [\mathrm{HA}^+]_{\mathrm{rel}} \quad \text{(eqn S10)}$$

$$k_{\mathrm{total}} = k_{\mathrm{weighted,A}} + k_{\mathrm{weighted,HA+}} \quad \text{(eqn S11)}$$

$$\Delta G^{\ddagger}_{\mathrm{weighted}} = RT\,\ln\left(\frac{k_{\mathrm{B}}T}{h \times k_{\mathrm{total}}}\right) \quad \text{(eqn S12)}$$

Table S14. Values for calculating $\Delta G^{\ddagger}_{weighted}$ for **1e**, **2a**, and **3c**

	pK_{aH}	EA_A (eV)	EA_{AH+} (eV)	$\Delta G^{\ddagger}_{predicted,A}$ (kcal/mol)	$\Delta G^{\ddagger}_{predicted,AH+}$ (kcal/mol)	$\Delta G^{\ddagger}_{weighted}$ (kcal/mol)
**1e**	5.64	2.351	2.473	24.7	23.4	24.6
**2a**	5.40	2.482	2.912	23.3	18.8	21.6
**3c**	6.44	2.289	2.567	25.4	22.4	23.8

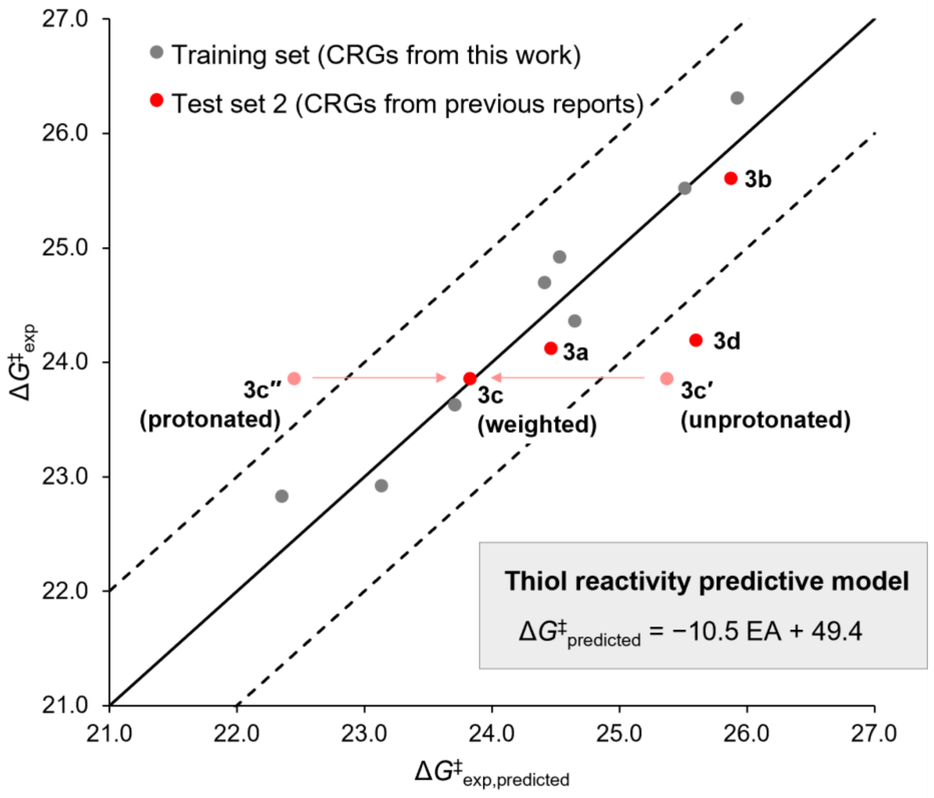

Figure S62. Example representation of $\Delta G^{\ddagger}_{weighted}$ for **3c** in test set 2. The protonated amine with one hydrogen bonding interaction was used to calculate EA for **3c″**.

CARTESIAN COORDINATES

methyl_thiolate

```
M06-2X SCF energy in solution (au):                        -438.199674965
M06-2X zero point corrected energy in solution (au):        -438.162588965
M06-2X free energy in solution (qh) (au):                   -438.186228

Cartesian coordinates
ATOM  X              Y            Z
C    -0.000000     0.000000    -1.096400
H     0.000000     1.008806    -1.453066
H    -0.873651    -0.504403    -1.453066
H     0.873651    -0.504403    -1.453066
S     0.000000    -0.000000     0.683600
```

1a

```
M06-2X SCF energy in solution (au):                        -571.735845329
M06-2X zero point corrected energy in solution (au):        -571.548804329
M06-2X free energy in solution (qh) (au):                   -571.587859

Cartesian coordinates
ATOM  X              Y            Z
C    -2.527520     1.398105     0.278106
C    -1.029115     1.624509     0.057883
H    -2.811242     1.619173     1.307870
H    -3.137531     2.012659    -0.380501
H    -0.573105     2.200705     0.864904
H    -0.845941     2.131924    -0.893962
C    -1.378505    -0.697101    -0.069048
O    -1.163754    -1.896413    -0.196477
C    -2.713762    -0.062310     0.005958
C    -3.844027    -0.739489    -0.147043
H    -3.813082    -1.799751    -0.332503
H    -4.808199    -0.266327    -0.092201
N    -0.441460     0.289125     0.018760
C     0.942592     0.124343     0.002043
C     1.792424     1.211912    -0.215335
C     1.544631    -1.121567     0.221519
C     3.160947     1.008298    -0.207759
H     1.403077     2.200347    -0.394411
H     0.944294    -2.002045     0.393270
H     3.838229     1.829977    -0.375780
C     3.644233    -0.269315     0.021040
H     4.703651    -0.481036     0.040787
N     2.849816    -1.308929     0.231261
```

TS1a

M06-2X SCF energy in solution (au): -1009.93306587
M06-2X zero point corrected energy in solution (au): -1009.70819987
M06-2X free energy in solution (qh) (au): -1009.752974
Imaginary frequency: -188.7 cm^{-1}

Cartesian coordinates

ATOM	X	Y	Z
C	1.371371	1.568265	-1.253698
C	-0.014280	1.729179	-0.607539
H	1.346236	1.888802	-2.302906
H	2.116378	2.184043	-0.737134
H	-0.665554	2.426141	-1.140517
H	0.076957	2.064344	0.434214
C	0.441825	-0.578600	-0.723903
O	0.267874	-1.794378	-0.485258
C	1.646019	0.091502	-1.112672
C	2.866552	-0.544572	-1.177503
H	2.886506	-1.629739	-1.184077
H	3.696856	-0.060028	-1.679269
N	-0.585177	0.375346	-0.643558
C	-1.876225	0.130277	-0.173074
C	-2.589547	1.114998	0.522204
C	-2.537507	-1.085931	-0.430149
C	-3.883558	0.835680	0.949491
H	-2.146017	2.083191	0.729179
H	-2.042048	-1.858275	-1.005179
H	-4.456775	1.579610	1.493531
C	-4.434877	-0.409063	0.672083
H	-5.441630	-0.656625	0.995333
N	-3.776305	-1.354068	-0.014674
S	4.032566	-0.387365	0.949629
C	2.612775	0.209117	1.903111
H	2.806038	1.187818	2.353695
H	1.744049	0.318409	1.231369
H	2.330034	-0.488879	2.697289

1b

M06-2X SCF energy in solution (au): -587.781799468
M06-2X zero point corrected energy in solution (au): -587.606283468
M06-2X free energy in solution (qh) (au): -587.645338

Cartesian coordinates

ATOM	X	Y	Z
C	2.521929	1.417557	0.137757
C	1.009095	1.626130	0.011758
H	3.072088	1.956653	-0.631088
H	2.887931	1.750302	1.109177
H	0.751812	2.129106	-0.924282
H	0.603565	2.200151	0.847171
C	1.374283	-0.704310	-0.022818
O	1.156051	-1.906611	-0.071884
C	2.704727	-0.062228	0.001420
C	3.835927	-0.751172	-0.083179
H	3.805959	-1.823747	-0.172325
H	4.799390	-0.274000	-0.065545
N	0.430407	0.285799	0.015037

C	-0.943192	0.111978	0.004585
C	-1.822682	1.198767	-0.097356
C	-1.564466	-1.142770	0.102922
N	-3.136003	1.048726	-0.101369
H	-1.481191	2.219460	-0.181285
N	-2.877459	-1.281063	0.100070
H	-0.988242	-2.052204	0.182824
C	-3.605469	-0.181480	-0.002066
H	-4.679237	-0.301900	-0.003867

TS1b
M06-2X SCF energy in solution (au): -1025.98074139
M06-2X zero point corrected energy in solution (au): -1025.76731839
M06-2X free energy in solution (qh) (au): -1025.812040
Imaginary frequency: -182.7 cm^{-1}

Cartesian coordinates

ATOM	X	Y	Z
C	-1.271771	1.965944	-0.314546
C	0.260784	1.932157	-0.392437
H	-1.629589	2.944407	0.018192
H	-1.710229	1.770481	-1.305179
H	0.706055	2.623063	0.335422
H	0.652990	2.167738	-1.384627
C	-0.454164	-0.030744	0.711215
O	-0.337925	-1.123984	1.309889
C	-1.577880	0.846884	0.646720
C	-2.799595	0.542715	1.219791
H	-2.833625	-0.235013	1.976259
H	-3.543941	1.324999	1.327255
N	0.594526	0.546440	-0.030963
C	1.905217	0.094311	-0.049768
C	2.258392	-1.260084	0.073811
C	2.972350	0.977184	-0.262304
N	3.527492	-1.667707	0.037316
H	1.498681	-2.021974	0.192222
N	4.243992	0.570576	-0.307600
H	2.798944	2.039236	-0.403804
C	4.462689	-0.732328	-0.142324
H	5.494522	-1.066589	-0.166837
S	-4.200942	-0.658389	-0.330466
C	-2.835661	-1.311171	-1.330351
H	-2.621488	-2.360160	-1.101765
H	-3.053741	-1.225650	-2.399364
H	-1.924357	-0.728805	-1.129547

1c
M06-2X SCF energy in solution (au): -876.460468777
M06-2X zero point corrected energy in solution (au): -876.295710777
M06-2X free energy in solution (qh) (au): -876.334874

Cartesian coordinates

ATOM	X	Y	Z
C	2.608788	1.354260	0.000413
C	1.123058	1.741643	0.001043
H	3.121854	1.738404	-0.880197
H	3.122206	1.736715	0.881563
H	0.841992	2.313096	-0.886757
H	0.842951	2.313228	0.889103
C	1.219351	-0.615159	0.000083
O	0.799092	-1.772370	-0.000307
C	2.615965	-0.147823	-0.000707
C	3.661307	-0.965761	-0.001617
H	3.510704	-2.031698	-0.002047
H	4.672906	-0.600870	-0.001937
N	0.415165	0.473667	0.001433
C	-0.957278	0.378461	0.000713
C	-1.873411	1.390889	-0.000707
S	-1.701138	-1.192120	0.001233
C	-3.199202	0.905450	-0.001947
H	-1.618289	2.433535	-0.000797
C	-3.262128	-0.448529	-0.000847
H	-4.061365	1.546545	-0.003397
H	-4.132348	-1.072345	-0.001367

TS1c
M06-2X SCF energy in solution (au): -1314.65822617
M06-2X zero point corrected energy in solution (au): -1314.45562217
M06-2X free energy in solution (qh) (au): -1314.500262
Imaginary frequency: -188.1 cm^{-1}

Cartesian coordinates

ATOM	X	Y	Z
C	1.322676	2.021360	-0.021267
C	-0.202812	2.037036	0.182065
H	1.659050	2.919282	-0.547842
H	1.838181	1.997193	0.950330
H	-0.707957	2.681889	-0.548349
H	-0.499403	2.353132	1.185287
C	0.400525	-0.085172	-0.682671
O	0.222421	-1.263437	-1.067359
C	1.561483	0.744578	-0.787515
C	2.756971	0.297199	-1.311618
H	2.750929	-0.608831	-1.909546
H	3.530090	1.015257	-1.563908
N	-0.604298	0.643915	-0.045806
C	-1.927870	0.273290	0.077422
C	-2.953811	1.087269	0.499694
S	-2.495868	-1.345785	-0.256018
C	-4.206530	0.400619	0.556849
H	-2.818842	2.130085	0.761051
C	-4.115503	-0.906623	0.185585
H	-5.133996	0.874220	0.860474
H	-4.903180	-1.647060	0.124538

S	4.160308	-0.649148	0.418639
C	2.800237	-1.023113	1.559288
H	1.893015	-0.492643	1.233077
H	2.569384	-2.092908	1.589490
H	3.030209	-0.689245	2.575986

1d
M06-2X SCF energy in solution (au): -876.461034483
M06-2X zero point corrected energy in solution (au): -876.296214483
M06-2X free energy in solution (qh) (au): -876.335318

Cartesian coordinates

ATOM	X	Y	Z
C	2.680080	1.355717	0.012071
C	1.172733	1.637143	0.000711
H	3.175719	1.780711	-0.859989
H	3.158125	1.764283	0.901791
H	0.868588	2.188862	-0.892439
H	0.855288	2.193883	0.885901
C	1.415040	-0.705572	-0.001889
O	1.123270	-1.899291	-0.005159
C	2.781569	-0.139689	0.000791
C	3.875681	-0.890013	-0.005749
H	3.789864	-1.963370	-0.013069
H	4.863561	-0.464833	-0.003989
N	0.537312	0.327337	-0.000449
C	-0.850168	0.213769	-0.000999
C	-1.536994	-0.967877	0.006931
C	-1.722389	1.341471	-0.009579
S	-3.235021	-0.724170	0.004521
H	-1.137684	-1.960402	0.014211
C	-3.028334	0.983428	-0.007519
H	-1.393110	2.362579	-0.016809
H	-3.890667	1.617719	-0.012179

TS1d
M06-2X SCF energy in solution (au): -1314.65778781
M06-2X zero point corrected energy in solution (au): -1314.45503481
M06-2X free energy in solution (qh) (au): -1314.499590
Imaginary frequency: -197.8 cm^{-1}

Cartesian coordinates

ATOM	X	Y	Z
C	-1.331055	1.959748	-0.227056
C	0.198792	1.879848	-0.329409
H	-1.655080	2.931041	0.157657
H	-1.788797	1.826715	-1.219230
H	0.682997	2.535575	0.407629
H	0.584930	2.130352	-1.320859
C	-0.549307	-0.101508	0.702010
O	-0.468620	-1.225480	1.249257
C	-1.662249	0.803807	0.681560

C	-2.882169	0.505044	1.248776
H	-2.936580	-0.314059	1.958879
H	-3.611782	1.294910	1.392237
N	0.501149	0.480590	-0.010514
C	1.824070	0.036070	-0.021236
C	2.887215	0.872446	-0.266554
C	2.239984	-1.331948	0.159471
S	4.385840	0.015478	-0.270973
H	2.873284	1.938571	-0.448951
C	3.590810	-1.477690	0.056589
H	1.559423	-2.150469	0.347687
H	4.164773	-2.391244	0.147968
S	-4.347046	-0.585725	-0.353274
C	-3.005290	-1.195032	-1.411971
H	-2.838065	-2.270089	-1.288984
H	-3.209391	-0.995354	-2.468709
H	-2.070304	-0.677005	-1.150906

1e

M06-2X SCF energy in solution (au): -588.986988685
M06-2X zero point corrected energy in solution (au): -588.789188685
M06-2X free energy in solution (qh) (au): -588.830155

Cartesian coordinates

ATOM	X	Y	Z
C	-2.396535	1.145788	-0.799323
C	-0.886180	1.205998	-1.060828
H	-2.746258	2.033047	-0.272189
H	-2.962218	1.061374	-1.725912
H	-0.445246	2.148762	-0.729088
H	-0.641724	1.061759	-2.117067
C	-1.258766	-0.667013	0.332386
O	-1.012172	-1.656419	1.013301
C	-2.587592	-0.074990	0.053102
C	-3.720230	-0.587931	0.513633
H	-3.698292	-1.476107	1.122044
H	-4.678488	-0.147944	0.301274
N	-0.333914	0.103249	-0.285439
C	1.030022	-0.188897	-0.312039
C	1.742249	-1.135986	-0.998769
C	3.142474	-0.044392	0.167981
H	1.384180	-1.879878	-1.681030
H	4.057692	0.293894	0.609429
N	3.059893	-1.030820	-0.689333
N	1.943981	0.508125	0.440588
C	1.661878	1.594871	1.338926
H	0.706310	1.416719	1.831204
H	2.448018	1.650521	2.088142
H	1.619770	2.540636	0.795675

TS1e

M06-2X SCF energy in solution (au): -1027.18582227
M06-2X zero point corrected energy in solution (au): -1026.94948227
M06-2X free energy in solution (qh) (au): -1026.995159
Imaginary frequency: -195.1 cm^{-1}

Cartesian coordinates

ATOM	X	Y	Z
C	1.200390	-1.801992	-1.142844
C	-0.229469	-1.316485	-1.449664
H	1.246567	-2.898166	-1.168211
H	1.910760	-1.425107	-1.887913
H	-0.847406	-2.068438	-1.944336
H	-0.220671	-0.408480	-2.067067
C	0.243714	-0.759767	0.795029
O	0.034385	-0.222523	1.906791
C	1.470171	-1.253534	0.240353
C	2.690025	-1.102301	0.859726
H	2.709788	-0.819994	1.907615
H	3.542035	-1.678499	0.516487
N	-0.783832	-1.003144	-0.118828
C	-2.034435	-0.392539	0.006543
C	-3.281332	-0.921299	0.221247
C	-3.550103	1.192793	0.031625
H	-3.535562	-1.962742	0.363051
H	-3.968986	2.189374	-0.013033
N	-4.227524	0.075419	0.234460
N	-2.225209	0.972363	-0.119052
C	-1.192334	1.973707	-0.340495
H	-0.649861	1.755474	-1.262970
H	-1.677095	2.946765	-0.428494
H	-0.496678	1.987635	0.501198
S	3.776468	0.955022	0.135466
C	2.335183	1.609576	-0.742915
H	1.982364	2.553005	-0.313410
H	2.539725	1.767728	-1.807021
H	1.511548	0.879865	-0.665386

1f

M06-2X SCF energy in solution (au): -908.791404347
M06-2X zero point corrected energy in solution (au): -908.600117347
M06-2X free energy in solution (qh) (au): -908.644788

Cartesian coordinates

ATOM	X	Y	Z
C	3.011682	-1.896749	-0.079926
C	1.531613	-1.512576	-0.001412
H	3.301308	-2.559186	0.733883
H	3.242461	-2.399444	-1.018870
H	1.053778	-1.867180	0.913858
H	0.946715	-1.880664	-0.845457
C	2.759635	0.507973	0.010668

O	3.008000	1.704055	0.031456
C	3.743898	-0.592439	0.001636
C	5.053899	-0.382169	0.056619
H	5.435290	0.623408	0.108587
H	5.763589	-1.189921	0.051408
N	1.510373	-0.054089	-0.008628
C	0.297997	0.595787	-0.011309
C	0.166889	1.993221	-0.022203
N	-0.762407	-0.211500	-0.006929
C	-1.099745	2.535375	-0.029171
H	1.047121	2.613396	-0.024543
C	-1.968565	0.335329	-0.019706
C	-2.212242	1.696251	-0.027770
H	-1.231407	3.605140	-0.036795
H	-3.217735	2.081076	-0.037770
C	-3.107573	-0.650278	0.005933
F	-4.211914	-0.184258	-0.607586
F	-2.815104	-1.818038	-0.579372
F	-3.495848	-0.950847	1.266159

TS1f
M06-2X SCF energy in solution (au): -1346.99092531
M06-2X zero point corrected energy in solution (au): -1346.76172631
M06-2X free energy in solution (qh) (au): -1346.811732
Imaginary frequency: -183.3 cm^{-1}

Cartesian coordinates

ATOM	X	Y	Z
C	-2.080017	-1.961033	-0.095621
C	-0.578668	-1.638605	-0.093660
H	-2.278320	-2.914415	-0.593988
H	-2.457337	-2.042498	0.935280
H	-0.066049	-2.113955	-0.937778
H	-0.072680	-1.933741	0.826377
C	-1.748798	0.306472	-0.778075
O	-1.911672	1.487984	-1.157830
C	-2.679199	-0.772950	-0.799761
C	-3.992208	-0.614533	-1.210199
H	-4.243080	0.259174	-1.803476
H	-4.594347	-1.495472	-1.408057
N	-0.530548	-0.176022	-0.250059
C	0.667686	0.507302	-0.168921
C	0.741367	1.921865	-0.164748
N	1.763155	-0.251842	-0.048494
C	1.980294	2.518542	-0.057196
H	-0.160594	2.512133	-0.232196
C	2.943291	0.364637	0.065400
C	3.134971	1.731221	0.059116
H	2.057612	3.601362	-0.049359
H	4.120556	2.170986	0.151469
C	4.108315	-0.583573	0.205904
F	4.234765	-1.387590	-0.867619

F	5.277191	0.060477	0.351695
F	3.970488	-1.393250	1.273089
S	-5.394912	0.016726	0.639229
C	-4.060072	0.687372	1.667116
H	-3.090077	0.354552	1.268653
H	-4.060206	1.781961	1.679812
H	-4.135536	0.326438	2.697483

1g

M06-2X SCF energy in solution (au): -924.828148917
M06-2X zero point corrected energy in solution (au): -924.648303917
M06-2X free energy in solution (qh) (au): -924.692766

Cartesian coordinates

ATOM	X	Y	Z
C	-4.020238	1.338276	-0.043092
C	-2.514203	1.621163	0.000406
H	-4.540036	1.789847	0.800238
H	-4.472251	1.720725	-0.957589
H	-2.232189	2.156699	0.909655
H	-2.184724	2.194170	-0.868390
C	-2.769790	-0.732071	0.004070
O	-2.485548	-1.917621	0.015111
C	-4.125735	-0.154354	0.003816
C	-5.221717	-0.904022	0.039066
H	-5.138500	-1.977035	0.068490
H	-6.207677	-0.475084	0.039719
N	-1.868430	0.310837	-0.008710
C	-0.500873	0.200996	-0.007966
C	0.331133	1.334923	0.022709
C	0.179212	-1.031995	-0.038625
N	1.647862	1.243830	0.021045
H	-0.047253	2.344381	0.051216
N	1.494768	-1.108864	-0.040854
H	-0.349210	-1.972513	-0.060498
C	2.166482	0.030966	-0.013527
C	3.670497	-0.078584	-0.000755
F	4.282066	1.085013	-0.250174
F	4.132536	-0.953198	-0.908983
F	4.137422	-0.499885	1.192412

TS1g

M06-2X SCF energy in solution (au): -1363.02859153
M06-2X zero point corrected energy in solution (au): -1362.81103753
M06-2X free energy in solution (qh) (au): -1362.861009
Imaginary frequency: -183.7 cm^{-1}

Cartesian coordinatesrATOM X Y Z

C	-2.869589	1.926274	-0.342697
C	-1.343313	2.081851	-0.396284
H	-3.349494	2.855888	-0.023806
H	-3.264619	1.673165	-1.338289

H	-0.997641	2.837962	0.320324
H	-0.966907	2.340572	-1.388759
C	-1.839838	0.054933	0.727298
O	-1.597874	-1.008966	1.340708
C	-3.054418	0.785471	0.623470
C	-4.246931	0.335050	1.171949
H	-4.198309	-0.429475	1.941234
H	-5.079737	1.025291	1.261887
N	-0.844452	0.757080	0.004368
C	0.500043	0.465386	0.003948
C	1.015036	-0.833432	0.201281
C	1.455414	1.461024	-0.258511
N	2.321056	-1.079764	0.179833
H	0.356210	-1.675561	0.363874
N	2.765547	1.207787	-0.283808
H	1.160363	2.486331	-0.453385
C	3.122683	-0.044061	-0.050908
C	4.597720	-0.373204	-0.063987
F	4.898550	-1.246376	-1.045488
F	4.983888	-0.950300	1.089664
F	5.366428	0.705300	-0.244933
S	-5.439866	-1.032311	-0.389214
C	-3.972913	-1.572303	-1.308528
H	-3.156802	-0.851575	-1.154004
H	-3.620826	-2.556426	-0.983192
H	-4.179624	-1.612755	-2.382258

1h

M06-2X SCF energy in solution (au): -588.967228884
M06-2X zero point corrected energy in solution (au): -588.769454884
M06-2X free energy in solution (qh) (au): -588.810704

Cartesian coordinates

ATOM	X	Y	Z
C	2.847201	1.223279	0.008086
C	1.365837	1.621081	0.002026
H	3.369496	1.606647	-0.867604
H	3.358982	1.596575	0.894366
H	1.095744	2.197669	-0.886924
H	1.088403	2.199594	0.887376
C	1.426131	-0.734455	-0.001324
O	1.031393	-1.899403	-0.003934
C	2.833508	-0.276925	0.000366
C	3.867605	-1.107551	-0.004454
H	3.701013	-2.171307	-0.009724
H	4.884921	-0.758384	-0.003424
N	0.638548	0.363845	0.000416
C	-0.750479	0.355678	-0.000784
C	-1.603970	-0.736012	0.004336
C	-1.606653	1.470821	-0.007444
H	-1.383844	-1.782444	0.009686
H	-1.363056	2.513066	-0.012974

N	-2.870617	1.081975	-0.006384
N	-2.856490	-0.244204	0.000596
C	-4.080326	-0.996033	0.005526
H	-4.903063	-0.286241	-0.010044
H	-4.131243	-1.639285	-0.872734
H	-4.141708	-1.611289	0.903106

TS1h

M06-2X SCF energy in solution (au): -1027.16224339
M06-2X zero point corrected energy in solution (au): -1026.92670839
M06-2X free energy in solution (qh) (au): -1026.973322
Imaginary frequency: -207.6 cm^{-1}

Cartesian coordinates

ATOM	X	Y	Z
C	1.558962	1.970513	0.048547
C	0.034212	2.050783	0.234273
H	1.947972	2.866762	-0.443220
H	2.058495	1.886810	1.025601
H	-0.431651	2.702062	-0.517768
H	-0.262852	2.401099	1.225954
C	0.537117	-0.076613	-0.647840
O	0.311186	-1.237059	-1.067390
C	1.738298	0.707358	-0.756964
C	2.896100	0.233391	-1.327331
H	2.843074	-0.655942	-1.947325
H	3.697345	0.923743	-1.567927
N	-0.413224	0.669869	0.026842
C	-1.769307	0.355227	0.095842
C	-2.421865	-0.847397	-0.150332
C	-2.797613	1.243257	0.477696
H	-2.068014	-1.818274	-0.457829
H	-2.720137	2.283568	0.762729
N	-3.986496	0.634608	0.464666
N	-3.736925	-0.621048	0.089263
C	-4.826300	-1.564780	-0.089870
H	-5.415093	-1.299369	-0.971007
H	-4.401190	-2.559616	-0.220884
H	-5.464177	-1.553622	0.795205
S	4.321657	-0.845065	0.332308
C	2.971325	-1.197960	1.492606
H	2.699449	-2.258695	1.499521
H	3.233020	-0.902808	2.513833
H	2.078697	-0.624881	1.200158

2a (protonated with one explicit H_2O molecule)

M062X SCF energy in solution (au): -648.632619349
M062X zero point corrected energy in solution (au): -648.408420349
M062X free energy in solution (qh) (au): -648.452554

Cartesian coordinates

ATOM	X	Y	Z
C	3.256301	1.352008	0.063635
C	1.747652	1.614162	0.003759
H	3.785294	1.826511	-0.761324
H	3.692044	1.721769	0.991619
H	1.476815	2.142868	-0.912924
H	1.409712	2.188928	0.867923
C	2.033059	-0.733310	-0.009296
O	1.770116	-1.922476	-0.029615
C	3.380830	-0.137448	-0.007957
C	4.485399	-0.873418	-0.061708
H	4.417988	-1.947227	-0.107359
H	5.467119	-0.434454	-0.062083
N	1.112665	0.297892	0.012119
C	-0.250830	0.176233	0.011997
C	-0.897821	-1.073486	0.058841
C	-1.074502	1.319524	-0.032249
C	-2.268816	-1.133643	0.055392
H	-0.319471	-1.980394	0.095660
C	-2.440122	1.177431	-0.033115
H	-0.659497	2.310330	-0.068853
N	-3.013981	-0.026328	0.007358
H	-2.781158	-2.081153	0.092941
H	-3.088812	2.037974	-0.066798
H	-4.057283	-0.103254	0.007143
O	-5.697082	-0.222441	0.014774
H	-6.283167	0.496489	-0.239457
H	-6.161538	-1.048321	-0.149895

2b

M062X SCF energy in solution (au): -892.517913772
M062X zero point corrected energy in solution (au): -892.364608772
M062X free energy in solution (qh) (au): -892.403652

Cartesian coordinates

ATOM	X	Y	Z
C	2.584987	1.353414	0.002229
C	1.101172	1.746322	-0.001415
H	3.101390	1.737393	-0.876313
H	3.096078	1.734842	0.885026
H	0.812408	2.315195	-0.886853
H	0.808394	2.317683	0.881063
C	1.199022	-0.618991	-0.000610
O	0.770806	-1.769222	-0.000627
C	2.592525	-0.148959	0.000457
C	3.637772	-0.968381	0.000072
H	3.487447	-2.034283	-0.000946
H	4.648836	-0.602404	0.000795
N	0.392554	0.477496	-0.001280
C	-0.963570	0.379619	-0.000735
S	-1.722062	-1.200711	0.000186
C	-3.063123	0.969982	0.000425

C	-3.242847	-0.372148	0.001102
H	-3.837015	1.714276	0.000797
H	-4.159537	-0.922788	0.002070
N	-1.770709	1.388230	-0.000542

2c

M062X SCF energy in solution (au): -587.779707428
M062X zero point corrected energy in solution (au): -587.604779428
M062X free energy in solution (qh) (au): -587.643880

Cartesian coordinates

ATOM	X	Y	Z
C	2.508273	1.443654	-0.073260
C	0.983298	1.640985	-0.025039
H	2.924465	1.850698	-0.998228
H	2.995577	1.949105	0.763645
H	0.602925	2.173128	-0.898166
H	0.661954	2.174340	0.871040
C	1.375368	-0.703774	0.025869
O	1.168192	-1.895628	0.074515
C	2.707862	-0.044996	-0.002180
C	3.835859	-0.753704	0.032685
H	3.804980	-1.839261	0.082455
H	4.809844	-0.272707	0.012781
N	0.422324	0.275923	-0.007901
C	-0.964938	0.090145	-0.001241
C	-1.575840	-1.177721	-0.078997
N	-1.691801	1.205463	0.069157
N	-2.899502	-1.302096	-0.077227
H	-0.988936	-2.081871	-0.146320
C	-3.019173	1.063980	0.071006
C	-3.626561	-0.181250	0.000618
H	-3.611641	1.971871	0.128559
H	-4.706737	-0.279213	0.003145

2d

M062X SCF energy in solution (au): -571.741622930
M062X zero point corrected energy in solution (au): -571.554858930
M062X free energy in solution (qh) (au): -571.593957

Cartesian coordinates

ATOM	X	Y	Z
C	-2.475613	-1.450441	0.002763
C	-0.952105	-1.601635	-0.000281
H	-2.926851	-1.909994	-0.875632
H	-2.922621	-1.905449	0.885735
H	-0.579150	-2.122517	-0.883666
H	-0.575567	-2.124975	0.880071
C	-1.393867	0.714958	-0.000223
O	-1.218561	1.928385	-0.000844
C	-2.704810	0.029685	0.000003
C	-3.858164	0.686348	-0.001854

H	-3.863041	1.763136	-0.003473
H	-4.806458	0.179203	-0.001823
N	-0.425626	-0.241719	0.000473
C	0.950596	-0.062532	0.000214
C	1.555014	1.198047	0.001633
N	1.654062	-1.194212	-0.001326
C	2.934856	1.257959	0.001323
H	0.951288	2.089406	0.002854
C	2.974690	-1.116173	-0.001605
C	3.670372	0.079643	-0.000350
H	3.430983	2.216568	0.002373
H	3.487484	-2.068119	-0.002844
H	4.747556	0.092024	-0.000616

2e

M062X SCF energy in solution (au): -725.357265045
M062X zero point corrected energy in solution (au): -725.123208045
M062X free energy in solution (qh) (au): -725.165740

Cartesian coordinates

ATOM	X	Y	Z
C	-3.530110	1.544813	0.387684
C	-2.014261	1.611543	0.184798
H	-3.792236	1.692644	1.436194
H	-4.059042	2.289840	-0.202525
H	-1.499081	2.074227	1.028168
H	-1.768165	2.158947	-0.730103
C	-2.628000	-0.630387	-0.140815
O	-2.558279	-1.831181	-0.376591
C	-3.881595	0.150227	-0.027172
C	-5.079357	-0.368446	-0.262301
H	-5.169628	-1.402766	-0.547993
H	-5.982102	0.209753	-0.176575
N	-1.584391	0.223897	0.052693
C	-0.224486	-0.097561	0.055713
C	0.194496	-1.435517	0.250452
C	0.728184	0.886640	-0.100690
C	1.518112	-1.763003	0.274278
H	-0.552517	-2.201737	0.374320
C	2.098597	0.572696	-0.079508
H	0.456558	1.918319	-0.253929
C	2.511200	-0.774526	0.109135
H	1.833944	-2.782612	0.424102
C	3.108371	1.545499	-0.242583
C	4.419691	1.156515	-0.213711
H	2.831730	2.579701	-0.388839
C	4.714818	-0.204770	-0.019495
H	5.221884	1.866556	-0.335697
H	5.742831	-0.541971	0.008630
N	3.811260	-1.134439	0.136202

2f
M062X SCF energy in solution (au): -1030.08445183
M062X zero point corrected energy in solution (au): -1029.87212683
M062X free energy in solution (qh) (au): -1029.914792

Cartesian coordinates

ATOM	X	Y	Z
C	3.482449	-0.724009	0.928594
C	2.140508	-1.448105	0.786179
H	3.604543	-0.324828	1.936560
H	4.328339	-1.373240	0.714310
H	1.699513	-1.732581	1.742786
H	2.241560	-2.343354	0.164883
C	1.962780	0.534576	-0.463022
O	1.488674	1.390202	-1.202041
C	3.380872	0.407337	-0.050657
C	4.344189	1.193912	-0.510188
H	4.110771	1.975278	-1.213436
H	5.369491	1.083329	-0.204529
N	1.278228	-0.480491	0.122253
C	-0.089737	-0.729375	-0.021792
C	-1.113925	0.272001	0.116775
C	-2.395526	-0.276725	-0.071429
C	-1.009137	1.626363	0.439028
C	-3.545663	0.491716	0.023188
C	-2.151771	2.389583	0.534493
H	-0.039269	2.070001	0.597237
C	-3.411947	1.831194	0.322751
H	-4.513511	0.042068	-0.131446
H	-2.071025	3.437360	0.779342
H	-4.290504	2.452120	0.400556
S	-2.323871	-1.987509	-0.385348
C	-0.600662	-1.962147	-0.274941
H	-0.069559	-2.882127	-0.413993

2g
M062X SCF energy in solution (au): -1030.08714526
M062X zero point corrected energy in solution (au): -1029.87493326
M062X free energy in solution (qh) (au): -1029.917698

Cartesian coordinates

ATOM	X	Y	Z
C	3.675886	1.426965	0.000996
C	2.176393	1.755754	0.000220
H	4.174681	1.831289	-0.878760
H	4.172875	1.828811	0.882932
H	1.874295	2.314972	-0.888393
H	1.873553	2.316250	0.887770
C	2.364965	-0.596547	0.000395
O	1.995171	-1.769023	0.000568
C	3.741736	-0.073274	-0.000645
C	4.817572	-0.850802	-0.002904

H	4.707575	-1.921687	-0.003844
H	5.814471	-0.447567	-0.003915
N	1.516617	0.461164	0.000829
C	-0.784206	1.306373	0.000428
C	-2.136548	-0.611885	0.000435
C	-2.112748	0.795481	0.000123
H	-0.556585	2.355495	0.000387
C	-3.330458	-1.314645	0.000059
C	-3.322805	1.493086	-0.000647
C	-4.514118	-0.603477	-0.000836
H	-3.324568	-2.393633	0.000228
C	-4.507589	0.791172	-0.001259
H	-3.316834	2.572134	-0.000970
H	-5.454243	-1.133005	-0.001500
H	-5.446454	1.323549	-0.002264
S	-0.525256	-1.296957	0.000937
C	0.149859	0.319921	0.000798

2h

M062X SCF energy in solution (au): -725.352632456
M062X zero point corrected energy in solution (au): -725.118317456
M062X free energy in solution (qh) (au): -725.161184

Cartesian coordinates

ATOM	X	Y	Z
C	-3.391257	0.468440	1.042966
C	-2.087471	1.273880	1.033598
H	-3.490020	-0.103617	1.966589
H	-4.268636	1.103360	0.941588
H	-1.640547	1.378668	2.023816
H	-2.243572	2.271814	0.613605
C	-1.831598	-0.435077	-0.563208
O	-1.319084	-1.117400	-1.443654
C	-3.244617	-0.469869	-0.117977
C	-4.168287	-1.227975	-0.692443
H	-3.903468	-1.861342	-1.522101
H	-5.190807	-1.238287	-0.358923
N	-1.194636	0.503952	0.180364
C	0.155300	0.859461	0.019106
C	1.180249	-0.119111	0.135856
C	0.511444	2.161771	-0.252689
C	0.957854	-1.460338	0.507613
C	2.513006	0.311471	-0.103372
H	-0.230784	2.943210	-0.330826
C	2.005371	-2.335671	0.592621
H	-0.045194	-1.792783	0.724026
C	3.570413	-0.617074	-0.014391
C	3.317748	-1.917830	0.318802
H	1.826043	-3.361260	0.876761
H	4.575306	-0.275962	-0.211301
H	4.124099	-2.631764	0.384416
C	2.724688	1.673587	-0.406042

H	3.725761	2.035432	-0.599350
N	1.771410	2.564298	-0.460606

2i

M062X SCF energy in solution (au): -553.480947997
M062X zero point corrected energy in solution (au): -553.313089997
M062X free energy in solution (qh) (au): -553.351727

Cartesian coordinates

ATOM	X	Y	Z
C	-2.289467	1.424724	0.172224
C	-0.782634	1.642401	-0.047047
H	-2.565815	1.716569	1.190368
H	-2.891609	2.010483	-0.524637
H	-0.338136	2.297409	0.704488
H	-0.562150	2.043845	-1.041134
C	-1.135630	-0.699047	0.040189
O	-0.875787	-1.907422	0.043065
C	-2.480032	-0.059396	-0.004407
C	-3.595827	-0.762512	-0.187956
H	-3.556570	-1.844224	-0.291536
H	-4.568764	-0.281864	-0.240957
N	-0.210258	0.293086	0.068132
C	1.169504	0.081416	0.026854
C	1.856535	-1.098357	0.080599
C	2.148237	1.130770	-0.084732
O	3.197833	-0.836341	0.010811
H	1.576149	-2.134018	0.166928
C	3.353640	0.511242	-0.089286
H	1.965994	2.194074	-0.154050
H	4.370388	0.868775	-0.156020

SUMMARY OF MEASURED RATES OF GSH ADDITION FOR TEST SET

Table S15. Summary of measured rates of GSH addition, half-lives, experimentally and DFT-calculated activation free energies for **2a–2i**. $\Delta G^{\ddagger}$ values are given in kcal/mol. [a]Blue dot indicates the location of substitution on the lactam CRG. [b]Calculated from the average half-life ($t_{1/2}$). [c]Weighted $\Delta G^{\ddagger}_{exp,pred}$. [d]EA for unprotonated species. [e]**2g** was dissolved in 100% DMSO-d$_6$.

Entry	Het/Aryl [a]	Compound	$t_{1/2}$ (min)	$k_{pseudo1st}$($\times 10^{-3}$ min^{-1})[b]	logk_{GSH} (M^{-1} s^{-1})	$\Delta G^{\ddagger}_{exp}$	$\Delta G^{\ddagger}_{exp,pred}$	EA (eV)
**1**	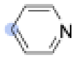	**2a**	4	173.29	-0.54	21.78	21.6[c]	2.48[d]
**2**	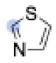	**2b**	39	18.00	-1.52	23.18	23.0	2.52
**3**	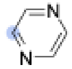	**2c**	77	9.00	-1.82	23.60	23.1	2.50
**4**	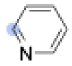	**2d**	131	5.31	-2.05	23.93	24.1	2.41
**5**	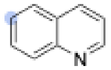	**2e**	733	0.95	-2.80	24.99	24.6	2.36
**6**	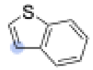	**2f**	1386	0.50	-3.08	25.39	25.0	2.33
**7**	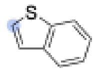	**2g**[e]	1412	0.49	-3.08	25.40	23.6	2.45
**8**	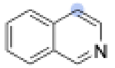	**2h**	–	–	–	–	–	2.39
**9**	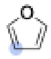	**2i**	–	–	–	–	–	2.27

PAPAIN PROTEASE INHIBITION ASSAY

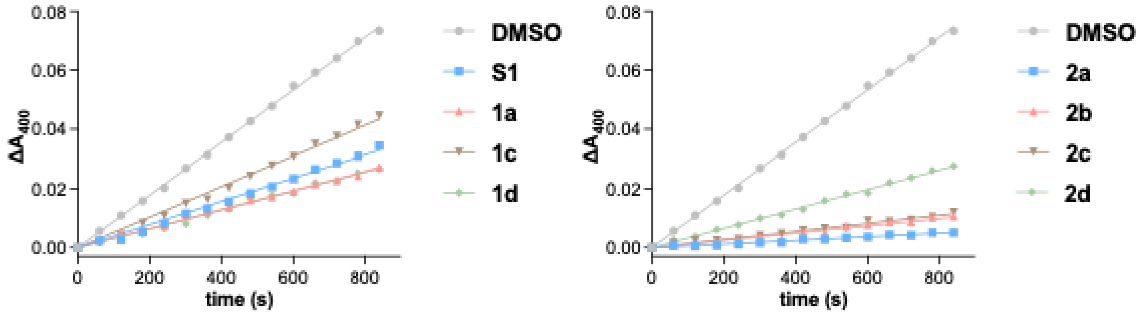

Figure S63. Progress curves for hydrolysis of a chromogenic substrate by papain (200 *μ*M) after incubation with the indicated compound (2 mM) for 22 h. Activity of papain incubated in parallel under identical conditions without compound is shown for comparison. Each treated enzyme sample was diluted 10-fold into a solution containing substrate (1 mM), and the change in absorbance at 400 nm measured as a function of time. Each data point is an average of three replicates. Linear regression was used to obtain apparent enzyme activity.

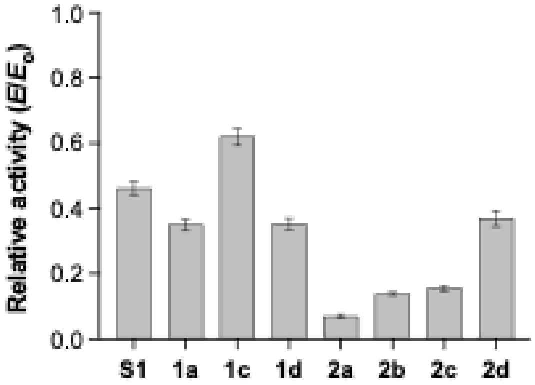

Figure S64. Fraction residual activity of papain (E/E_o) after incubation with the indicated *N*-(hetero)aryl *α*-methylene–*γ*-lactam for 22 h at a concentration of 200 *μ*M enzyme and 2 mM compound normalized to activity of a control sample incubated under identical conditions without compound. Each data point is the average and standard deviation from triplicate measurements.

HPLC PURITY

The purity for lactams **1a–1h** was determined using HPLC and are listed in Table S16. All compounds showed approximately 94% purity or greater except for **1e** (see Figure S69 for details). However, from the HPLC traces, the other peaks present are from instrument background noise, decreasing the calculated HPLC purity. HPLC purity was assessed using Agilent 1200 series HPLC with a G1365B Multi-Wavelength Detector and an Agilent Technologies Zorbax SB-C18 column (4.6 x 150 mm, 5 μm particles, P/N 883975-902) fitted with an Agilent Technologies Guard SB-C18 (P/N 820950-920).

Approximately 1 mg of each lactam was dissolved in DMSO (5.6 mM solution) in a 1-dram vial. 1.8 μL of the 5.6 mM solution was taken and diluted in 30% acetonitrile and water to make a 500 μM solution. This was diluted with an additional 10 μL for a final concentration of 0.5 mM solution. An aliquot (15 μL) of each solution was injected and eluted with a 30 min gradient method (5-95%) using water (solvent A) and acetonitrile (solvent B) and a 1 mL/minute flow rate allowed for detection (A_{280} or A_{260}) of lactams **1a–1h**. Oven temperature was set to 40 ºC.

Table S16. Purity of lactams **1a–1h** established by HPLC.

Compound	Purity (%)
**1a**	95.8
**1b**	94.7
**1c**	93.6
**1d**	97.2
**1e**	45.8
**1f**	95.0
**1g**	95.5
**1h**	97.0

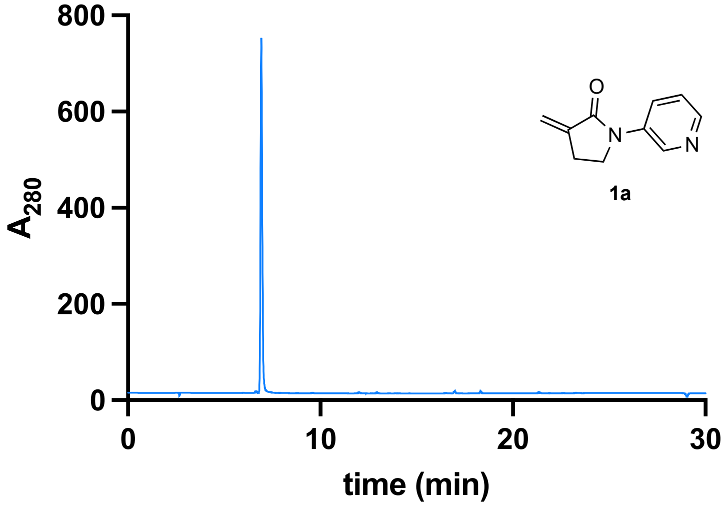

Entry	Time	Area
1	6.92981482	4626.21631
2	7.36866665	32.5989227
3	11.9891806	21.2161427
4	12.943182	14.9585886
5	16.9703827	27.7223759
6	18.3281898	27.5062084
7	21.351862	16.3727055
8	29.2522392	64.2830353

Figure S65. HPLC trace of **1a**. 95.8% pure.

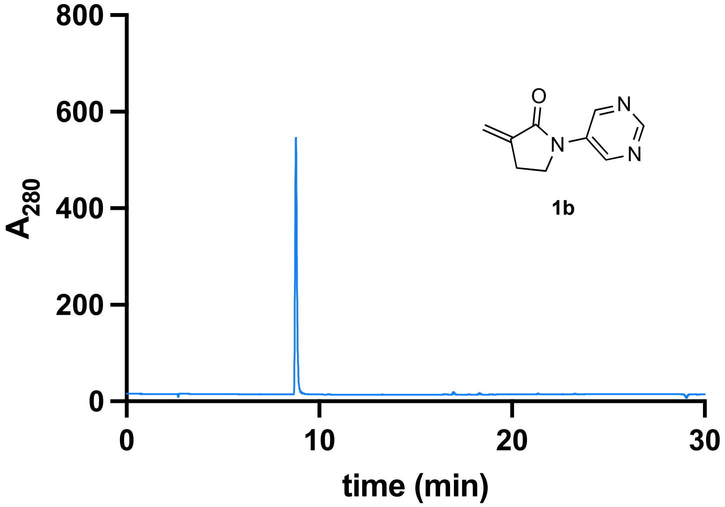

Entry	Time	Area
1	2.81999993	16.6002502
2	13.2485905	4512.55664
3	16.4933033	13.0753965
4	16.9632607	31.6621647
5	18.3199997	16.867588
6	29.2512074	52.3784409
7	29.2558613	81.6393661

Figure S66. HPLC trace of **1b.** 94.7% pure.

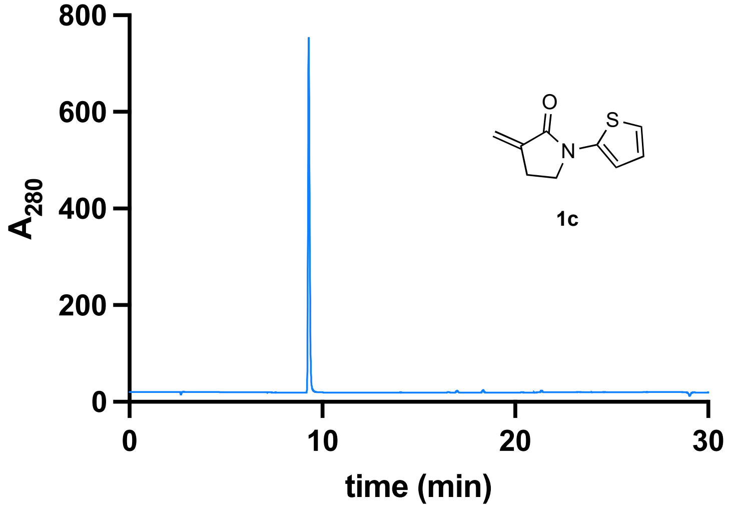

Entry	Time	Area
1	13.4003954	2445.94141
2	14.5634632	19.7434311
3	16.231905	21.2370605
4	16.9674911	38.4838943
5	18.3203278	15.2017813
6	18.6315556	16.3579617
7	29.2644367	54.9615059

Figure S67. HPLC trace of **1c**. 93.6% pure.

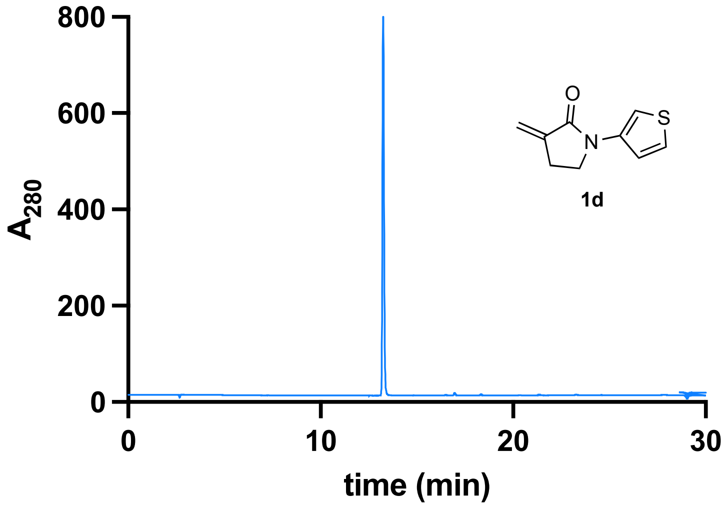

Entry	Time	Area
1	2.81999993	16.6002502
2	13.2485905	4512.55664
3	16.4933033	13.0753965
4	16.9632607	31.6621647
5	18.3199997	16.867588
6	29.2512074	52.3784409

Figure S68. HPLC trace of **1d.** 97.2% pure.

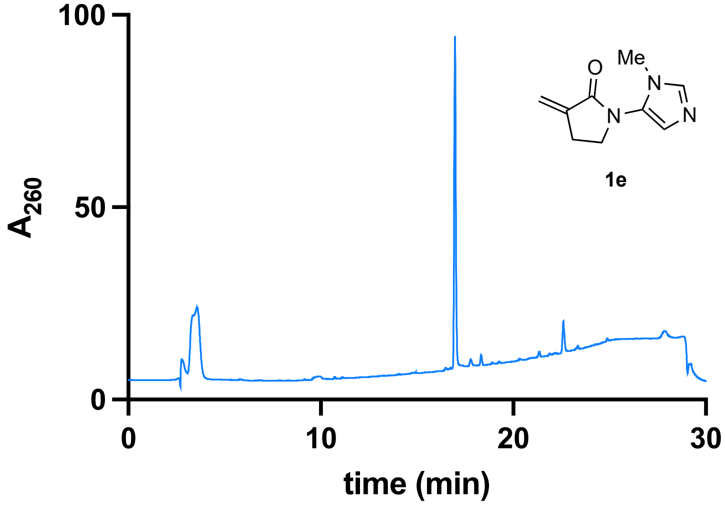

Entry	Time	Area
1	16.9703789	517.160706
2	17.7930603	23.096632
3	18.3286114	18.8253841
4	22.6001568	58.8312836
5	27.8720284	228.716736
6	28.8366661	245.565247
7	29.1470299	14.8177614
8	29.2327309	22.3507156

Figure S69. HPLC trace of **1e**. 45.8% pure. The DMSO peak at 2–4 min was excluded from analysis. Due to the poor absorbance of imidazoles,[33–34] the purity of **1e** is impacted by the signal-to-noise from 22–30 min. The thiol reactivity data and correlation in the predictive model supports that **1e** is behaving as expected.

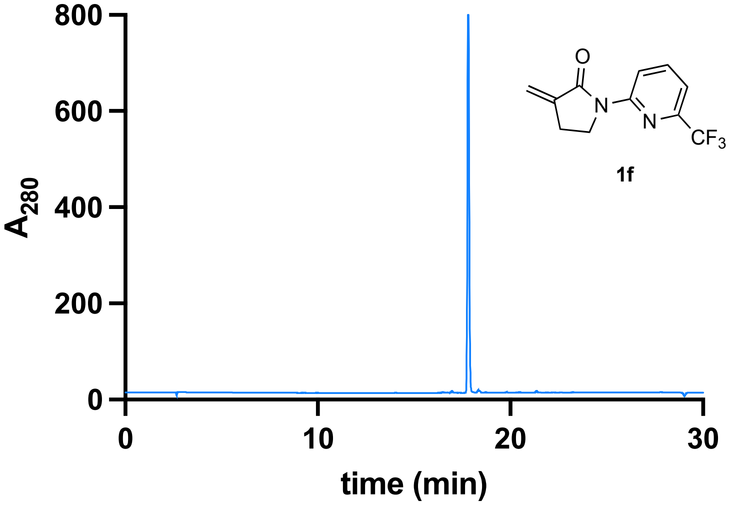

Entry	Time	Area
1	2.8211112	19.2356777
2	16.502903	13.251009
3	16.9663677	25.7254238
4	17.8111877	5446.55957
5	18.3233738	35.946415
6	18.4108105	18.7188149
7	19.7993031	11.1258822
8	20.4732838	9.32065201
9	21.3528709	25.0216007
10	29.2408333	126.482681

Figure S70. HPLC trace of **1f**. 95.0% pure.

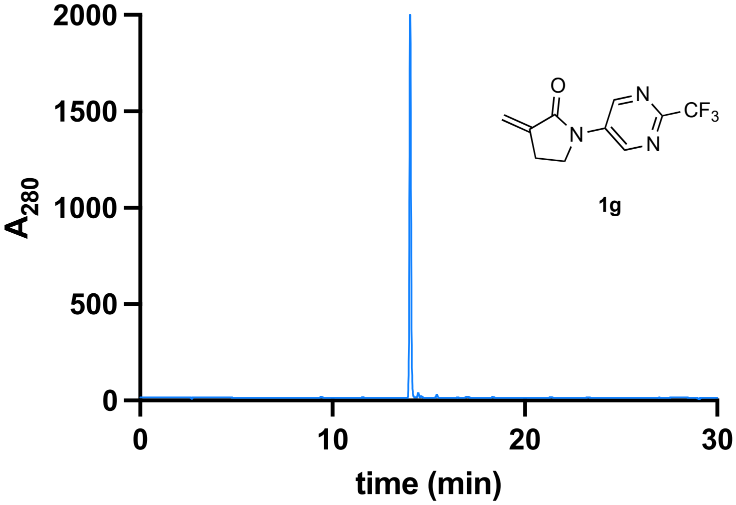

Entry	Time	Area
1	2.82555556	20.765564
2	9.41841412	36.245388
3	11.5717306	17.6645031
4	14.0354662	11783.0635
5	14.4563885	143.785934
6	14.6185694	55.2863922
7	15.4216394	83.5584335
8	16.9756718	22.8863239
9	17.047287	31.5677814
10	18.3273869	29.2740555
11	21.3580666	22.5961647
12	29.2472019	87.0249176

Figure S71. HPLC trace of **1g**. 95.5% pure.

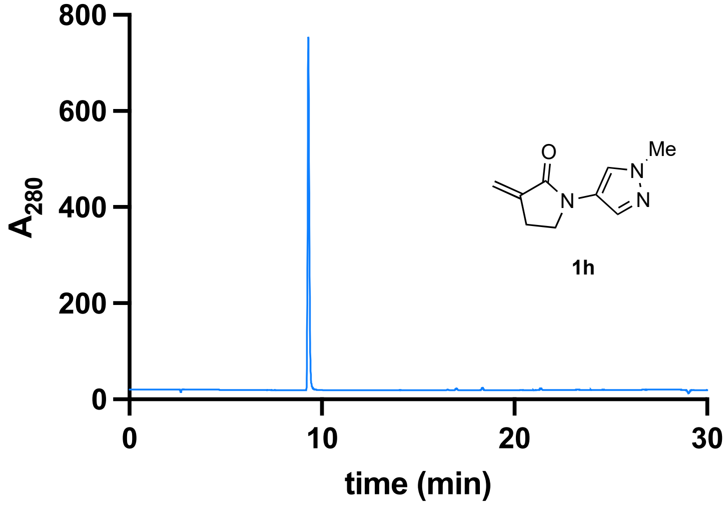

Entry	Time	Area
1	2.81916666	22.2186127
2	9.29581833	4327.30908
3	16.9799881	25.10396
4	18.3315525	30.5902767
5	21.3568268	22.7747211
6	29.2417583	34.6542931

Figure S72. HPLC trace of **1h**. 97.0% pure.

The purity for lactams **2a**–**2g** was determined using HPLC and are listed in Table S17. The DMSO peak at 2–4 min was excluded from analysis. All compounds showed approximately 95% purity or greater with the exception of **2g**. HPLC purity was assessed using a Shimadzu LC20AD HPLC with a Prominence SPD-20AV UV/Vis Detector and an Agilent Technologies Zorbax SB-C18 column (4.6 x 150 mm, 5 µm particles, P/N 883975-902) fitted with an Agilent Technologies Guard SB-C18 (P/N 820950-920).

Approximately 1 mg of each lactam was dissolved in DMSO (5.6 mM solution) in a 1-dram vial. 1.8 μL of the 5.6 mM solution was taken and diluted in 30% acetonitrile and water to make a 500 μM solution. This was diluted with an additional 10 μL for a final concentration of 0.5 mM solution. An aliquot (15 μL) of each solution was injected and eluted with a 30 min gradient method (5-95%) using water (solvent A) and acetonitrile (solvent B) and a 1 mL/minute flow rate allowed for detection (A_{280} or A_{260}) of lactams **2a**–**2g**. Oven temperature was set to 40 ºC.

Table S17. Purity of lactams **2a–2g** established by HPLC.

Compound	Purity (%)
**2a**	95.9
**2b**	95.9
**2c**	95.1
**2d**	96.2
**2e**	96.3
**2f**	96.3
**2g**	91.3

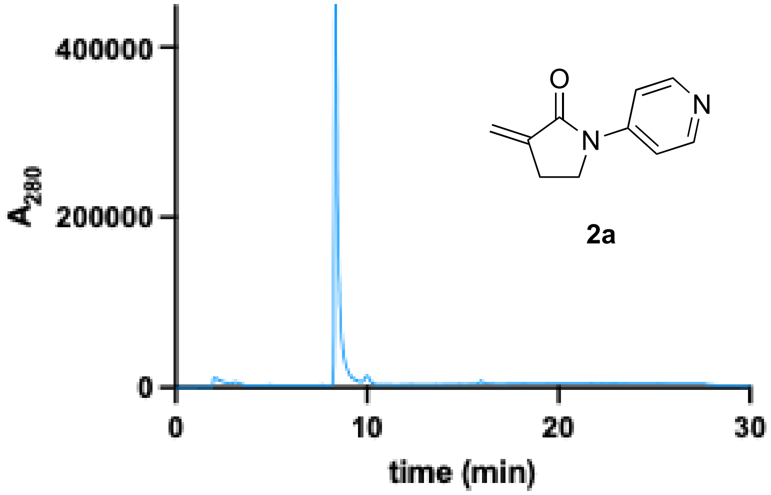

Entry	Time	Area
1	4.937	16449
2	5.566	6683
3	6.407	10066
4	7.027	4161
5	7.632	7116
6	7.989	635
7	8.352	5907113
8	9.99	155634
9	14.86	4296
10	15.374	1921
11	15.933	31580
12	18.15	2772
13	19.452	4326
14	22.964	2027
15	23.476	2290

Figure S73. HPLC trace for **2a**. 95.9% pure.

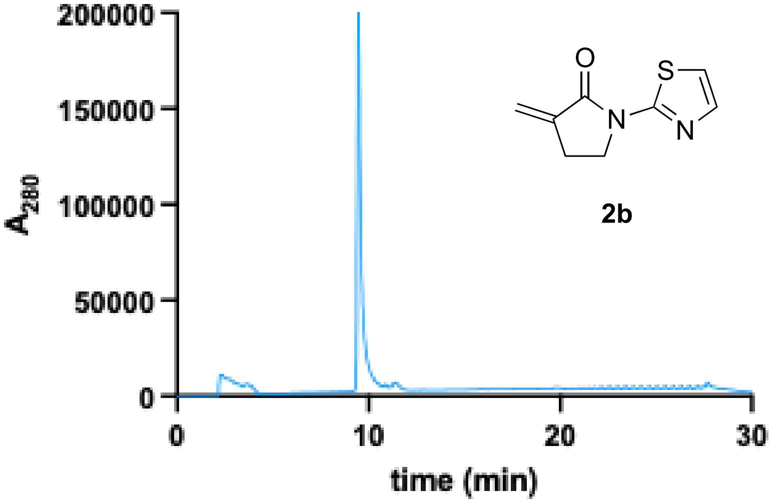

Entry	Time	Area
1	6	2753
2	6.569	821
3	9.459	2854659
4	10.876	9836
5	11.393	64665
6	12.859	3122
7	15.319	2580
8	15.786	5092
9	19.746	7955
10	27.409	2127
11	27.676	24633.000

Figure S74. HPLC trace for **2b**. 95.9% pure.

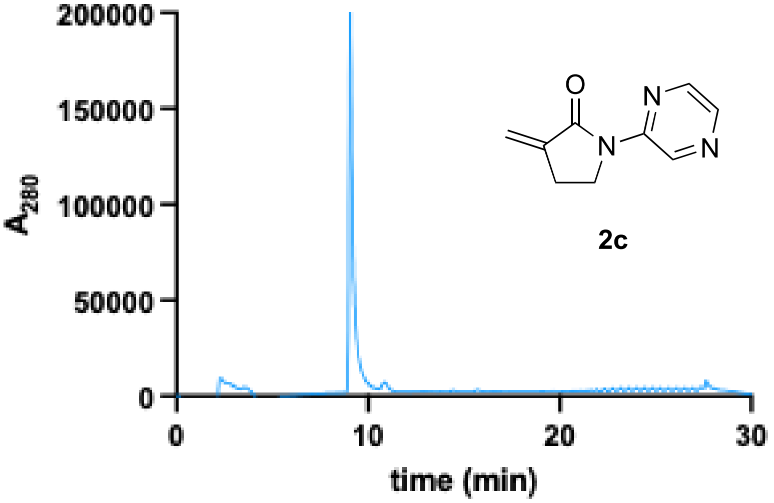

Entry	Time	Area
1	9.048	3072017
2	10.862	75243
3	14.421	9497
4	15.695	26790
5	27.485	1425
6	27.635	45708

Figure S75. HPLC trace for **2c**. 95.1% pure.

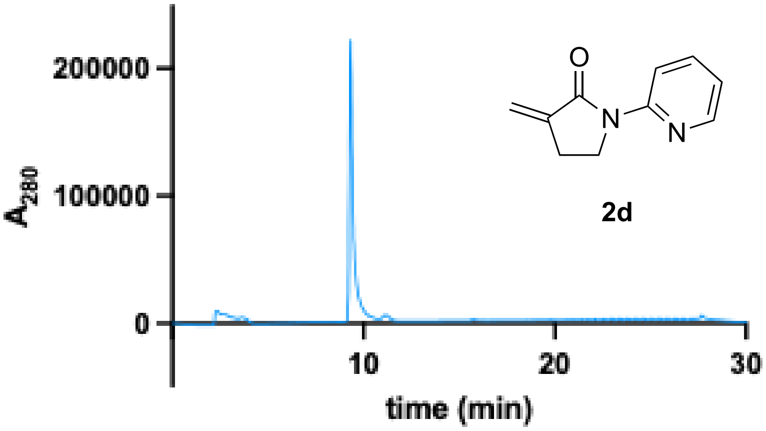

Entry	Time	Area
1	9.309	3178202
2	11.156	80423
3	15.712	13965
4	27.403	2569
5	27.536	661
6	27.669	25057

Figure S76. HPLC trace for **2d**. 96.2% pure.

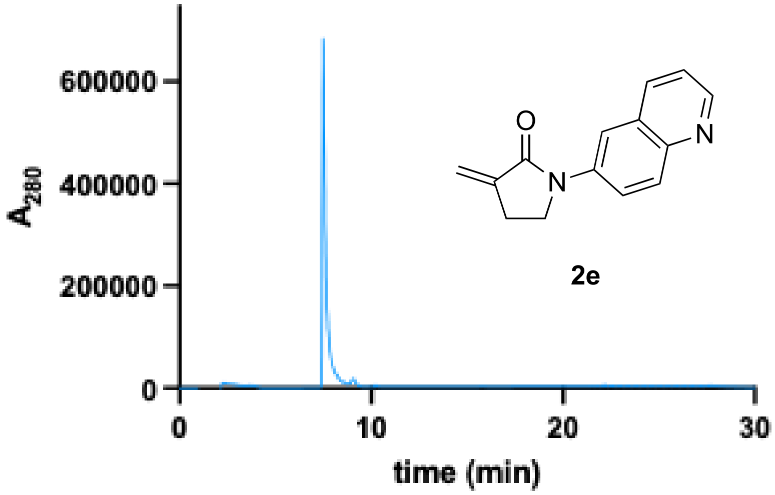

Entry	Time	Area
1	6.474	3210
2	7.486	8284180
3	8.587	12188
4	9.044	202358
5	10.212	31764
6	12.38	2951
7	15.718	19150
8	22.15	21865
9	27.402	2160
10	27.675	19229

Figure S77. HPLC trace for **2e**. 96.3% pure.

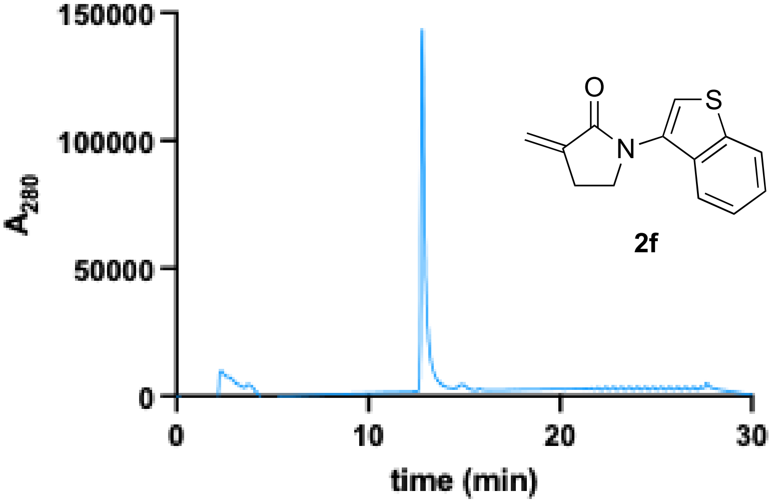

Entry	Time	Area
1	12.807	2140937
2	14.328	754
3	14.894	54057
4	15.768	7707
5	27.492	421
6	27.624	19210

Figure S78. HPLC trace for **2f**. 96.3% pure.

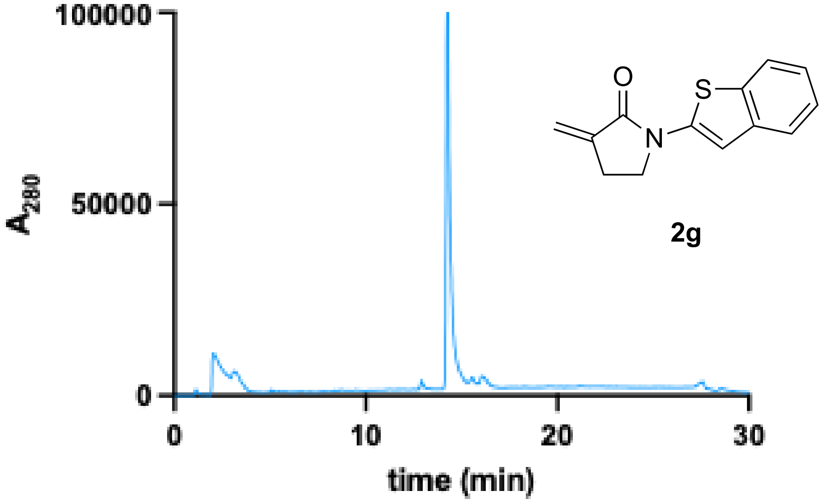

Entry	Time	Area
1	1.131	13444
2	5.069	4910
3	5.442	1794
4	5.729	608
5	6.605	2821
6	8.368	1993
7	8.673	3827
8	12.903	19179
9	14.26	1410979
10	15.553	19110
11	16.06	53094
12	21.205	1685
13	28.567	11308

Figure S79. HPLC trace for **2g**. 91.3% pure.

For biological activity:

The purity of the DMSO stock solutions for lactams **1a**, **1c**, **1d**, **2a–2d**, and **S1** used for the papain protease inhibition assay was determined using HPLC. All compounds showed ≥95% purity. HPLC purity was assessed using a Hitachi LaChrom Elite HPLC with a Hitachi L-2455 Diode Array Detector and a Phenomenex Luna C18 100 Å column (250 x 4.6 mm, 5 µm particles, P/N 00G-4041-E0) fitted with a Phenomenex SecurityGuard Cartridge (C18, 4 x 3.0 mm, P/N AJ0-4287).

Each 200 mM DMSO stock solution was diluted in 30% acetonitrile and water to make a 500 μM solution. An aliquot (15 μL) of each solution was injected and eluted with a 30 min gradient method (5-95%) using water (solvent A) and acetonitrile (solvent B) and a 1 mL/minute flow rate.

Additionally, the DMSO stock solutions were submitted to high-resolution mass spectrometry on a Fisher Scientific QExactive with an Orbitrap mass analyzer using ESI as the ionization source. In several spectra, artifacts from DMSO were observed, e.g. *m/z* = 157.0353–157.0354 corresponding to the $[M+H]^+$ peak for $(C_2H_6OS)_2$ and *m/z* = 179.0173 corresponding to the $[M+Na]^+$ peak for $(C_2H_6OS)_2$.

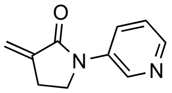

1a

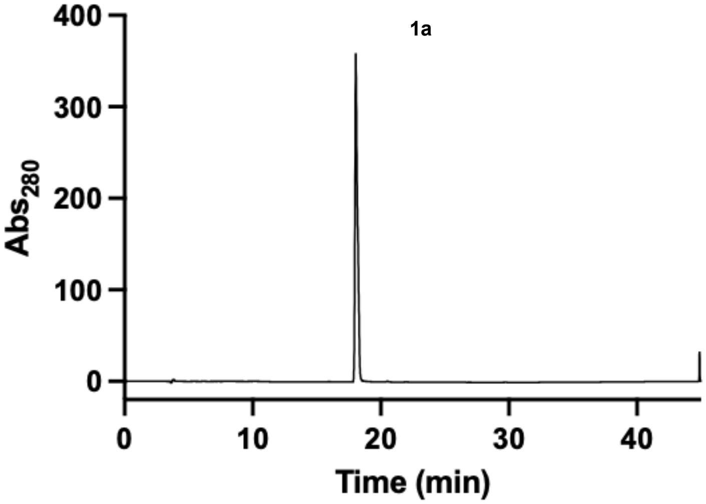

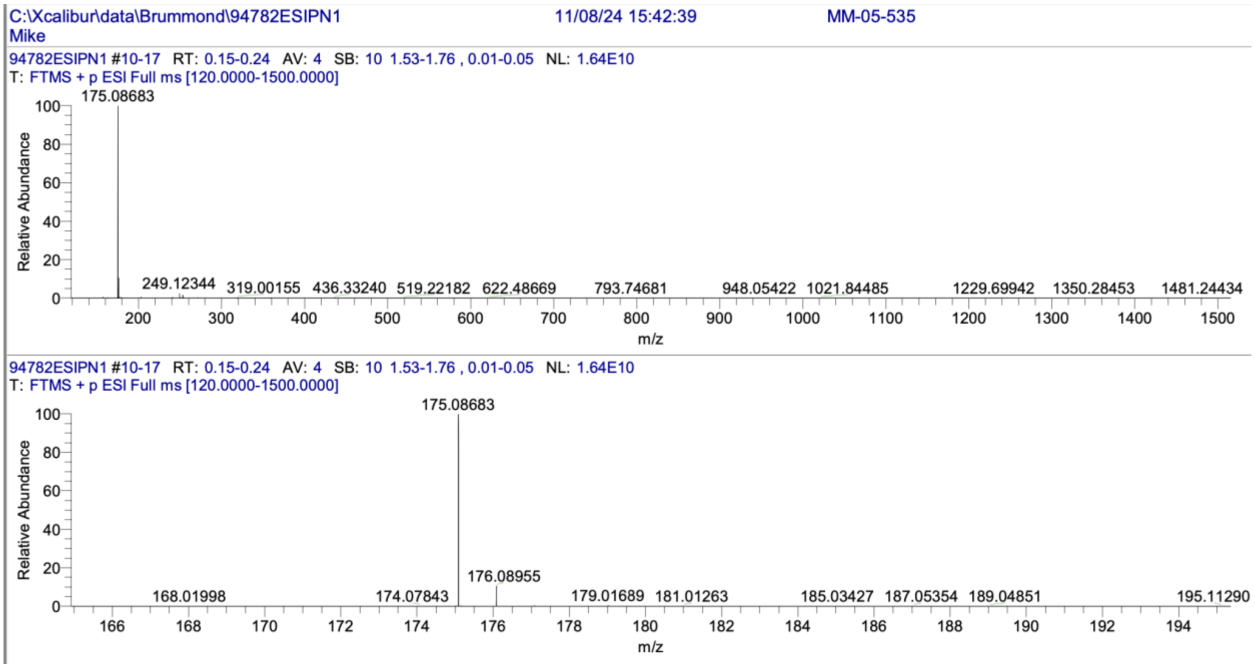

94782ESIPN1#11-17 RT: 0.15-0.24 AV: 4
SB: 10 1.53-1.76 , 0.01-0.05
T: FTMS + p ESI Full ms [120.0000-1500.0000]
m/z= 164.92199-195.35761

m/z	Intensity	Relative	Theo. Mass	Delta (ppm)	Composition
175.08683	16391095296.0	100.00	175.08659	1.38	$C_{10}H_{11}ON_2$

Figure S80. HPLC trace (top) and HRMS ESI-MS spectrum (bottom) for **1a**.

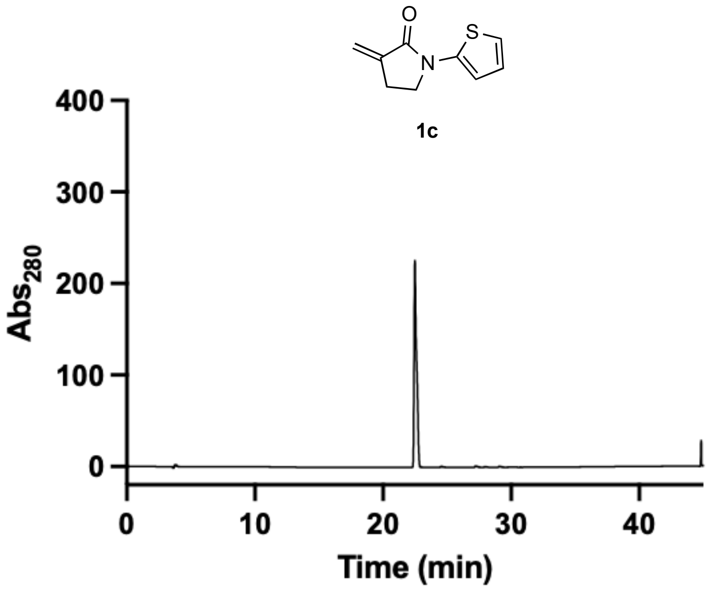

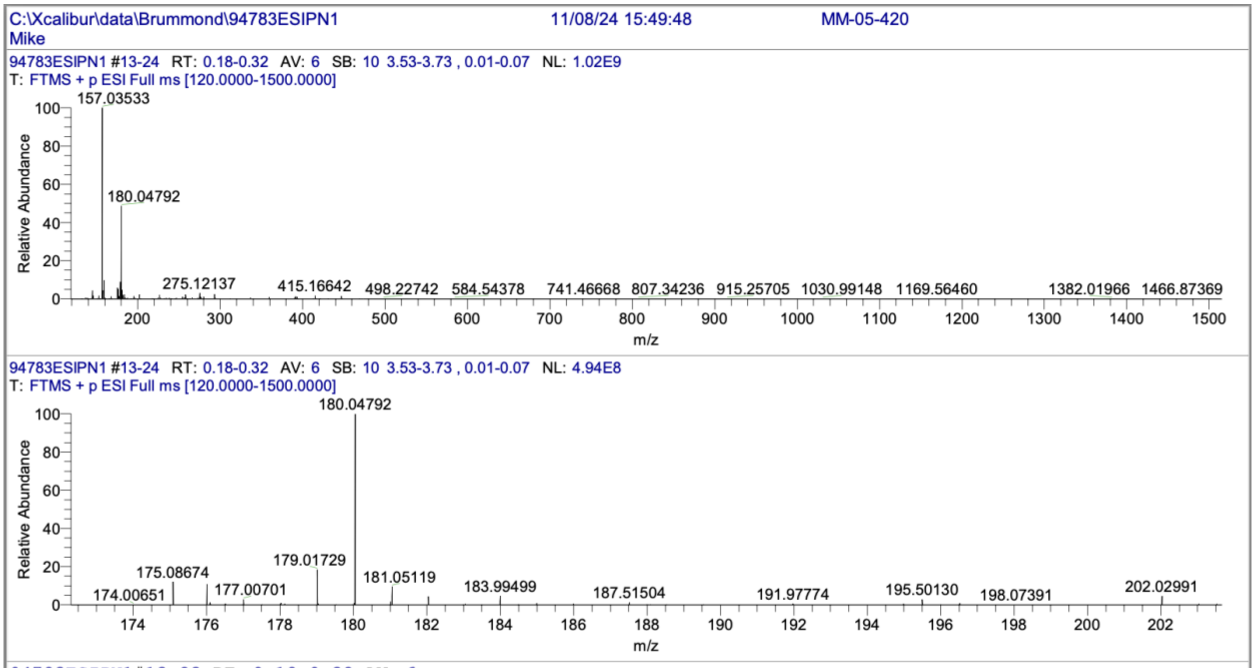

94783ESIPN1#13-23 RT: 0.18-0.32 AV: 6
SB: 10 3.53-3.73 , 0.01-0.07
T: FTMS + p ESI Full ms [120.0000-1500.0000]
m/z= 172.30030-203.65833

m/z	Intensity	Relative	Theo. Mass	Delta (ppm)	Composition
180.04792	495492224.0	100.00	180.04776	0.86	$C_9H_{10}ONS$

Figure S81. HPLC trace (top) and HRMS ESI-MS spectrum (bottom) for **1c**.

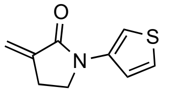

1d

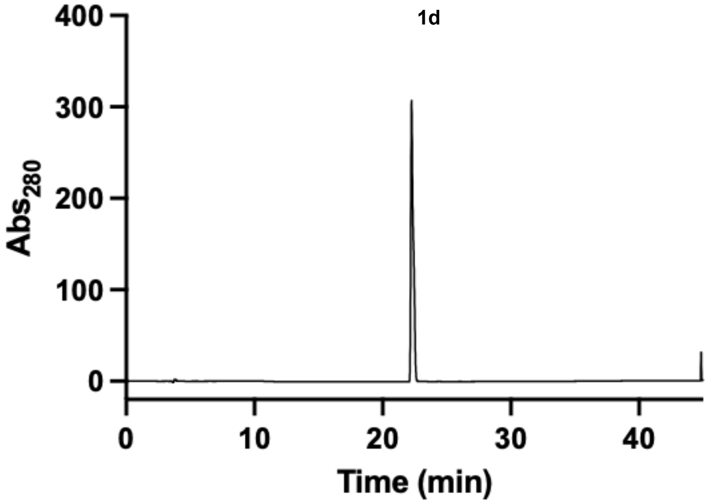

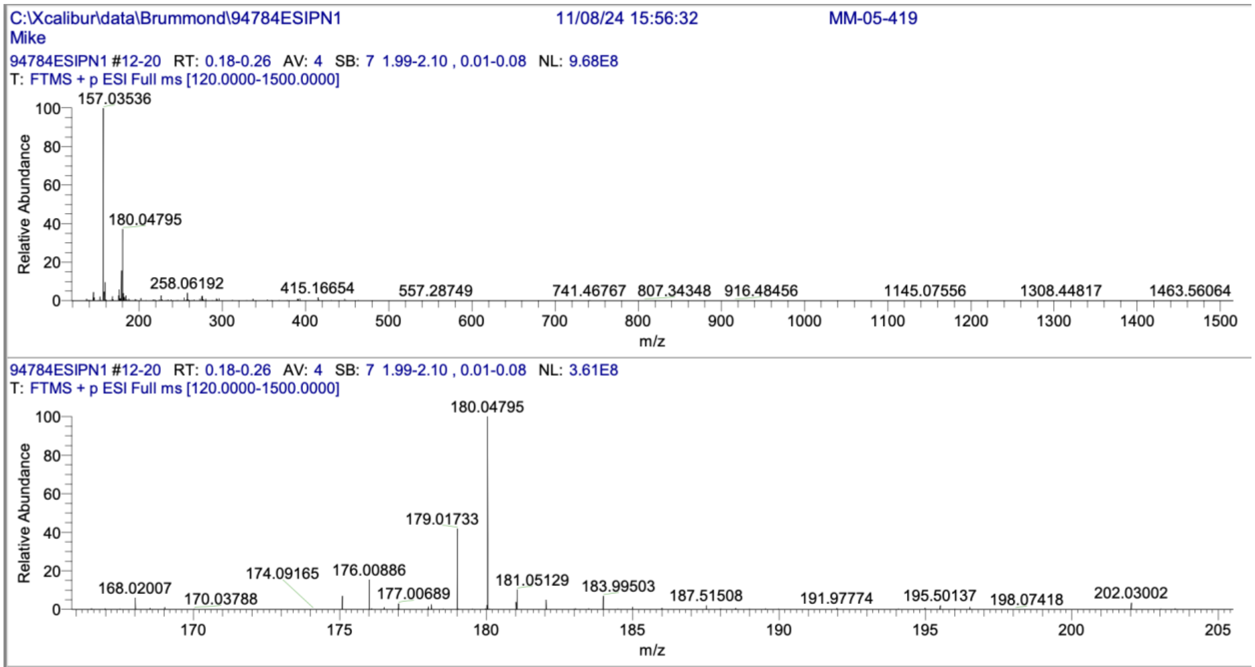

94784ESIPN1#13-19 RT: 0.18-0.26 AV: 4
SB: 7 1.99-2.10 , 0.01-0.08
T: FTMS + p ESI Full ms [120.0000-1500.0000]
m/z= 165.84365-205.50197

m/z	Intensity	Relative	Theo. Mass	Delta (ppm)	Composition
180.04795	371987040.0	100.00	180.04776	1.07	$C_9H_{10}ONS$

Figure S82. HPLC trace (top) and HRMS ESI-MS spectrum (bottom) for **1d**.

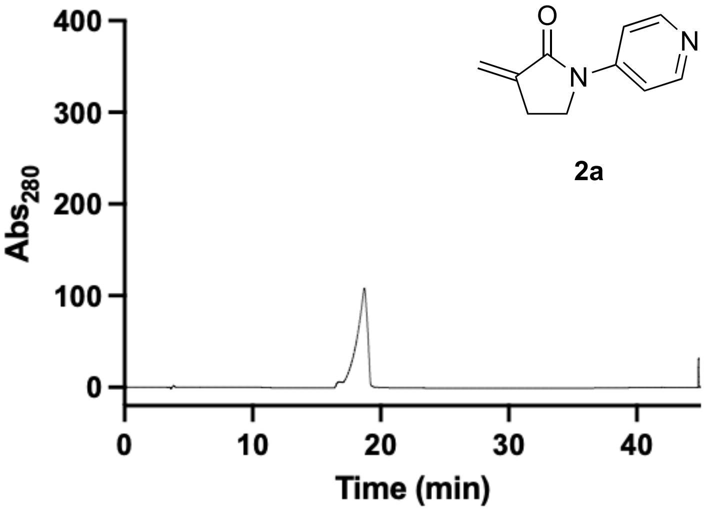

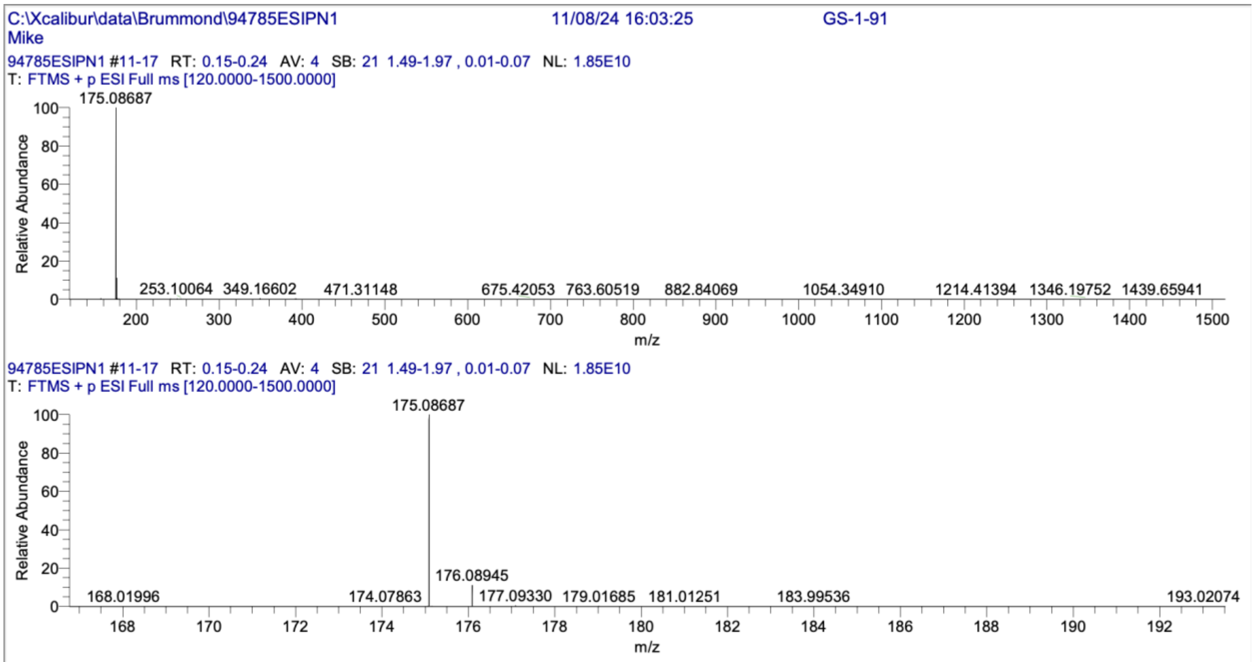

94785ESIPN1#11-17 RT: 0.15-0.24 AV: 4
SB: 21 1.49-1.97 , 0.01-0.07
T: FTMS + p ESI Full ms [120.0000-1500.0000]
m/z= 166.76633-193.51292

m/z	Intensity	Relative	Theo. Mass	Delta (ppm)	Composition
175.08687	19005882368.0	100.00	175.08659	1.61	$C_{10}H_{11}ON_2$

Figure S83. HPLC trace (top) and HRMS ESI-MS spectrum (bottom) for **2a**.

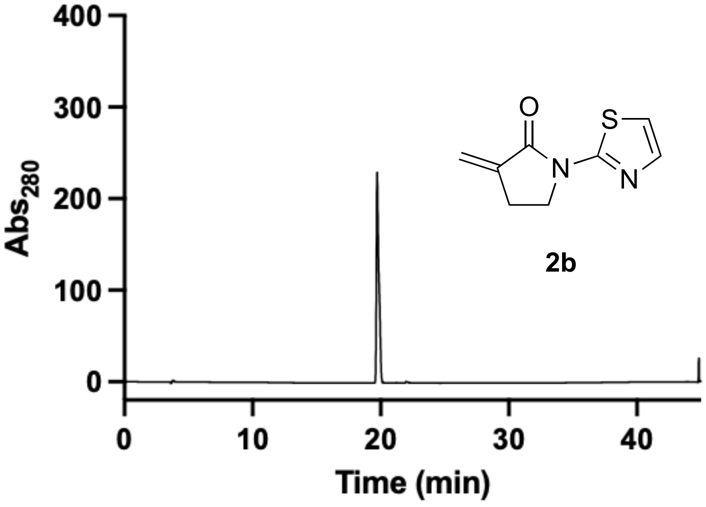

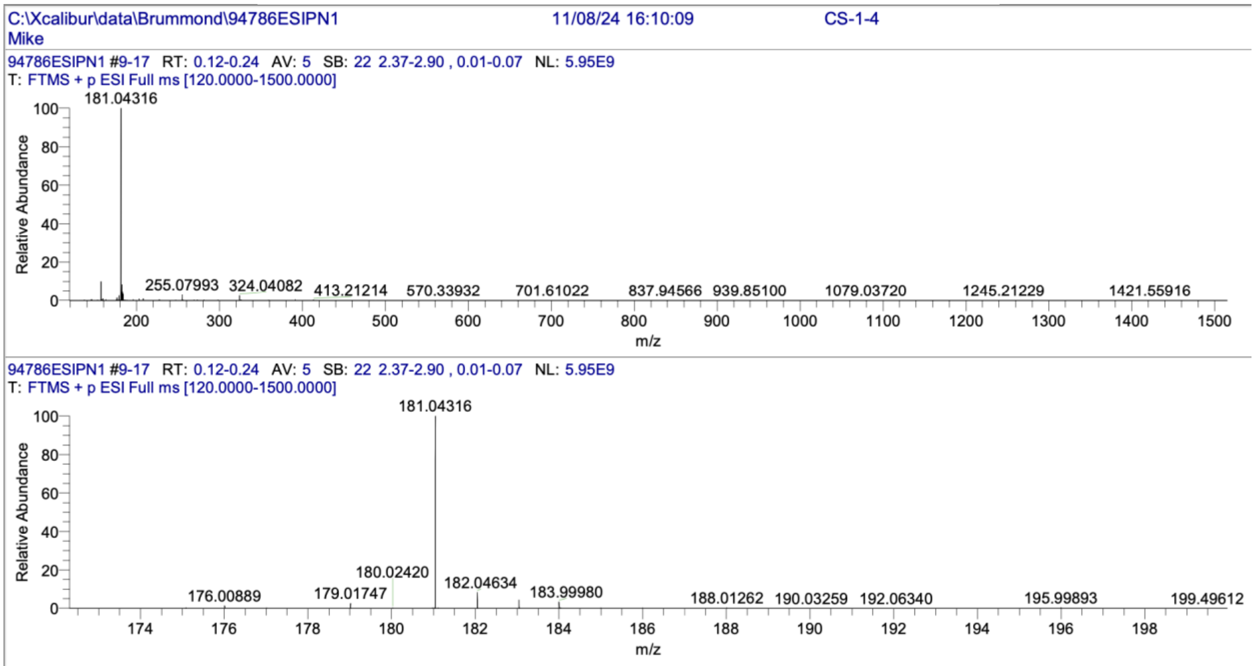

94786ESIPN1#9-17 RT: 0.12-0.24 AV: 5
SB: 22 2.37-2.90 , 0.01-0.07
T: FTMS + p ESI Full ms [120.0000-1500.0000]
m/z= 172.29946-199.96804

m/z	Intensity	Relative	Theo. Mass	Delta (ppm)	Composition
181.04316	5986863616.0	100.00	181.04301	0.82	$C_8H_9ON_2S$

Figure S84. HPLC trace (top) and HRMS ESI-MS spectrum (bottom) for **2b**.

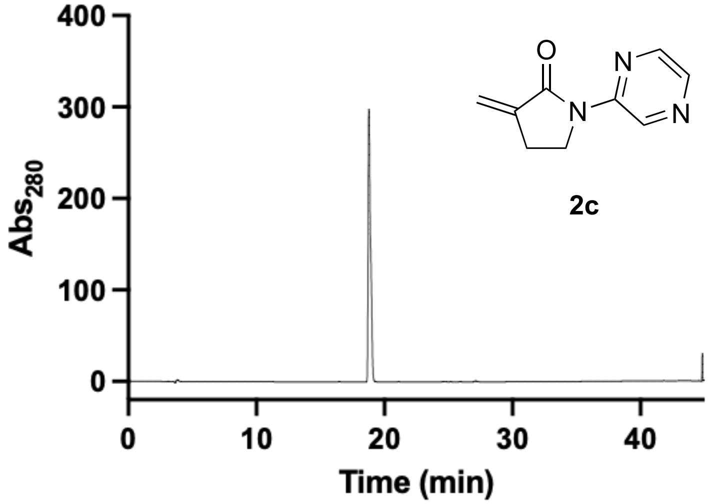

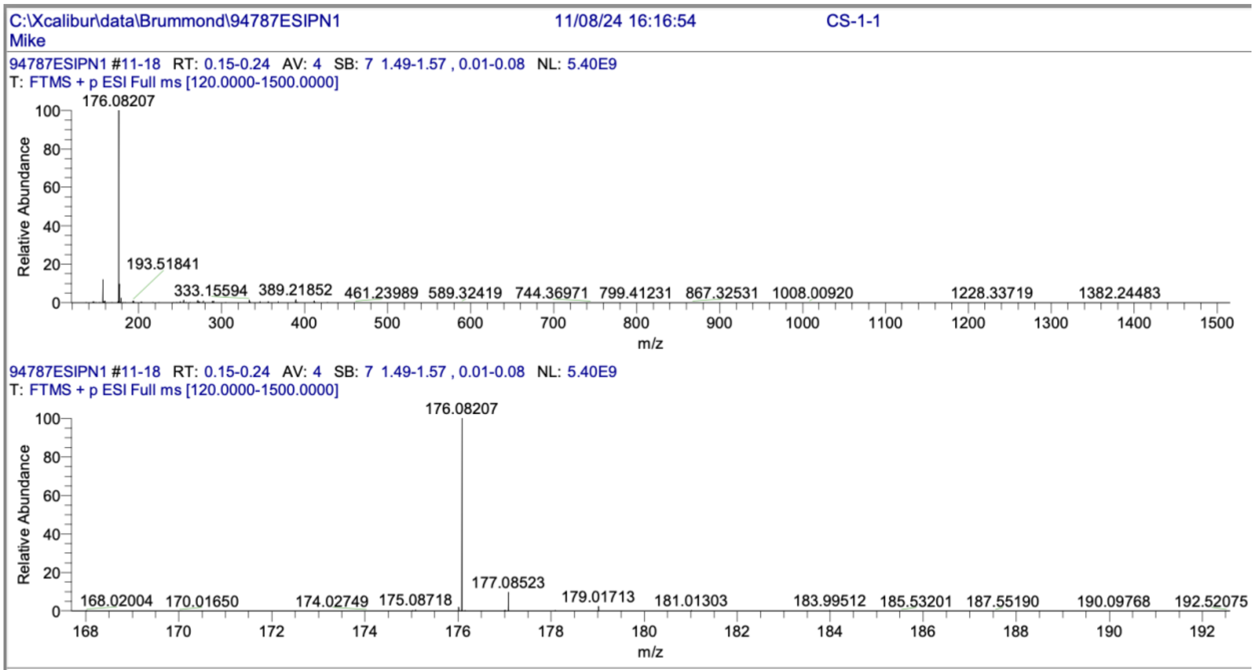

94787ESIPN1#11-17 RT: 0.15-0.24 AV: 4
SB: 7 1.49-1.57 , 0.01-0.08
T: FTMS + p ESI Full ms [120.0000-1500.0000]
m/z= 167.68823-192.59001

m/z	Intensity	Relative	Theo. Mass	Delta (ppm)	Composition
176.08207	5562454016.0	100.00	176.08184	1.31	$C_9 H_{10} O N_3$

Figure S85. HPLC trace (top) and HRMS ESI-MS spectrum (bottom) for **2c**.

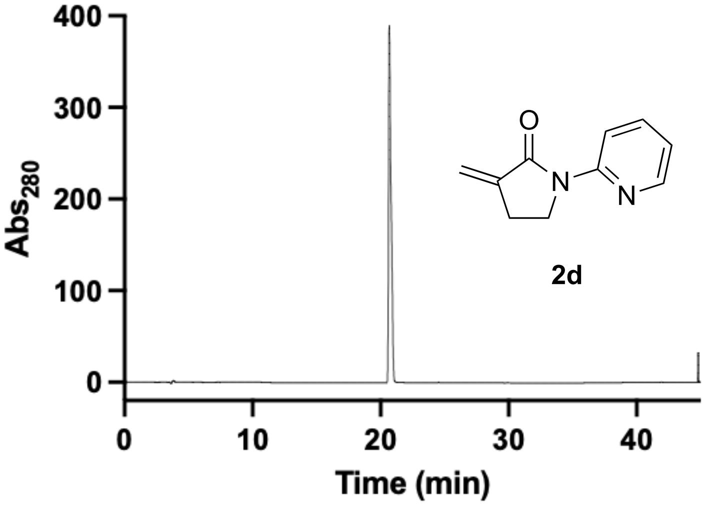

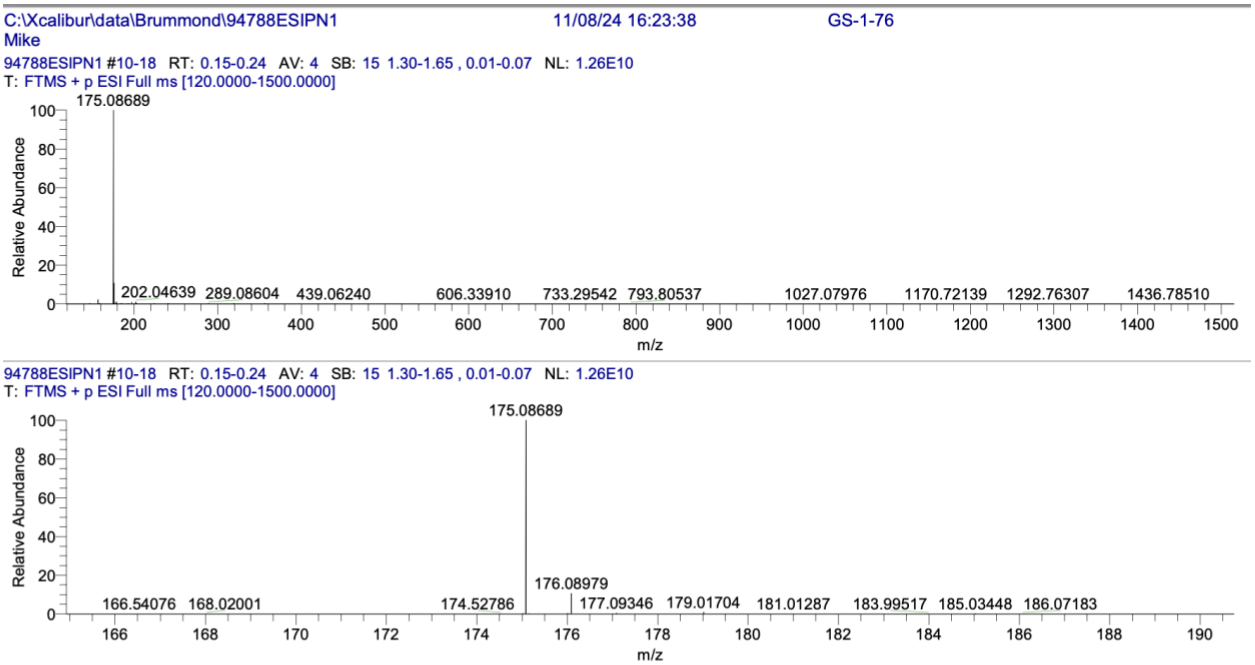

94788ESIPN1#11-17 RT: 0.15-0.24 AV: 4
SB: 15 1.30-1.65 , 0.01-0.07
T: FTMS + p ESI Full ms [120.0000-1500.0000]
m/z= 164.92173-190.74603

m/z	Intensity	Relative	Theo. Mass	Delta (ppm)	Composition
175.08689	12857411584.0	100.00	175.08659	1.74	$C_{10}H_{11}ON_2$

Figure S86. HPLC trace (top) and HRMS ESI-MS spectrum (bottom) for **2d**.

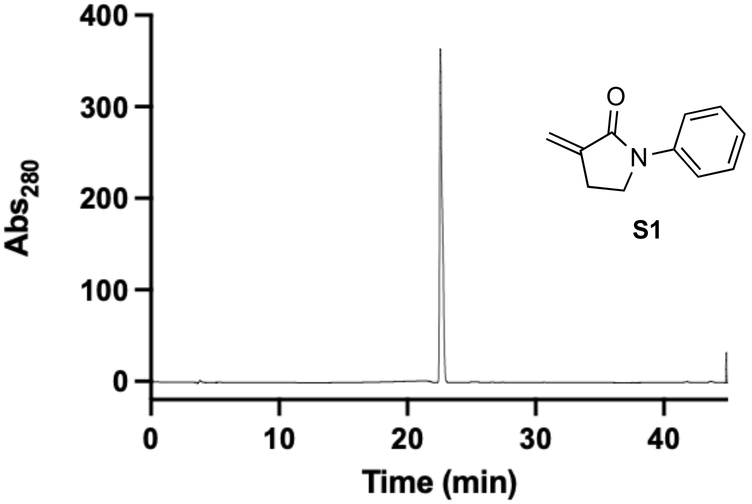

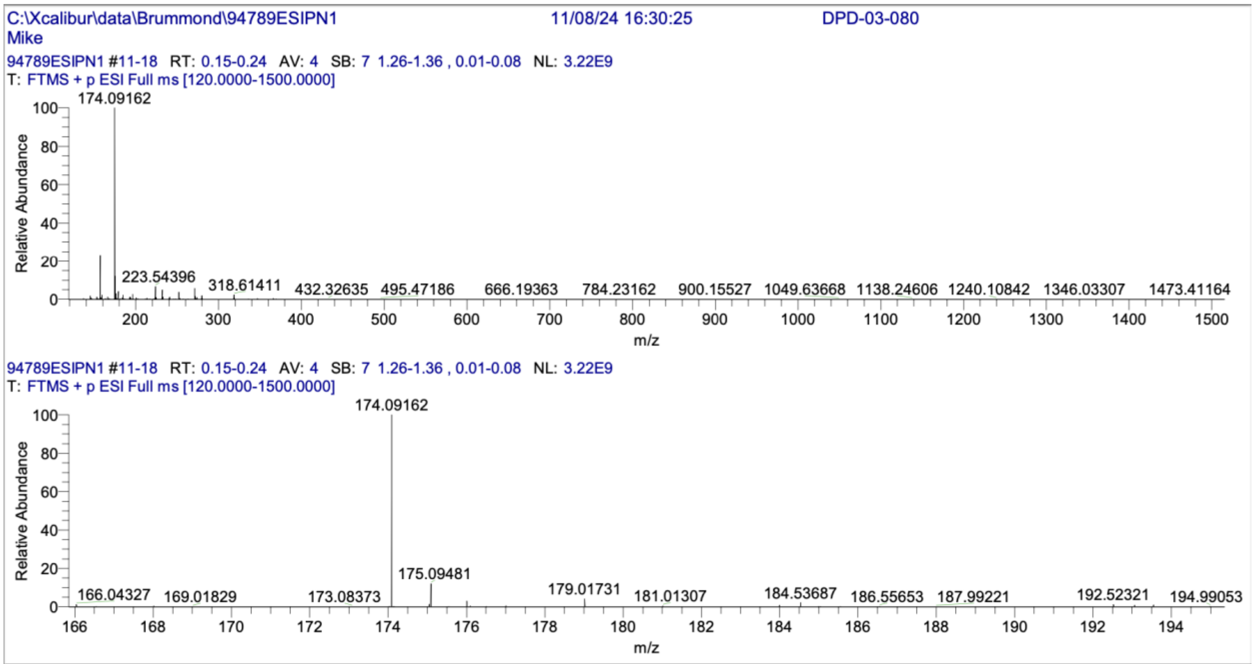

94789ESIPN1#11-17 RT: 0.15-0.24 AV: 4
SB: 7 1.26-1.36 , 0.01-0.08
T: FTMS + p ESI Full ms [120.0000-1500.0000]
m/z= 165.84355-195.35673

m/z	Intensity	Relative	Theo. Mass	Delta (ppm)	Composition
174.09162	3221909504.0	100.00	174.09134	1.59	$C_{11}H_{12}ON$

Figure S87. HPLC trace (top) and HRMS ESI-MS spectrum (bottom) for **S1**.

REFERENCES

1. Meanwell, N. A., Chapter Five – A Synopsis of the Properties and Applications of Heteroaromatic Rings in Medicinal Chemistry. In Advances in Heterocyclic Chemistry, Scriven, E. F. V.; Ramsden, C. A., Eds. Academic Press: 2017; Vol. 123, pp 245-361.
2. Gaussian 16, Revision C.01, M. J. Frisch, G. W. Trucks, H. B. Schlegel, G. E. Scuseria, M. A. Robb, J. R. Cheeseman, G. Scalmani, V. Barone, G. A. Petersson, H. Nakatsuji, X. Li, M. Caricato, A. V. Marenich, J. Bloino, B. G. Janesko, R. Gomperts, B. Mennucci, H. P. Hratchian, J. V. Ortiz, A. F. Izmaylov, J. L. Sonnenberg, D. Williams-Young, F. Ding, F. Lipparini, F. Egidi, J. Goings, B. Peng, A. Petrone, T. Henderson, D. Ranasinghe, V. G. Zakrzewski, J. Gao, N. Rega, G. Zheng, W. Liang, M. Hada, M. Ehara, K. Toyota, R. Fukuda, J. Hasegawa, M. Ishida, T. Nakajima, Y. Honda, O. Kitao, H. Nakai, T. Vreven, K. Throssell, J. A. Montgomery, Jr., J. E. Peralta, F. Ogliaro, M. J. Bearpark, J. J. Heyd, E. N. Brothers, K. N. Kudin, V. N. Staroverov, T. A. Keith, R. Kobayashi, J. Normand, K. Raghavachari, A. P. Rendell, J. C. Burant, S. S. Iyengar, J. Tomasi, M. Cossi, J. M. Millam, M. Klene, C. Adamo, R. Cammi, J. W. Ochterski, R. L. Martin, K. Morokuma, O. Farkas, J. B. Foresman, and D. J. Fox, Gaussian, Inc., Wallingford CT, 2019.
3. Zhao, Y.; Truhlar, D. G. The M06 Suite of Density Functionals for Main Group Thermochemistry, Thermochemical Kinetics, Noncovalent Interactions, Excited States, and Transition Elements: Two New Functionals and Systematic Testing of Four M06-Class Functionals and 12 Other Functionals. *Theor. Chem. Acc.* **2008**, *120* (1–3), 215–241. https://doi.org/10.1007/s00214-007-0310-x.
4. Marenich, A. V.; Cramer, C. J.; Truhlar, D. G. Universal Solvation Model Based on Solute Electron Density and on a Continuum Model of the Solvent Defined by the Bulk Dielectric Constant and Atomic Surface Tensions. *J. Phys. Chem. B* **2009**, *113* (18), 6378–6396. https://doi.org/10.1021/jp810292n.
5. Ribeiro, R. F.; Marenich, A. V.; Cramer, C. J.; Truhlar, D. G. Use of Solution-Phase Vibrational Frequencies in Continuum Models for the Free Energy of Solvation. *J. Phys. Chem. B* **2011**, *115* (49), 14556–14562. https://doi.org/10.1021/jp205508z.
6. Luchini, G.; Alegre-Requena, J. V.; Funes-Ardoiz, I.; Paton, R. S. GoodVibes: Automated Thermochemistry for Heterogeneous Computational Chemistry Data. *F1000Research* **2020**, *9*, 291. https://doi.org/10.12688/f1000research.22758.1.
7. Pracht, P.; Bohle, F.; Grimme, S. Automated Exploration of the Low-Energy Chemical Space with Fast Quantum Chemical Methods. *Phys. Chem. Chem. Phys.* **2020**, *22* (14), 7169–7192. https://doi.org/10.1039/C9CP06869D.
8. Bannwarth, C.; Ehlert, S.; Grimme, S. GFN2-xTB—An Accurate and Broadly Parametrized Self-Consistent Tight-Binding Quantum Chemical Method with Multipole Electrostatics and Density-Dependent Dispersion Contributions. *J. Chem. Theory Comput.* **2019**, *15* (3), 1652–1671. https://doi.org/10.1021/acs.jctc.8b01176.
9. Ehlert, S.; Stahn, M.; Spicher, S.; Grimme, S. Robust and Efficient Implicit Solvation Model for Fast Semiempirical Methods. *J. Chem. Theory Comput.* **2021**, *17* (7), 4250–4261. https://doi.org/10.1021/acs.jctc.1c00471.
10. Erbay, T. G.; Dempe, D. P.; Godugu, B.; Liu, P.; Brummond, K. M. Thiol Reactivity of *N*-Aryl α-Methylene-γ-Lactams: A Reactive Group for Targeted Covalent Inhibitor Design. *J. Org. Chem.* **2021**, *86* (17), 11926–11936. https://doi.org/10.1021/acs.joc.1c01335.
11. NBO Version 3.1, E. D. Glendening, A. E. Reed, J. E. Carpenter, and F. Weinhold.

12. O'Boyle, N. M.; Banck, M.; James, C. A.; Morley, C.; Vandermeersch, T.; Hutchison, G. R. Open Babel: An Open Chemical Toolbox. *J. Cheminformatics* **2011**, *3* (1), 33. https://doi.org/10.1186/1758-2946-3-33.
13. *CYLview, 1.0b*; Legault, C. Y., Université de Sherbrooke, 2009. http://www.cylview.org (accessed 2024-02-17).
14. Chai, J.-D.; Head-Gordon, M. Long-Range Corrected Hybrid Density Functionals with Damped Atom–Atom Dispersion Corrections. *Phys. Chem. Chem. Phys.* **2008**, *10* (44), 6615. https://doi.org/10.1039/b810189b.
15. *IQmol Version 3.1.2*; Andrew Gilbert, 2023. http://iqmol.org/index.html (accessed 2024-02-17).
16. 'electron affinity' in *IUPAC Compendium of Chemical Terminology*, 3rd ed. International Union of Pure and Applied Chemistry; 2006. Online version 3.0.1, 2019. https://doi.org/10.1351/goldbook.E01977.
17. Lian, P.; Johnston, R. C.; Parks, J. M.; Smith, J. C. Quantum Chemical Calculation of pK_as of Environmentally Relevant Functional Groups: Carboxylic Acids, Amines, and Thiols in Aqueous Solution. *J. Phys. Chem. A* **2018**, *122* (17), 4366–4374. https://doi.org/10.1021/acs.jpca.8b01751.
18. Greg Landrum; Paolo Tosco; Brian Kelley; Ric; David Cosgrove; sriniker; Riccardo Vianello; gedeck; NadineSchneider; Gareth Jones; Eisuke Kawashima; Dan N; Andrew Dalke; Brian Cole; Matt Swain; Samo Turk; Aleksandr Savelev; Alain Vaucher; Maciej Wójcikowski; Ichiru Take; Vincent F. Scalfani; Daniel Probst; Kazuya Ujihara; guillaume godin; Axel Pahl; Rachel Walker; Juuso Lehtivarjo; Francois Berenger; strets123; jasondbiggs. Rdkit/Rdkit: Release_2023.09.5, 2024. https://doi.org/10.5281/ZENODO.10633624.
19. Becke, A. D. Density-Functional Thermochemistry. III. The Role of Exact Exchange. *J. Chem. Phys.* **1993**, *98* (7), 5648–5652. https://doi.org/10.1063/1.464913.
20. Grimme, S.; Antony, J.; Ehrlich, S.; Krieg, H. A Consistent and Accurate *Ab Initio* Parametrization of Density Functional Dispersion Correction (DFT-D) for the 94 Elements H-Pu. *J. Chem. Phys.* **2010**, *132* (15), 154104. https://doi.org/10.1063/1.3382344.
21. Liptak, M. D.; Shields, G. C. Accurate pK_a Calculations for Carboxylic Acids Using Complete Basis Set and Gaussian-n Models Combined with CPCM Continuum Solvation Methods. *J. Am. Chem. Soc.* **2001**, *123* (30), 7314–7319. https://doi.org/10.1021/ja010534f.
22. Kelly, C. P.; Cramer, C. J.; Truhlar, D. G. Aqueous Solvation Free Energies of Ions and Ion−Water Clusters Based on an Accurate Value for the Absolute Aqueous Solvation Free Energy of the Proton. *J. Phys. Chem. B* **2006**, *110* (32), 16066–16081. https://doi.org/10.1021/jp063552y.
23. Isse, A. A.; Gennaro, A. Absolute Potential of the Standard Hydrogen Electrode and the Problem of Interconversion of Potentials in Different Solvents. *J. Phys. Chem. B* **2010**, *114* (23), 7894–7899. https://doi.org/10.1021/jp100402x.
24. Thapa, B.; Schlegel, H. B. Density Functional Theory Calculation of pK_a's of Thiols in Aqueous Solution Using Explicit Water Molecules and the Polarizable Continuum Model. *J. Phys. Chem. A* **2016**, *120* (28), 5726–5735. https://doi.org/10.1021/acs.jpca.6b05040.
25. Hansch, Corwin.; Leo, A.; Taft, R. W. A Survey of Hammett Substituent Constants and Resonance and Field Parameters. *Chem. Rev.* **1991**, *91* (2), 165–195. https://doi.org/10.1021/cr00002a004.

26. Verloop, A.; Hoogenstraaten, W.; Tipker, J. Development and Application of New Steric Substituent Parameters in Drug Design. In *Drug Design*; Elsevier, 1976; pp 165–207. https://doi.org/10.1016/B978-0-12-060307-7.50010-9.
27. *Morfeus*; Kjell Jorner, 2021. https://digital-chemistry-laboratory.github.io/morfeus/index.html (accessed 2024-02-17).
28. Lu, T.; Chen, F. Multiwfn: A Multifunctional Wavefunction Analyzer. *J. Comput. Chem.* **2012**, *33* (5), 580–592. https://doi.org/10.1002/jcc.22885.
29. Wolinski, K.; Hinton, J. F.; Pulay, P. Efficient Implementation of the Gauge-Independent Atomic Orbital Method for NMR Chemical Shift Calculations. *J. Am. Chem. Soc.* **1990**, *112* (23), 8251–8260. https://doi.org/10.1021/ja00179a005.
30. *Rowan Scientific*. https://www.rowansci.com (accessed 2025-01-15).
31. Wagen, C.; Wagen, A. Efficient and Accurate pK_a Prediction Enabled by Pre-Trained Machine-Learned Interatomic Potentials. March 8, 2024. https://doi.org/10.26434/chemrxiv-2024-8489b.
32. Anstine, D.; Zubatyuk, R.; Isayev, O. AIMNet2: A Neural Network Potential to Meet Your Neutral, Charged, Organic, and Elemental-Organic Needs. October 12, 2023. https://doi.org/10.26434/chemrxiv-2023-296ch.
33. National Center for Biotechnology Information (2024). PubChem Compound Summary for CID 795, Imidazole. https://pubchem.ncbi.nlm.nih.gov/compound/Imidazole (accessed 2024-02-02).
34. Li, X.; Hu, X.; Jiang, Z.; Sun, Y. Method for detecting related substance imidazole in starting material F of dabigatran etexilate mesylate. CN106033079B. https://patents.google.com/patent/CN106033079B/en#patentCitations (accessed 2024-02-02).
